# Three-Component Approach to Densely Functionalized Trifluoromethyl Allenols by Asymmetric Organocatalysis

**DOI:** 10.1021/jacs.3c02852

**Published:** 2023-04-26

**Authors:** Marie Deliaval, Ramasamy Jayarajan, Lars Eriksson, Kálmán J. Szabó

**Affiliations:** † Department of Organic Chemistry, Arrhenius Laboratory, Stockholm University, SE-106 91 Stockholm, Sweden; ‡ Department of Materials and Environmental Chemistry, Arrhenius Laboratory, Stockholm University, SE-106 91 Stockholm, Sweden

## Abstract

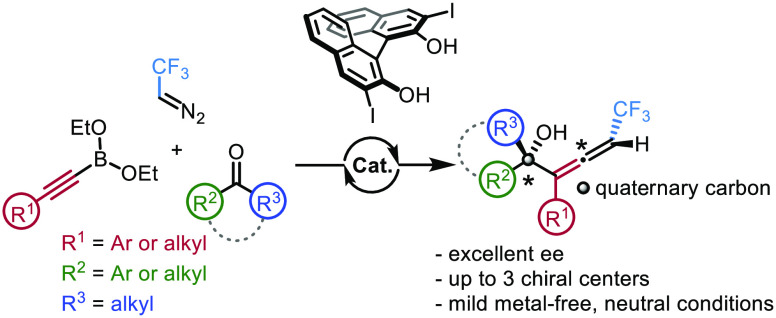

We have developed a new three-component catalytic coupling reaction of alkynyl boronates, diazomethanes, and aliphatic/aromatic ketones in the presence of BINOL derivatives. The reaction proceeds with a remarkably high enantio- and diastereoselectivity (up to three contiguous stereocenters) affording tertiary CF_3_-allenols in a single operational step. The reaction proceeds under mild, neutral, metal-free conditions, which leads to a high level of functional group tolerance.

Chiral allenes represent a very important group of functional molecules.^1−6^ One of the most important groups of allenes are allenols incorporating an allene and an adjacent carbinol functionality.^3,7^ The chiral allenol motif occurs in furanomycin,^8^ leiodolide,^9,10^ allopregnanolone,^11^ adenallene,^12^ and related drugs and drug intermediates (Figure 1a). CF_3_-allenes with potential axial chirality^13−24^ are also attractive targets due to the beneficial pharmacological effects of CF_3_ groups.^25,26^

**Figure 1:**
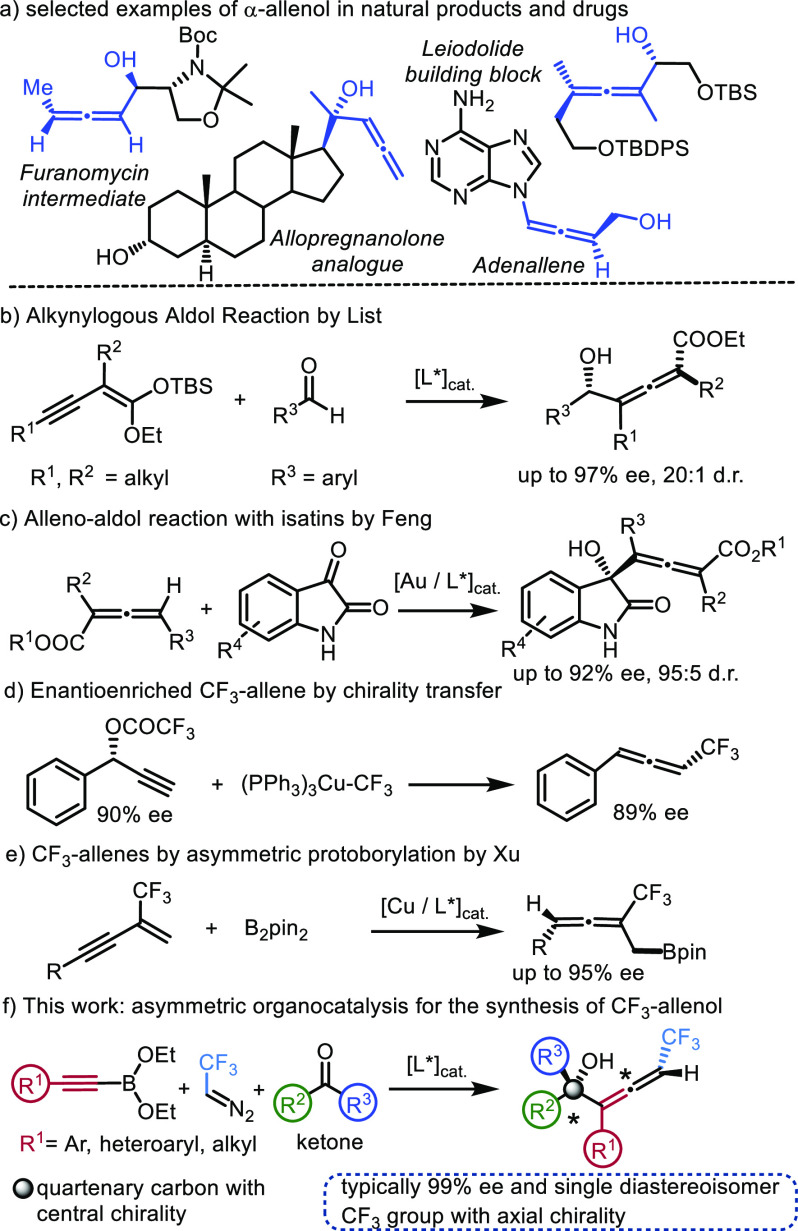
(a) Example of bioactive allenols. (b–f) Asymmetric synthesis of chiral allenols and CF_3_-allenes.

Many excellent methods have appeared for the preparation of allenols.^3,27−34^ An interesting approach is based on application of propargyl boronate species.^35−37^ An excellent organocatalytic method for diastereoselective synthesis of chiral allenols was reported by List and co-workers^38^ (Figure 1b). In addition, several highly selective transition metal catalyzed procedures were reported for the synthesis of allenols.^29,39−43^ A particular challenge is selective synthesis of α-allenols with tertiary alcohol.^44^ Liu, Feng, and co-workers^45^ reported one of the few methods for highly enantio- and diastereoselective synthesis of tertiary allenols (Figure 1c). Later, Liu and co-workers^46^ reported a three-component reaction for asymmetric synthesis of tertiary allenols. Our group^13^ (Figure 1d) and the Bäckvall group^15^ presented synthesis of chiral trifluoromethyl allenes based on chirality transfer. Xu and co-workers^14^ reported a Cu-catalyzed method (Figure 1e). Here, we report a new organocatalytic, three-component coupling (Figure 1f) of alkynyl boronates, trifluoromethyl diazoethane, and ketones to obtain chiral allenols.

The optimal conditions (Table 1, entry 1) using alkynyl ethylboronate **1a** with a CF_3_-diazomethane **2** and acetophenone **3a** in the presence of 30 mol % BINOL derivative **4** resulted in CF_3_-allenol derivative **5a** in 98% ee as a single diastereomer with 71% isolated yield. Decreasing of the catalyst loading led to a decrease of the yields (entries 2, 3). When the bromo analog of **4** was used as catalyst, the yield was 50% (entry 4), while with unsubstituted BINOL the ee dropped to 40% and the yield was decreased to 20% (entry 5). Without catalyst only traces of racemic **5a** was formed (entry 6). When the reaction was performed using isopropyl ester, **5a** was obtained with 97% ee, albeit with a poor yield of 33% (entry 7). At 50 °C the yield decreased to 33% (entry 8). In toluene the selectivity (91% ee) and yield (69%) dropped (entry 9). In the absence of molecular sieves, the selectivity was maintained (98%), but the yield decreased to 27% (entry 10).

**Table 1:**
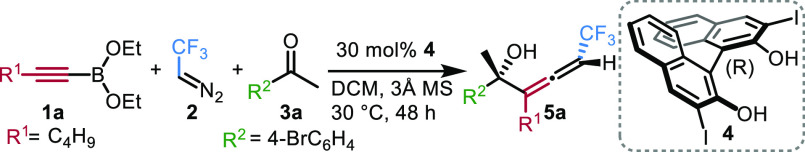
Optimal Conditions for the Synthesis of Trifluoromethyl Allenols^a^

entry	change	yield^b^ (%)	ee (%)
1	no change	71	98
2	20 mol % of catalyst **4**	50	96
3	10 mol % of catalyst **4**	40	95
4	30 mol % (*R*)-3,3′-dibromo-1,1′-bi-2-naphthol instead of **4**	50	92
5	20 mol % (*R*)-1,1′-bi(2-naphthol) instead of **4**	20	40
6	no catalyst	traces	
7	^*i*^Pr instead of Et protected alkynyl boronic ester	33	97
8	reaction at 50 °C instead of 30 °C	33	94
9	toluene instead of DCM	69	91
10	no molecular sieves added	27	98

a Alkynyl boronate **1a** (0.1 mmol), **2** (0.3 mmol), and **3a** (0.15 mmol) were reacted in DCM (0.8 mL) for 48 h at 30 °C.

b Isolated yield and ee over two steps.

Using the optimal conditions, we studied the synthetic scope of the reactions (Figure 2). Unless otherwise stated we detected formation of only a single diastereoisomer of the product. The reaction affording **5a** could be scaled up to 1 mmol scale without significant changing of the selectivity (98% ee). Notably, the NMR yield in this reaction was 91%, but the isolated yield was only 68% due to purification losses (see below). Allenol product **5b** with a ^t^Bu aryl group formed with 61% yield and 95% ee with high diastereoselectivity (d.r. 97:3). In the presence of an even more electron donating substituent (p-OMe) the reaction proceeds with 88% ee, but the resulting compound is vinyl-allene **5c**, indicating dehydration of the allenol product. When, the m-OMe-substituted acetophenone analog was used, product **5d** was formed without any OH group elimination. Changing the *n*-Bu substituent of the alkynyl boronate to cyclohexyl gave **5e** with 76% yield and 99% ee. Diaryl-substituted allenol **5f** was obtained with excellent selectivity (99% ee) and in good yield (70%). Trifluormethyl phenyl-substituted alkyne was found to be less reactive than its phenyl analogue. The reaction at elevated temperature gave **5g** in 68% yield with excellent selectivity. Tertiary-butyl dimethylsilyl (TBDMS)-substituted alkynyl boronate reacted readily with bromo acetophenone (**3a**), affording **5h** with 99% ee and 66% yield. Other derivatives with substitution at the para position of the phenyl group reacted similarly to **3a**, giving **5i**–**k** with 99% ee and 56–61% yield. Dimethyl sulfone substituted acetophenone also reacted with 99% ee and in 60% yield. Product **5l** was solid, and we were able to determine its absolute configuration, by X-ray diffraction. Accordingly, the axial chiral part of the molecule has an *R* configuration, while the quaternary carbon has an *S* configuration. The other chiral allenol products were oils resisting crystallization, and therefore their X-ray analysis was not possible. Thus, the configurations were tentatively assigned based on the absolute configuration of **5l.**

**Figure 2:**
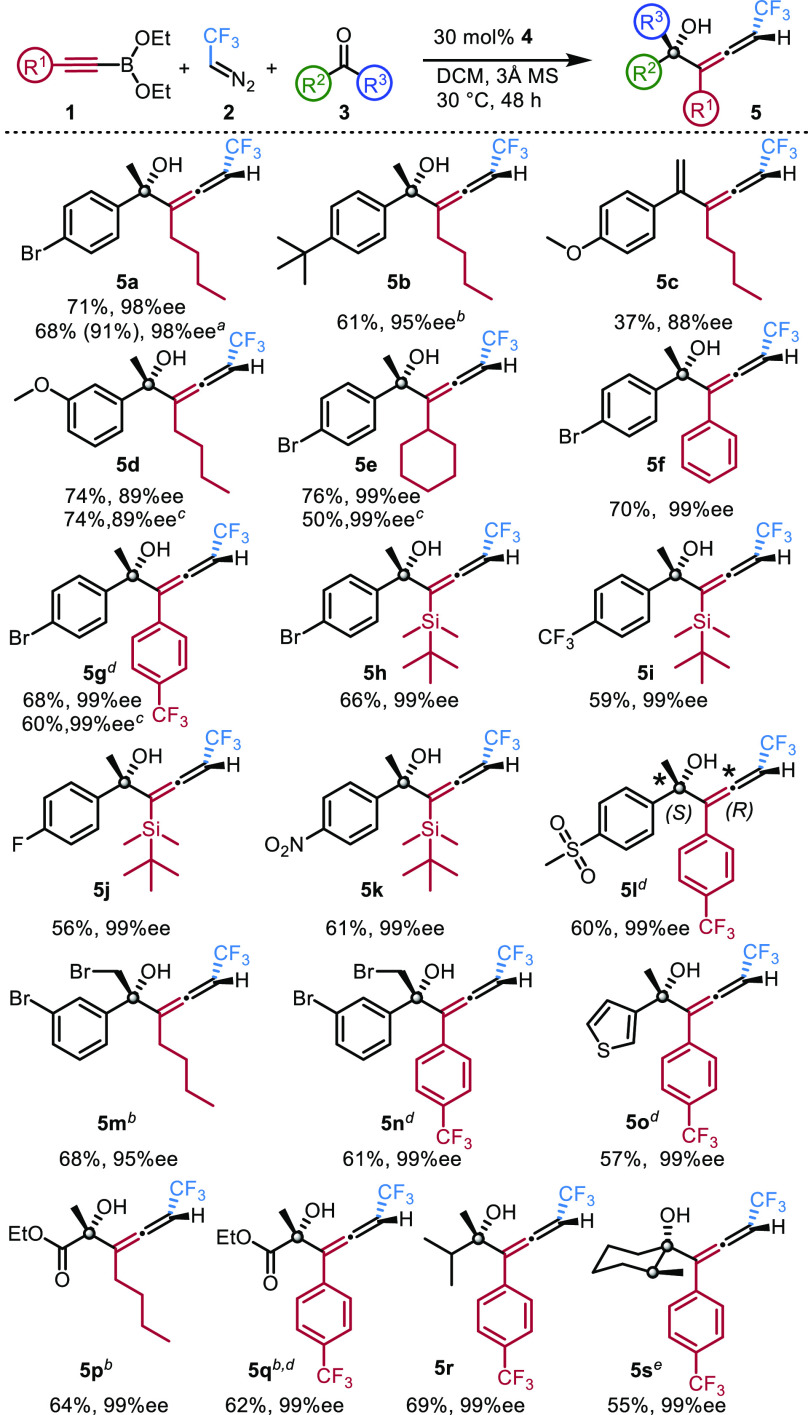
Synthetic scope of the three-component coupling. For standard reaction conditions see footnote *a* in Table 1. Unless otherwise stated a single diastereomer was obtained and the isolated yields are given. ^*a*^1 mmol scale; The yield in parentheses is ^19^F NMR yield. ^*b*^The diastereoselectivity is 97:3 (**5b**, **5m**), 91:9 (**5p**), and 96:4 (**5q**). ^*c*^20 mol % catalyst **4** was used. ^*d*^At 40 °C. ^*e*^At room temperature.

Dibrominated tertiary allenols **5m**–**n** were obtained with excellent selectivity (95% and 99% ee) and 61–68% yield. Heteroaromatic allenol **5o** was obtained in high selectivity and in good yield (57%). Ethyl pyruvate could be used as ketone component, affording **5p** and **5q** in good yields (62–64%) and high enantioselectivity (99%). However, in this reaction formation of the two diastereomeric forms of **5p** and **5q** was observed in 91:9 and 96:4 ratio, respectively. The lower diastereoselectivity is due to the relatively small difference of the size of the substituents of the keto group in ethyl pyruvate.

Application of isopropyl methyl ketone afforded **5r** with 99% ee and in 69% yield. For preparation of **5s** an excess of racemic methyl cyclohexanone was used. Interestingly, **5s** was formed with three contiguous stereocenters as a single stereoisomer (99% ee) indicating a kinetic resolution process. We have also briefly studied the possibilities of using other stabilized diazomethane derivatives than CF_3_-diazoethane **2**. TMS-diazomethane derivative **6** (Figure 3a) proved to be less reactive than **2**; thus, we conducted the reaction at 60 °C in toluene. The coupling of **6** with various alkenyl boronate substrates **1** (R^1^ = 4-CF_3_C_6_H_4_, C_4_H_9_) and ketones (R^2^ = 4-NO_2_C_6_H_4_, 4-CF_3_C_6_H_4_, 3-BrC_6_H_4_, R^3^ = CH_3_, CH_2_Br) proceeded with high selectivity (95–98% ee) formation of a single diastereomer for allenols **7a**–**d**. Stabilized diazo-carbonyl, -ester, or -cyano precursors (instead of **2** or **6**) cannot be used in asymmetric homologation, as with these types of diazo compounds and organoboronates the Hooz reaction^47−49^ takes place, which occurs without formation of a C–B stereocenter.

**Figure 3:**
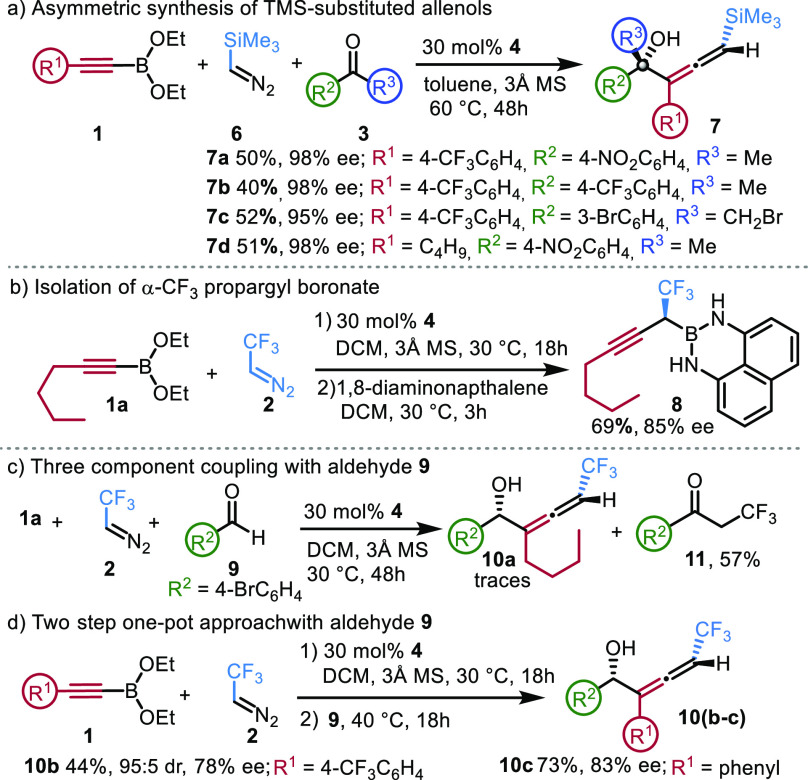
(a) Extension to TMS-diazo methane. (b, c) Control reactions. (d) Reactions with aldehydes in two operational steps.

A control reaction (Figure 3b) was conducted without ketone. In this reaction α-CF_3_ propargylboronic acid was formed by homologation of alkynyl boronate **1a**. The product was air sensitive, and therefore the boron was protected by diamino-naphthalene to give **8**, which could be purified by silica gel chromatography. Propargyl boronate **8** is much less stable^47,50^ than its alkyl, benzyl, or allyl analogs. Nevertheless, we determined the structure and optical purity (85% ee) of **8**. The absolute configuration of **8** was tentatively assigned to be *S* based on the X-ray structures obtained for the allyl,^50^ benzyl, and methyl-cyclopropyl^47^ analogs, which all have *S* configuration. Unfortunately, the low stability and resistance to crystallization prevented the X-ray analysis of **8**.

When the ketone component of the reaction was replaced by aldehyde **9**, a complex reaction mixture was formed containing only traces of the expected allenyl product **10a** (Figure 3c). The main component was CF_3_-ketone **11**,^51^ which was formed by the reaction of **2** and **9**. Carreira and Morandi^52^ reported that **2** reacts with aldehydes in the presence of Lewis acids to give CF_3_-ketones, such as **11**. Apparently, in this reaction boronate **1a** served as Lewis acid. Notably, under the optimized reaction conditions **2** did not react with ketones. With a slight modification of the optimized conditions, even aldehydes could be used for one-pot synthesis of allenols (Figure 3d). First, alkynyl boronate **1** was reacted with **2** at 30 °C for 18 h; then, aldehyde **9** was added, affording secondary allenol **10b** with 78% ee, acceptable diastereoselectivity (95:5), and 44% yield. The yield (73%) and the selectivity (83% ee) were slightly improved when phenyl-substituted alkenyl boronate was used as substrate (**10c**).

Previous studies^47,50^ on asymmetric homologation of alkyl, aryl, and vinylboronates with diazomethane derivatives in the presence of catalytic amounts of BINOL derivatives indicated that the reaction proceeds via transesterification of the organoboronate derivative, such as **1**, with **4** (Figure 4). According to ^1^H NMR studies (SI), EtOH was formed in the reaction of ethylboronate **1a** and **4**, which indicated formation of BINOL boronate **12** in an equilibrium process. This reactive^53^ chiral boron ester **12** forms an ate complex with trifluoromethyl diazomethane **2**, which undergoes 1,2-borotropic rearrangement to give chiral α-CF_3_ propargylboronate **13**. Similar reactions for synthesis of α-CF_3_ organoboronates have been reported in the literature.^47,50,54,55^ Isolation of propargylboronate **8** (Figure 3b) indicates that the reactions proceeded via intermediate **14**. According to our results (Table 1, entries 1, 4, 5), the C–X bond length of the 3,3′-substituents of BINOL is particularly important for the stereoinduction, and application of the iodo derivative **4** gives the highest selectivity for the reaction. The next step is reaction of **14** with ketone **3** to give α-tertiary allenol derivative **15**. As mentioned above, there are several examples in the literature^35−37^ for reaction of propargyl boronates with aldehydes to give secondary allenols. However, we were not able to find a single report about the reaction of propargyl boronates with ketones to give tertiary allenols. The presence of the BINOL moiety^56^ and the CF_3_ substituent probably increases the Lewis acidity of the boron. The reaction with ketones, similarly to aldehydes, is supposed to proceed via a Zimmerman–Traxler-type TS.^37,57,58^ Based on previous studies the enantioselectivity is determined in the **12** → **14** process, while^47,50,53^ the diastereoselectivity is controlled by the chirality transfer in the allenylboration step **14 → 15**. Finally, ethanolysis of **15** leads to formation of the boric ester of product **5** (**16**) and regeneration of the catalyst.

**Figure 4:**
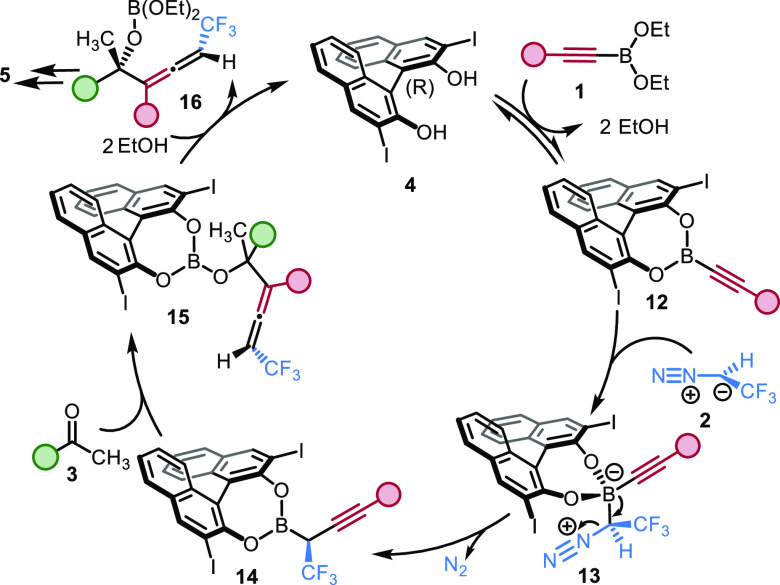
Suggested mechanism of the three-component reaction.

The above two-step, homologation–allenylboration model explains a number of features of the reaction. The stereoselectivity over two steps was remarkably high, typically 98–99% ee. As mentioned above, the CF_3_-propargyl boronates (**8**) are very unstable compounds. In the three-component coupling with ketones these species underwent allenylboration immediately after formation, leading to excellent selectivities. However, when the allenylboronate compounds were isolated (Figure 3b) or reacted with aldehydes in an operationally two-step process (Figure 3d), partial racemization or other side reactions occurred over time (18–30 h, 30 °C), which led to a drop of the ee to 80–85%. The difference of the ee obtained for **8** and **5a** may also be explained by the different stability/reactivity of the reaction intermediates, such as diastereomeric BINOL esters **14**.

The yields are typically in the range of 60–70% over two steps. The purification losses were usually due to the low polarity of the CF_3_ and in particular of the TMS allenes, which makes the separation difficult from the unreacted unpolar reaction components (**2**/**6** and **3**/**9** were used in excess, **4** in 20–30 mol %).

Esterification of catalyst **4** and solvolysis of intermediate **15** turning over the catalytic cycle occur by release and consumption of EtOH (Figure 4). Probably small structural changes of the alkynyl boronate substrate may affect these processes, slowing down the catalytic reactions. This may explain that formation of certain products (such as **5d, 5g**) proceeded with similar yields and selectivities using either 20 or 30 mol % catalyst, while other substrates gave lower yields (**5a**, **5e**) with maintained selectivities using lower catalyst loadings.

In summary, we have developed a new three-component reaction for synthesis of tertiary chiral allenols with an axially chiral CF_3_ group. The reaction is based on organocatalytic homologation of alkynyl boronates, which undergoes in situ allenylboration with ketones. The mild, neutral, and metal-free conditions of the presented three-component reaction allow application of aliphatic ketones, and many substituents, such as bromo, nitro, and methanesulfonyl, are tolerated. This reaction gives an operationally simple access to densely functionalized chiral tertiary allenols, which can be employed as building blocks in natural product synthesis and drug design.^1,2,7^

## Supporting Information Available

Experimental procedures, characterization data, and NMR spectra of compounds (PDF)
