# Supplementary material for: Three-Component Approach to Densely Functionalized Trifluoromethyl Allenols by Asymmetric Organocatalysis
Source: J Am Chem Soc. 2023 Apr 26;145(18):10001–6. doi: 10.1021/jacs.3c02852 (PMC10176480; doi:10.1021/jacs.3c02852)
Supplement: Supplementary file 1 — ja3c02852_si_001.pdf [file ja3c02852_si_001.pdf]

Supplementary materials for

Three-Component Approach to Densely Functionalized Trifluoromethyl Allenols by Asymmetric Organocatalysis

Marie Deliaval[1†], Ramasamy Jayarajan[1†], Lars Eriksson[2], Kálmán J. Szabó[1*]

[1]Department of Organic Chemistry, Arrhenius Laboratory, Stockholm University, SE-106 91 Stockholm, Sweden. [2]Department of Materials and Environmental Chemistry, Arrhenius Laboratory, Stockholm University, SE-106 91 Stockholm, Sweden. [†]These authors contributed equally to this work.

Table of contents

1. General information 2
2. Experimental Procedures and Spectral data 3
   Preparation of alkynyl boronate (**1**): 3
   General procedure A: Three-component synthesis of chiral trifluoromethyl allenols 5 3
   General procedure B: Synthesis of chiral allenol derivative (**7**) using TMS-diazomethane 4
   Preparation of BDan derivative: 29
   Reaction with aldehyde with two operational steps 30
   Control experiment – for tracing intermediate **12** 33
3. References 34
4. NMR Spectra: 35

Materials and Methods

1. General information

All reactions were carried out under argon atmosphere using screw cap reaction tubes unless otherwise stated. All alkynes, ketones and borates were purchased from commercial sources. In prior to be use triethyl and triisopropyl borates were freshly distilled as well as phenylacetylene and cyclohexylacetylene. CF_3-diazocompound was synthesised in DCM or toluene by the reported procedure[1] and was stored in the freezer over the activated molecular sieves (3Å pellets). TMS-diazocompound was purchased from commercial source (2 M in Et_2O/ 0.6 M in hexane). Iodo-BINOL **4** was prepared by a reported procedure.[2] *n*BuLi was purchased and used freshly. For column chromatography, silica gel (35-70 microns) was used. TLC was performed using aluminium backed plates pre-coated (0.25 mm) with Silica Gel 60 F254 with a suitable solvent system and was visualized using UV fluorescence and/or developed with permanganate stain or PMA stain. All isolated compounds were characterized by NMR spectroscopy using Bruker 400 MHz and 500 MHz spectrometers. Copies of the NMR spectra can be found in the end of this Supporting Material. All ^{1}H NMR were reported in unit parts per million (ppm), and were measured relative to the signals for residual chloroform (7.26 ppm) in the deuterated solvent, unless otherwise stated. All ^{13}C were reported in ppm relative to deuterochloroform (77.16 ppm), unless otherwise stated. ^{29}Si NMR was performed for the compounds **5h**-**5k** and **7a-d**. All crude NMR analysis were performed by using trifluorotoluene as the internal standard. High resolution mass spectrometry (HRMS) was obtained using a Bruker Daltonics microTOF Mass Spectrometer with an ESI techniques. Optical rotation was measured on an AUTOPOL IV polarimeter. X-ray diffraction was performed using a Bruker D8 ADVANCE kappa geometry diffractometer equipped with a Bruker Photon 100 CMOS detector.

Chiral SFC analysis: All the enantiomeric excess analysis was performed with chiral SFC unless otherwise stated. Chiral SFC analysis was performed using Chiralpak IA-3, Chiralpak IB N-3, Chiralpak IC-3 and Chiralcel OJ-3, eluting with MeOH/CO_2 or ^{i}PrOH/CO_2 (SFC) and monitored by DAD (Diode Array Detector). Retention times (t_R) are quoted in minutes.

Chiral GC analysis: Chiral GC was performed for products **5i** and **5j** using a GC-MS (Chiraldex β-6TBDM 30 m column, helium gas carrier at 1 mL/min, constant pressure).

Chiral HPLC analysis: Chiral HPLC analysis were performed for products **5c, 5q** and **7b** using Chiralcel OD-H and Chiralpak AD-H columns, eluting with ^{i}PrOH/hexane.

2. Experimental Procedures and Spectral data

Preparation of alkynyl boronate (1):

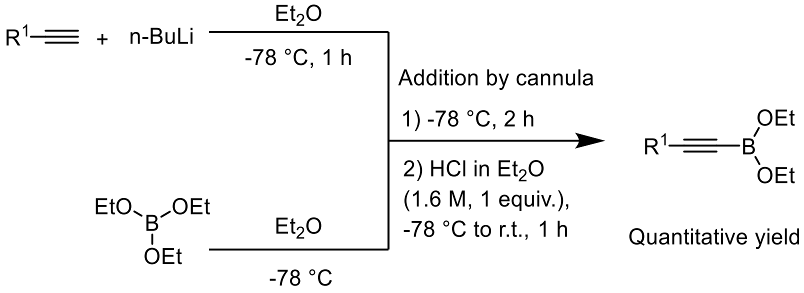

Alkynyl boronates were prepared by using reported procedure with slight modification.[3] An oven-dried round-bottom flask was vacuum and backfilled with argon three times. Alkyne (2 mmol, 1 equiv.) and dry diethyl ester (4 mL) were added. The solution was cooled down to -78°C and *n*-BuLi (2 mmol, 1.0 equiv., 1.6 M in hexane) was added. The reaction mixture was stirred 1h at -78°C. In parallel, another round bottom flask charged with argon was added freshly distilled triethyl borate (2 mmol) and dry diethyl ether (4 mL). The solution was cooled down to -78°C and the lithiated acetylide from the first flask was slowly added to the triethyl borate solution using canula. The reaction mixture was maintained at -78°C for 2h. Then, anhydrous HCl (1 mL, 1 equiv., 2.0 M in diethyl ether) was added, and the reaction mixture was stirred 15 min at -78°C, and became a clear solution. The cooling bath was removed and the mixture was stirred for an additional 45 min and a white precipitate was formed. In the meantime, oven-dried third flask was charged with Argon. A phase separator multi-layered with filter paper was connected with needle and the pistol on the top. The crude mixture was carefully filtered into the third flask under argon. The excess diethyl ether was evaporated by vacuum and back filled with argon. The solvent free alkynyl boronate was made into 0.25 M solution either in DCM or in toluene under argon and was allowed to be stored in the freezer up to 3-4 weeks. This stock solution was used for the reactions without further purification.

General procedure A: Three-component synthesis of chiral trifluoromethyl allenols 5

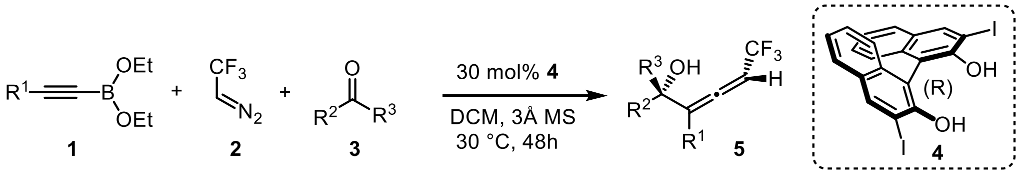

A reaction tube was charged with catalyst **4** (0.03 mmol) and was brought into the glovebox. Alkynyl boronic ester **1** (0.1 mmol, 400 µL of 0.25 mol/L stock solution in DCM), molecular sieves (20 mg), CF_3-diazomethane **2** (0.3 mmol) and ketone **3** (0.15 mmol) were added sequentially. The total volume was maintained to 0.8 mL. The reaction mixture was stirred at 30 °C for 48 hours in the fume hood. The product **5** was isolated by silica gel chromatography.

Preparation of racemates: The racemates of allenol derivatives were prepared by following the above procedure except that the equimolar mixture of (R) and (S) catalyst **4** was used. A minor deviation of a 50/50 ratio of the S/R enantiomers may occur due to weighting errors of the (R) and (S) enantiomers of the catalyst and/or the different reactivity and stability of the stereoisomeric reaction intermediates, such as **14** (see Figure 4 in the paper).

General procedure B: Synthesis of chiral allenol derivative (7) using TMS-diazomethane

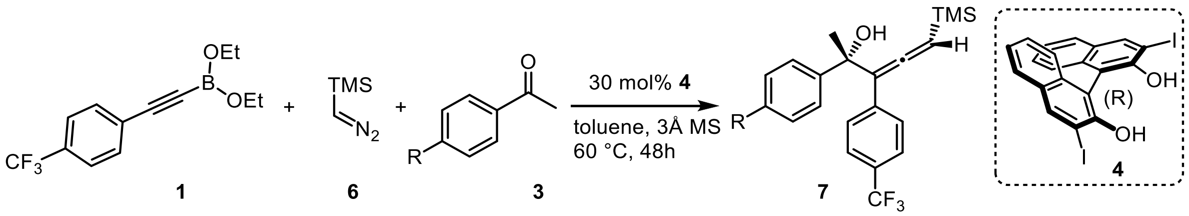

A reaction tube was charged with catalyst **4** (0.03 mmol) and was brought into the glovebox. Diethyl ((4-(trifluoromethyl)phenyl)ethynyl)boronate **1** (0.1 mmol, 400 µL of 0.25 mol/L stock solution in toluene), molecular sieves (20 mg), TMS-diazomethane **6** (0.3 mmol, 2.0 M solution in Et_2O) and ketone **3** (0.15 mmol) were added sequentially. The total volume was maintained to 0.8 mL with dry toluene. The reaction mixture was stirred at 60 °C for 48 hours in the fume hood. The reaction mixture was quenched with saturated NH_4Cl solution. The product **7** was isolated by silica gel chromatography.

(S)-2-(4-bromophenyl)-3-((R)-3,3,3-trifluoroprop-1-en-1-ylidene)heptan-2-ol (5a)

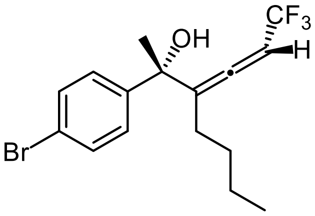

This compound was obtained according to general procedure A. Product **5a** was isolated in 71% yield (25.6 mg, 0.071 mmol) as a colourless oil by silica gel chromatography using pentane/DCM 2:1 as eluent and R_f = 0.4 with PMA stain. Product **5a** was isolated in 68% yield (24.5 mg, 0.068 mmol) with 1 mmol scale reaction. 91% NMR yield was observed. Crude NMR of the reaction mixture was determined by ^{19}F NMR using trifluorotoluene as internal standard.

^{1}H NMR (500 MHz, $CDCl_3$) δ 7.47 (d, J = 8.6 Hz, 2H), 7.31 (d, J = 8.6 Hz, 2H), 5.68 (qt, J = 5.7, 3.4 Hz, 1H), 2.07 – 1.96 (m, 1H), 1.96 (s, 1H), 1.80 – 1.73 (m, 1H), 1.66 (s, 3H) 1.36 – 1.21 (m, 4H), 0.83 (t, J = 7.2 Hz, 3H). **^{13}C NMR (126 MHz, $CDCl_3$) δ** 202.1 (q, J = 5.8 Hz), 144.0, 131.5, 127.2, 123.0 (q, J = 270.6 Hz), 121.5, 120.4, 89.8 (q, J = 38.7 Hz), 75.2, 30.3, 29.7, 27.0, 22.3, 13.9. **^{19}F NMR (377 MHz, $CDCl_3$) δ** -60.2 (d, J = 5.9). **HRMS (pos. ESI) m/z:** calcd for $C_{16}H_{18}BrF_3ONa$ [M+Na] 385.0385. Found 385.0368, also found 387.0351.
$[\alpha]_D^{27}$ = -97.20 (c 0.25, $CHCl_3$).

Determination of *ee* by Chiral SFC: Daicel CHIRALCEL IA-3, 25°c, 0.46 cm ϕ, 10 cm column, 2% MeOH in CO_2, flow rate: 1.2 mL/min; t_R: 2.7 min (major enantiomer), 3.8 min (minor enantiomer), *ee* = 98%.

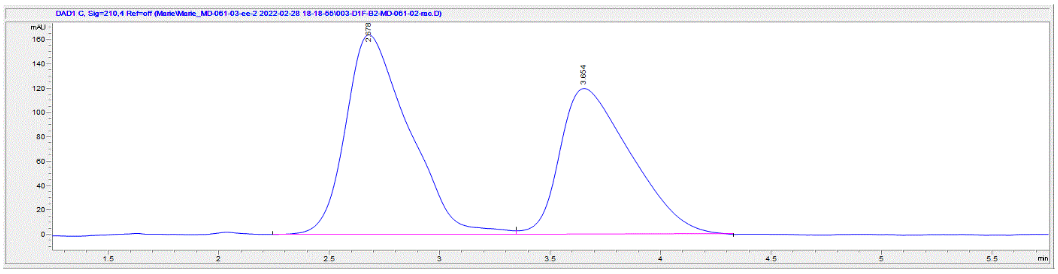

Signal 3: DAD1 C, Sig=210,4 Ref=off

Peak #	RetTime [min]	Type	Width [min]	Area [mAU*s]	Height [mAU]	Area %
1	2.678	BV	0.2886	3281.18823	164.58774	54.9074
2	3.654	VB	0.3354	2694.66577	120.25574	45.0926
Totals :				5975.85400	284.84348	

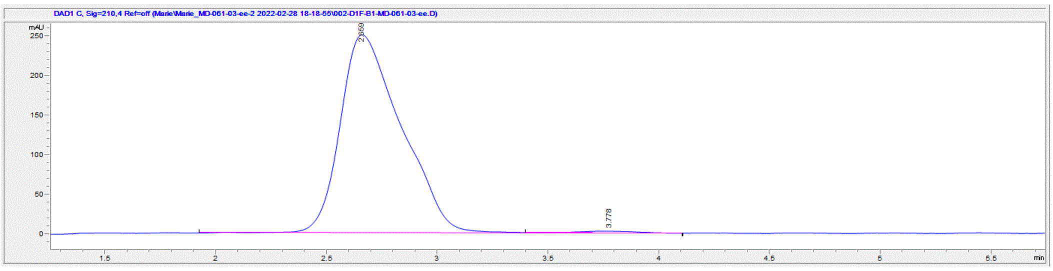

Signal 3: DAD1 C, Sig=210,4 Ref=off

Peak #	RetTime [min]	Type	Width [min]	Area [mAU*s]	Height [mAU]	Area %
1	2.659	VV R	0.2831	4850.79004	251.41524	98.9959
2	3.778	VB E	0.2354	49.20065	2.50188	1.0041
Totals :				4899.99069	253.91712	

(*S*)-2-(4-(tert-butyl)phenyl)-3-((*R*)-3,3,3-trifluoroprop-1-en-1-ylidene)heptan-2-ol (5b)

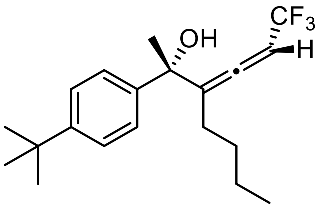

This compound was obtained according to the above general procedure A. Product **5b** was isolated in 61% yield (20.6 mg, 0.061 mmol) in a diastereomeric ratio of 97:3 (based on ^{19}F NMR) as yellow oil by silica gel chromatography using pentane/DCM 2:1 as eluent and PMA as TLC stain. R_f = 0. 23 blue stain.

^{1}H NMR (500 MHz, $CDCl_3$) δ 7.35 (d, *J* = 2.73 Hz, 4H), 5.67 (dtd, *J* = 9.19, 5.65, 3.17 Hz, 1H), 2.07–2.00 (m, 1H), 1.95 (s, 1H), 1.87–1.80 (m, 1H), 1.69 (s, 3H), 1.37–1.34 (m, 2H) 1.32 (s, 9H), 1.29–1.27 (m, 2H), 0.82 (t, *J* = 7.20 Hz, 3H). **^{13}C NMR (126 MHz, $CDCl_3$) δ** 202.1 (q, *J* = 5.8 Hz), 150.4, 141.9, 125.3, 125.0, 123.1 (q, *J* = 270.5 Hz), 120.9, 89.5 (q, *J* = 38.7 Hz), 75.3, 34.6, 31.5, 29.9, 29.7, 27.1, 22.3, 13.9.
^{19}F NMR (377 MHz, $CDCl_3$) δ -60.2 ppm (*J* = 5.6 Hz).
HRMS (pos. ESI) m/z: calcd for $C_{20}H_{27}F_3NaO$ [M+Na] 363.1906. Found 363.1907.
$[\alpha]_D^{27}$ = -58.4 (*c* 0.25, $CHCl_3$).

Determination of *ee* by Chiral SFC: Daicel CHIRALCEL OJ, 25°c, 0.46 cm ϕ, 10 cm column, 2% MeOH in CO_2, flow rate: 1.2 mL/min; t_R: 5.06 min (major enantiomer), 6.53 min (minor enantiomer), *ee* (major enantiomer) = 95%

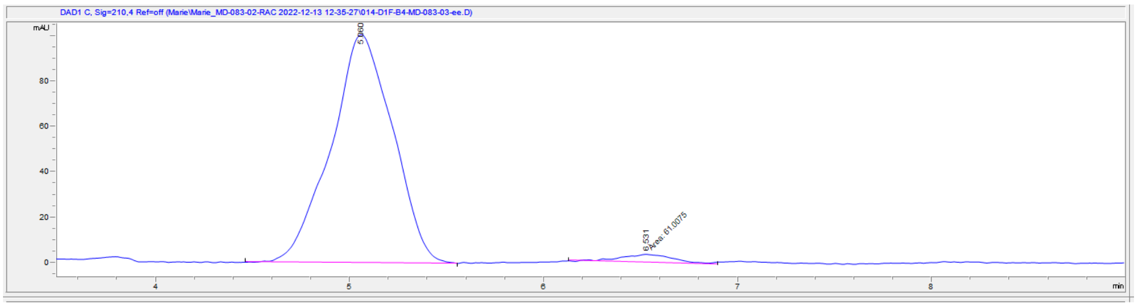

Signal 3: DAD1 C, Sig=210,4 Ref=off

Peak #	RetTime [min]	Type	Width [min]	Area [mAU*s]	Height [mAU]	Area %
1	5.060	BB	0.3135	2235.75903	100.50077	97.3438
2	6.531	MM	0.3017	61.00750	3.37002	2.6562
Totals :				2296.76653	103.87079	

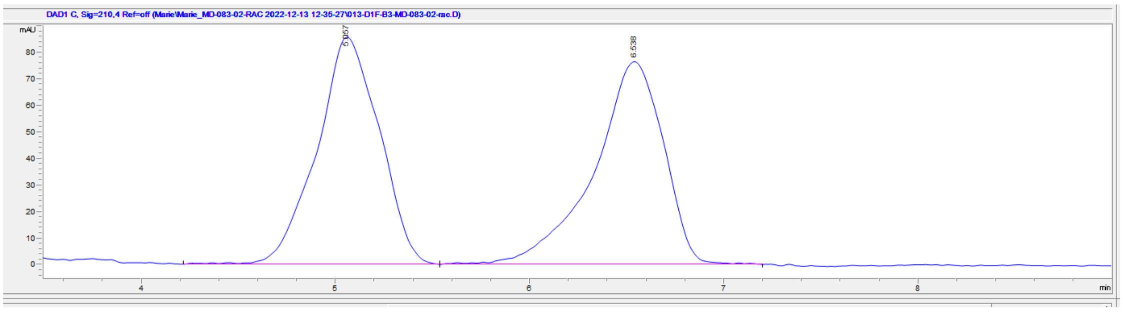

Signal 3: DAD1 C, Sig=210,4 Ref=off

Peak #	RetTime [min]	Type	Width [min]	Area [mAU*s]	Height [mAU]	Area %
1	5.057	VB R	0.3131	1915.18604	85.57468	50.1442
2	6.538	VV R	0.3549	1904.17102	76.37643	49.8558
Totals :				3819.35706	161.95111	

(*R*)-1-methoxy-4-(3-(3,3,3-trifluoroprop-1-en-1-ylidene)hept-1-en-2-yl)benzene (5c)

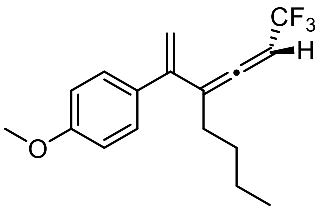

This compound was obtained according to the above general procedure A. Product **5c** was isolated in 37% yield (11 mg, 0.037 mmol) as volatile yellow oil by silica gel chromatography using pentane/DCM 10:1 as eluent and PMA as TLC stain. R_f = 0.48 blue stain.

^{1}H NMR (500 MHz, CDCl$_3$) δ 7.24 (d, *J* = 8.7 Hz, 2H), 6.85 (d, *J* = 8.6 Hz, 2H), 5.45-5.41 (m, 1H), 5.29 (s, 1H), 5.26 (s, 1H), 3.81 (s, 3H), 2.32-2.28 (m, 2H), 1.48 (p, *J* = 7.3 Hz, 2H), 1.39 (p, *J* = 7.1 Hz, 2H), 0.92 (t, *J* = 7.3 Hz, 3H).

^{13}C NMR (126 MHz, CDCl$_3$) δ 206.5 (d, J = 5.7 Hz), 159.5, 143.8, 132.5, 129.1, 122.0 (q, *J* = 270.1 Hz), 118.8, 114.7, 113.6, 87.2 (q, *J* = 38.7 Hz), 55.4, 30.0, 29.6, 22.4, 14.0. **^{19}F NMR (377 MHz, CDCl$_3$) δ** – 60.2 (d, *J* = 5.8 Hz).**HRMS (pos. ESI) m/z:** calcd for $C_{17}H_{19}F_3NaO$ [M+Na] 319.1280. Found 319.1257.

$[\alpha]_D^{27}$ = 7.0 (*c* 0.25, $CHCl_3$).

Determination of *ee* by Chiral HPLC: Daicel CHIRALCEL OD-H, 25°c, 0.46 cm ϕ, 25 cm column, 1% *i*PrOH in Hexane, flow rate: 1.0 mL/min; t_R: 4.7 min (minor enantiomer), 5.8 min (major enantiomer), *ee* = 88%

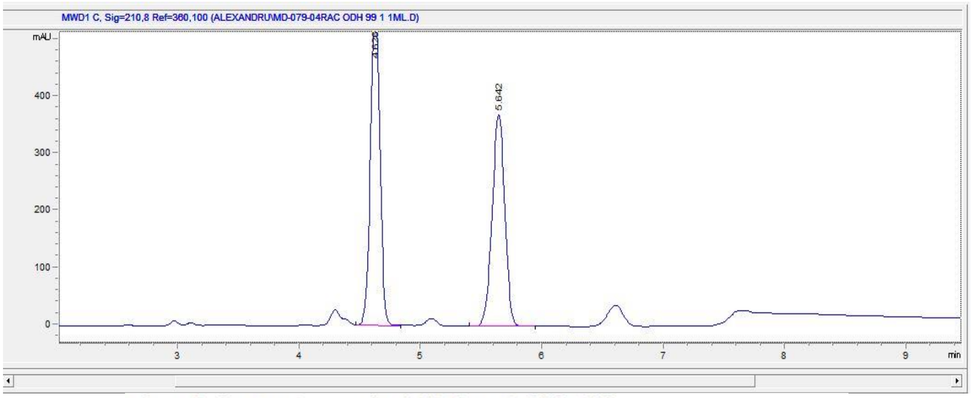

Signal 3: MWD1 C, Sig=210,8 Ref=360,100

Peak #	RetTime [min]	Type	Width [min]	Area [mAU*s]	Height [mAU]	Area %
1	4.626	VV	0.0815	3081.59204	584.17096	53.3417
2	5.642	BV	0.1126	2695.48169	371.59241	46.6583
Totals :				5777.07373	955.76337	

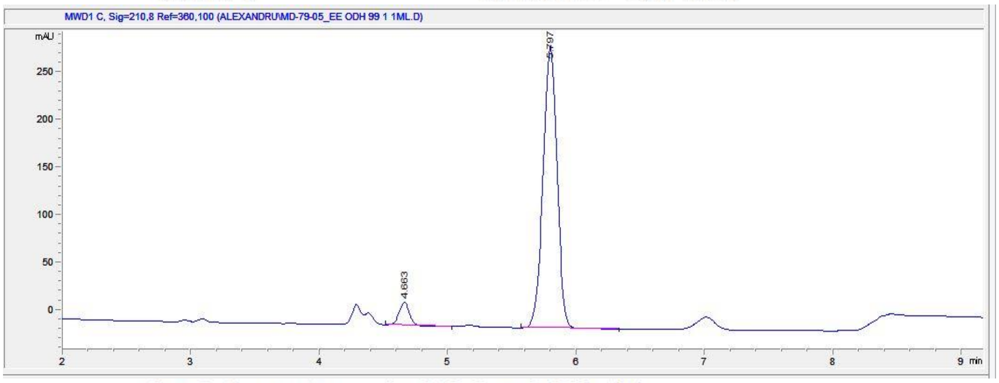

Signal 3: MWD1 C, Sig=210,8 Ref=360,100

Peak #	RetTime [min]	Type	Width [min]	Area [mAU*s]	Height [mAU]	Area %
1	4.663	VV	0.0890	138.46719	24.11334	5.7790
2	5.797	BB	0.1185	2257.56274	297.51895	94.2210
Totals :				2396.02994	321.63229	

(*S*)-2-(3-methoxyphenyl)-3-((*R*)-3,3,3-trifluoroprop-1-en-1-ylidene)heptan-2-ol (5d)

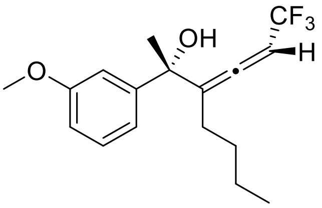

This compound was obtained according to the above general procedure A. Product **5d** was isolated in 74% yield (23.2 mg, 0.074 mmol) as yellow **oil** by silica gel chromatography using pentane/DCM 2:1 as eluent and PMA as TLC stain. R_f = 0.55 blue stain. Product **5d** was isolated in 74% yield with 20 mol% catalyst **4.**

^{1}H NMR (500 MHz, CDCl$_3$) δ 7.26 (t, *J* = 7.95 Hz, 1H), 7.03–7.02 (m, 1H), 6.99–6.98 (m, 1H), 6.82 (ddd, 8.15, 2.59, 0.91, 1H), 5.68 (dtd, *J* = 9.18, 5.74, 3.42, 1H), 3.82 (s, 3H), 2.07–2.00 (m, 1H), 1.98 (s,1H), 1.85–1.78 (m, 1H), 1.68 (s, 3H), 1.36–1.31 (m, 4H), 0.82 (t, *J* =7.21, 3H).

^{13}C NMR (126 MHz, CDCl$_3$) δ 202.1 (q, *J* = 5.8 Hz), 159.8, 146.7, 129.5, 123.1 (q, *J* = 270.7 Hz), 120.7, 117.7, 112.7, 111.3, 89.6 (q, *J* = 38.6 Hz), 75.4, 55.4, 30.1, 29.7, 27.1, 22.3, 13.9.

^{19}F NMR (377 MHz, CDCl$_3$) δ – 60.2 (*J* = 5.6 Hz).

HRMS (pos. ESI) m/z: calcd for $C_{17}H_{21}F_3NaO_2$ [M+Na] 337.1386. Found 337.1386.

$[\alpha]_D^{27}$ = -51.2 (*c* 0.25, CHCl$_3$).

Determination of *ee* by Chiral SFC: Daicel CHIRALCEL IB, 25°c, 0.46 cm ϕ, 25 cm column, 3% MeOH in CO_2, flow rate: 1.0 mL/min; t_R: 9.53 min (minor enantiomer), 10.44 min (major enantiomer), *ee* (major enantiomer) = 89%

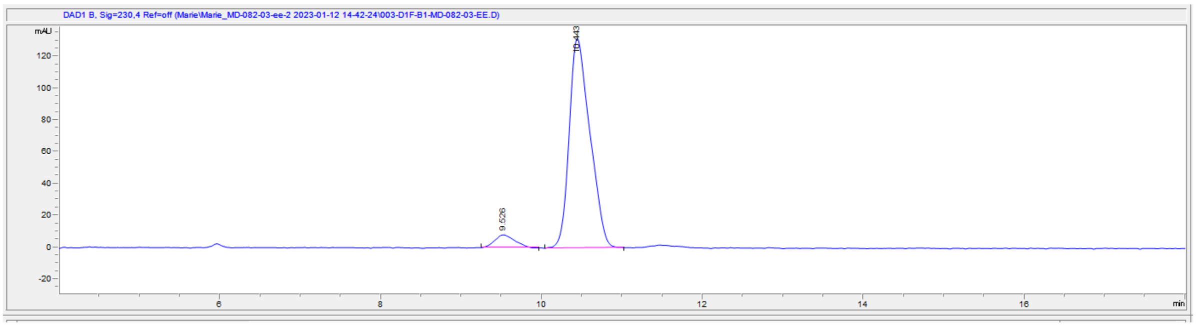

Signal 2: DAD1 B, Sig=230,4 Ref=off

Peak #	RetTime [min]	Type	Width [min]	Area [mAU*s]	Height [mAU]	Area %
1	9.526	BB	0.2338	141.99867	8.04891	5.6750
2	10.443	BB	0.2633	2360.16577	131.58835	94.3250
Totals :				2502.16444	139.63726	

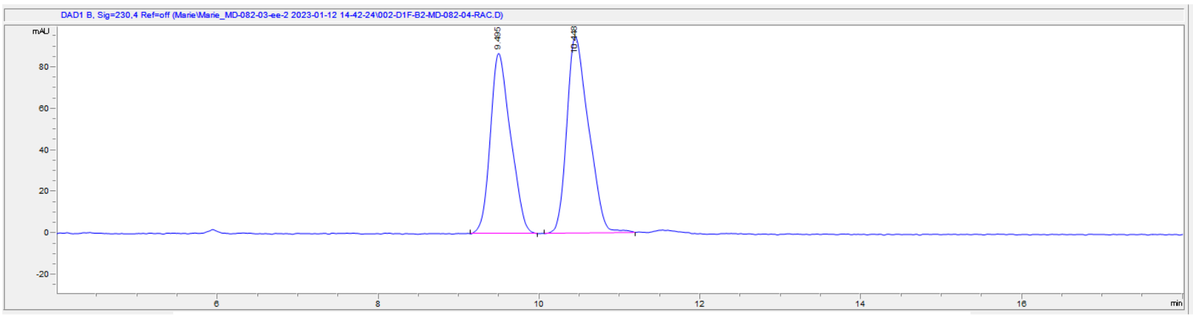

Peak #	RetTime [min]	Type	Width [min]	Area [mAU*s]	Height [mAU]	Area %
1	9.495	BB	0.2583	1519.03235	86.73018	46.0604
2	10.448	BV R	0.2777	1778.88416	94.48399	53.9396
Totals :				3297.91650	181.21416	

(2*S*,4*R*)-2-(4-bromophenyl)-3-cyclohexyl-6,6,6-trifluorohexa-3,4-dien-2-ol (5e)

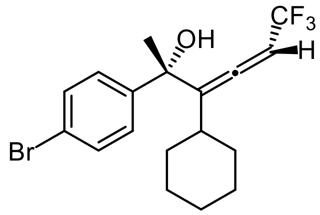

This compound was obtained according to the above general procedure A. Product **5e** was isolated in 76% yield (29.7 mg, 0.076 mmol) as colourless oil by silica gel chromatography using pentane/DCM 2:1 as eluent and PMA as TLC stain. R_f = 0.25 blue stain. Product **5e** was isolated in 50% yield with 20 mol% catalyst **4**.

^{1}H NMR (400 MHz, CDCl$_3$) δ 7.46 (d, *J* = 8.56 Hz, 2H), 7.32 (d, *J* = 8.60 Hz, 2H), 5.70 (q, *J* = 5.62 Hz, 1H), 1.93 (s, 1H), 1.83-1.75 (m, 2H), 1.70-1.66 (m. 1H), 1.64 (s, 3H), 1.59-1.57 (m, 2H), 1.40-1.37 (m, 1H), 1.18-0.98 (m, 5H).**^{13}C NMR (101 MHz, CDCl$_3$) δ** 203.0 (q, *J* = 5.8 Hz), 143.9, 131.4, 127.3, 1254, 123.0 (q, *J* = 270.7 Hz), 121.4, 90.2 (q, *J* = 38.6 Hz) 75.4, 37.5, 34.7, 33.8, 30.7, 26.6, 26.5, 25.8. **^{19}F NMR (377 MHz, CDCl$_3$) δ** -60.1 (d, *J* = 5.7 Hz). **HRMS (pos. ESI) m/z:** calcd for $C_{18}H_{20}BrF_3ONa$ [M+Na] 411.0542. Found 411.0540, also found 431.0510. $[\alpha]_D^{27}$ = –80.8 (*c* 0.25, $CHCl_3$).

Determination of *ee* by Chiral SFC: Daicel CHIRALCEL IF-3, 25°c, 0.46 cm ϕ, 10 cm column, 2% ^{i}PrOH in CO_2, flow rate: 1.2 mL/min; t_R: 3.5 min (minor enantiomer), 5.8 min (major enantiomer), *ee* = 99%.

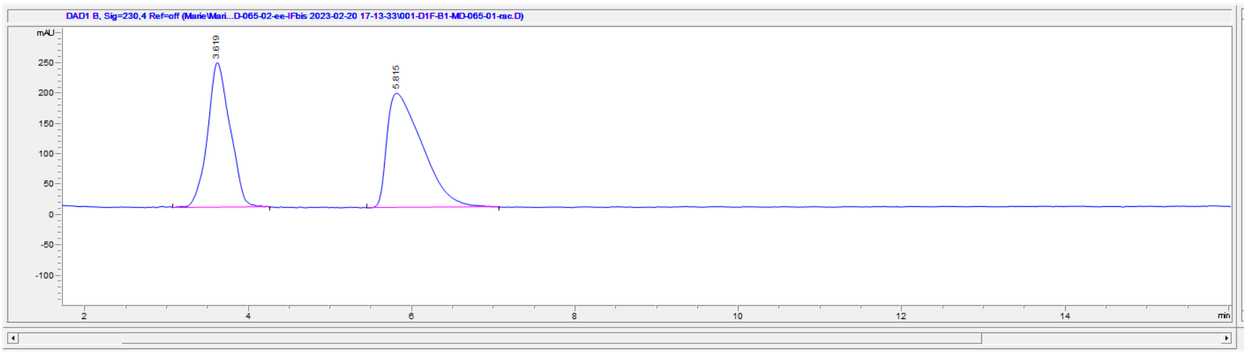

Signal 2: DAD1 B, Sig=230,4 Ref=off

Peak #	RetTime [min]	Type	Width [min]	Area [mAU*s]	Height [mAU]	Area %
1	3.619	VV R	0.2667	4586.77734	238.19077	44.6746
2	5.815	VV R	0.4017	5680.31299	188.40561	55.3254
Totals :				1.02671e4	426.59637	

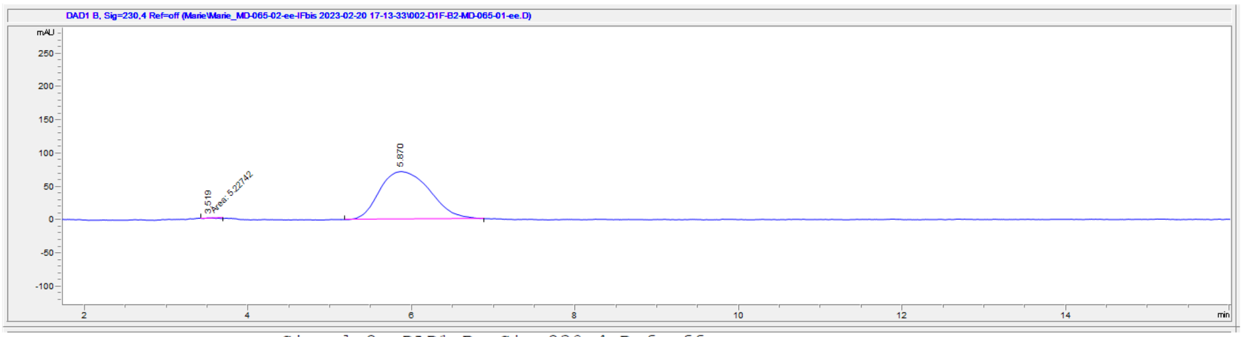

Signal 2: DAD1 B, Sig=230,4 Ref=off

Peak #	RetTime [min]	Type	Width [min]	Area [mAU*s]	Height [mAU]	Area %
1	3.519	MM	0.0994	5.22742	8.76201e-1	0.1690
2	5.870	VB R	0.5041	3088.09863	72.03970	99.8310
Totals :				3093.32605	72.91590	

(2*S*,4*R*)-2-(4-bromophenyl)-6,6,6-trifluoro-3-phenylhexa-3,4-dien-2-ol (5f)

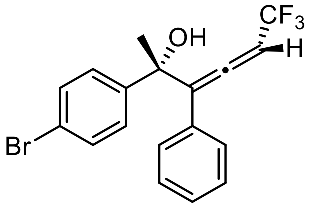

This compound was obtained according to general procedure A. Product **5c** was isolated in 70% yield (26.7 mg, 0.07 mmol) as colorless oil by silica gel chromatography using pentane/DCM 2:1 as eluent and R_f = 0.35.

^{1}H NMR (400 MHz, CDCl$_3$) δ 7.51-7.48 (m, 2H), 7.43-7.39 (m, 2H), 7.26-7.22 (m, 3H), 7.15-7.12 (m, 2H), 5.90 (q, *J* = 5.7 Hz, 1H), 2.23 (s,1H), 1.74 (s, 3H). **^{13}C NMR (101 MHz, CDCl$_3$) δ** 204.9 (q, *J* = 5.8 Hz), 144.7, 131.8, 131.7, 128.8, 128.7, 127.2, 122.8 (q, *J* = 271.3 Hz), 121.6, 120.4, 89.3 (q, *J* = 39.0 Hz), 75.5, 32.0. **^{19}F NMR (377 MHz, CDCl$_3$) δ** -60.0 (d, *J* = 5.5 Hz). **HRMS (pos. ESI) m/z:** calcd for $C_{18}H_{14}BrF_3ONa$ [M+Na] 405.0072. Found 405.0076, also found 407.0055. $[\alpha]_D^{27}$ = -105.20 (*c* 0.25, CHCl$_3$).

Determination of *ee* by Chiral SFC: Daicel CHIRALCEL OJ-3, 25°c, 0.46 cm ϕ, 10 cm column, 2% MeOH in CO$_2$, flow rate: 1.2 mL/min; t_R: 3.9 min (minor enantiomer), 4.7 min (major enantiomer), *ee* = 99%.

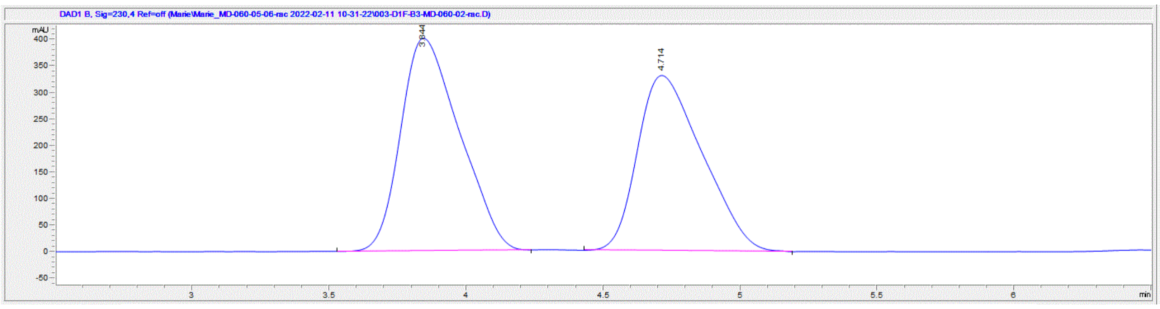

Signal 2: DAD1 B, Sig=230,4 Ref=off

Peak #	RetTime [min]	Type	Width [min]	Area [mAU*s]	Height [mAU]	Area %
1	3.844	BB	0.2243	6004.87451	401.87744	52.4255
2	4.714	BB	0.2458	5449.24512	331.50262	47.5745
Totals :				1.14541e4	733.38007	

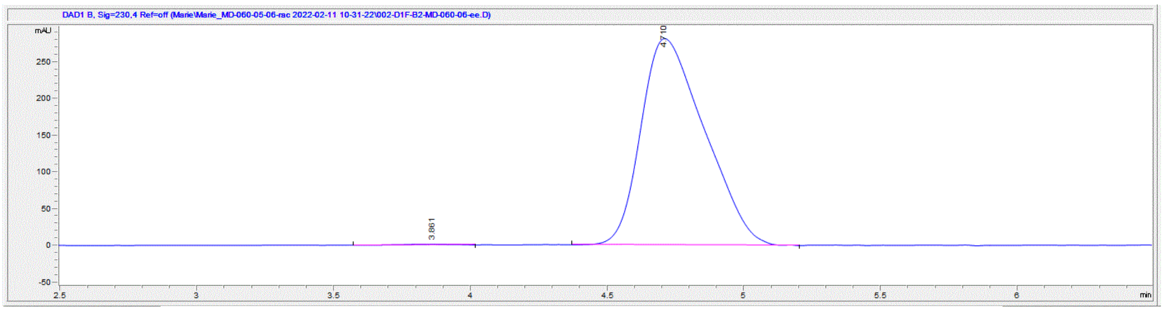

Signal 2: DAD1 B, Sig=230,4 Ref=off

Peak #	RetTime [min]	Type	Width [min]	Area [mAU*s]	Height [mAU]	Area %
1	3.861	BV	0.1733	16.20131	1.20183	0.3543
2	4.710	BV R	0.2407	4556.24658	281.70468	99.6457
Totals :				4572.44789	282.90652	

(2*S*,4*R*)-2-(4-bromophenyl)-6,6,6-trifluoro-3-(4-(trifluoromethyl)phenyl)hexa-3,4-dien-2-ol (5g)

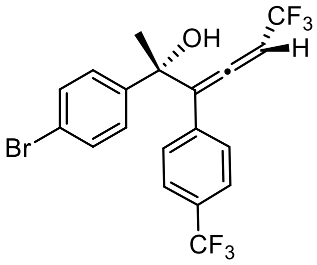

This compound was obtained according to general procedure A except that the reaction temperature is 40 °C. Product **5d** was isolated in 68% yield (30.5 mg, 0.068 mmol) as a colourless oil by silica gel chromatography using pentane/DCM (3:1 to 2:1) as eluent and R_f = 0.34 (pentane/DCM, 2:1). Product **5g** was isolated in 60% yield with 20 mol% catalyst **4.**

^{1}H NMR (400 MHz, CDCl₃) δ 7.49 (d, *J* = 8.5 Hz, 4H), 7.39 (d, *J* = 8.5 Hz, 2H), 7.30 (d, *J* = 8.2 Hz, 2H), 5.97 (q, *J* = 5.6 Hz, 1H), 2.19 (s, 1H), 1.76 (s, 3H). **^{13}C NMR (101 MHz, $CDCl_3$)** δ 205.1 (q, *J* = 5.6 Hz), 144.0, 135.7, 131.9, 130.6 (q, *J* = 32.7 Hz), 129.2, 127.1, 125.5 (q, *J* = 3.7 Hz), 124.0 (q, *J* = 272.2 Hz), 122.5 (q, *J* = 271.4 Hz), 121.9, 119.7, 90.1 (q, *J* = 39.2 Hz), 75.6, 32.3. **^{19}F NMR (377 MHz, $CDCl_3$)** δ -59.9 (d, *J* = 5.7 Hz), -62.9. **HRMS (pos. ESI) m/z:** calcd for $C_{19}H_{13}BrF_6ONa$ [M+Na] 472.9946. Found 472.9933, also found 474.9891. $[\alpha]_D^{27}$ = -74.4 (*c* 0.25, $CHCl_3$).

Determination of *ee* by Chiral SFC: CHIRALCEL OJ-3, 25 °C, 0.3 cm ϕ, 15 cm column, 2% MeOH in CO_2, flow rate 0.8 mL/min; t_R: 3.6 min (minor enantiomer), 4.6 min (major enantiomer), *ee* = 99 %.

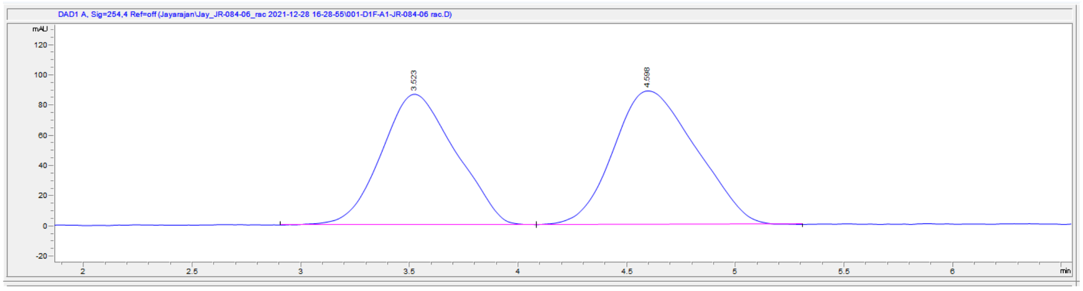

Signal 1: DAD1 A, Sig=254,4 Ref=off

Peak #	RetTime [min]	Type	Width [min]	Area [mAU*s]	Height [mAU]	Area %
1	3.523	BB	0.3516	2056.73242	86.44348	46.7125
2	4.598	BB	0.3975	2346.22949	88.43492	53.2875
Totals :				4402.96191	174.87840	

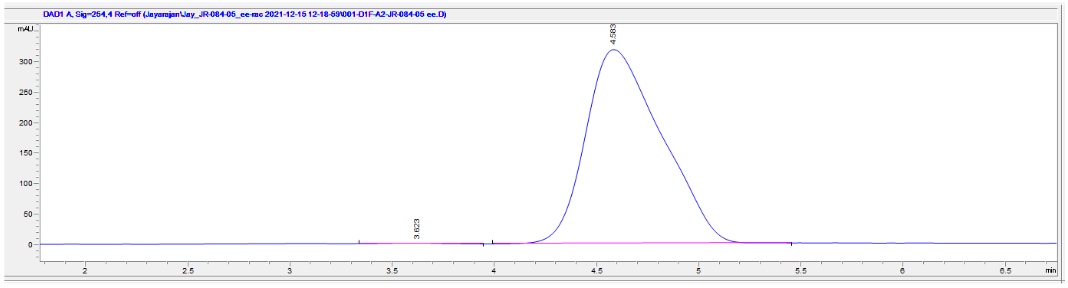

Signal 1: DAD1 A, Sig=254,4 Ref=off

Peak #	RetTime [min]	Type	Width [min]	Area [mAU*s]	Height [mAU]	Area %
1	3.623	BB	0.2297	19.65809	1.05343	0.2380
2	4.583	BB	0.3868	8239.76172	319.55551	99.7620
Totals :				8259.41981	320.60895	

(2*S*,4*S*)-2-(4-bromophenyl)-3-(tert-butyldimethylsilyl)-6,6,6-trifluorohexa-3,4-dien-2-ol (5h)

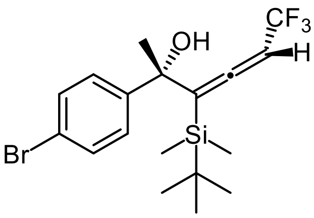

This compound was obtained according to general procedure A. Product **5e** was isolated in 66% yield (28.0 mg, 0.066 mmol) as colorless oil by silica gel chromatography using pentane/DCM 5:1 as eluent and R_f = 0.26.

^{1}H NMR (500 MHz, $CDCl_3$) δ 7.45(d, *J* = 8.5 Hz, 2H), 7.32 (d, *J* = 8.5 Hz, 2H), 5.35(q, *J* = 6.0 Hz, 1H), 2.01 (s, 1H), 1.70 (s, 3H), 0.89 (s, 9H), -0.01 (s, 3H), -0.06 (s, 3H). **^{13}C NMR (126 MHz, $CDCl_3$) δ** 204.9 (q, *J* = 5.6 Hz), 145.8, 131.3, 127.0, 123.9 (q, *J* = 270.0 Hz), 121.2, 112.4, 83.1 (q, *J* = 38.8 Hz), 76.3 (q, *J* = 2.2 Hz), 33.4, 27.1, 18.1, -3.9, -4.1. **^{19}F NMR (377 MHz, $CDCl_3$) δ** -58.7 (d, *J* = 6.2 Hz). **^{29}Si (99 MHz, $CDCl_3$) δ** 4.1 (q, *J* = 1.8 Hz). **HRMS (pos. ESI) m/z:** calcd for $C_{18}H_{24}BrF_3OSiNa$ [M+Na] 443.0624. Found 443.0613, also found 445.0602. $[\alpha]_D^{27}$ = -89.200 (*c* 0.25, $CHCl_3$).

Determination of *ee* by Chiral SFC: Daicel CHIRALCEL OJ-H, 25°c, 0.3 cm *ϕ*, 15 cm column, 5% MeOH in CO_2, flow rate: 1.2 mL/min; t_R: 7.3 min (major enantiomer), 8.1 min (minor enantiomer), *ee* = >99 %.

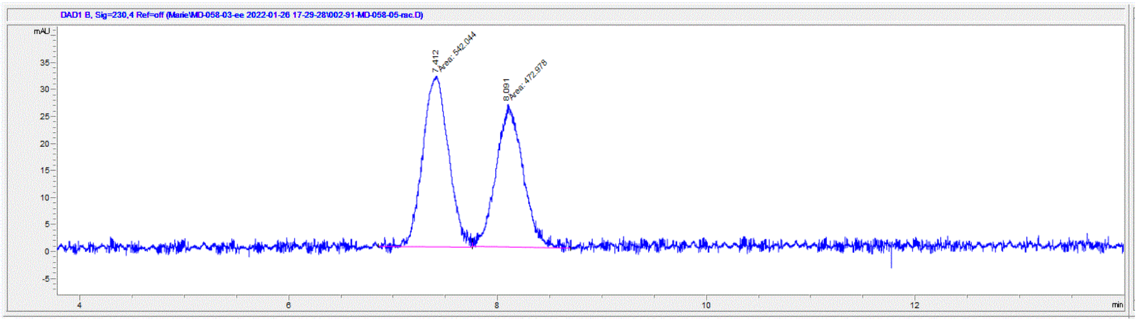

Signal 2: DAD1 B, Sig=230,4 Ref=off

Peak #	RetTime [min]	Type	Width [min]	Area [mAU*s]	Height [mAU]	Area %
1	7.412	MM	0.2865	542.04376	31.53307	53.4022
2	8.091	MM	0.2990	472.97754	26.36070	46.5978
Totals :				1015.02130	57.89376	

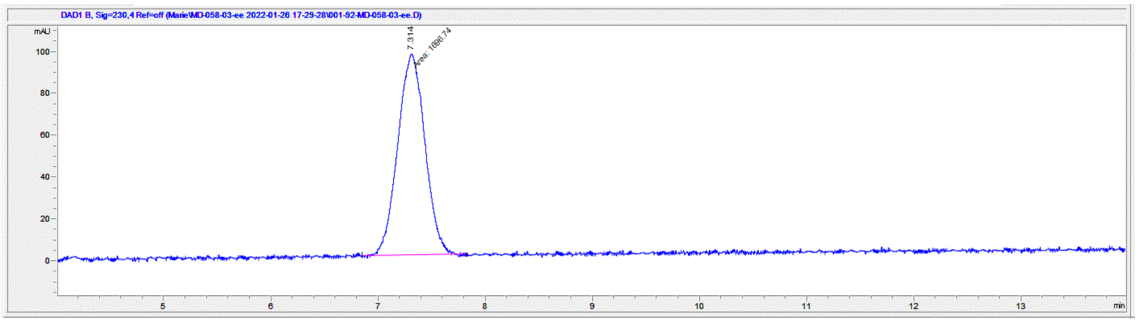

Signal 2: DAD1 B, Sig=230,4 Ref=off

Peak #	RetTime [min]	Type	Width [min]	Area [mAU*s]	Height [mAU]	Area %
1	7.314	MM	0.2944	1696.73657	96.04147	100.0000
Totals :				1696.73657	96.04147	

(2*S*,4*S*)-3-(tert-butyldimethylsilyl)-6,6,6-trifluoro-2-(4-(trifluoromethyl)phenyl)hexa-3,4-dien-2-ol (5i)

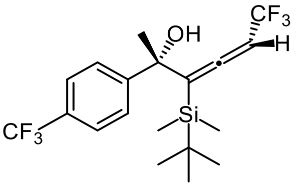

This compound was obtained according to general procedure A. Product **5f** was isolated in 59% yield (24.0 mg, 0.058 mmol) as colorless oil by silica gel chromatography using pentane/DCM 2:1 as eluent and R_f = 0.61 with PMA stain.

^{1}H NMR (400 MHz, $CDCl_3$) δ 7.61-7.55 (m, 4H), 5.37 (q, *J* = 6.0 Hz, 1H), 2.04 (s,1H), 1.74 (s, 3H), 0.90 (s, 9H), 0.00 (s, 3H), -0.07 (s, 3H). **^{13}C NMR (126 MHz, $CDCl_3$) δ** 204.7 (q, *J* = 5.5 Hz), 150.6, 129.6 (q, *J* = 32.4 Hz), 125.6, 125.3 (q, *J* = 3.7 Hz), 124.3 (q, *J* = 271.9 Hz), 123.9 (q, *J* = 270.0 Hz), 112.3, 83.2 (q, *J* = 38.9 Hz), 76.5 (d, *J* = 2.2 Hz), 33.5, 27.1, 18.1, -3.9, -4.1. **^{19}F NMR (377 MHz, $CDCl_3$) δ** -58.7 (d, *J* = 6.1 Hz), -62.4. **^{29}Si NMR (99 MHz, $CDCl_3$) δ** 4.3 (q, *J* = 1.8 Hz). **HRMS (pos. ESI) m/z:** calcd for $C_{19}H_{24}F_6OSiNa$ [M+Na] 433.1393. Found 433.1382. $[\alpha]_D^{27}$ = -80.40 (*c* 0.25, $CHCl_3$).

Determination of *ee* by Chiral GC: Chiraldex β-DM column (30 m column, helium gas carrier at 1.0 mL/min, constant pressure), t_R: 32.5 min (minor enantiomer), 35.6 min (major enantiomer), *ee* = 99%

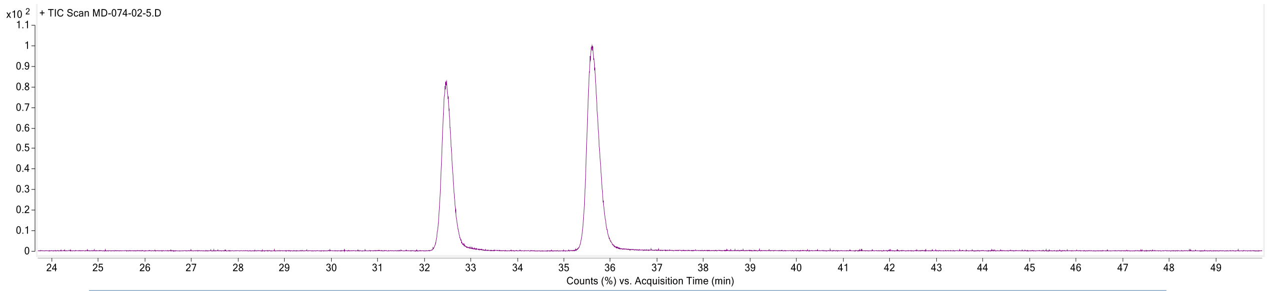

Peaks: + TIC Scan - MD-074-02-5.D

Area	Height	Peak	RT	Area %	Area Sum %	Type	Saturated	Width	FWHM	SNR	RI
2970136.18	168158.5	2	35.606	100	57.72	M		0.829	0.274		
2175443.81	139874.82	1	32.481	73.24	42.28	M		1.235	0.234		

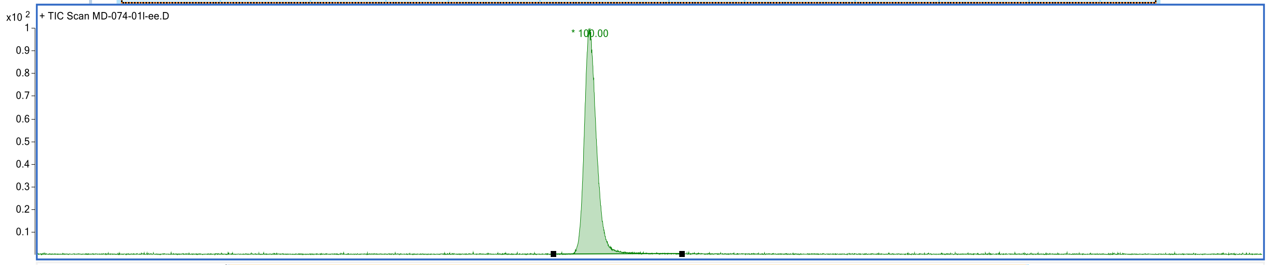

Peaks: + TIC Scan - MD-074-01l-ee.D

Area	Height	Peak	RT	Area %	Area Sum %	Type	Saturated	Width	FWHM	SNR	RI
2074189.25	116025.2	1	35.559	100	100	M		2.77	0.269		

(2*S*,4*S*)-3-(tert-butyldimethylsilyl)-6,6,6-trifluoro-2-(4-fluorophenyl)hexa-3,4-dien-2-ol (5j)

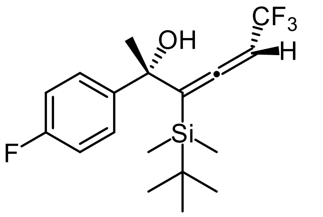

This compound was obtained according to general procedure A. Product **5j** was isolated in 56% yield (20.0 mg, 0.055 mmol) as colourless oil by silica gel chromatography using pentane/DCM 2:1 as eluent, dry loading and the R_f = 0.25 with PMA stain.

^{1}H NMR (500 MHz, CDCl$_3$) δ 7.43-7.39 (m, 2H), 7.03-6.98 (m, 2H), 5.33 (q, *J* = 6.0 Hz, 1H), 1.98 (s, 1H), 1.72 (s, 3H), 0.89 (s, 9H), -0.01 (s, 3H), -0.06 (s, 3H). **^{13}C NMR (126 MHz, CDCl$_3$)** δ 204.6 (q, *J* = 5.7 Hz), 162.0 (d, *J* = 245.7 Hz), 142.5 (d, *J* = 3.3 Hz), 126.9 (d, *J* = 8 Hz), 124.0 (q, *J* = 270.0), 115.0 (d, *J* = 21.4 Hz), 112.7, 83.1 (q, *J* = 38.9 Hz), 76.3 (d, *J* = 2.1 Hz), 33.4, 27.1, 18.1, -3.9, -4.1. **^{19}F NMR (377 MHz, CDCl$_3$) δ** -58.7 (d, *J* = 5.9 Hz), -115.9 (m). **^{29}Si (99 MHz, CDCl$_3$) δ** 4.0 (q, *J* = 1.7 Hz). **HRMS (pos. ESI) m/z:** calcd for $C_{18}H_{24}F_4OSiNa$ [M+Na] 383.1425. Found 383.1412. $[\alpha]_D^{27}$ = – 68.40 (*c* 0.25, $CHCl_3$).

Determination of *ee* by Chiral GC: Chiraldex β-DM column (30 m column, helium gas carrier at 1.0 mL/min, constant pressure), t_R: 32.5 min (minor enantiomer), 33.2 min (major enantiomer), *ee* = 99%

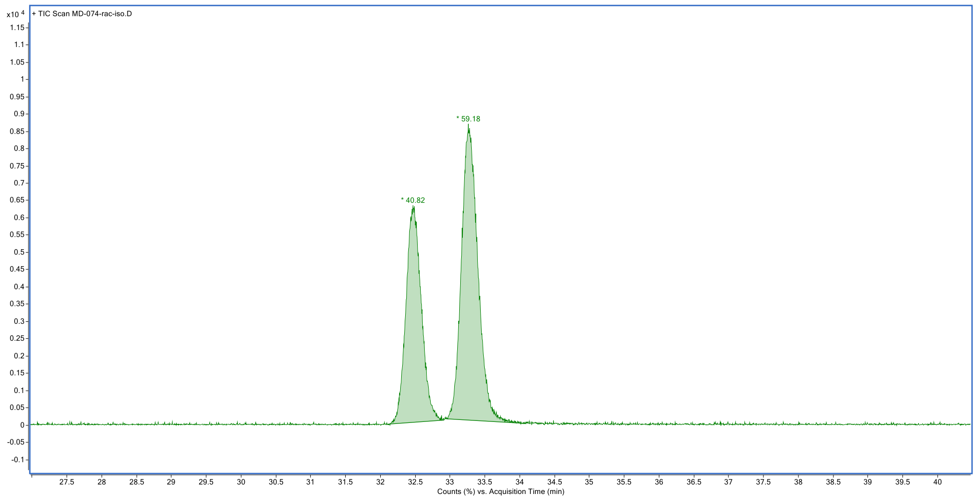

Area	Height	Peak	RT	Area %	Area Sum %	Type	Saturated	Width	FWHM	SNR	RI
687416.81	44882.42	1	32.468	68.98	40.82	M		0.812	0.239		
996562.57	61248.12	2	33.263	100	59.18	M		1.142	0.248		

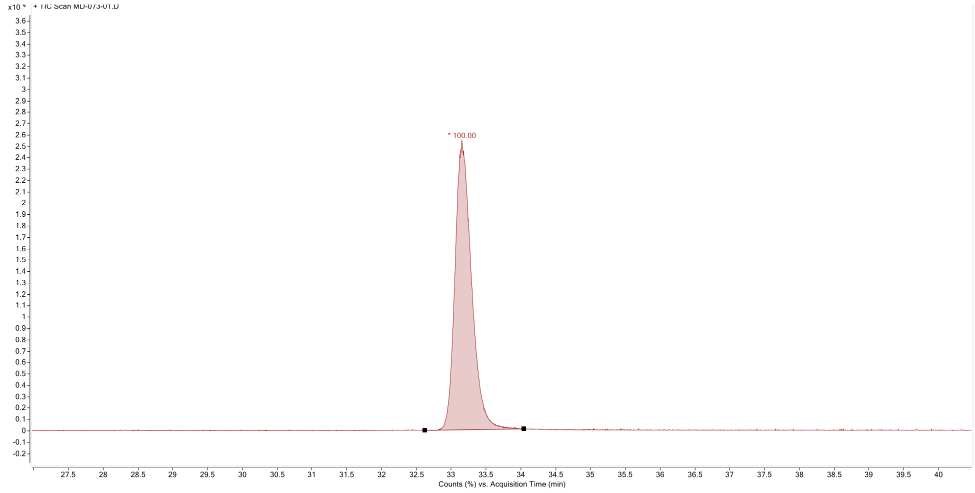

Area	Height	Peak	RT	Area %	Area Sum %	Type	Saturated	Width	FWHM	SNR	RI
3641006.83	221661.13	1	33.153	100	100	M		1.421	0.248		

(2*S*,4*S*)-3-(tert-butyldimethylsilyl)-6,6,6-trifluoro-2-(4-nitrophenyl)hexa-3,4-dien-2-ol (5k)

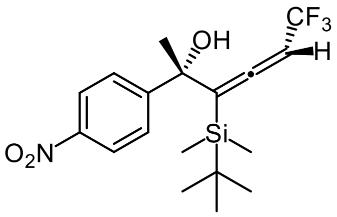

This compound was obtained according to general procedure A. Product **5k** was isolated in 61% yield (23.5 mg, 0.061 mmol) as white solid by silica gel chromatography using pentane/DCM 2:1 as eluent and R_f = 0.3. Mp = 69.5°C.

^{1}H NMR (500 MHz, $CDCl_3$) δ 8.20 – 8.18 (m, 2H), 7.64 – 7.62 (m, 2H), 5.40 (q, J = 5.9 Hz, 1H), 2.15 (s, 1H), 1.75 (s, 3H), 0.89 (s, 9H), -0.00 (s, 3H), -0.07 (s, 3H). **^{13}C NMR (126 MHz, $CDCl_3$) δ** 204.8 (q, J = 5.6 Hz), 153.9, 147.1, 126.2, 123.8 (q, J = 270.1 Hz), 123.6, 112.1, 83.5 (q, J = 39.0 Hz), 76.5 (q, J = 2.1 Hz), 33.4, 27.0, 18.0, -3.9, -4.1. **^{19}F NMR (377 MHz, $CDCl_3$) δ** -58.7 (d, J = 6.1 Hz). **^{29}Si NMR (99 MHz, $CDCl_3$) δ** 4.5 (q, J = 1.5 Hz). **HRMS (pos. ESI) m/z:** calcd for $C_{18}H_{24}F_3NO_3SiNa$ [M+Na] 410.1351. Found 410.1370. $[\alpha]_D^{27}$ = -116.40 (c 0.25, $CHCl_3$).

Determination of *ee* by Chiral SFC: Daicel CHIRALCEL IA-3, 25°c, 0.46 cm ϕ, 10 cm column, 5% MeOH in CO_2, flow rate: 1.2 mL/min; t_R: 1.5 min (major enantiomer), 2.5 min (minor enantiomer), *ee* = 99%.

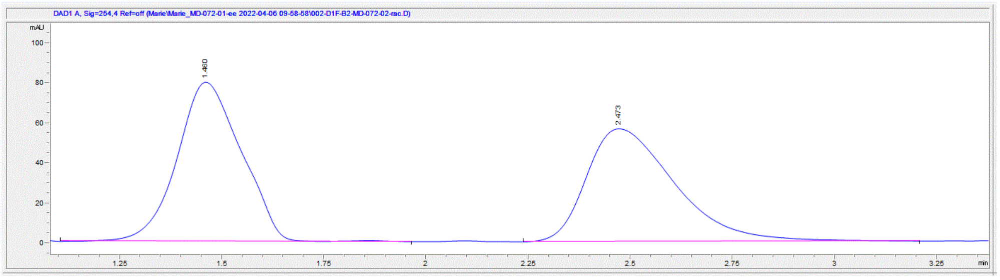

Signal 1: DAD1 A, Sig=254,4 Ref=off

Peak #	RetTime [min]	Type	Width [min]	Area [mAU*s]	Height [mAU]	Area %
1	1.460	BV R	0.1622	875.80573	79.71476	50.8224
2	2.473	BB	0.2352	847.46088	56.43490	49.1776
Totals :				1723.26660	136.14966	

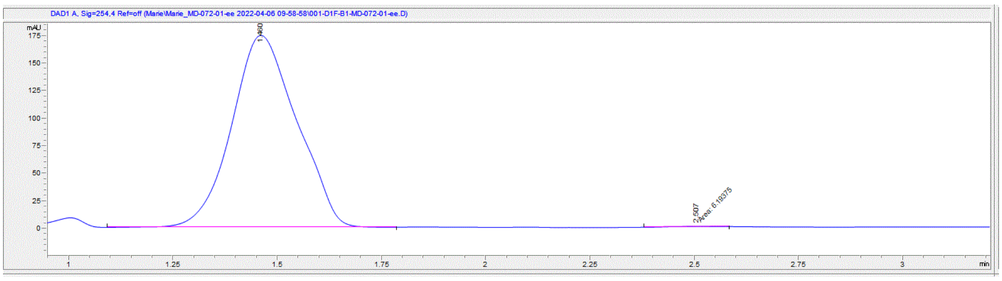

Signal 1: DAD1 A, Sig=254,4 Ref=off

Peak #	RetTime [min]	Type	Width [min]	Area [mAU*s]	Height [mAU]	Area %
1	1.460	BB	0.1608	1901.67444	175.00050	99.6754
2	2.507	MM	0.1170	6.19375	6.54689e-1	0.3246
Totals :				1907.86819	175.65519	

(2*S*,4*R*)-6,6,6-trifluoro-2-(4-(methylsulfonyl)phenyl)-3-(4-(trifluoromethyl)phenyl)hexa-3,4-dien-2-ol (5l)

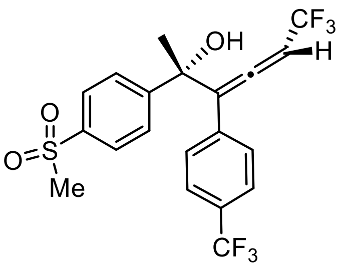

This compound was obtained according to general procedure A except that the reaction temperature is 40 °C. Product **5l** was isolated in 60% yield (27.2 mg, 0.060 mmol) as a white solid by silica gel chromatography using pentane/ Et_2O (2:1 to 1:2) as eluent and R_f = 0.52 (Et_2O/pentane, 2:1). Mp =184-188 °C.

^{1}H NMR (400 MHz, $CDCl_3$) δ 7.92 (d, J = 8.4 Hz, 2H), 7.72 (d, J = 8.5 Hz, 2H), 7.49 (d, J = 8.3 Hz, 2H), 7.33 (d, J = 8.2 Hz, 2H), 6.00 (q, J = 5.6 Hz, 1H), 3.06 (s, 3H), 2.52 (s, 1H), 1.79 (s, 3H). **^{13}C NMR (101 MHz, $CDCl_3$)** δ 205.3 (q, J = 5.6 Hz), 151.2, 139.9, 135.3, 130.7 (q, J = 32.7 Hz), 129.1, 127.9, 126.3, 125.6 (q, J = 3.7 Hz), 123.9 (q, J = 272.3 Hz), 122.4 (q, J = 271.5 Hz), 119.3, 90.5 (q, J = 39.2 Hz), 75.7, 44.6, 32.6. **^{19}F NMR (377 MHz, $CDCl_3$) δ** -59.9 (d, J = 5.6 Hz), -62.9. **HRMS (pos. ESI) m/z:** calcd for $C_{20}H_{16}F_6NaO_3S$ [M+Na] 473.0617. Found 473.0622.
$[\alpha]_D^{26}$ = -96.4 (c 0.25, $CHCl_3$).

Determination of *ee* by Chiral SFC: CHIRALCEL IA-3, 25 °C, 0.3 cm ϕ, 15 cm column, 5% MeOH in CO_2, flow rate 0.8 mL/min; t_R: 4.6 min (minor enantiomer), 6.2 min (major enantiomer), *ee* = 99 %.

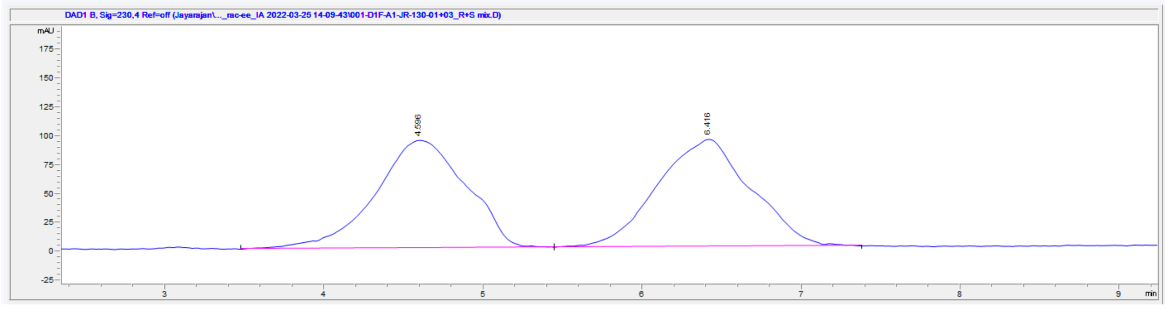

Signal 2: DAD1 B, Sig=230,4 Ref=off

Peak #	RetTime [min]	Type	Width [min]	Area [mAU*s]	Height [mAU]	Area %
1	4.596	VV R	0.5098	3721.56226	93.32466	49.4167
2	6.416	BV R	0.5293	3809.41846	92.87660	50.5833
Totals :				7530.98071	186.20126	

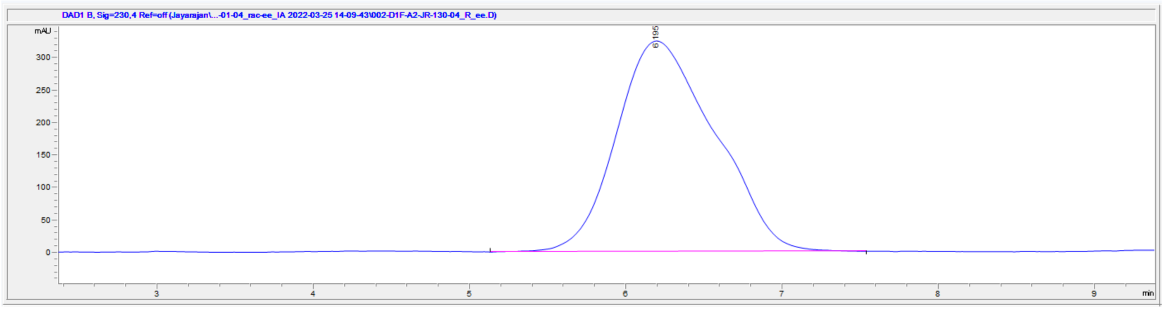

Signal 2: DAD1 B, Sig=230,4 Ref=off

Peak #	RetTime [min]	Type	Width [min]	Area [mAU*s]	Height [mAU]	Area %
1	6.195	BB	0.6370	1.42294e4	324.85867	100.0000
Totals :				1.42294e4	324.85867	

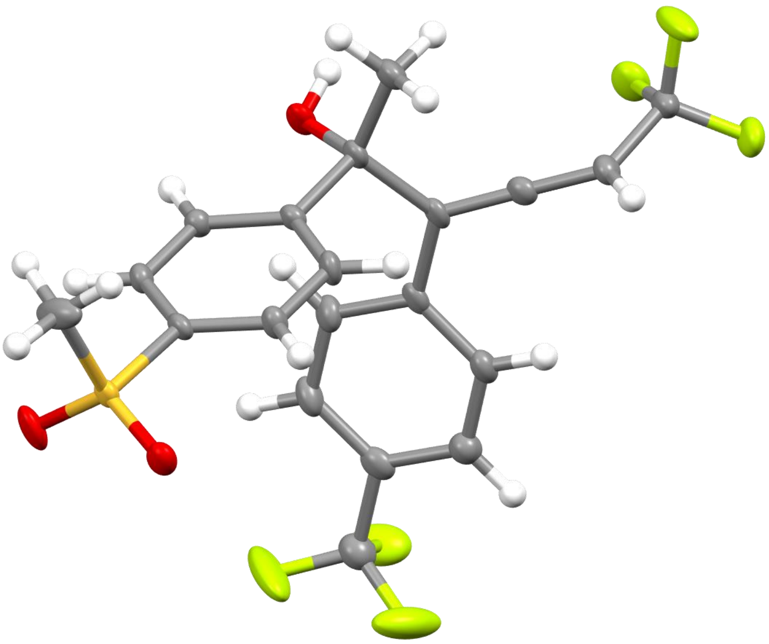

Figure S1: X-ray structure of **5l** (CCDC number: 2174020). See the attached 5l_Xray.cif file.

(*S*)-1-bromo-2-(3-bromophenyl)-3-((*R*)-3,3,3-trifluoroprop-1-en-1-ylidene)heptan-2-ol (5m)

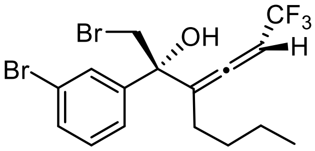

This compound was obtained according to the above general procedure A. Product **5m** was isolated in 68% yield (30 mg, 0.068 mmol) in a diastereomeric ratio of 97:3 (based on ^{19}F NMR) as colourless oil by silica gel chromatography using pentane/DCM 3:1 as eluent and PMA as TLC stain. R_f = 0.33 blue stain.

^{1}H NMR (500 MHz, $CDCl_3$) δ 7.61 (t, *J* = 1.88 Hz, 1H), 7.46 (ddd, *J* = 7.90, 1.96, 1.04 Hz, 1H), 7.34 (ddd, *J* = 7.91, 1.84, 1.06 Hz, 1H), 7.24 (t, *J* = 7.87 Hz, 1H), 5.75 (qt, *J* = 9.26 Hz, 5.82, 3.46 Hz, 1H), 3.88 (d, *J* = 10.72 Hz, 1H), 3.75 (d, *J* = 10.71 Hz, 1H), 2.91 (s, 1H), 2.16–2.10 (m, 1H), 1.85–1.78 (m, 1H), 1.39–1.22 (m, 4H), 0.83 (t, *J* = 7.15, 3H).
^{13}C NMR (126 MHz, $CDCl_3$) δ 201.7 (q, *J* = 5.7 Hz), 143.5, 131.6, 130.2, 128.9, 124.3, 123.0, 122.6 (q, *J* = 271.1 Hz), 116.8, 90.5 (q, *J* = 39.1 Hz), 75.7, 43.1, 29.4, 27.2, 22.2, 13.9.
^{19}F NMR (377 MHz, $CDCl_3$) δ -60.11 (d, *J* = 5.6 Hz)
HRMS (pos. ESI) m/z: calcd for $C_{16}H_{17}Br_2F_3NaO$ [M+Na] 464.9471, 462.9490, 466.9450. Found. 464.9475, 462.9492, 466.9452.
$[\alpha]_D^{27}$ = -96.8 (*c* 0.25, $CHCl_3$).

Determination of *ee* by Chiral SFC: Daicel CHIRALCEL IB, 25°c, 0.46 cm ϕ, 25 cm column, 1% MeOH in CO_2, flow rate: 1.0 mL/min; t_R: min (major enantiomer), min (minor enantiomer), *ee* (major enantiomer) = 95%

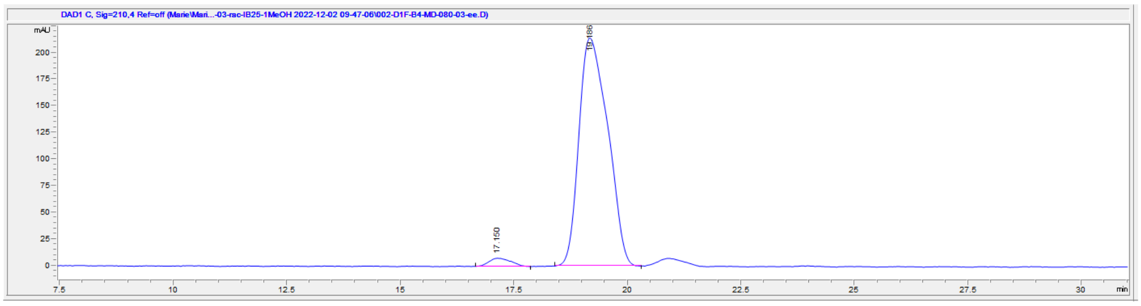

Signal 3: DAD1 C, Sig=210,4 Ref=off

Peak #	RetTime [min]	Type	Width [min]	Area [mAU*s]	Height [mAU]	Area %
1	17.150	BB	0.3974	260.10403	7.75322	2.6680
2	19.186	BB	0.6515	9489.02246	214.74107	97.3320
Totals :				9749.12650	222.49430	

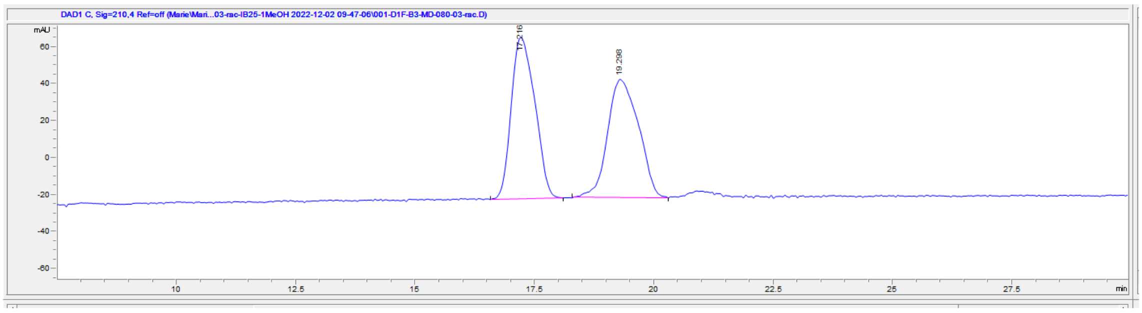

Signal 3: DAD1 C, Sig=210,4 Ref=off

Peak #	RetTime [min]	Type	Width [min]	Area [mAU*s]	Height [mAU]	Area %
1	17.216	BB	0.4943	3012.13818	87.34727	50.8546
2	19.298	BB	0.5494	2910.90186	64.15159	49.1454
Totals :				5923.04004	151.49886	

(2*S*,4*R*)-1-bromo-2-(3-bromophenyl)-6,6,6-trifluoro-3-(4-(trifluoromethyl)phenyl)hexa-3,4-dien-2-ol (5n)

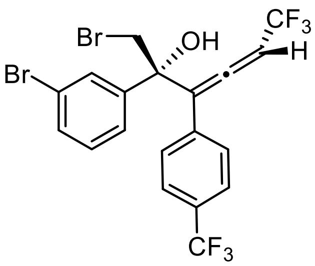

This compound was obtained according to general procedure A except that the reaction temperature is 40 °C. Product **5n** was isolated in 61% yield (32.2 mg, 0.061 mmol) as a colourless oil by silica gel chromatography using pentane/DCM (3:1 to 2:1) as eluent and R_f = 0.5 (pentane/DCM, 2:1).

^{1}H NMR (400 MHz, $CDCl_3$) δ 7.72 (s, 1H), 7.52 (d, *J* = 8.1 Hz, 3H), 7.45 (d, *J* = 7.9 Hz, 1H), 7.38 – 7.26 (m, 3H), 6.04 (q, *J* = 5.8 Hz, 1H), 3.94 (d, *J* = 10.8 Hz, 1H), 3.76 (d, *J* = 10.8 Hz, 1H), 3.13 (s, 1H). **^{13}C NMR (101 MHz, $CDCl_3$) δ** 204.8 (q, *J* = 5.6 Hz), 142.9, 135.3, 132.1, 130.8 (q, *J* = 32.7 Hz), 130.6, 129.3, 129.0, 125.5 (q, *J* = 3.8 Hz), 124.5, 123.9 (q, *J* = 272.2 Hz), 123.3, 122.3 (q, *J* = 271.9 Hz), 116.4, 90.9 (q, *J* = 39.5 Hz), 76.0, 43.5. **^{19}F NMR (377 MHz, $CDCl_3$) δ** -59.6 (d, *J* = 5.9 Hz), -62.9. **HRMS (pos. ESI) m/z:** calcd for $C_{19}H_{11}Br_2F_6O$ [M-H] 528.9056. Found 528.9069, also found 526.9086, and 530.9054. $[\alpha]_D^{28}$ = -92.8 (*c* 0.25, $CHCl_3$).

Determination of *ee* by Chiral SFC: CHIRALCEL OJ-3, 25 °C, 0.3 cm *ϕ*, 15 cm column, 2% MeOH in CO_2, flow rate 1.2 mL/min; t_R: 4.4 min (major enantiomer), 5.6 min (minor enantiomer), *ee* = 99 %.

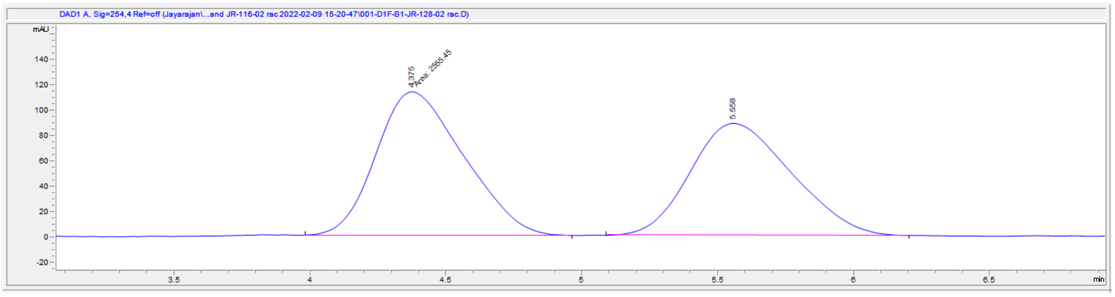

Signal 1: DAD1 A, Sig=254,4 Ref=off

Peak #	RetTime [min]	Type	Width [min]	Area [mAU*s]	Height [mAU]	Area %
1	4.375	FM	0.3749	2555.45239	113.60120	52.7547
2	5.558	BB	0.3861	2288.57837	88.37213	47.2453
Totals :				4844.03076	201.97333	

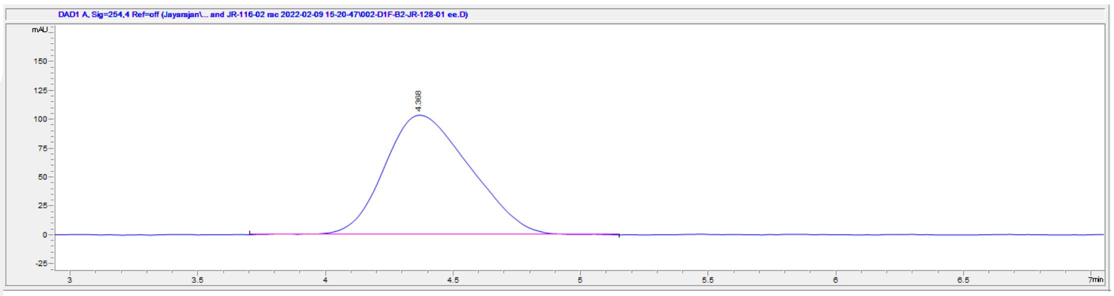

Signal 1: DAD1 A, Sig=254,4 Ref=off

Peak #	RetTime [min]	Type	Width [min]	Area [mAU*s]	Height [mAU]	Area %
1	4.368	VV R	0.3619	2431.38037	103.54720	100.0000
Totals :				2431.38037	103.54720	

(2*R*,4*R*)-6,6,6-trifluoro-2-(thiophen-3-yl)-3-(4-(trifluoromethyl)phenyl)hexa-3,4-dien-2-ol (5o)

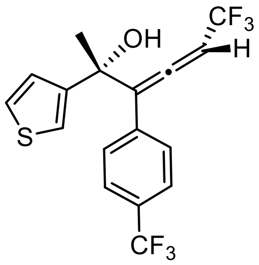

This compound was obtained according to general procedure A except that the reaction temperature is 40 °C. Product **5o** was isolated in 57% yield (21.5 mg, 0.057 mmol) as a yellowish oil by silica gel chromatography using pentane/Et_2O (5:1) and second chromatography with pentane/EA (8:1) as eluent and R_f = 0.57 (pentane/EA, 8:1).

^{1}H NMR (400 MHz, $CDCl_3$) δ 7.52 (d, *J* = 8.3 Hz, 2H), 7.34 (dd, *J* = 5.0, 3.0 Hz, 1H), 7.32 – 7.26 (m, 3H), 7.08 (dd, *J* = 5.0, 1.3 Hz, 1H), 5.87 (q, *J* = 5.7 Hz, 1H), 2.23 (s, 1H), 1.80 (s, 3H). **^{13}C NMR (101 MHz, $CDCl_3$) δ** 204.4 (q, *J* = 5.8 Hz), 146.8, 136.3, 130.5 (q, *J* = 32.7 Hz), 129.2, 126.9, 125.8, 125.5 (q, *J* = 3.7 Hz), 124.0 (q, *J* = 272.1 Hz), 122.6 (q, *J* = 271.1 Hz), 121.4, 120.5, 89.7 (q, *J* = 39.1 Hz), 74.2, 30.6. **^{19}F NMR (377 MHz, $CDCl_3$) δ** -60.0 (d, *J* = 5.7 Hz), -62.8. **HRMS (pos. ESI) m/z:** calcd for $C_{17}H_{12}F_6NaOS$ [M+Na] 401.0405. Found 401.0415. $[\alpha]_D^{26}$ = -54.4 (*c* 0.25, $CHCl_3$).

Determination of *ee* by Chiral SFC: CHIRALCEL IA-3, 25 °C, 0.3 cm ϕ, 15 cm column, 2% MeOH in CO_2, flow rate 1.2 mL/min; t_R: 3.0 min (minor enantiomer), 3.8 min (major enantiomer), *ee* = 99 %.

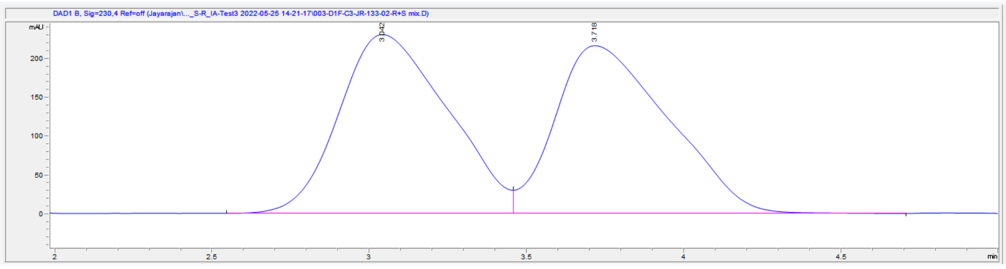

Signal 2: DAD1 B, Sig=230,4 Ref=off

Peak #	RetTime [min]	Type	Width [min]	Area [mAU*s]	Height [mAU]	Area %
1	3.042	BV	0.3561	5552.57031	231.28452	50.6105
2	3.718	VB	0.3714	5418.60352	216.96965	49.3895
Totals :				1.09712e4	448.25417	

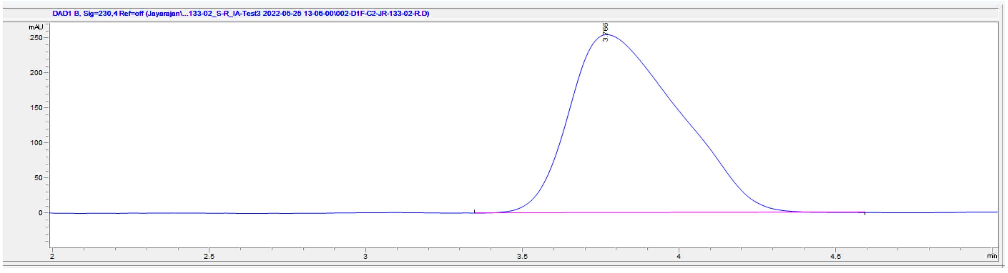

Signal 2: DAD1 B, Sig=230,4 Ref=off

Peak #	RetTime [min]	Type	Width [min]	Area [mAU*s]	Height [mAU]	Area %
1	3.766	BV R	0.3711	6202.39600	255.56898	100.0000
Totals :				6202.39600	255.56898	

Ethyl (*R*)-2-hydroxy-2-methyl-3-((*R*)-3,3,3-trifluoroprop-1-en-1-ylidene)heptanoate (5p)

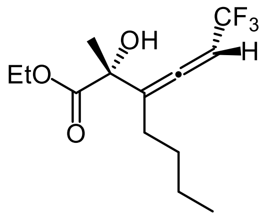

This compound was obtained according to general procedure A. Product **5p** was isolated in 64% yield (18.0 mg, 0.064 mmol) with diastereomeric ratio of 91:9 (based on ^{19}F NMR) as volatile colorless oil by silica gel chromatography using pentane/DCM 1:1 as eluent and R_f = 0.53 with permanganate stain.

^{1}H NMR (400 MHz, CDCl$_3$) δ 5.65-5.59 (qd, *J* = 5.8, 3.0), 4.31-4.17 (m, 2H) 3.52 (s, 1H), 2.24-2.15 (m, 1H), 2.04-1.95 (m, 1H), 1.54 (s, 3H), 1.43-1.31 (m, 6H), 1.30 (t, *J* = 7.1 Hz, 3H), 0.89 (t, *J* = 7.0 Hz, 3H). **^{13}C NMR (101 MHz, CDCl$_3$) δ** 202.5 (q, *J* = 5.9 Hz), 175.0, 122.8 (q, *J* = 270.6 Hz), 116.6, 89.9 (q, *J* = 38.9 Hz), 74.7 (d, *J* = 1.4 Hz), 62.8, 29.5, 26.5, 24.3, 22.3, 14.1, 13.9. **^{19}F NMR (377 MHz, CDCl$_3$)** δ -60.5 (d, *J* = 6.0 Hz). **HRMS (pos. ESI) m/z:** calcd for $C_{13}H_{19}F_3O_3Na$ [M+Na] 303.1179. Found 303.1169. $[\alpha]_D^{27}$ = -55.20 (*c* 0.25, CHCl$_3$).

Determination of *ee* by Chiral SFC: Daicel CHIRALCEL IC-3, 25°c, 0.46 cm ϕ, 10 cm column, 15% MeOH in CO_2, flow rate: 0.8 mL/min; *t*R: 9.1 min (minor enantiomer), 10.4 min (major enantiomer), *ee* = 99%.

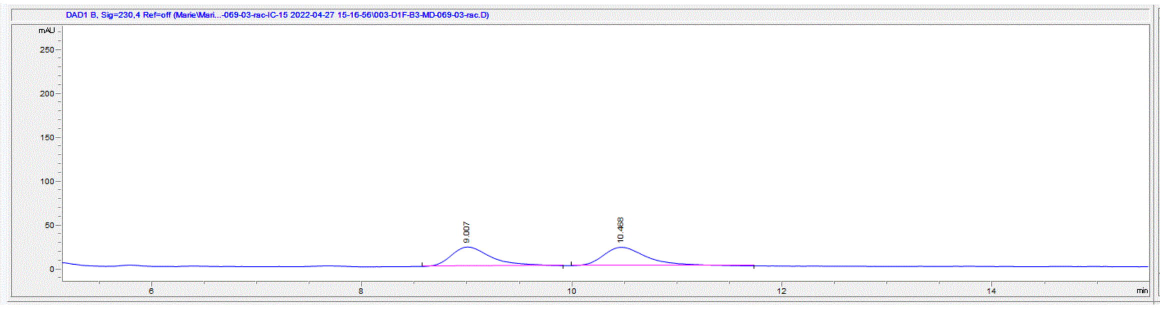

Signal 2: DAD1 B, Sig=230,4 Ref=off

Peak #	RetTime [min]	Type	Width [min]	Area [mAU*s]	Height [mAU]	Area %
1	9.007	BB	0.3599	581.20984	21.87920	48.4826
2	10.468	BB	0.3781	617.59003	20.93719	51.5174
Totals :				1198.79987	42.81639	

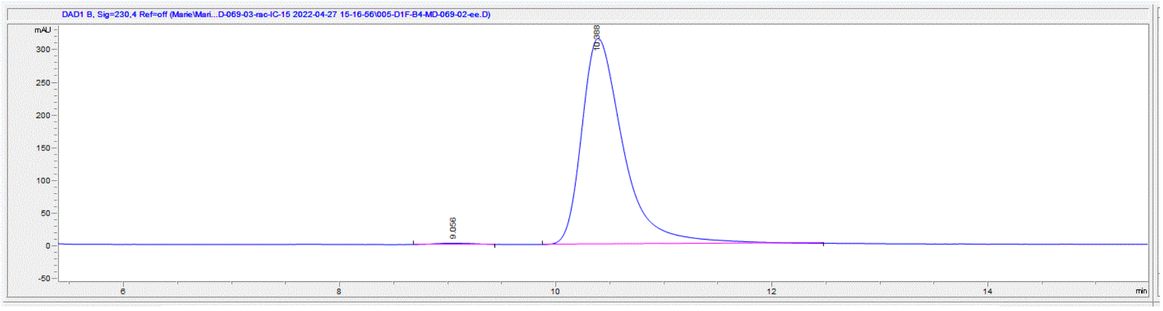

Signal 2: DAD1 B, Sig=230,4 Ref=off

Peak #	RetTime [min]	Type	Width [min]	Area [mAU*s]	Height [mAU]	Area %
1	9.056	BB	0.2837	46.26491	1.94389	0.5448
2	10.388	BB	0.4040	8445.84180	315.83820	99.4552
Totals :				8492.10671	317.78208	

Ethyl (2*R*,4*R*)-6,6,6-trifluoro-2-hydroxy-2-methyl-3-(4-(trifluoromethyl)phenyl)hexa-3,4-dienoate (5q)

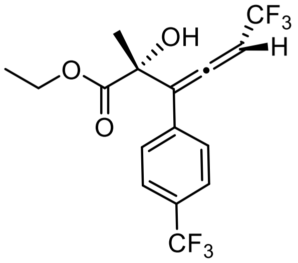

This compound was obtained according to general procedure A except that the reaction temperature is 40 °C. Product **5q** was isolated in 62% yield (22.7 mg, 0.062 mmol) in a diastereomeric ratio of 96:4 (based on ^{19}F NMR) as a colourless oil by silica gel chromatography using pentane/DCM (3:1 to 2:1) as eluent. R_f = 0.22 (pentane/DCM, 2:1).

^{1}H NMR (400 MHz, CDCl$_3$) δ 7.61 (d, *J* = 8.3 Hz, 2H), 7.54 (d, *J* = 8.2 Hz, 2H), 5.88 (q, *J* = 5.7 Hz, 1H), 4.35 – 4.14 (m, 2H), 3.69 (s, 1H), 1.63 (s, 3H), 1.24 (t, *J* = 7.1 Hz, 3H). **^{13}C NMR (101 MHz, CDCl$_3$) δ** 205.1 (q, *J* = 5.7 Hz), 174.8, 135.8, 130.9 (q, *J* = 32.7 Hz), 129.0, 125.6 (q, *J* = 3.7 Hz), 124.0 (q, *J* = 272.2 Hz), 122.3 (q, *J* = 271.4 Hz), 116.1, 90.0 (q, *J* = 39.3 Hz), 75.2, 63.2, 25.4, 14.0. **^{19}F NMR (377 MHz, CDCl$_3$) δ** -60.2 (d, *J* = 5.8 Hz), -62.8. **HRMS (pos. ESI) m/z:** calcd for $C_{16}H_{14}F_6NaO_3$ [M+Na] 391.0739. Found 391.0744.
$[\alpha]_D^{29}$ = -58.4 (*c* 0.25, CHCl$_3$).

Determination of *ee* by Chiral HPLC: CHIRACEL® OD-H, hexane/iPrOH 98/2, flow rate: 1 mL/min; t_R: 5.3 min (minor enantiomer), 5.8 min (major enantiomer), *ee* = 99 %.

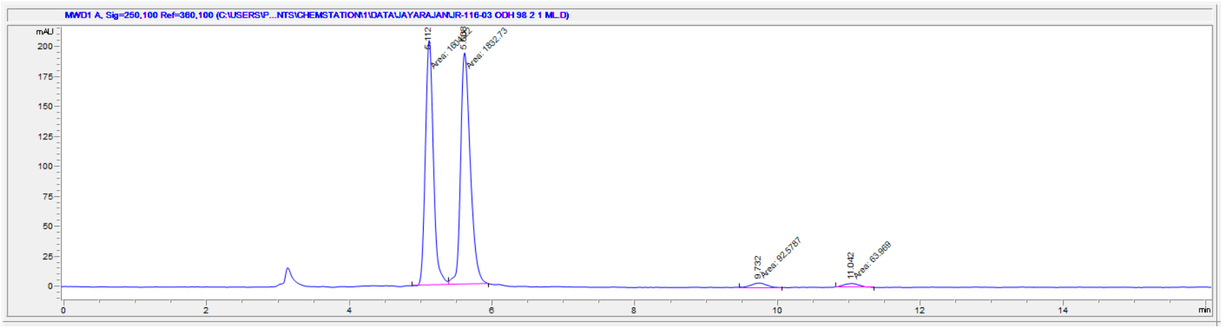

Signal 1: MWD1 A, Sig=250,100 Ref=360,100

Peak #	RetTime [min]	Type	Width [min]	Area [mAU*s]	Height [mAU]	Area %
1	5.112	MF	0.1304	1604.22119	204.97496	44.6424
2	5.608	FM	0.1576	1832.72632	193.85252	51.0012
3	9.732	MM	0.3315	92.57874	4.65421	2.5763
4	11.042	MM	0.2976	63.96896	3.58307	1.7801

Totals : 3593.49521 407.06477

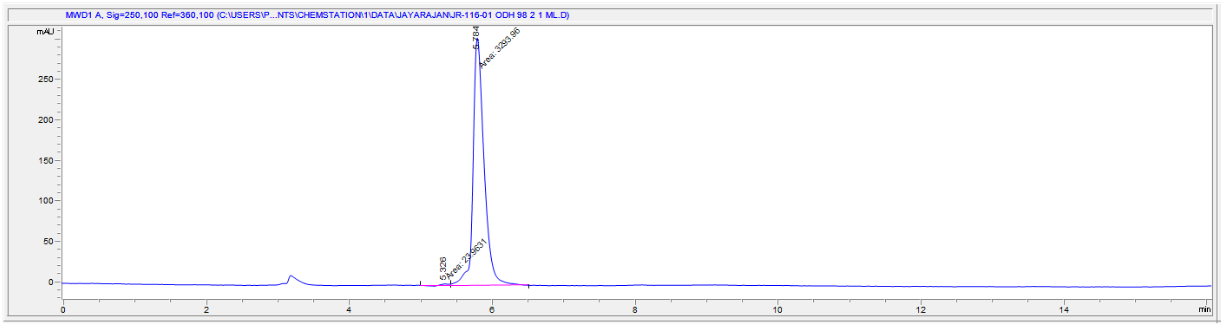

Signal 1: MWD1 A, Sig=250,100 Ref=360,100

Peak #	RetTime [min]	Type	Width [min]	Area [mAU*s]	Height [mAU]	Area %
1	5.326	MF	0.1788	23.96314	2.23377	0.7222
2	5.784	FM	0.1798	3293.96118	305.33252	99.2778

Totals : 3317.92432 307.56629

(3*S*,5*R*)-7,7,7-trifluoro-2,3-dimethyl-4-(4-(trifluoromethyl)phenyl)hepta-4,5-dien-3-ol (5r)

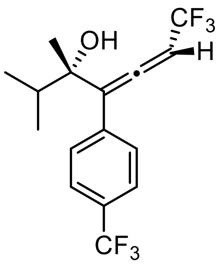

This compound was obtained according to the general procedure A. Product **5r** was isolated in 69% yield (23.2 mg, 0.069 mmol) as a colourless oil by silica gel chromatography using pentane:DCM (2:1) as eluent. R_f = 0.32.

^{1}H NMR (400 MHz, CDCl$_3$) δ 7.60 (s, 4H), 5.72 (q, *J* = 5.7 Hz, 1H), 1.95 (p, *J* = 6.8 Hz, 1H), 1.64 (s, 1H), 1.39 (s, 3H), 1.02 (d, *J* = 6.8 Hz, 3H), 0.90 (d, *J* = 6.8 Hz, 3H).

^{13}C NMR (126 MHz, CDCl$_3$) δ 204.1 (q, *J* = 5.7 Hz), 137.2, 130.5 (q, *J* = 32.7 Hz), 129.7, 125.5 (q, *J* = 3.7 Hz), 124.1 (d, *J* = 272.1 Hz), 122.7 (q, *J* = 271.0 Hz), 120.2, 88.3 (q, *J* = 39.1 Hz), 77.1, 35.2, 23.5, 17.6, 16.6.

^{19}F NMR (377 MHz, CDCl$_3$) δ -60.1 (d, *J* = 5.7 Hz), -62.7.

HRMS (pos. ESI) m/z: calcd for $C_{16}H_{15}F_6O$ [M-H] 337.1033. Found 337.1034.

$[\alpha]_D^{23}$ = 23.6 (*c* 0.25, CHCl$_3$).

Determination of *ee* by Chiral SFC: CHIRALCEL AD-H, 25 °C, 0.3 cm *ϕ*, 25 cm column, 2% MeOH in CO_2, flow rate 1.2 mL/min; t_R: 6.2 min (minor enantiomer), 7.7 min (major enantiomer), *ee* = 99 %.

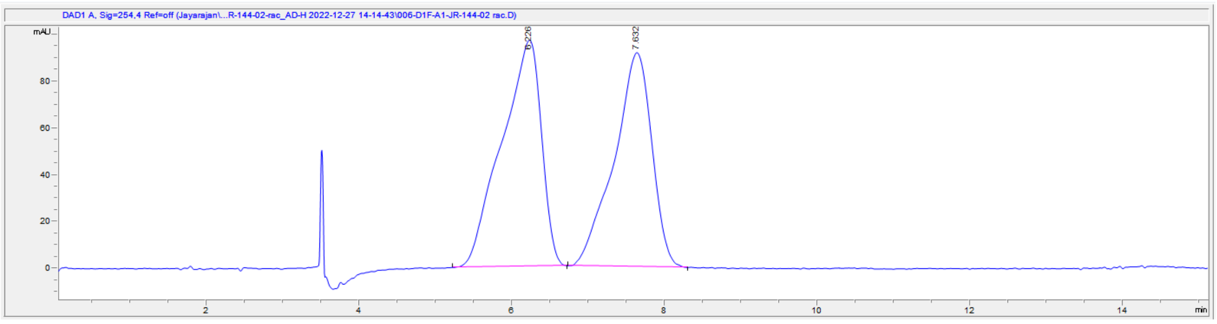

Signal 1: DAD1 A, Sig=254,4 Ref=off

Peak #	RetTime [min]	Type	Width [min]	Area [mAU*s]	Height [mAU]	Area %
1	6.226	BB	0.4925	3492.60767	96.90769	52.5753
2	7.632	BB	0.4948	3150.44434	91.70194	47.4247
Totals :				6643.05200	188.60963	

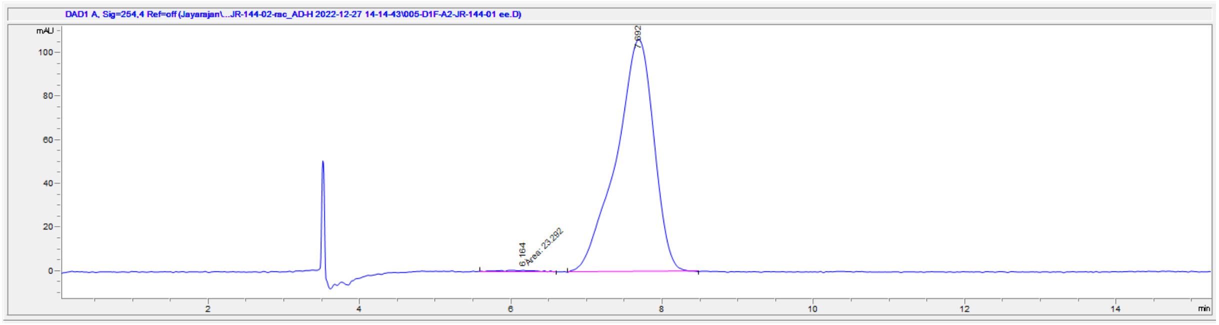

Signal 1: DAD1 A, Sig=254,4 Ref=off

Peak #	RetTime [min]	Type	Width [min]	Area [mAU*s]	Height [mAU]	Area %
1	6.164	MM	0.5709	23.29198	6.80031e-1	0.6240
2	7.692	BB	0.5131	3709.53857	106.82530	99.3760
Totals :				3732.83055	107.50533	

(1*S*,2*S*)-2-methyl-1-((*R*)-4,4,4-trifluoro-1-(4-(trifluoromethyl)phenyl)buta-1,2-dien-1-yl)cyclohexan-1-ol (5s)

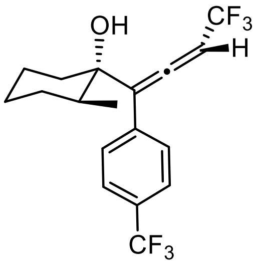

This compound was obtained according to general procedure A except that the reaction is at room temperature. Product **5s** was isolated in 55% yield (20.1 mg, 0.055 mmol) as a colourless oil by silica gel chromatography using pentane/DCM (3:1 to 2:1) as eluent and R_f = 0.38 (pentane/DCM, 2:1).

^{1}H NMR (400 MHz, $CDCl_3$) δ 7.62 (d, *J* = 8.2 Hz, 2H), 7.48 (d, *J* = 8.1 Hz, 2H), 5.70 (q, *J* = 5.7 Hz, 1H), 1.93 – 1.90 (m, 1H), 1.78 – 1.54 (m, 6H), 1.52 (s, 1H), 1.47 – 1.36 (m, 1H), 1.28 – 1.24 (m, 1H), 1.02 (d, *J* = 6.7 Hz, 3H). **^{13}C NMR (126 MHz, $CDCl_3$) δ** 203.2 (q, *J* = 5.7 Hz), 137.3, 130.5 (q, *J* = 32.7 Hz), 129.7, 125.4 (q, *J* = 3.7 Hz), 124.1 (q, *J* = 272.2 Hz), 122.9 (q, *J* = 271.0 Hz), 121.2, 88.6 (q, *J* = 39.0 Hz), 75.7, 38.6, 37.7, 30.3, 25.4, 21.5, 16.0. **^{19}F NMR (377 MHz, $CDCl_3$) δ** -60.0 (d, *J* = 5.8 Hz), -62.7. **HRMS (pos. ESI) m/z:** calcd for $C_{18}H_{18}F_6NaO$ [M+Na] 387.1154. Found 387.1160.
$[\alpha]_D^{28}$ = -5.6 (*c* 0.25, $CHCl_3$).

Determination of *ee* by Chiral SFC: CHIRALCEL IA-3, 25 °C, 0.3 cm ϕ, 15 cm column, 0.1% MeOH in CO_2, flow rate 1.5mL/min; t_R: 1.7 min (major enantiomer), 4.1 min (minor enantiomer), *ee* = 99 %.

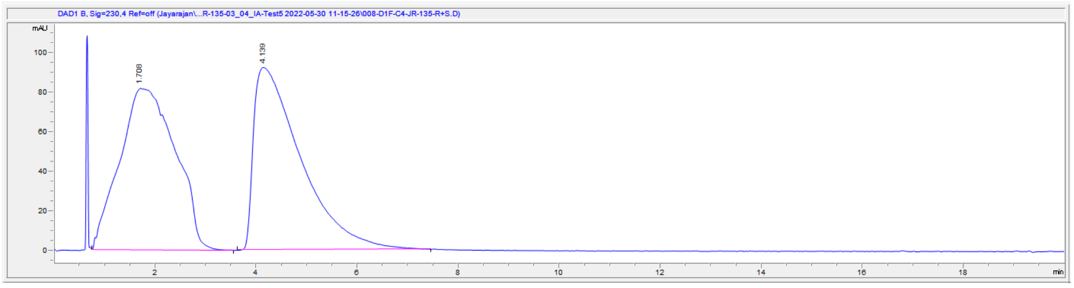

Signal 2: DAD1 B, Sig=230,4 Ref=off

Peak #	RetTime [min]	Type	Width [min]	Area [mAU*s]	Height [mAU]	Area %
1	1.708	VB	0.9583	6312.69971	81.99041	51.0905
2	4.139	BB	0.8671	6043.20850	92.63027	48.9095

Totals : 1.23559e4 174.62068

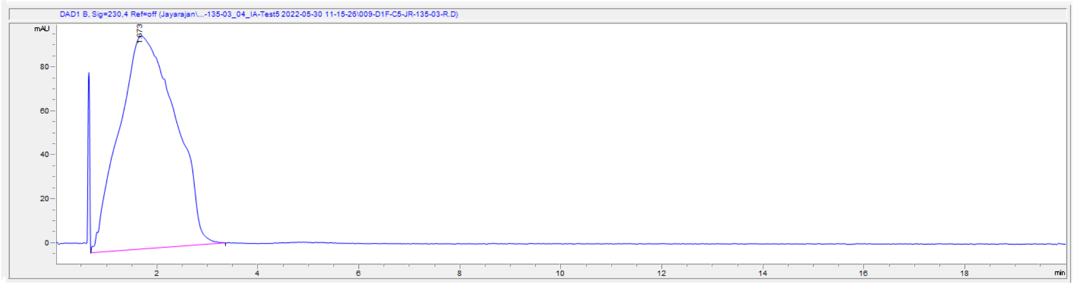

Signal 2: DAD1 B, Sig=230,4 Ref=off

Peak #	RetTime [min]	Type	Width [min]	Area [mAU*s]	Height [mAU]	Area %
1	1.673	BB	0.9824	7502.07324	97.20833	100.0000

Totals : 7502.07324 97.20833

(2*S*,4*R*)-2-(4-nitrophenyl)-3-(4-(trifluoromethyl)phenyl)-5-(trimethylsilyl)penta-3,4-dien-2-ol (7a)

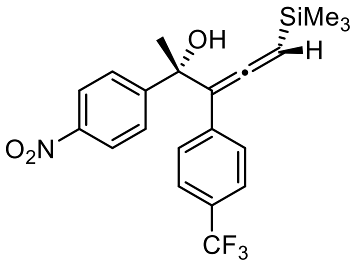

This compound was obtained according to general procedure B. Product **7a** was isolated in 50% yield (20.9 mg, 0.050 mmol) as a pale-yellow oil by silica gel chromatography using pentane/EtOAc (20:1 to 14:1) as eluent and R_f = 0.32 (pentane/EtOAc, 14:1).

^{1}H NMR (400 MHz, $CDCl_3$) δ 8.18 – 8.16 (m, 2H), 7.73 – 7.71 (m, 2H), 7.39 (d, *J* = 8.4 Hz, 2H), 7.32 (d, *J* = 8.4 Hz, 2H), 5.71 (s, 1H), 2.15 (s, 1H), 1.72 (s, 3H), 0.25 (s, 9H). **^{13}C NMR (126 MHz, $CDCl_3$) δ** 208.5, 154.5, 147.1, 138.2, 128.4 (q, *J* = 32.5 Hz), 128.0, 126.3, 125.3 (q, *J* = 3.8 Hz), 123.8, 122.1 (q, *J* = 271.7 Hz), 105.4, 89.2, 75.4, 34.2, -0.57. **^{19}F NMR (377 MHz, $CDCl_3$) δ** -62.6. **^{29}Si NMR (99 MHz, $CDCl_3$) δ** -3.4. **HRMS (pos. ESI) m/z:** calcd for $C_{21}H_{22}F_3NaO_3Si$ [M+Na] 444.1213. Found 444.1233. $[\alpha]_D^{27}$ = -57.6 (*c* 0.25, $CHCl_3$).

Determination of *ee* by Chiral SFC: CHIRALCEL OJ-3, 25 °C, 0.3 cm ϕ, 15 cm column, 2% MeOH in CO_2, flow rate 1.2 mL/min; t_R: 4.2 min (majorenantiomer), 5.8 min (minor enantiomer), *ee* = 98 %.

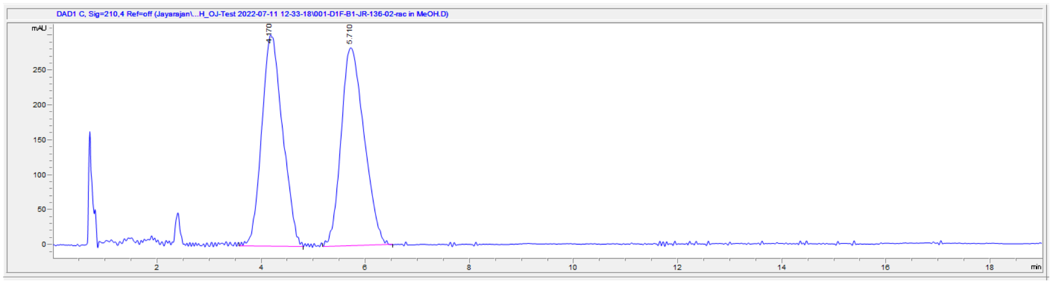

Signal 3: DAD1 C, Sig=210,4 Ref=off

Peak #	RetTime [min]	Type	Width [min]	Area [mAU*s]	Height [mAU]	Area %
1	4.170	VV R	0.3705	8460.46094	303.10941	49.1753
2	5.710	VV R	0.3997	8744.21777	284.01364	50.8247
Totals :				1.72047e4	587.12305	

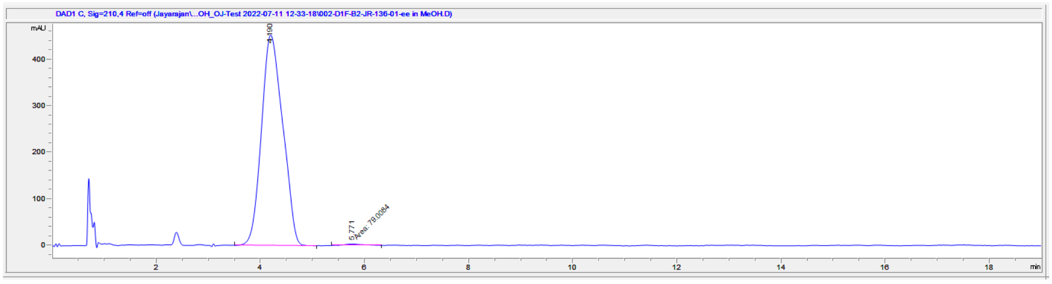

Signal 3: DAD1 C, Sig=210,4 Ref=off

Peak #	RetTime [min]	Type	Width [min]	Area [mAU*s]	Height [mAU]	Area %
1	4.190	BV R	0.4197	1.28614e4	452.41113	99.3894
2	5.771	MM	0.4426	79.00840	2.97538	0.6106
Totals :				1.29404e4	455.38652	

(2*S*,4*R*)-2,3-bis(4-(trifluoromethyl)phenyl)-5-(trimethylsilyl)penta-3,4-dien-2-ol (7b)

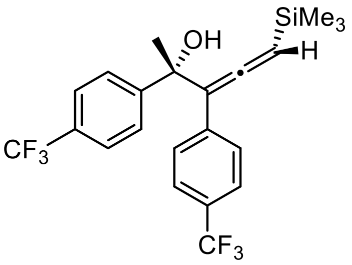

This compound was obtained according to general procedure B. Product **7b** was isolated in 40% yield (17.6 mg, 0.040 mmol) as a colourless oil by silica gel chromatography using pentane/DCM (4:1 to 2:1) as eluent and R_f = 0.31 (pentane/DCM, 4:1). After several attempts for purification small traces of 4-CF_3-acetophenone derivative was still present in the product.

^{1}H NMR (400 MHz, $CDCl_3$) δ 7.67 (d, *J* = 8.2 Hz, 2H), 7.58 (d, *J* = 8.3 Hz, 2H), 7.40 (d, *J* = 8.4 Hz, 2H), 7.33 (d, *J* = 8.4 Hz, 2H), 5.67 (s, 1H), 2.09 (s, 1H), 1.71 (s, 3H), 0.25 (s, 9H). **^{13}C NMR (101 MHz, $CDCl_3$) δ** 208.7, 151.1, 138.5, 129.3 (q, *J* = 32.3 Hz), 128.2 (q, *J* = 32.4 Hz), 128.0, 125.7, 125.5 (q, *J* = 3.7 Hz), 125.2 (q, *J* = 3.7 Hz), 121.6 (q, *J* = 272.0 Hz), 121.6 (q, *J* = 271.8 Hz), 105.7, 88.8, 75.4, 34.2, -0.6. **^{19}F NMR (377 MHz, $CDCl_3$) δ** -62.4, -62.6. **HRMS (pos. ESI) m/z:** calcd for $C_{22}H_{22}F_6NaOSi$ [M+Na] 467.1236. Found 467.1256.
$[\alpha]_D^{29}$ = -21.6 (*c* 0.25, $CHCl_3$).

Determination of *ee* by Chiral HPLC: CHIRACEL® AD-H, hexane/iPrOH 98/2, flow rate: 1 mL/min; t_R: 9.3 min (major enantiomer), 12.6 min (minor enantiomer), *ee* = 98%.

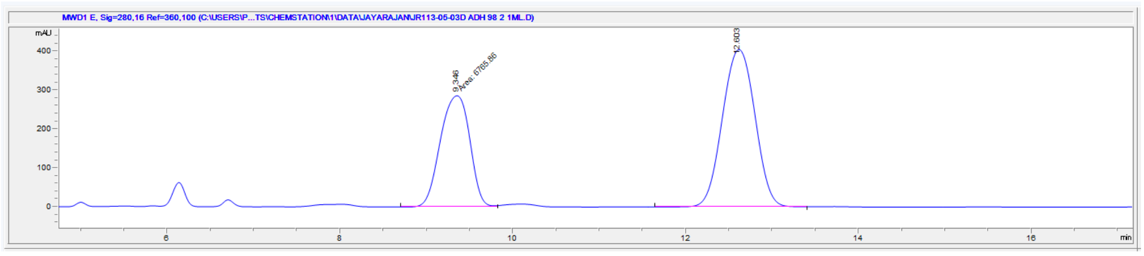

Signal 5: MWD1 E, Sig=280,16 Ref=360,100

Peak #	RetTime [min]	Type	Width [min]	Area [mAU*s]	Height [mAU]	Area %
1	9.346	BV R	0.3879	6947.62207	285.71274	39.6684
2	12.603	BB	0.4209	1.05666e4	404.32108	60.3316
Totals :				1.75143e4	690.03381	

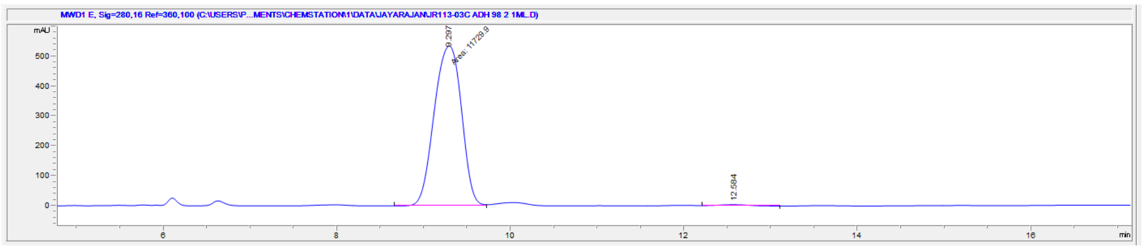

Signal 5: MWD1 E, Sig=280,16 Ref=360,100

Peak #	RetTime [min]	Type	Width [min]	Area [mAU*s]	Height [mAU]	Area %
1	9.297	MF	0.3650	1.17299e4	535.60876	99.1053
2	12.584	VB	0.3795	105.89558	4.18026	0.8947
Totals :				1.18358e4	539.78903	

(2*S*,4*R*)-1-bromo-2-(3-bromophenyl)-3-(4-(trifluoromethyl)phenyl)-5-(trimethylsilyl)penta-3,4-dien-2-ol (7c)

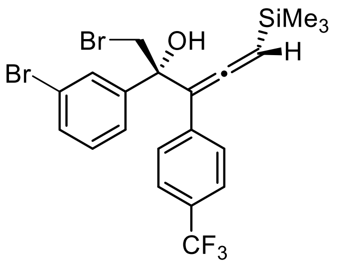

This compound was obtained according to the general procedure B. Product **7c** was isolated in 52% yield (27.8 mg, 0.052 mmol) as a colourless oil by silica gel chromatography using pentane/Et_2O (20:1 to 14:1) as eluent, dry loading. R_f = 0.46 (pentane/ Et_2O, 30:1).

^{1}H NMR (400 MHz, $CDCl_3$) δ 7.78 (s, 1H), 7.53 (d, *J* = 7.8 Hz, 1H), 7.48 – 7.45 (m, 3H), 7.38 (d, *J* = 8.2 Hz, 2H), 7.29 – 7.24 (m, 1H), 5.75 (s, 1H), 4.00 (d, *J* = 10.5 Hz, 1H), 3.73 (d, *J* = 10.5 Hz, 1H), 2.98 (s, 1H), 0.32 (s, 9H). **^{13}C NMR (101 MHz, $CDCl_3$) δ** 208.1, 145.0, 138.2, 131.4, 130.3, 129.8, 129.2, 128.4 (q, *J* =33 Hz), 128.3, 127.1 (q, *J* = 271.8 Hz), 125.1 (q, *J* = 3.7 Hz), 124.7, 123.0, 102.6, 89.8, 74.5, 45.6, -0.5. **^{19}F NMR (377 MHz, $CDCl_3$) δ** -62.6. **^{29}Si NMR (99 MHz, $CDCl_3$) δ** -3.00.

HRMS (pos. ESI) m/z: calcd for $C_{21}H_{21}Br_2F_3NaOSi$ [M+Na] 554.9573. Found 554.9561, also found 556.9537, 558.9512.

$[\alpha]_D^{24}$ = –50.8 (*c* 0.25, $CHCl_3$).

Determination of *ee* by Chiral SFC: CHIRALCEL AD-H, 25 °C, 0.3 cm ϕ, 25 cm column, 2% MeOH in CO_2, flow rate 1.2 mL/min; t_R: 17.3 min (majorenantiomer), 20.3 min (minor enantiomer), *ee* = 95%.

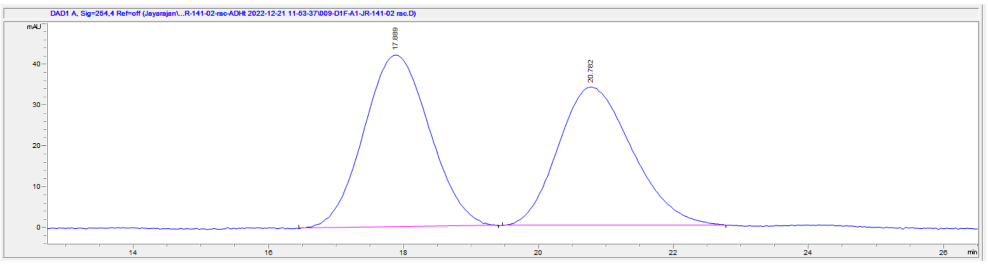

Signal 1: DAD1 A, Sig=254,4 Ref=off

Peak #	RetTime [min]	Type	Width [min]	Area [mAU*s]	Height [mAU]	Area %
1	17.889	BB	0.8149	2895.01440	42.49064	52.5165
2	20.782	BB	0.9089	2617.56909	34.20094	47.4835
Totals :				5512.58350	76.69159	

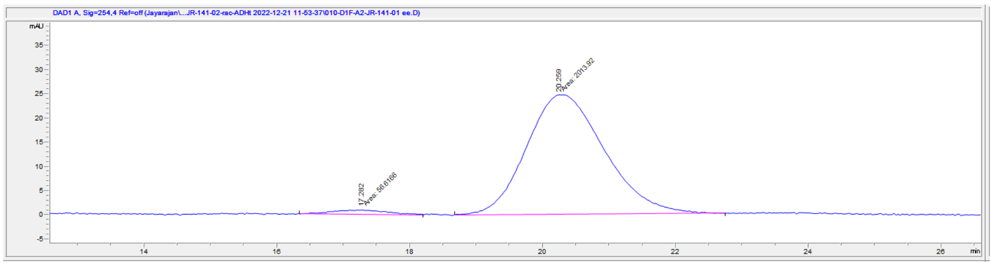

Signal 1: DAD1 A, Sig=254,4 Ref=off

Peak #	RetTime [min]	Type	Width [min]	Area [mAU*s]	Height [mAU]	Area %
1	17.282	MM	0.9860	56.61662	9.57007e-1	2.7344
2	20.259	MM	1.3629	2013.92444	24.62867	97.2656
Totals :				2070.54106	25.58567	

(*S*)-2-(4-nitrophenyl)-3-((*R*)-2-(trimethylsilyl)vinylidene)heptan-2-ol (7d)

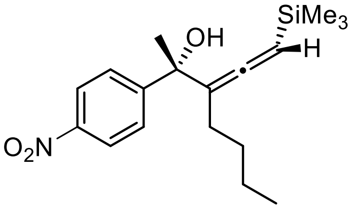

This compound was obtained according to the general procedure B. Product **7d** was isolated in 51% yield (17.0 mg, 0.051 mmol) as a colourless oil by silica gel chromatography using pentane/EtOAc (20:1 to 14:1) as eluent. R_f = 0.56 (pentane/EtOAc, 14:1).

^{1}H NMR (400 MHz, CDCl$_3$) δ 8.17 (d, *J* = 8.4 Hz, 2H), 7.63 (d, *J* = 8.4 Hz, 2H), 5.29 (t, *J* = 4.2 Hz, 1H), 1.86 (s, 1H), 1.84-1.77 (m, 1H) 1.62 (s, 3H), 1.60-1.56 (m, 1H) 1.20-1.32 (m, 4H), 0.81 (t, *J* = 7.0 Hz, 3H), 0.15 (s, 9H).
^{13}C NMR (126 MHz, CDCl$_3$) δ 206.5, 154.7, 146.9, 126.6, 123.4, 104.5, 88.3, 75.5, 31.2, 30.6, 26.4, 22.6, 14.0, -0.6.
^{29}Si NMR (99 MHz, CDCl$_3$) δ -5.16.
HRMS (pos. ESI) m/z: calcd for $C_{18}H_{27}NaNO_3Si$ [M+Na] 356.1652. Found 356.1646.
$[\alpha]_D^{24}$ = −106.4 (*c* 0.25, CHCl$_3$).
Determination of *ee* by Chiral SFC: CHIRALCEL AD-H, 25 °C, 0.3 cm ϕ, 25 cm column, 3% MeOH in CO_2, flow rate 1.2 mL/min; t_R: 21.5 min (major enantiomer), 25.7 min (minor enantiomer), *ee* = 98 %.

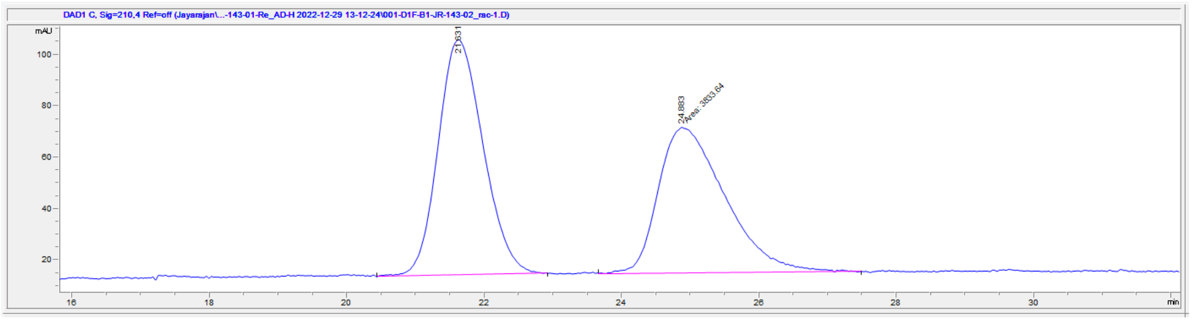

Signal 3: DAD1 C, Sig=210,4 Ref=off

Peak #	RetTime [min]	Type	Width [min]	Area [mAU*s]	Height [mAU]	Area %
1	21.631	VV R	0.6061	4190.02051	91.52997	52.2208
2	24.883	MM	1.1269	3833.64331	56.69692	47.7792

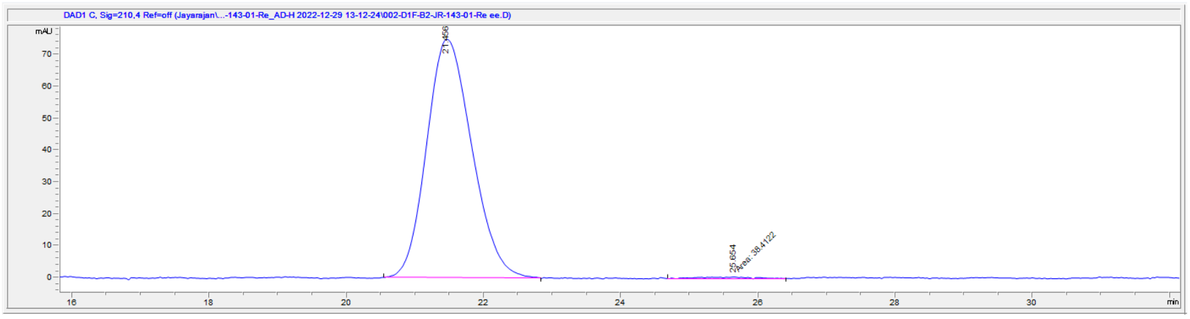

Signal 3: DAD1 C, Sig=210,4 Ref=off

Peak #	RetTime [min]	Type	Width [min]	Area [mAU*s]	Height [mAU]	Area %
1	21.456	BB	0.5644	3436.95117	74.75820	98.8947
2	25.654	MM	1.0072	38.41222	6.35639e-1	1.1053

Totals : 3475.36339 75.39384

Preparation of BDan derivative:

(*S*)-2-(1,1,1-trifluorooct-3-yn-2-yl)-2,3-dihydro-1H-naphtho[1,8-de][1,3,2]diazaborinine (8)

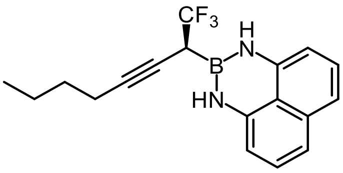

A reaction tube was charge with (R)-iodo-BINOL **4** (0.03 mmol) and was brought into the glovebox. Molecular sieves (20 mg), alkynyl boronic ester **1** (0.1 mmol, 400 µL of 0.25 mol/L stock solution in DCM), and CF_3-diazomethane **2** (0.3 mmol) were added sequentially. The total volume was maintained to 0.8 mL and the reaction mixture was stirred at 30 °C for 18 h. Then, 1,8-diaminonaphtalene (0,15 mmol) was added inside the glovebox and stirred for an additional 3 h. The product **8** was isolated in 69% yield (22.8 mg, 0.069 mmol) as colourless oil by flash silica gel chromatography using pentane/DCM 4:1 as a eluent and R_f = 0.62 with PMA stain. Compound **8** is fairly oxygen sensitive and slowly decomposes even under inert conditions. The minor impurities (^{1}H NMR) in the isolated product is probably due to oxidation under silica gel purification.

^{1}H NMR (500 MHz, $CDCl_3$) δ 7.14-7.11 (m, 2H), 7.08-7.06 (m, 2H), 6.37 (dd, *J* = 7.2 Hz, 1.1 Hz, 2H), 5.98 (s, 2H), 3.04-2.97 (m, 1H), 2.29 (td, *J* = 7.0, 2.5 Hz, 2H), 1.59-1.54 (m, 2H), 1.51-1.45 (m, 2H), 0.96 (t, *J* = 7.3 Hz, 3H). **^{13}C NMR (126 MHz, $CDCl_3$) δ** 140.1, 139.8, 136.4, 129.6, 127.7, 126.3 (q, *J* = 277.4 Hz), 120.1, 118.9, 118.7, 106.8, 106.6, 86.0, 71.0 (d, *J* = 5.4 Hz), 30.9, 22.1, 18.6, 13.7. **^{19}F NMR (377 MHz, $CDCl_3$) δ** -63.1 (d, *J* = 10.6 Hz). **^{11}B NMR (128 MHz, $CDCl_3$) δ** 27.8 ppm. **HRMS (pos. ESI) m/z:** calcd for $C_{18}H_{18}BF_3N_2Na$ [M+Na] 353.1411. Found 353.1418. $[\alpha]_D^{27}$ = +11.60 (*c* 0.25, $CHCl_3$).

Determination of *ee* by Chiral SFC: Daicel CHIRALCEL OJ-3, 25°c, 0.46 cm ϕ, 10 cm column, 2% *i*PrOH in CO_2, flow rate: 1.2 mL/min; t_R: 5.5 min (major enantiomer), 6.0 min (minor enantiomer), *ee* = 85%.

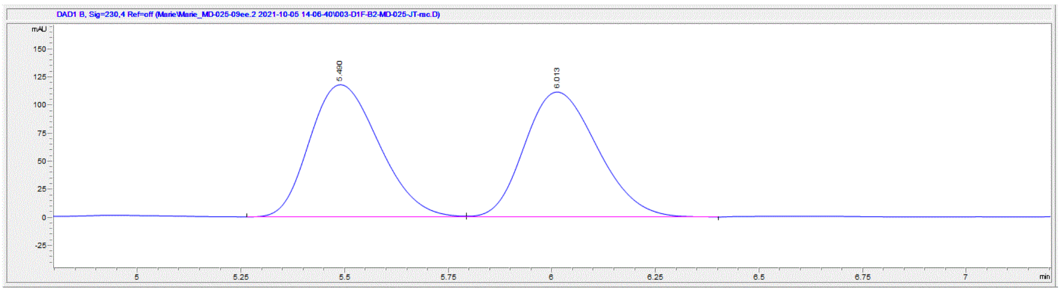

Signal 2: DAD1 B, Sig=230,4 Ref=off

Peak #	RetTime [min]	Type	Width [min]	Area [mAU*s]	Height [mAU]	Area %
1	5.490	BV	0.1859	1368.34167	117.50932	50.1760
2	6.013	VB	0.1947	1358.74402	111.06465	49.8240
Totals :				2727.08569	228.57397	

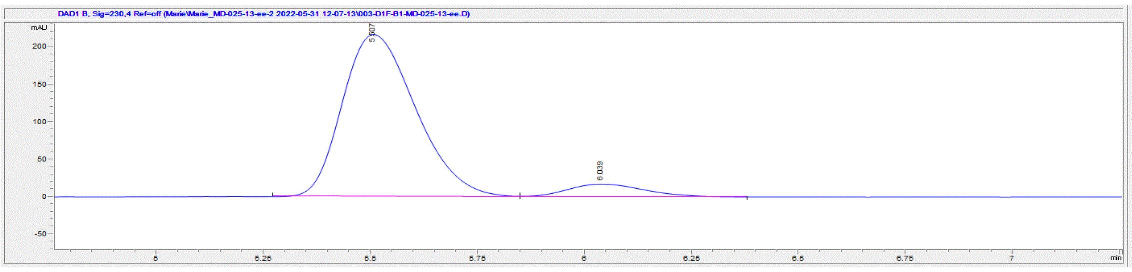

Signal 2: DAD1 B, Sig=230,4 Ref=off

Peak #	RetTime [min]	Type	Width [min]	Area [mAU*s]	Height [mAU]	Area %
1	5.507	BV	0.1868	2579.89722	216.80186	92.3578
2	6.039	VB	0.1946	213.47554	16.99117	7.6422
Totals :				2793.37276	233.79303	

Reaction with aldehyde with two operational steps

(1*S*,3*R*)-1-(4-bromophenyl)-5,5,5-trifluoro-2-(4-(trifluoromethyl)phenyl)penta-2,3-dien-1-ol (10b)

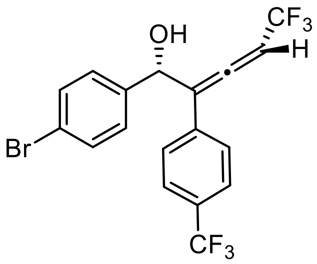

The homologation reaction was done by following above procedure using diethyl ((4-(trifluoromethyl)phenyl)ethynyl)boronate **1** (0.1 mmol, 400 μL of 0.25 mol/L stock solution in DCM). After 18 h, the excess CF_3-diazomethane was evaporated by argon blow until the volume reduced to 0.5 mL and aldehyde was added. The reaction mixture was stirred at 40 °C for 18 h. The crude mixture was purified by silica gel chromatography to provide the product **10b** in 44% yield (19.1 mg, 0.044 mmol) in a diastereomeric ratio of 95:5 (based on ^{19}F NMR) as a pale-yellow oil. Eluent: pentane/DCM (3:1 to 1:1) and R_f = 0.17 (pentane/DCM, 1:1).

^{1}H NMR (400 MHz, $CDCl_3$) δ 7.57 (d, *J* = 8.3 Hz, 2H), 7.53 – 7.45 (m, 2H), 7.44 (d, *J* = 8.2 Hz, 2H), 7.34 – 7.26 (m, 1H), 5.99 (qd, *J* = 5.7, 2.5 Hz, 1H), 5.76 (dd, *J* = 5.1, 2.5 Hz, 1H), 2.30 (d, *J* = 5.0 Hz, 1H). **^{13}C NMR (101 MHz, $CDCl_3$)** δ 205.6 (q, *J* = 5.5 Hz), 139.2, 135.4, 132.2, 130.8 (q, *J* = 32.8 Hz), 128.6, 127.8, 126.0 (q, *J* = 3.7 Hz), 123.9 (q, *J* = 272.2 Hz), 122.9, 122.4 (q, *J* = 271.5 Hz), 116.9, 91.9 (q, *J* = 39.2 Hz), 72.4. **^{19}F NMR (377 MHz, $CDCl_3$)** δ -60.0 (d, *J* = 5.5 Hz), -62.9. **HRMS (pos. ESI) m/z:** calcd for $C_{18}H_{10}BrF_6O$ [M-H] 434.9825. Found 434.9807, also found 436.9794.
$[\alpha]_D^{27}$ = 55.2 (*c* 0.25, $CHCl_3$).

Determination of *ee* by Chiral SFC: CHIRALCEL OJ-3, 25 °C, 0.3 cm ϕ, 15 cm column, 5% MeOH in CO_2, flow rate 0.8 mL/min; t_R: 4.1 min (minor enantiomer), 6.0 min (major enantiomer), *ee* = 78%.

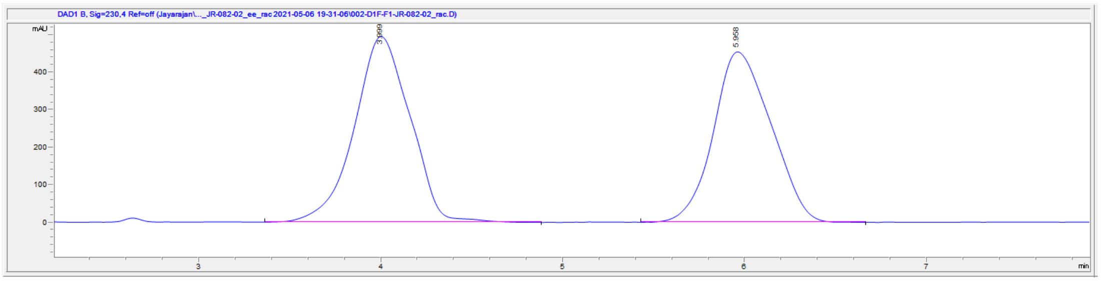

Signal 2: DAD1 B, Sig=230,4 Ref=off

Peak #	RetTime [min]	Type	Width [min]	Area [mAU*s]	Height [mAU]	Area %
1	3.999	BV R	0.3088	1.05980e4	496.90790	51.3858
2	5.958	BV R	0.3287	1.00264e4	455.71005	48.6142
Totals :				2.06244e4	952.61795	

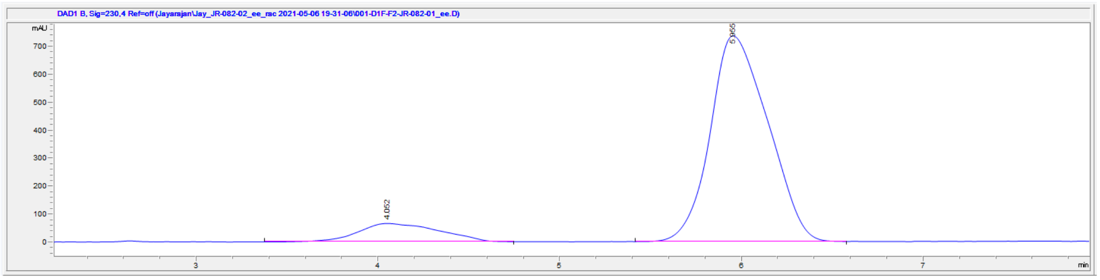

Signal 2: DAD1 B, Sig=230,4 Ref=off

Peak #	RetTime [min]	Type	Width [min]	Area [mAU*s]	Height [mAU]	Area %
1	4.052	BB	0.4207	2056.37622	65.77573	11.2180
2	5.955	BB	0.3656	1.62746e4	737.11066	88.7820
Totals :				1.83310e4	802.88638	

(2*S*,4*R*)-2-(4-bromophenyl)-3-cyclohexyl-6,6,6-trifluorohexa-3,4-dien-2-ol (10c).

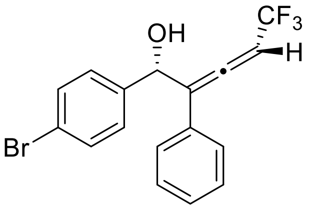

The homologation reaction was done by following above procedure using diethyl ((phenyl)ethynyl)boronate **1** (0.1 mmol, 400 μL of 0.25 mol/L stock solution in DCM). After 18 h, the excess CF_3-diazomethane was evaporated by argon blow until the volume reduced to 0.5 mL and aldehyde was added. The reaction mixture was stirred at 40 °C for 18 h. The crude mixture was purified by silica gel chromatography to provide the product **10c** in 73% yield (26.8 mg, 0.073 mmol) as a colourless oil. Eluent: pentane/EtOAc (10:1) and R_f = 0.21

^{1}H NMR (500 MHz, $CDCl_3$) δ 7.49–7.46 (m, 2H), 7.32–7.29 (m, 7H), 5.94 (qd, *J* = 5.71, 2.57 Hz, 1H), 5.76 (dd, *J* =5.13, 2.58 Hz, 1H), 2.26 (d, *J* = 5.16 Hz, 1H). **^{13}C NMR (126 MHz, $CDCl_3$) δ** 205.1 (q, *J* = 5.8 Hz), 139.6, 132.0, 131.5, 129.1, 128.7, 127.4, 122.6, 122.6 (q, *J* = 271.5 Hz), 118.0 Hz, 91.5 (q, *J* = 39.0 Hz), 72.4. **^{19}F NMR (377 MHz, $CDCl_3$) δ** -60.0 (d, *J* = 5.6 Hz). **HRMS (pos. ESI) m/z:** calcd for $C_{17}H_{12}BrF_3NaO$ [M+Na]390.9916. Found 390.9915, also found 392.9895. $[\alpha]_D^{27}$ = +88.40 (*c* 0.25, $CHCl_3$).

Determination of *ee* by Chiral SFC: Daicel CHIRALCEL IA, 25°c, 0.46 cm ϕ, 10 cm column, 5% MeOH in CO_2, flow rate: 1.2 mL/min; t_R: 7.43 min (major enantiomer), 9.12 min (minor enantiomer), *ee* (major enantiomer) = 83%

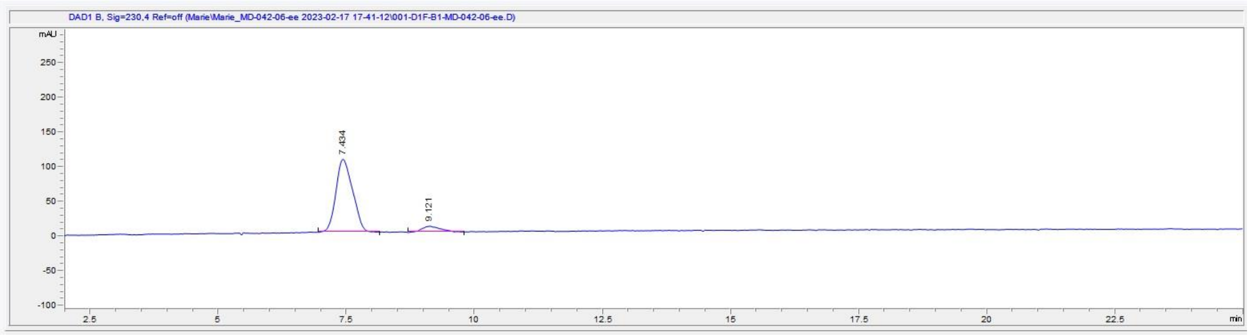

Signal 2: DAD1 B, Sig=230,4 Ref=off

Peak #	RetTime [min]	Type	Width [min]	Area [mAU*s]	Height [mAU]	Area %
1	7.434	BV R	0.3307	2416.74902	105.70741	91.3473
2	9.121	BV R	0.3178	228.92323	8.56641	8.6527

Totals : 2645.67226 114.27382

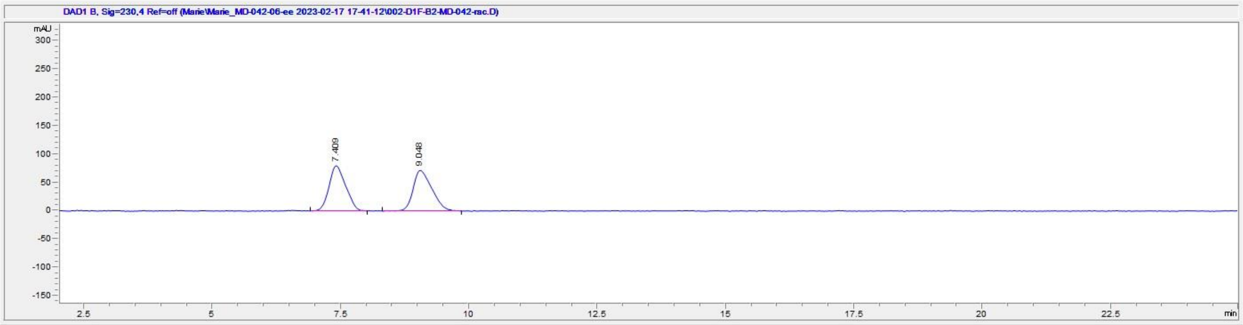

Signal 2: DAD1 B, Sig=230,4 Ref=off

Peak #	RetTime [min]	Type	Width [min]	Area [mAU*s]	Height [mAU]	Area %
1	7.409	BV R	0.3214	1803.59106	79.87501	49.3626
2	9.048	VV R	0.3521	1850.16882	71.91758	50.6374

Totals : 3653.75989 151.79259

Control experiment – for tracing intermediate 12

The reaction of alkenyl diethyl-boronate **1a** (0.1 mmol, 18 mg) with catalyst **4** (0.1 mmol, 54 mg) in $CDCl_3$ (0.8 mL) was monitored by ^{1}H-NMR. After 10 min formation of EtOH was detected indicating transesterification of **1a** with **4**. A sole CH_2 signal by the B-O-CH_2CH_3 group indicates the formation of a BINOL-**1a** diester, such as **12** (Figure 4 in the paper). As the B-O-C$\underline{H}_2$CH$_3$ and the CH$_3$C$\underline{H}_2$-OH signals are present in the NMR sample over time indicates that the **1a** + **4** → **12** reaction is an equilibrium process.

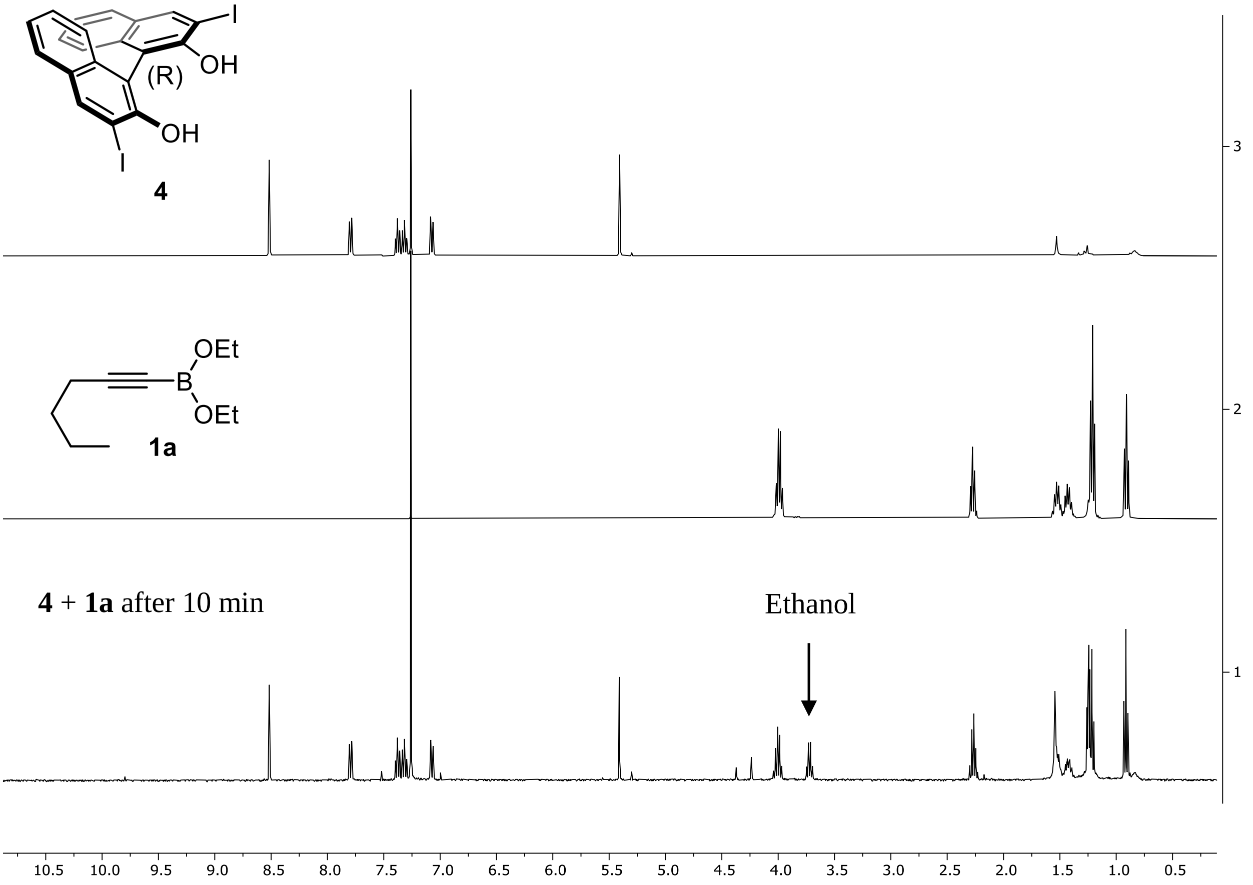

3. References

1. a) Argintaru, O. A.; Ryu, D.; Aron, I.; Molander, G. A. *Angew. Chem. Int. Ed.* **2013**, *52*, 13656-13660. b) Jayarajan, R.; Kireilis, T.; Eriksson, L.; Szabo, K. J. *Chem. Eur. J.* **2022**, doi.org/10.1002/chem.202202059.

2. Milburn, R. R.; Hussain, S. M. S.; Prien, O.; Ahmed, Z.; Snieckus, V. *Org. Lett.* **2007**, *9*, 4403-4406.

3. a) Jiang, Y.; Diagne, A.B.; Thomson, R.J.; Schaus, S. E. *J. Am. Chem. Soc.*, **2017**, *139*, 1998-2005. b) Brown, H. C.; Bhat, N.G.; Srebnik, M. *Tetrahedron Lett*. **1988**, *29*, 2631-2634.

4. NMR Spectra: ^{1}H NMR (500 MHz, $CDCl_3$) of compound **5a**

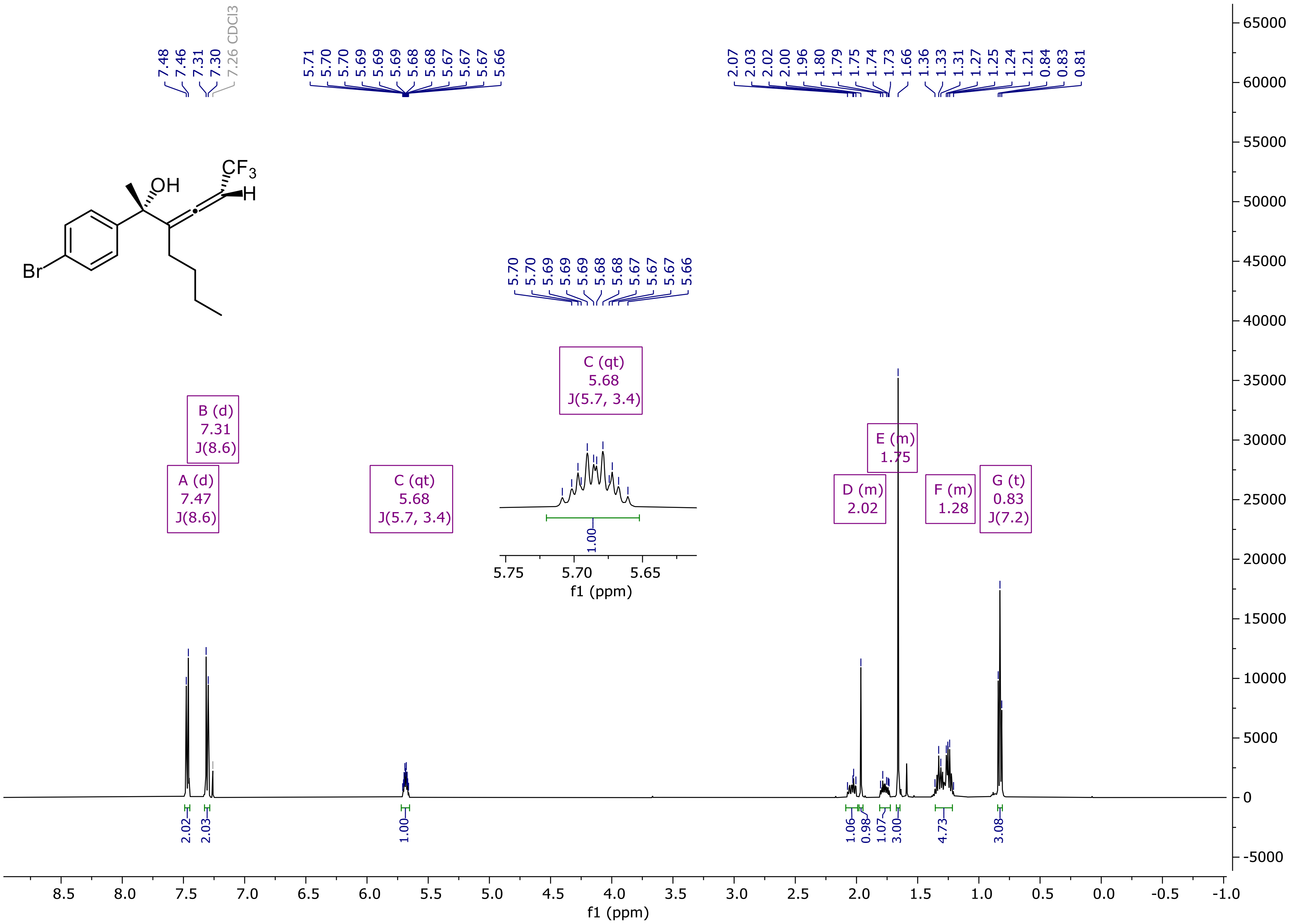

^{13}C NMR (126 MHz, $CDCl_3$) of compound **5a**

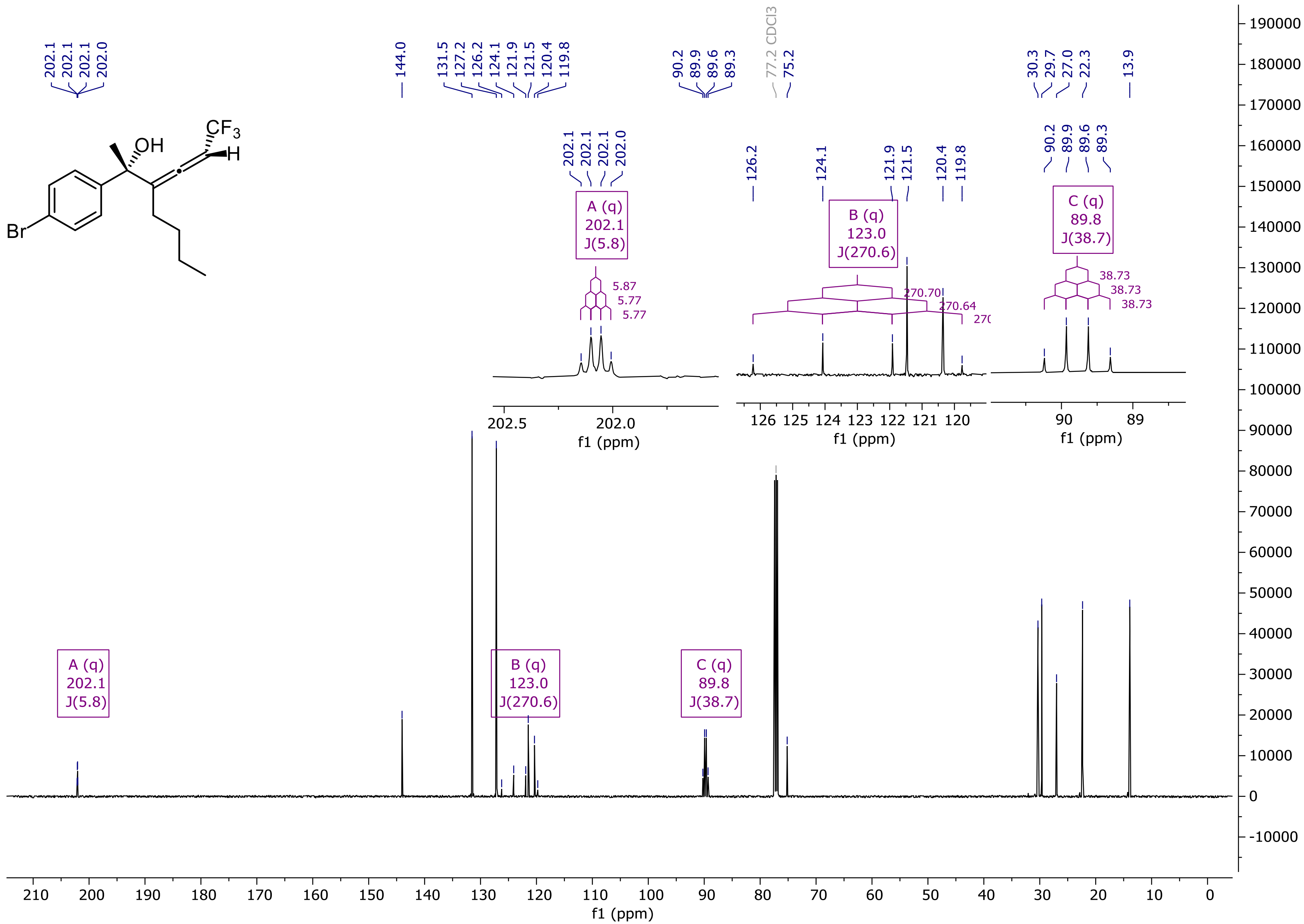

^{19}F NMR (377 MHz, CDCl$_3$) of compound **5a**

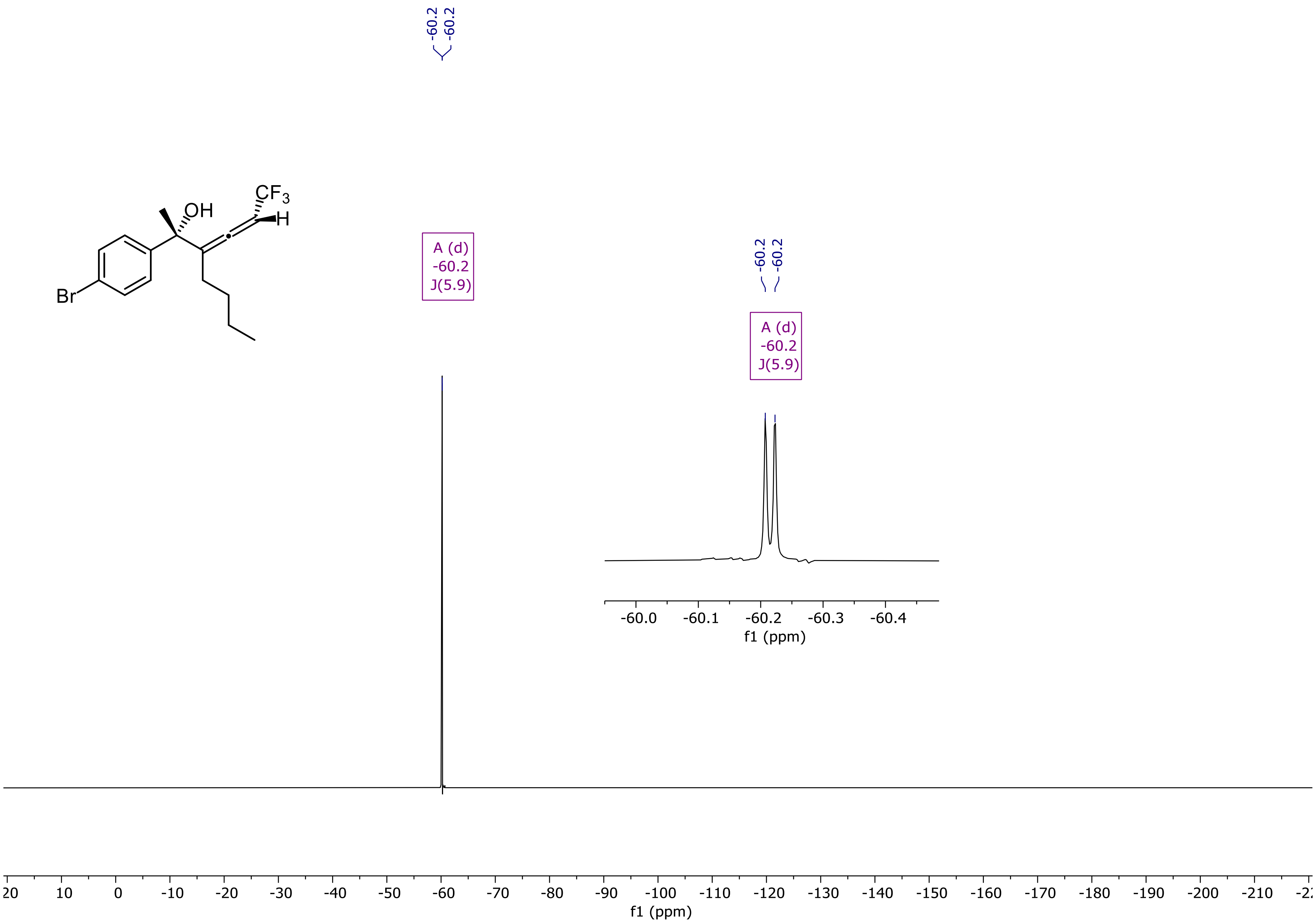

^{1}H NMR (500 MHz, $CDCl_3$) of compound **5b**

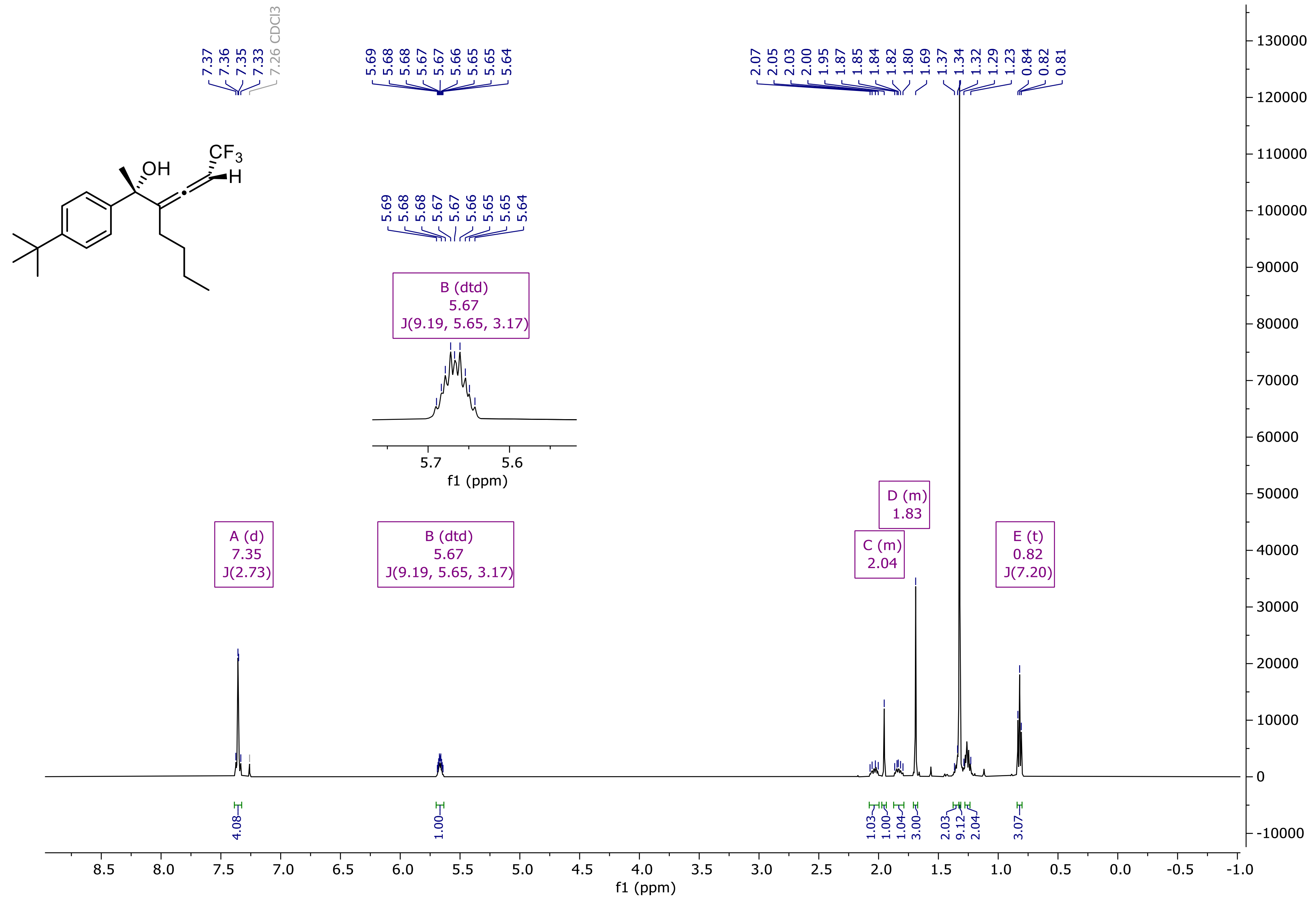

^{13}C NMR (126 MHz, $CDCl_3$) of compound **5b**

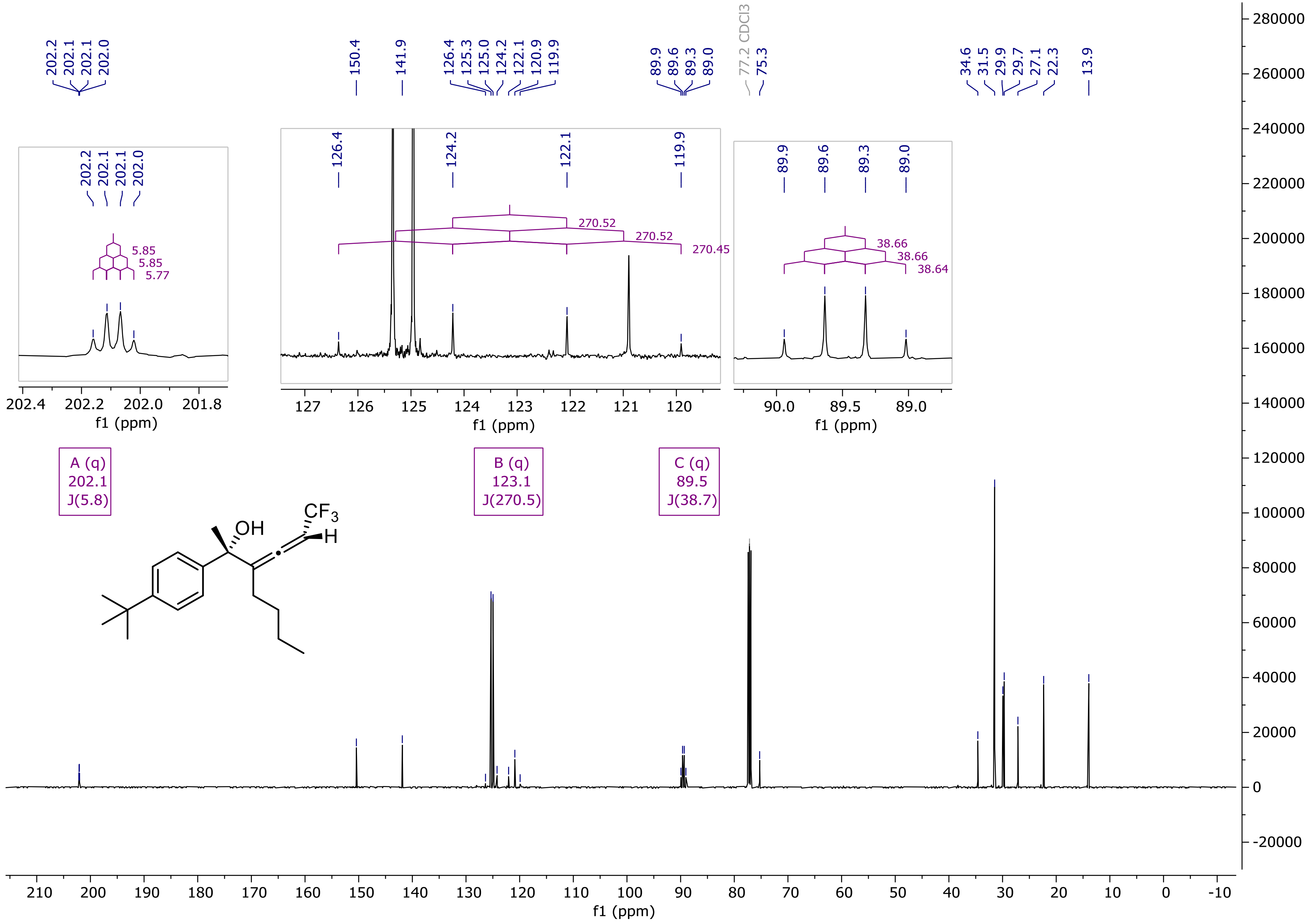

^{19}F NMR (377 MHz, $CDCl_3$) of compound **5b**

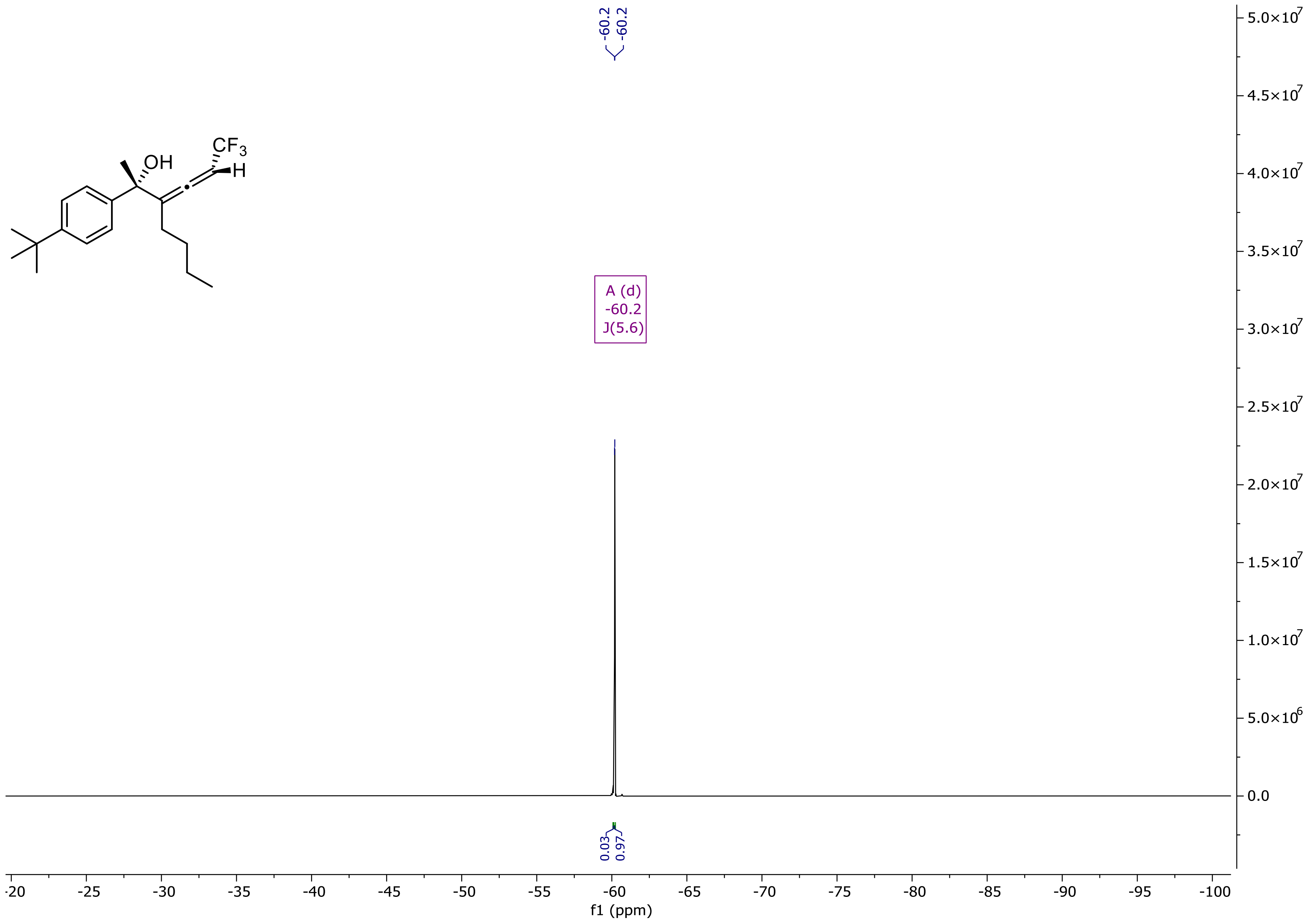

^{1}H NMR (500 MHz, $CDCl_3$) of compound **5c**

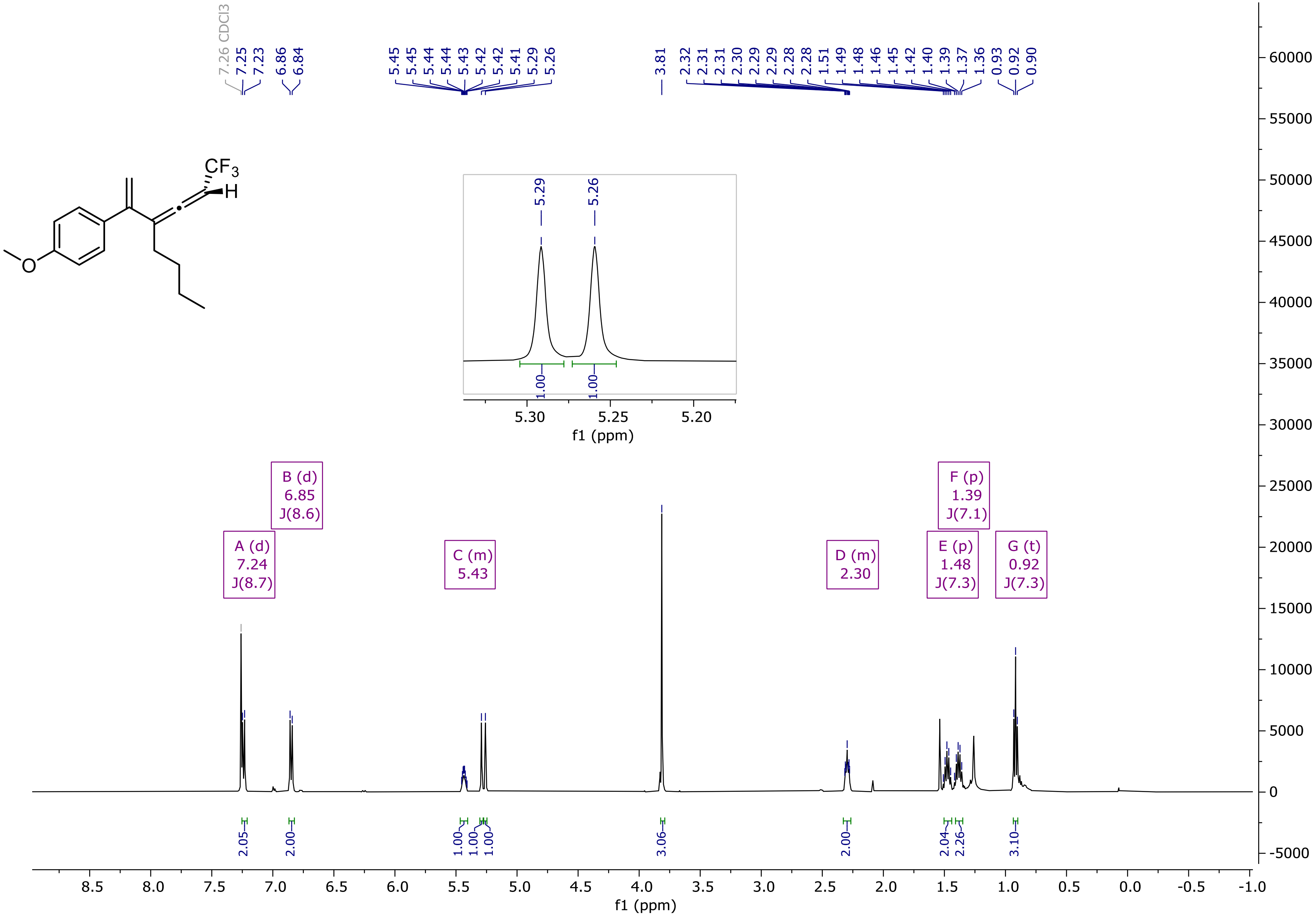

^{13}C NMR (126 MHz, $CDCl_3$) of compound **5c**

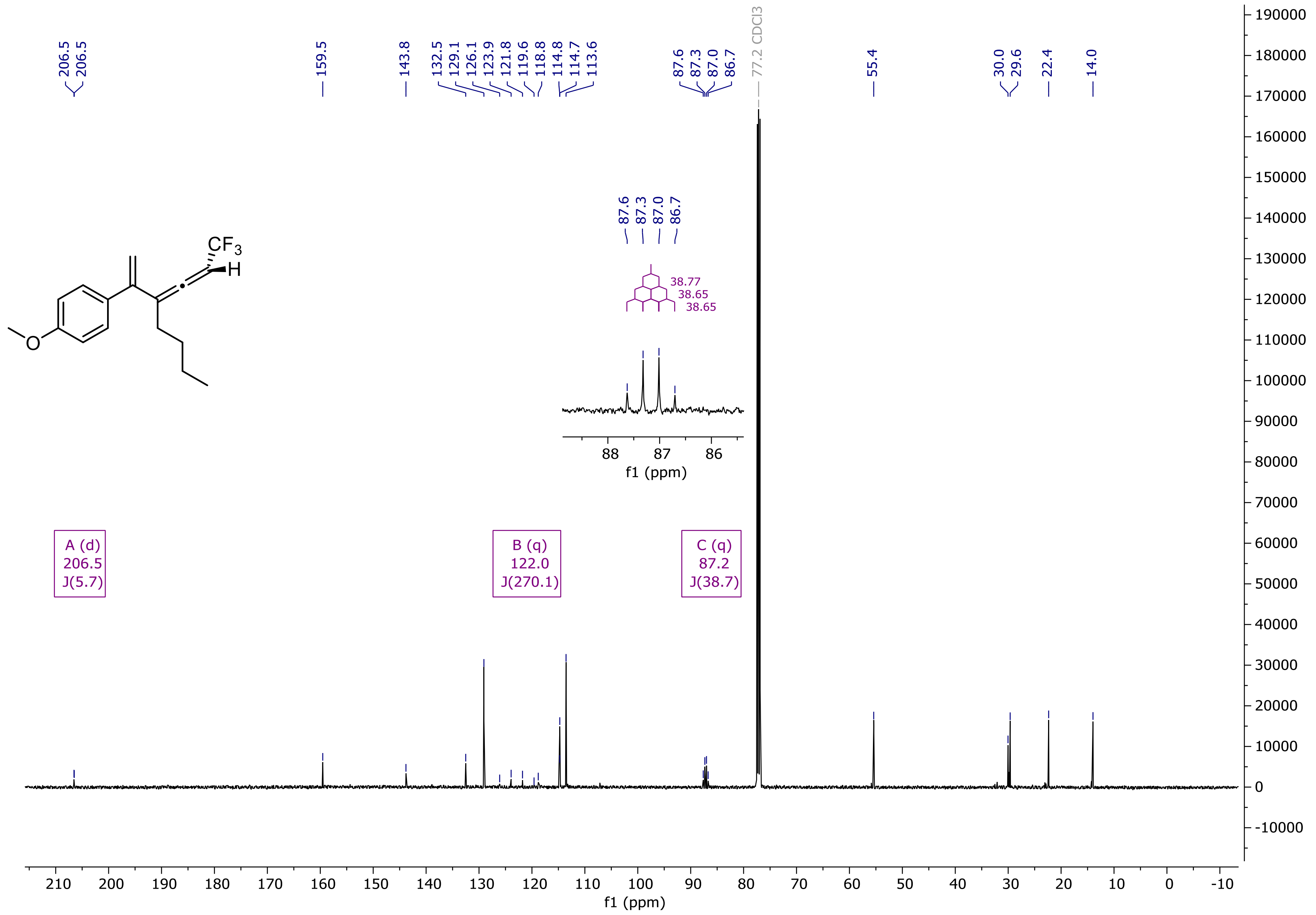

^{19}F NMR (377 MHz, $CDCl_3$) of compound **5c**

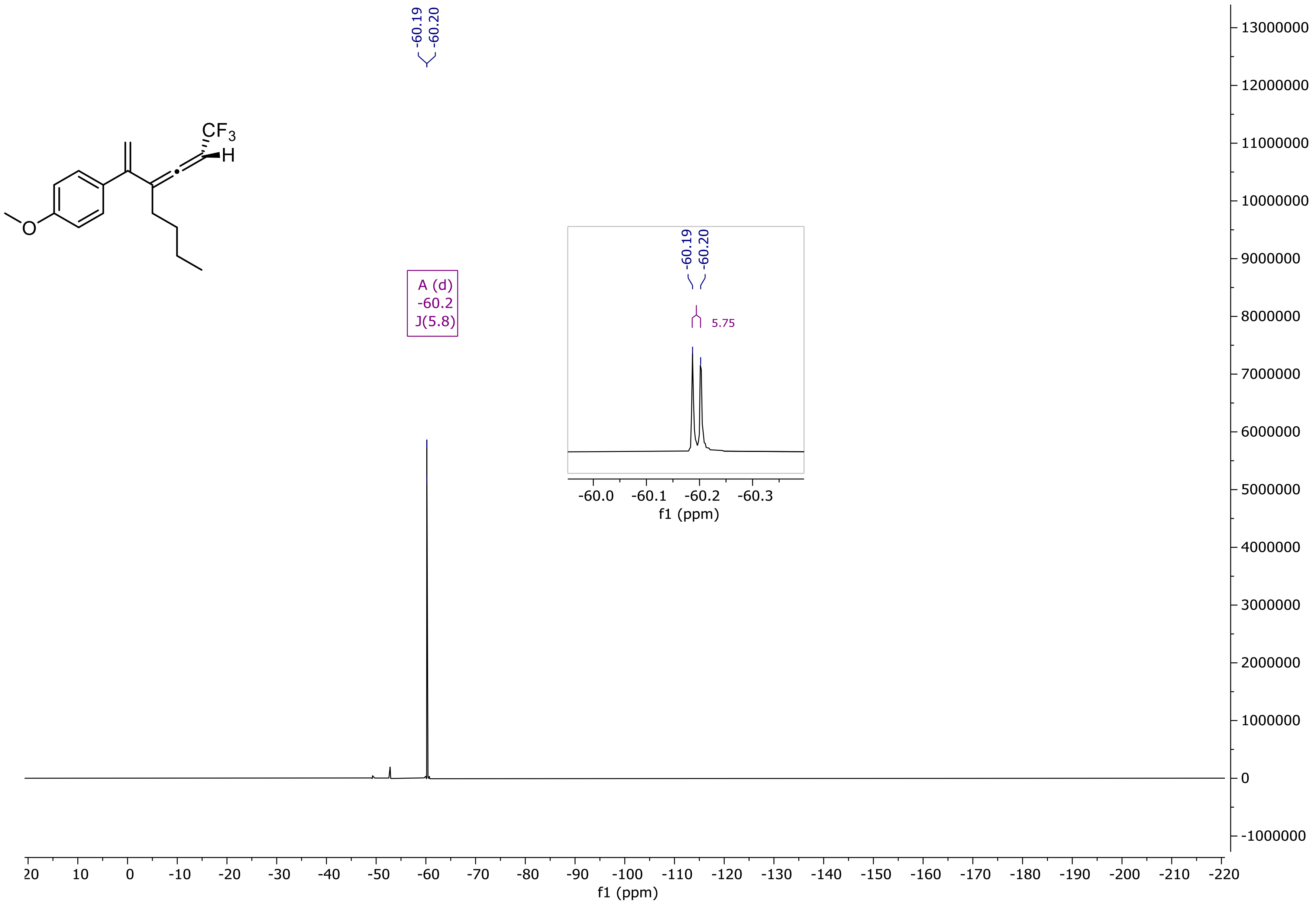

^{1}H NMR (500 MHz, $CDCl_3$) of compound **5d**

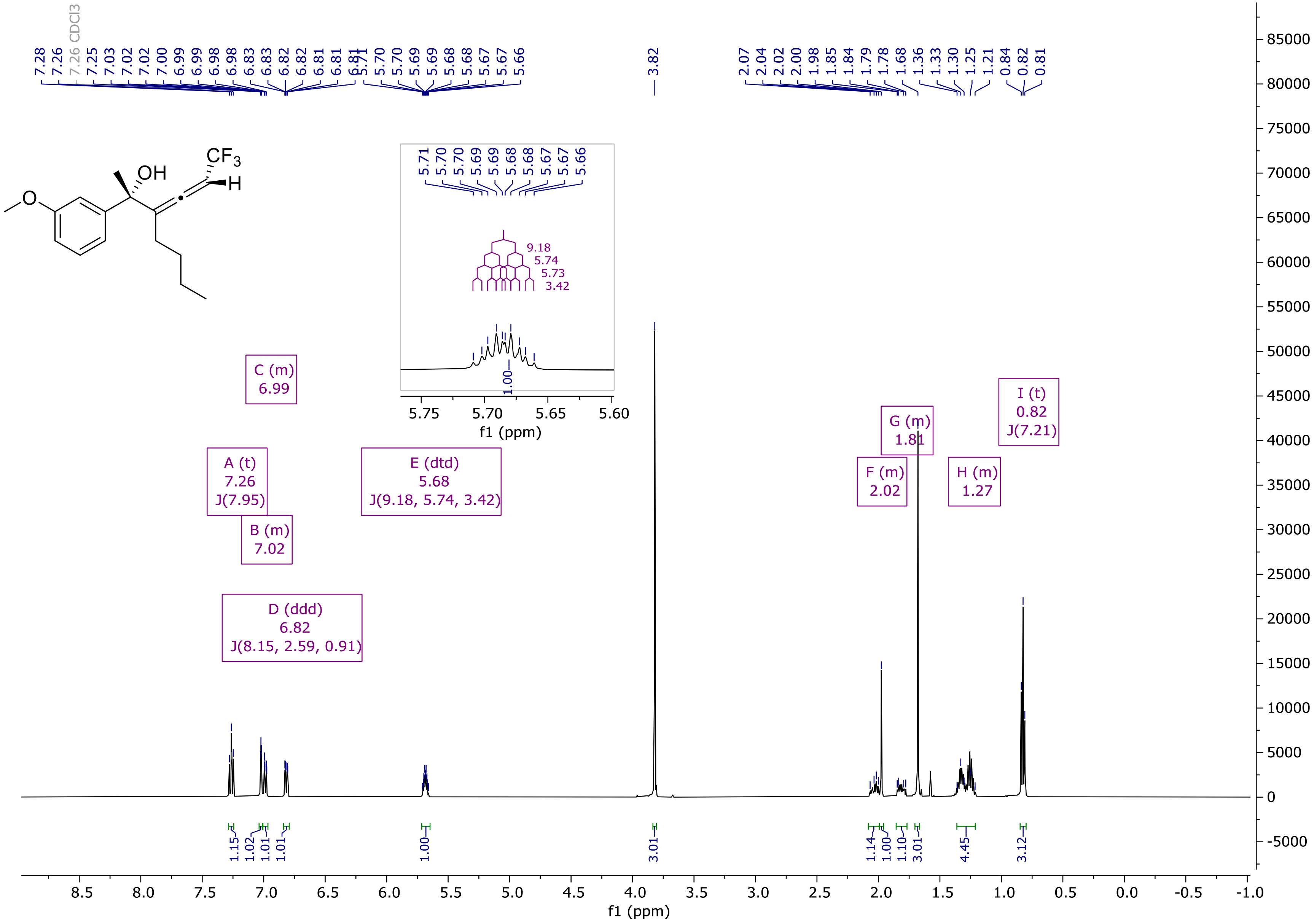

^{13}C NMR (126 MHz, $CDCl_3$) of compound **5d**

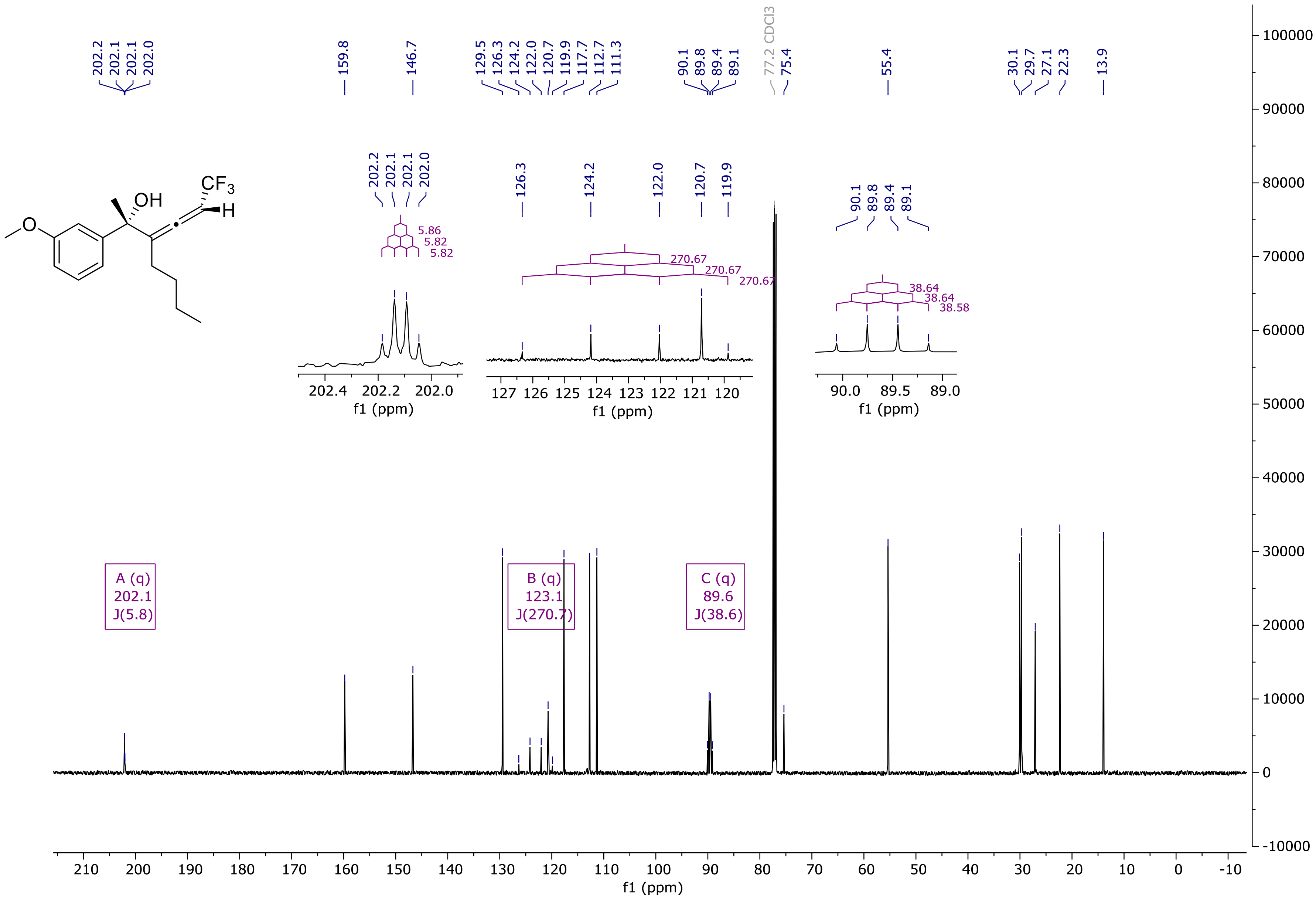

^{19}F NMR (377 MHz, $CDCl_3$) of compound **5d**

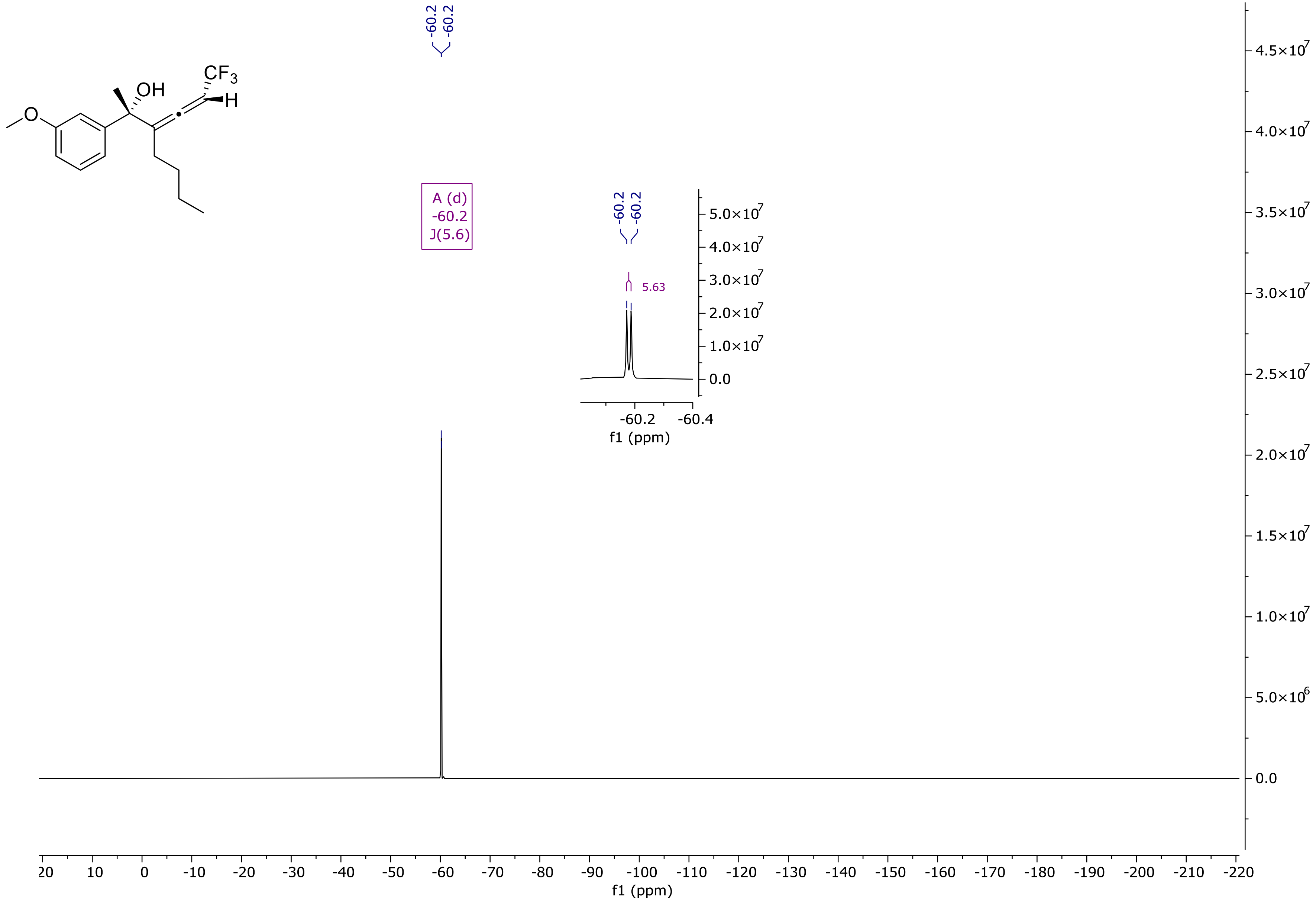

^{1}H NMR (400 MHz, $CDCl_3$) of compound **5e**

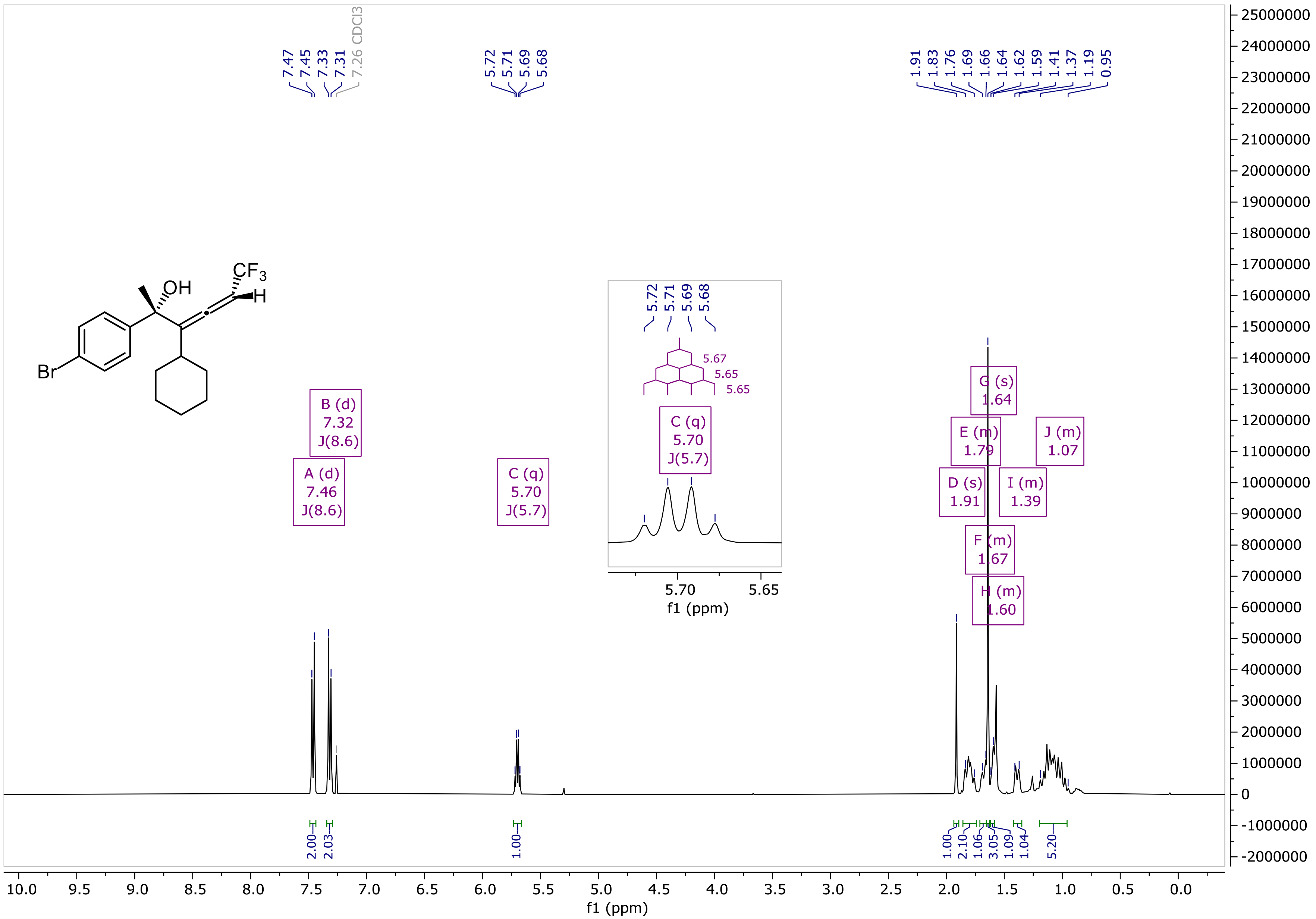

^{13}C NMR (101 MHz, $CDCl_3$) of compound **5e**

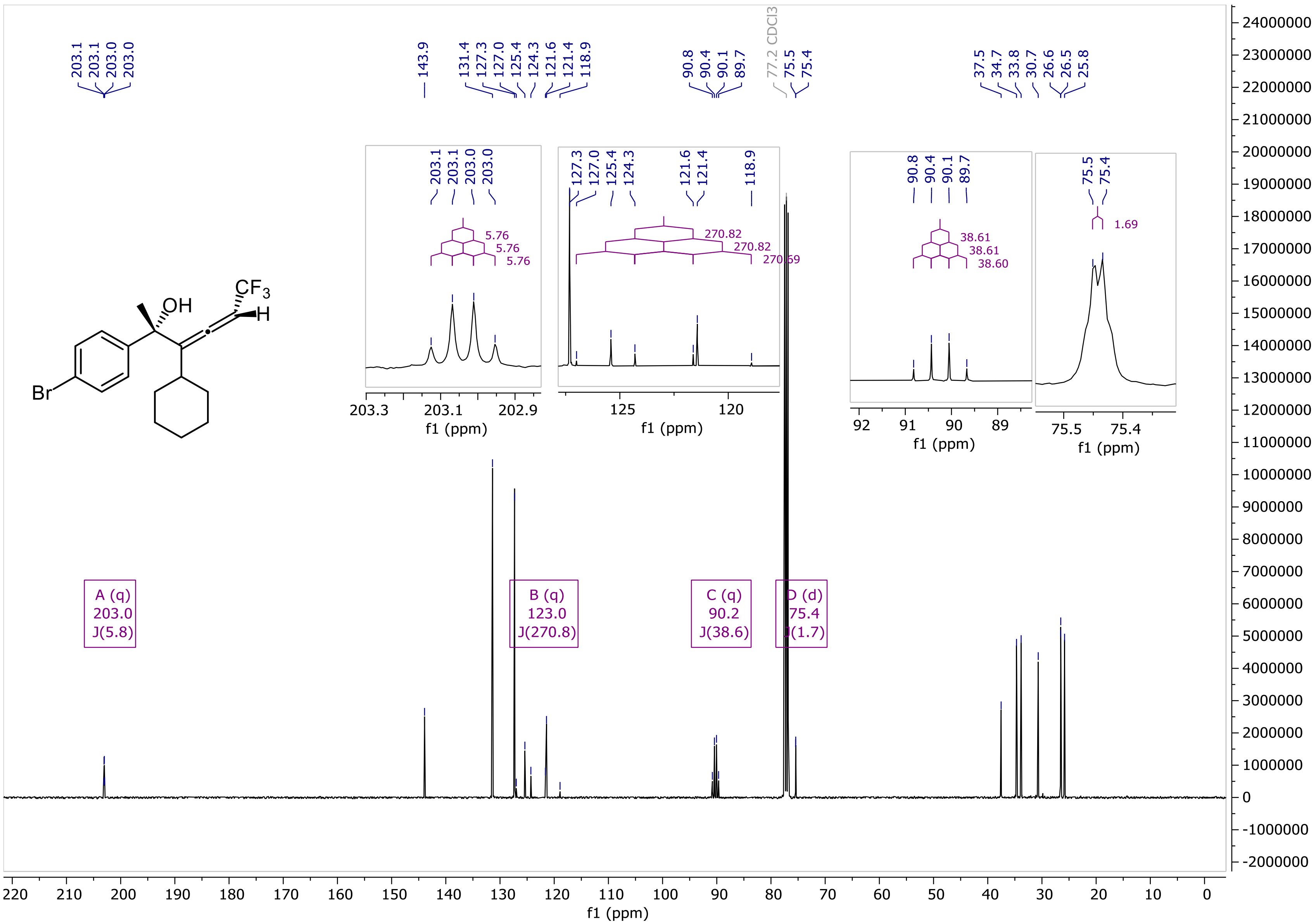

^{19}F NMR (377 MHz, $CDCl_3$) of compound **5e**

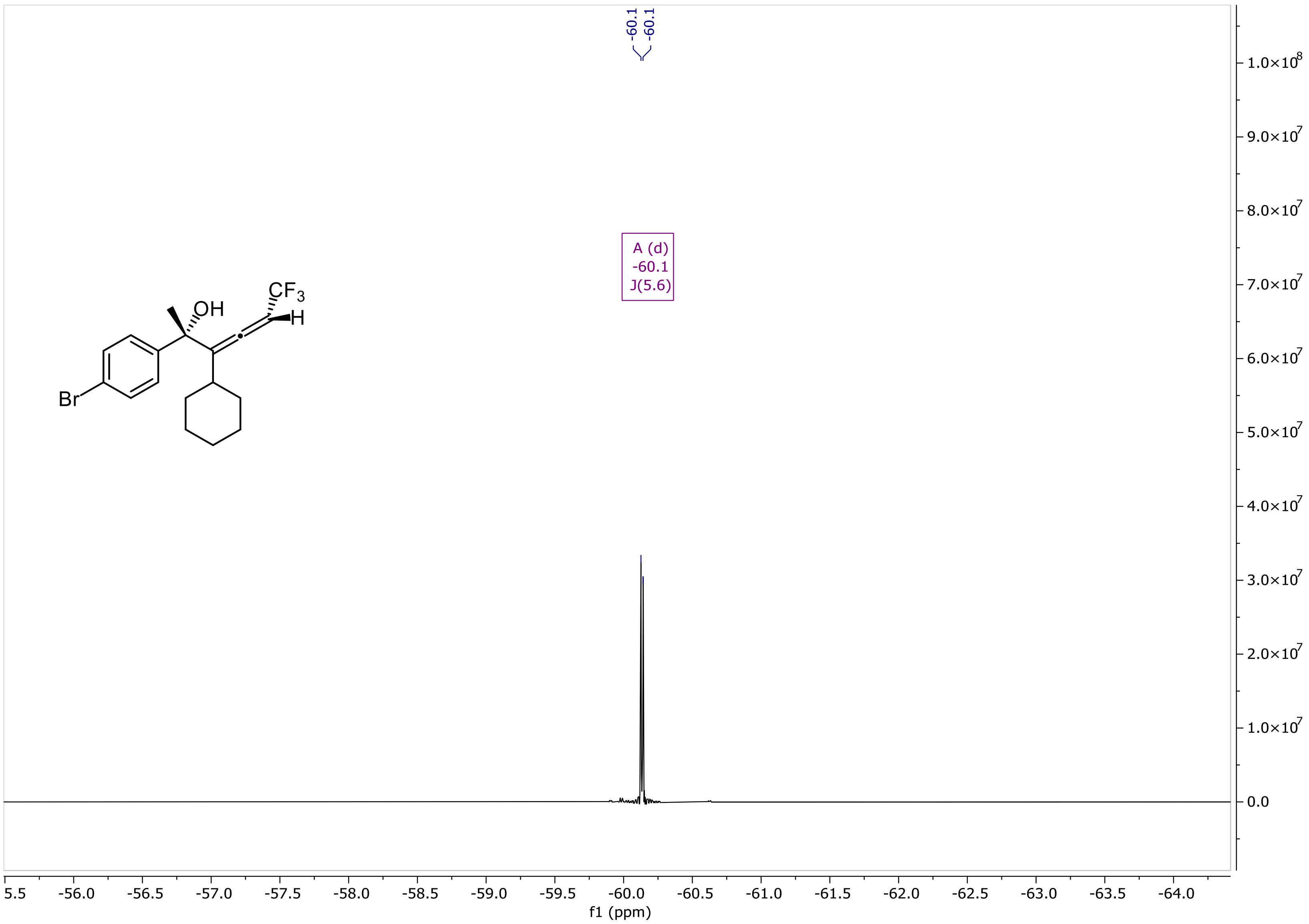

^{1}H NMR (400 MHz, $CDCl_3$) of compound **5f**

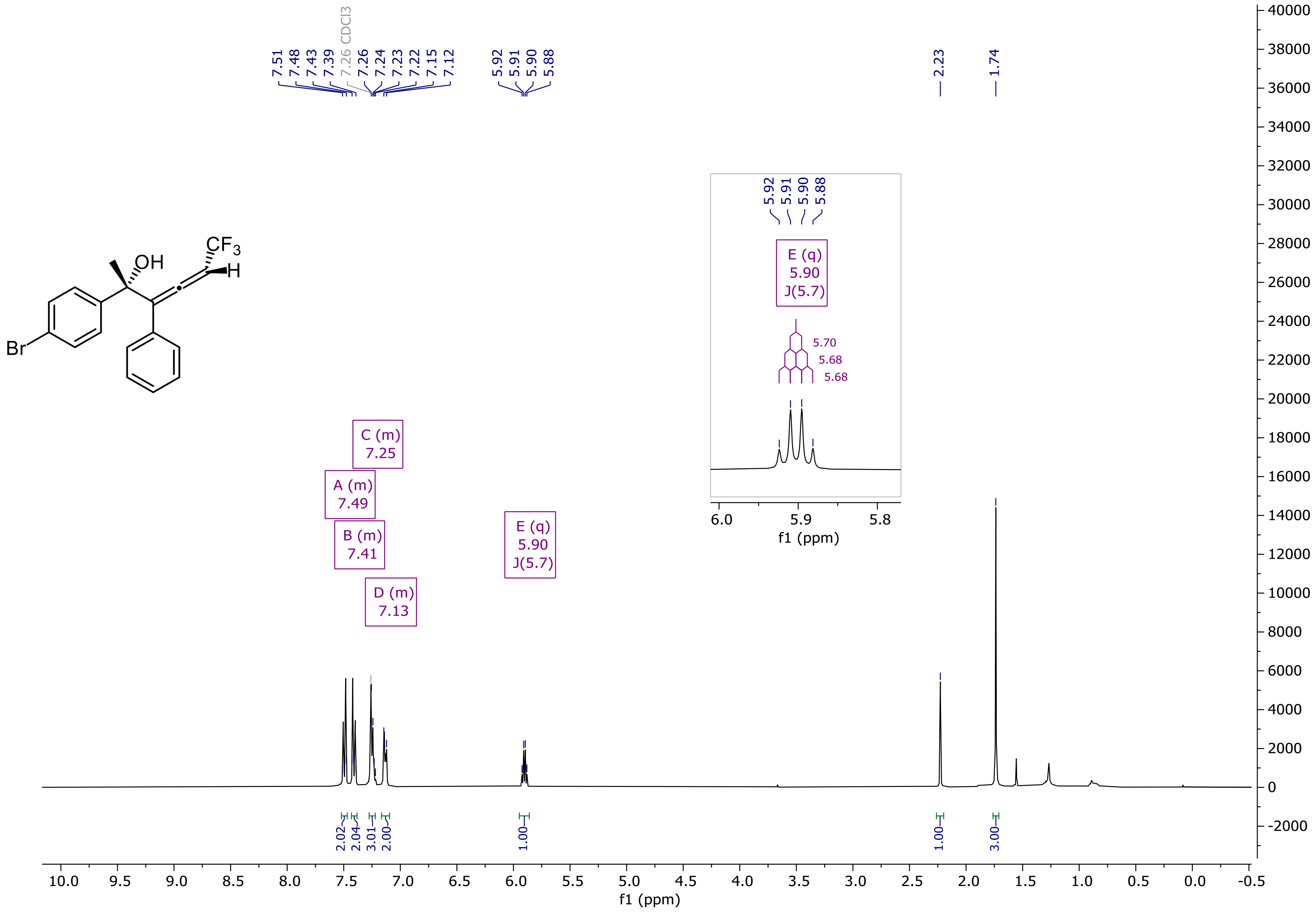

^{13}C NMR (101 MHz, $CDCl_3$) of compound **5f**

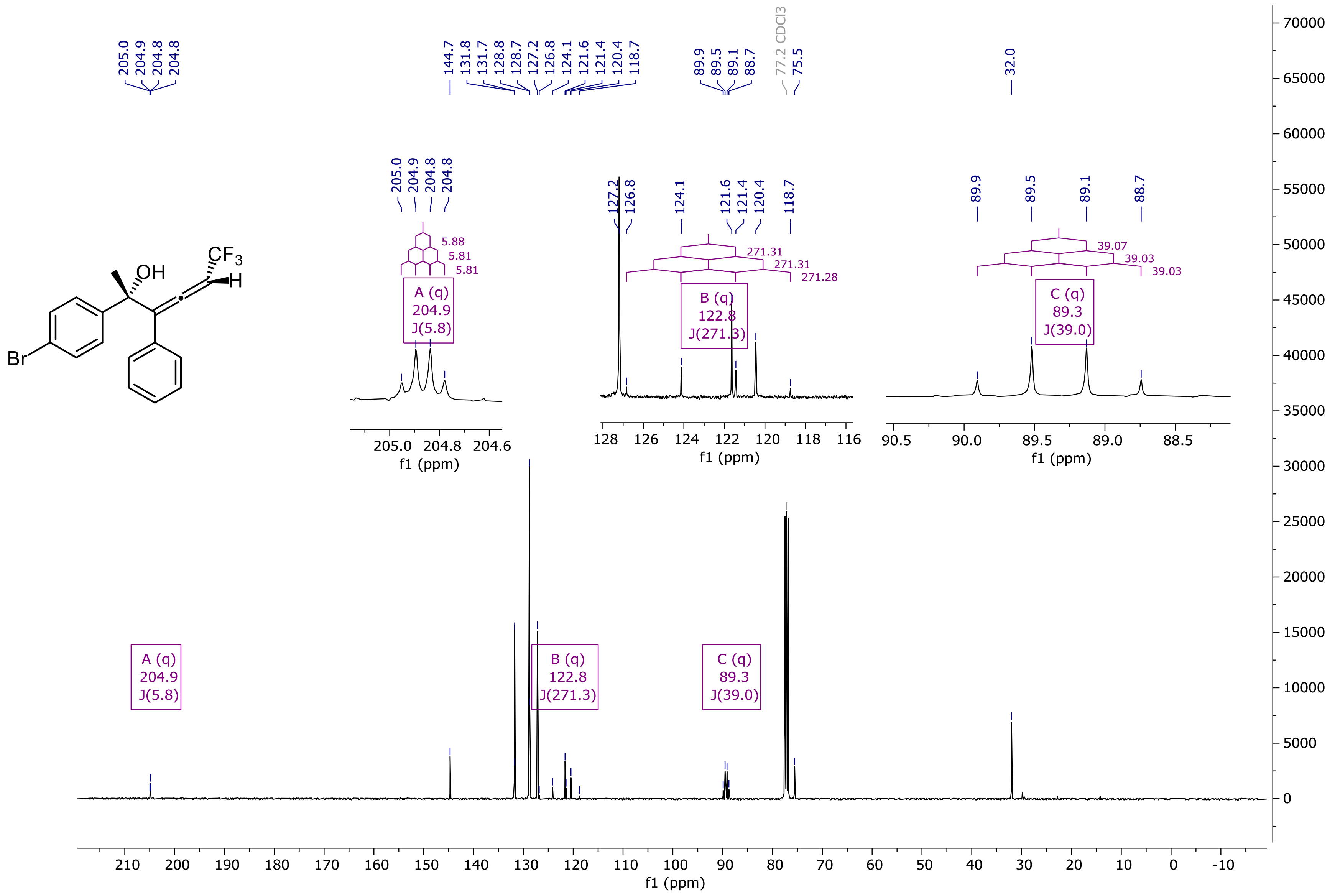

^{19}F NMR (377 MHz, $CDCl_3$) of compound **5f**

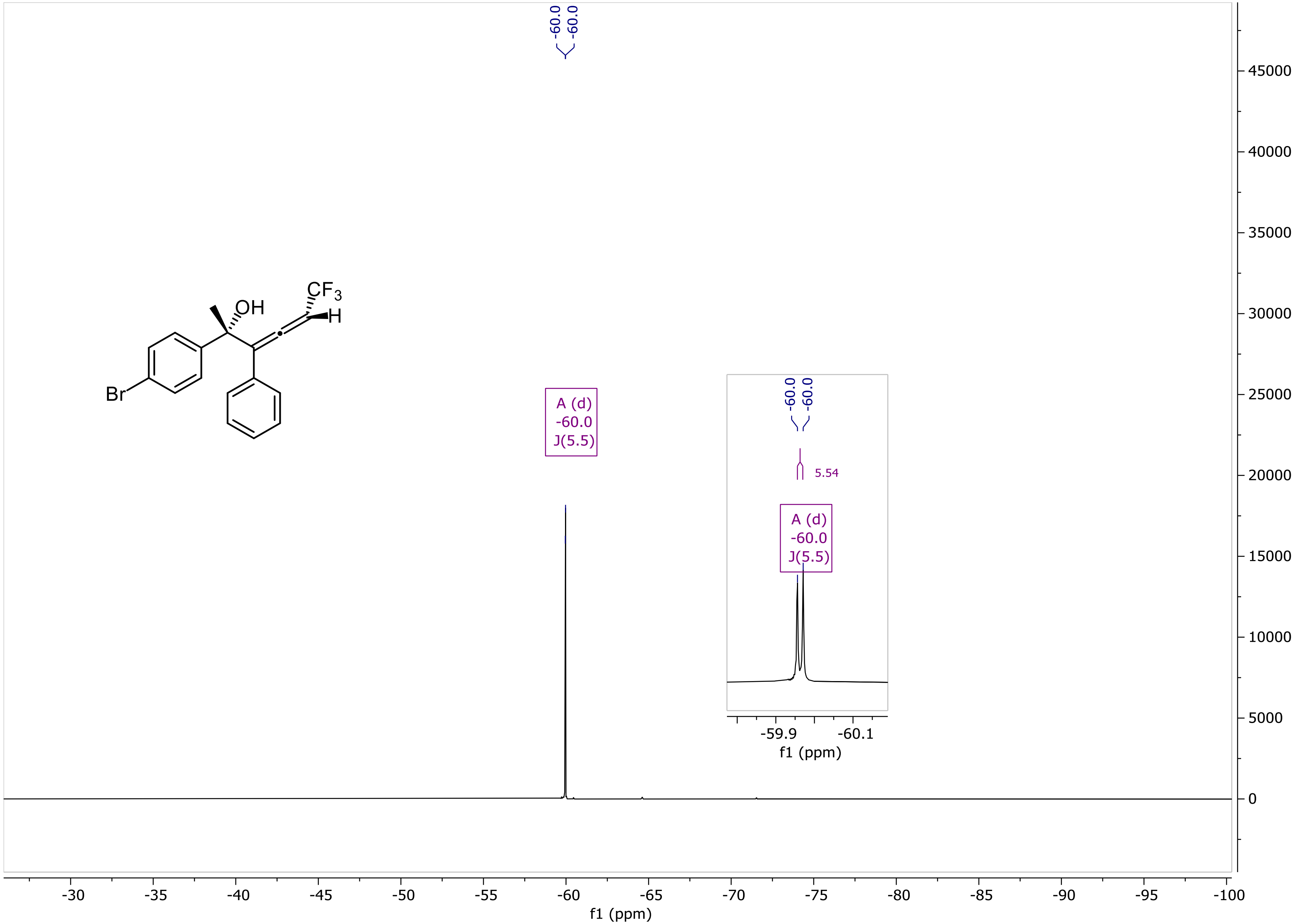

^{1}H NMR (400 MHz, $CDCl_3$) of compound **5g**

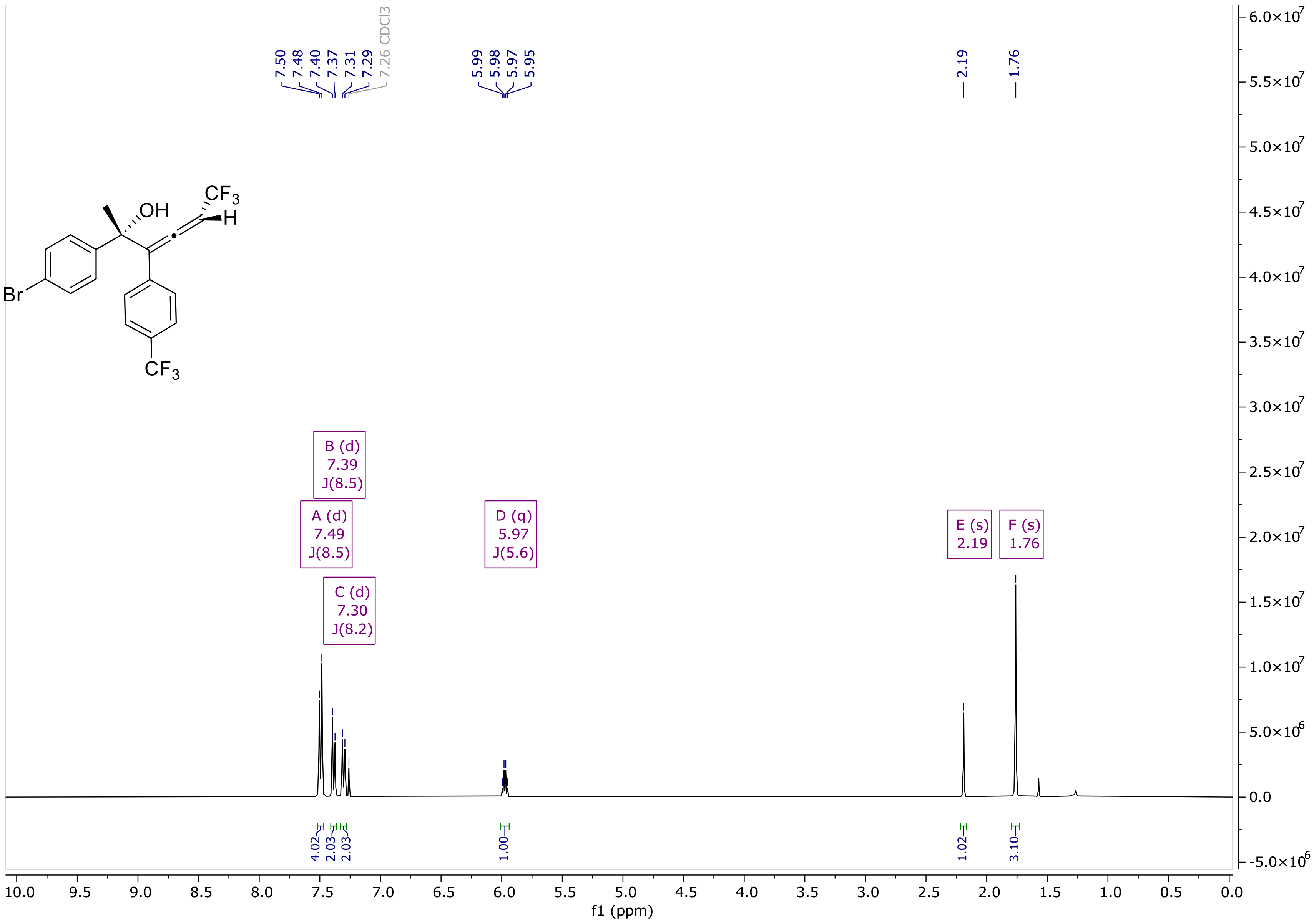

^{13}C NMR (101 MHz, $CDCl_3$) of compound **5g**

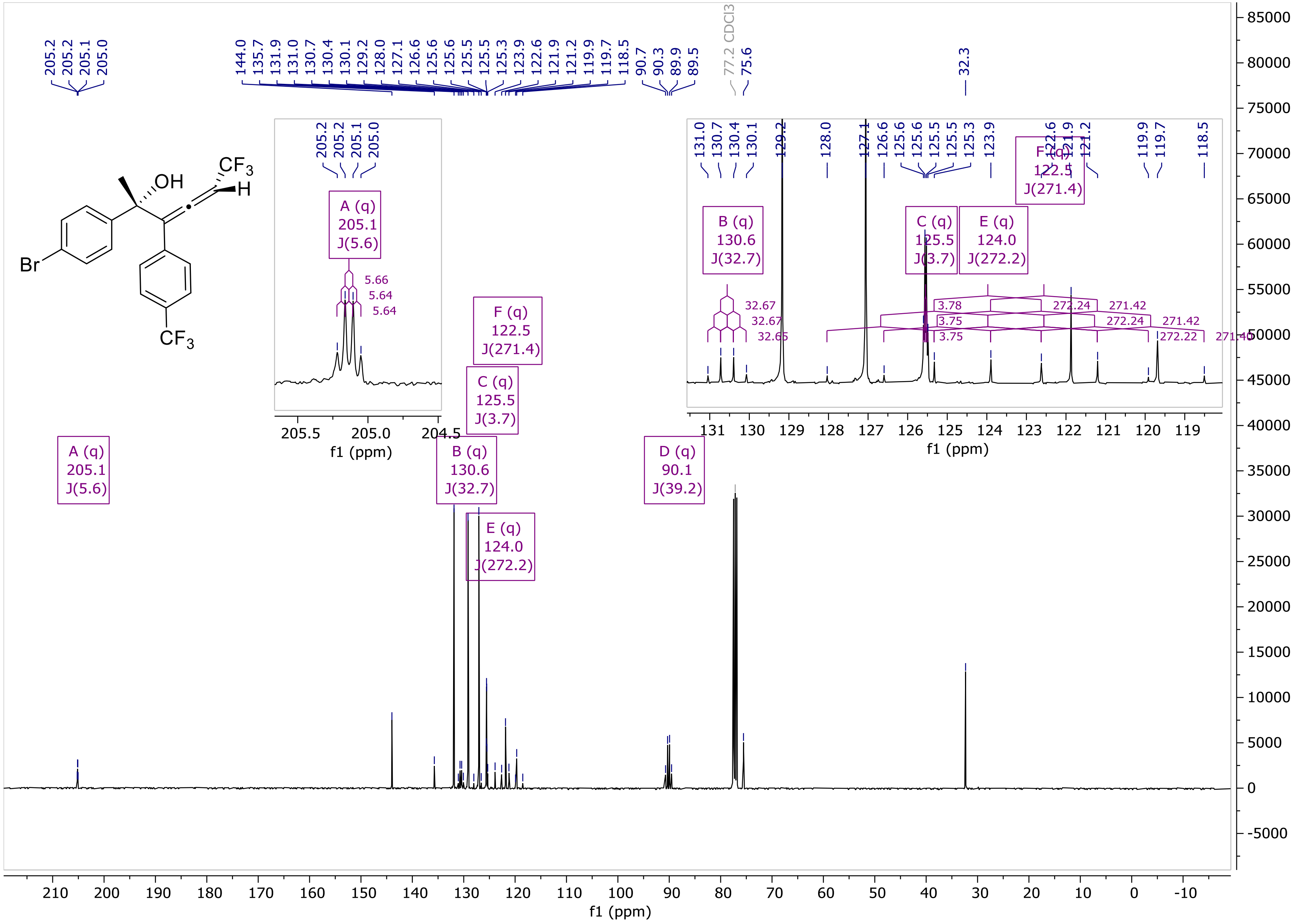

^{19}F NMR (377 MHz, $CDCl_3$) of compound **5g**

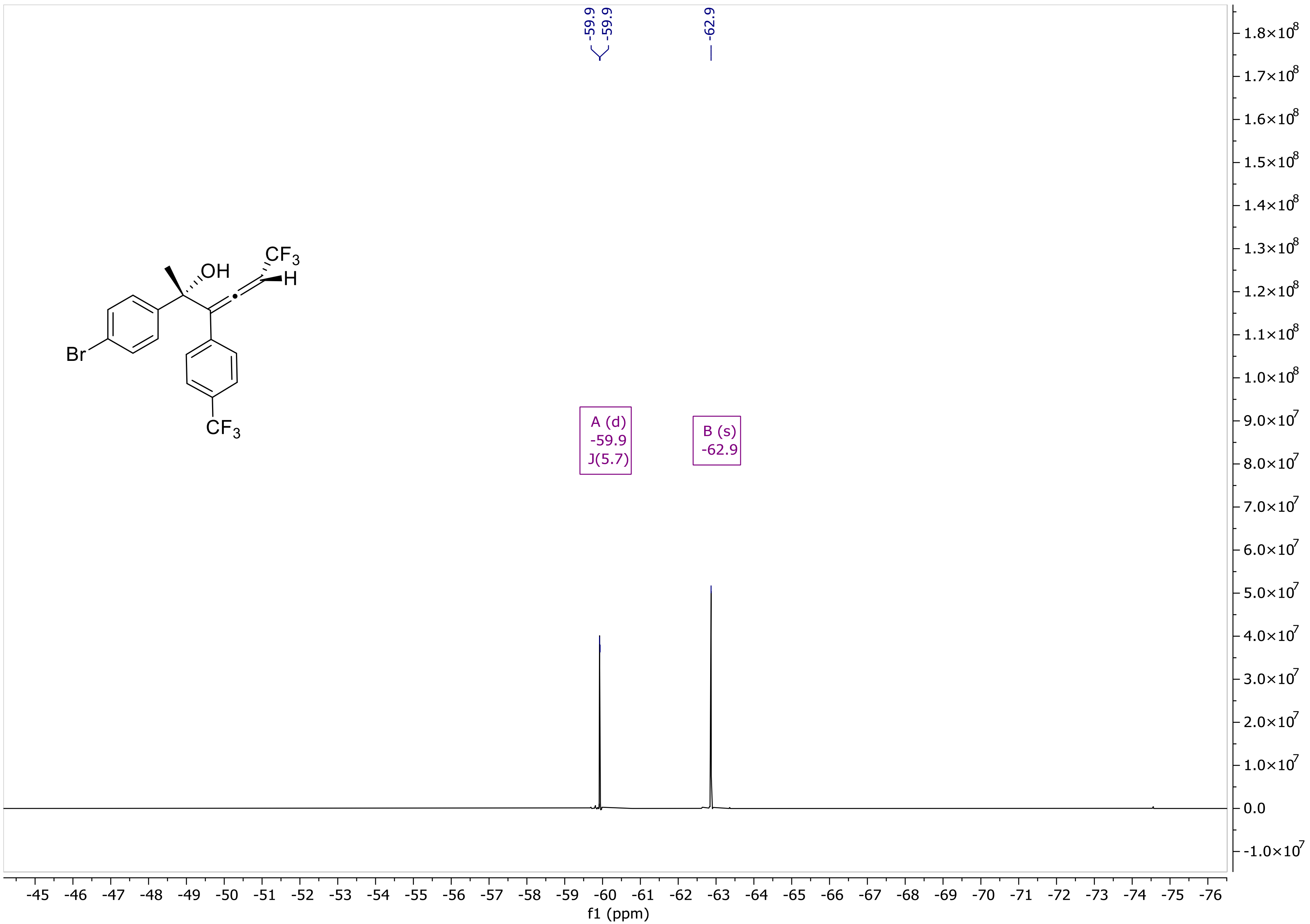

^{1}H NMR (500 MHz, $CDCl_3$) of compound **5h**

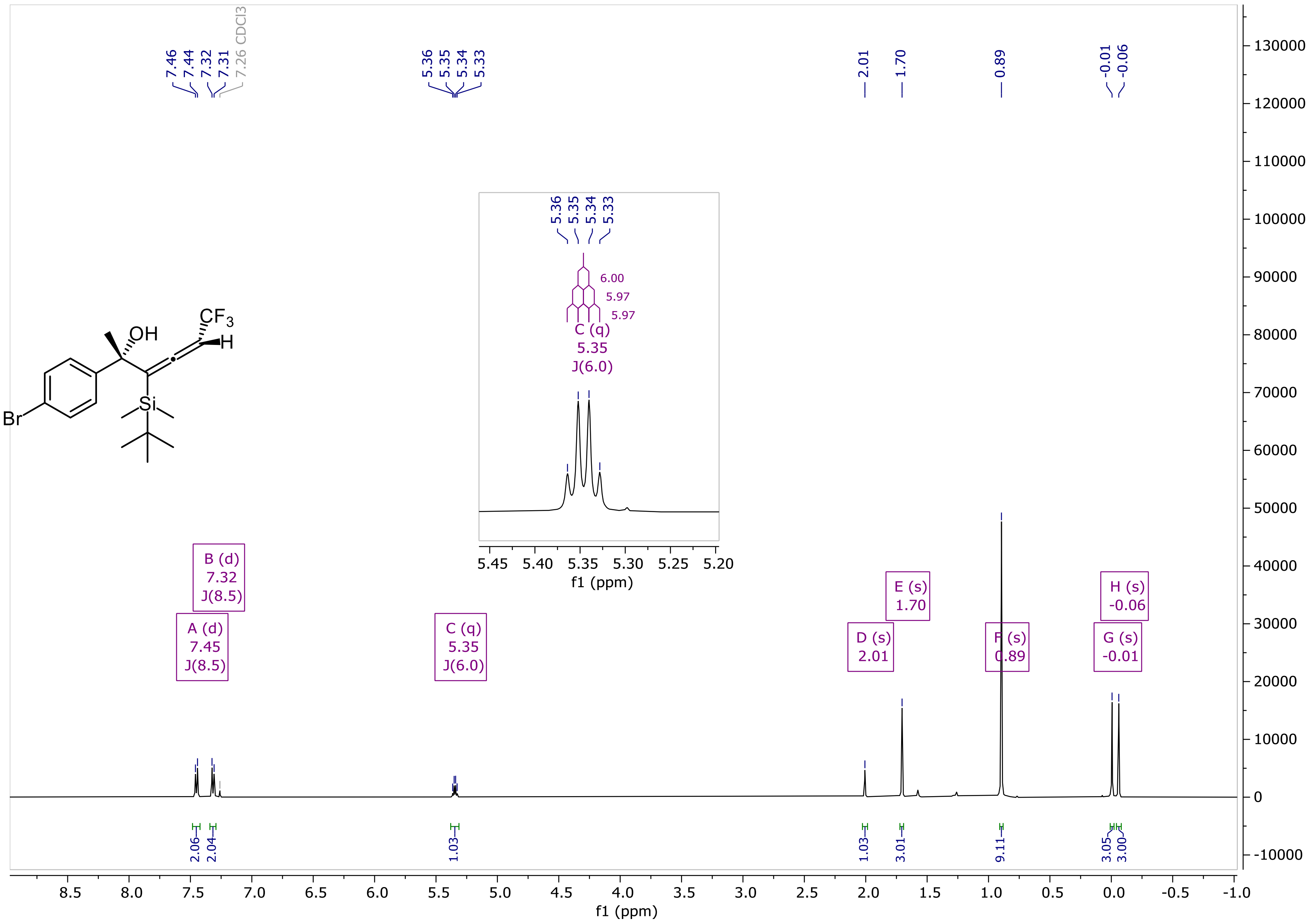

^{13}C NMR (126 MHz, $CDCl_3$) of compound **5h**

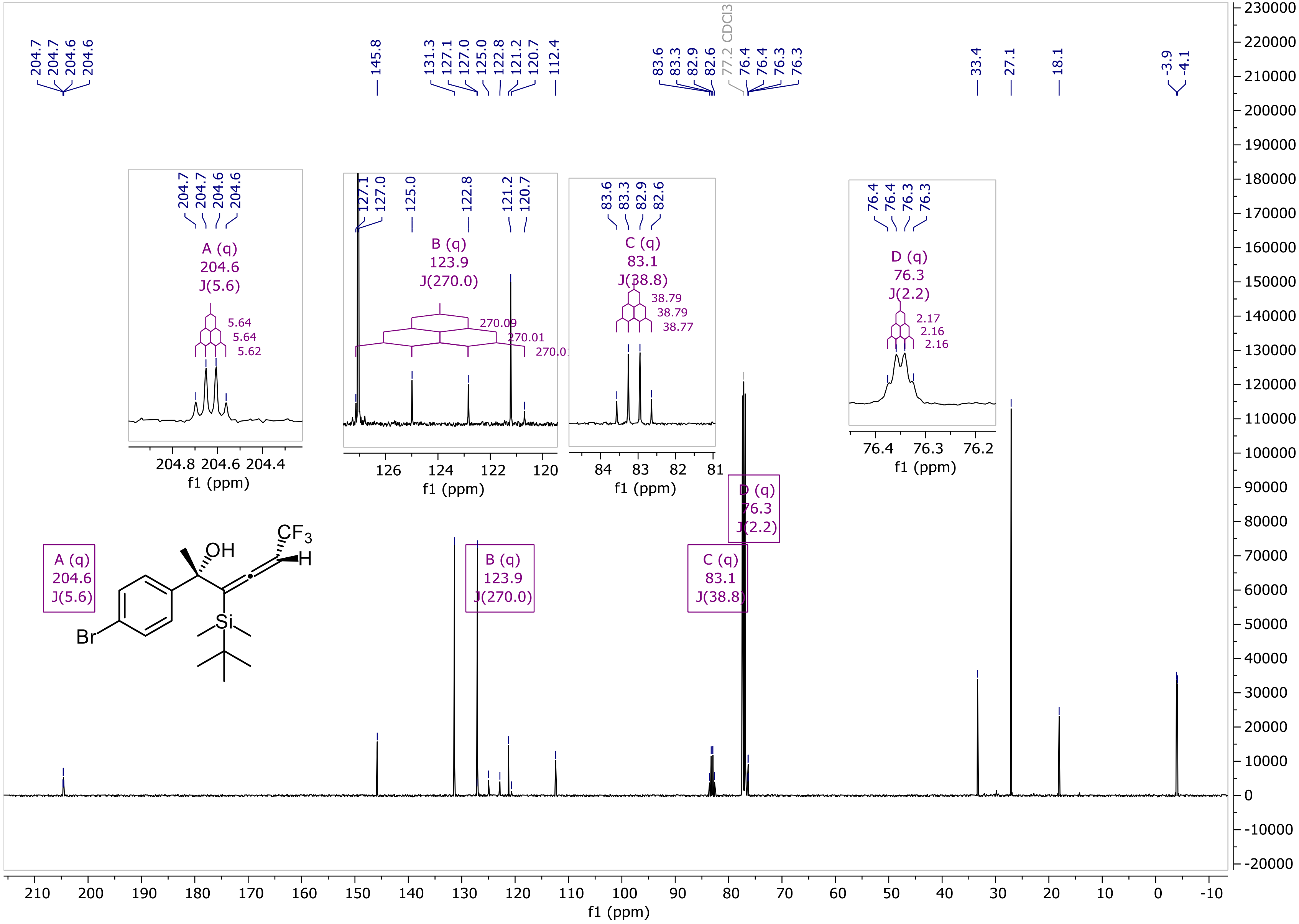

^{19}F NMR (377 MHz, $CDCl_3$) of compound **5h**

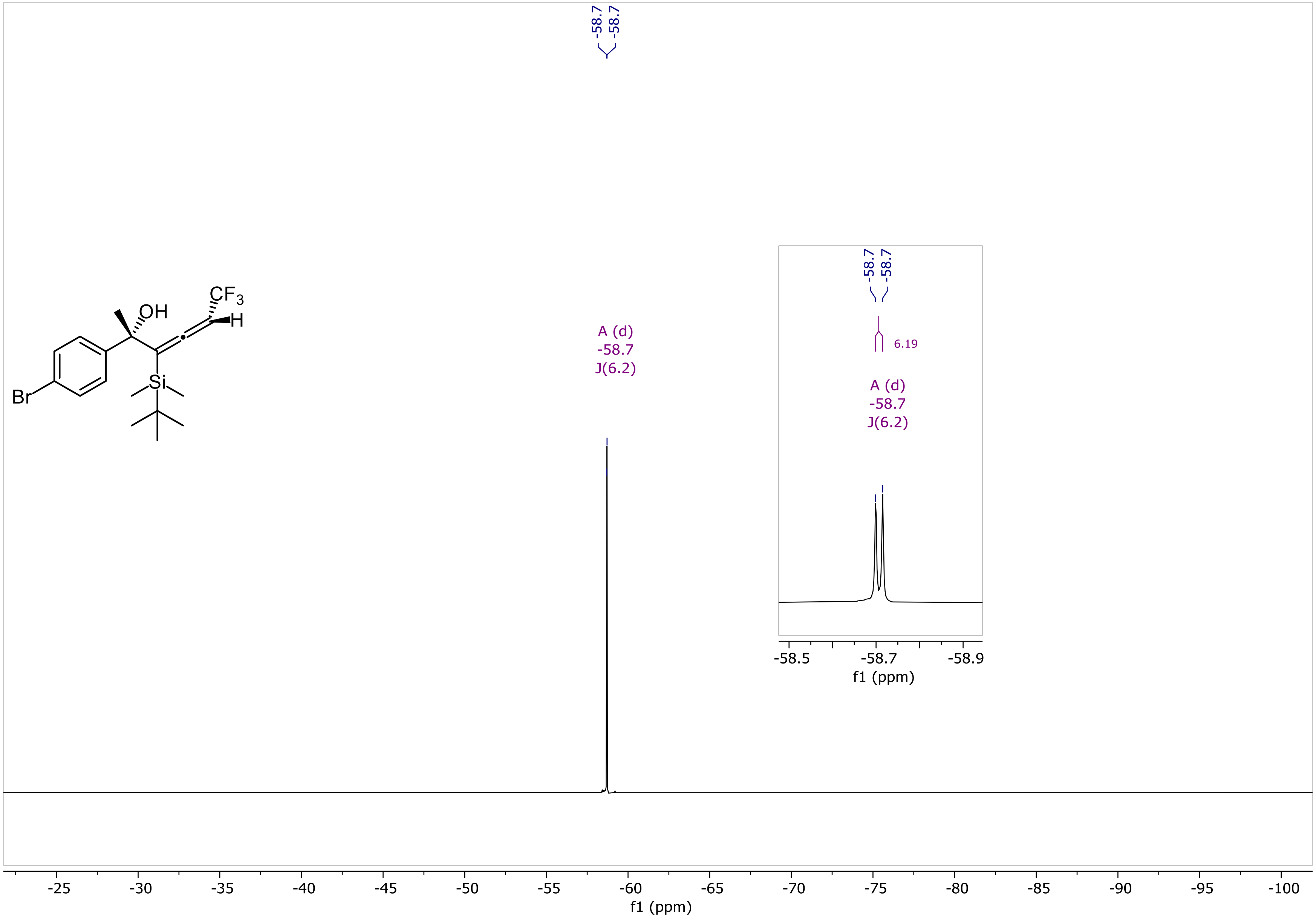

^{29}Si NMR (99 MHz, CDCl$_3$) of compound **5h**

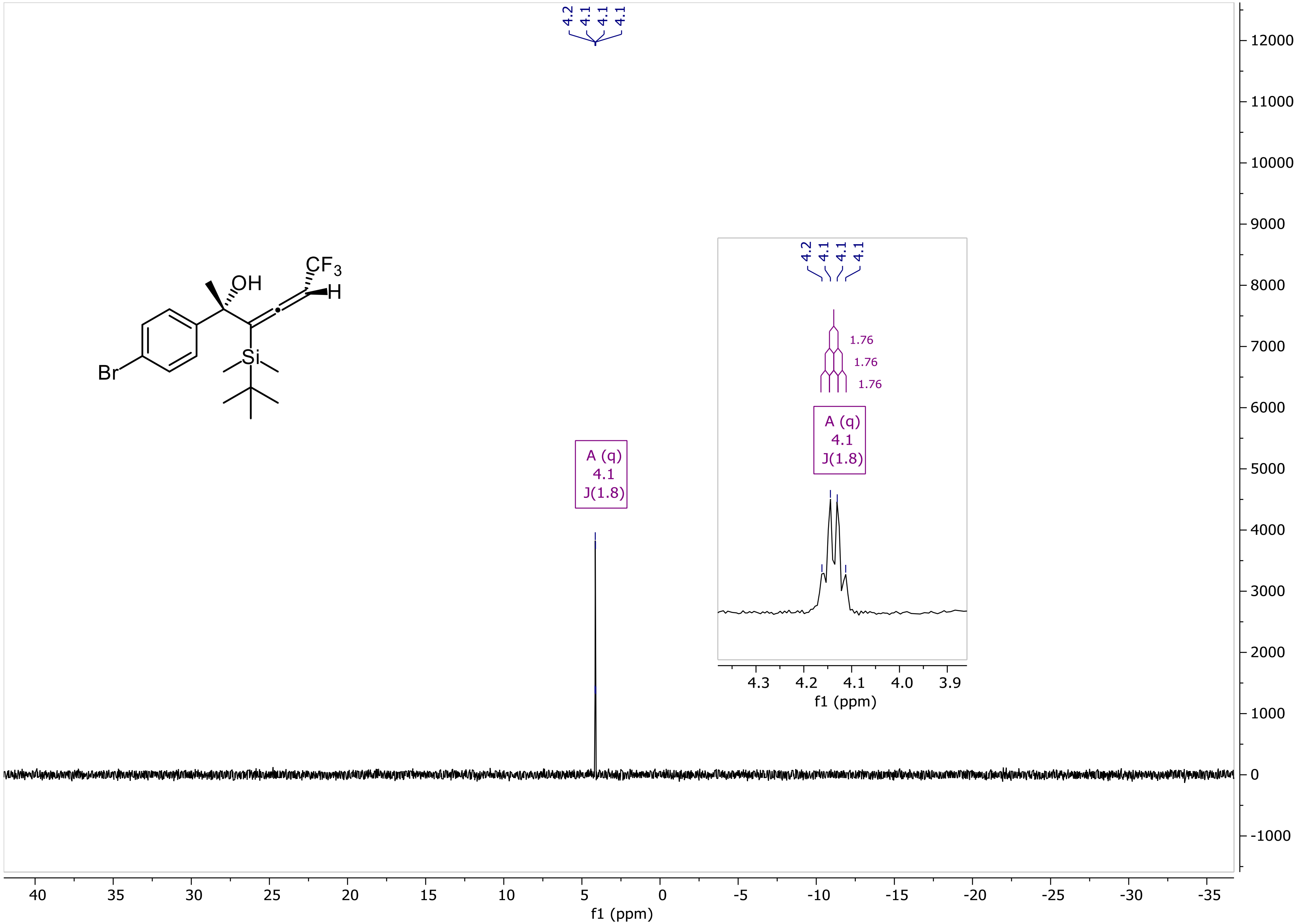

^{1}H NMR (400 MHz, $CDCl_3$) of compound **5i**

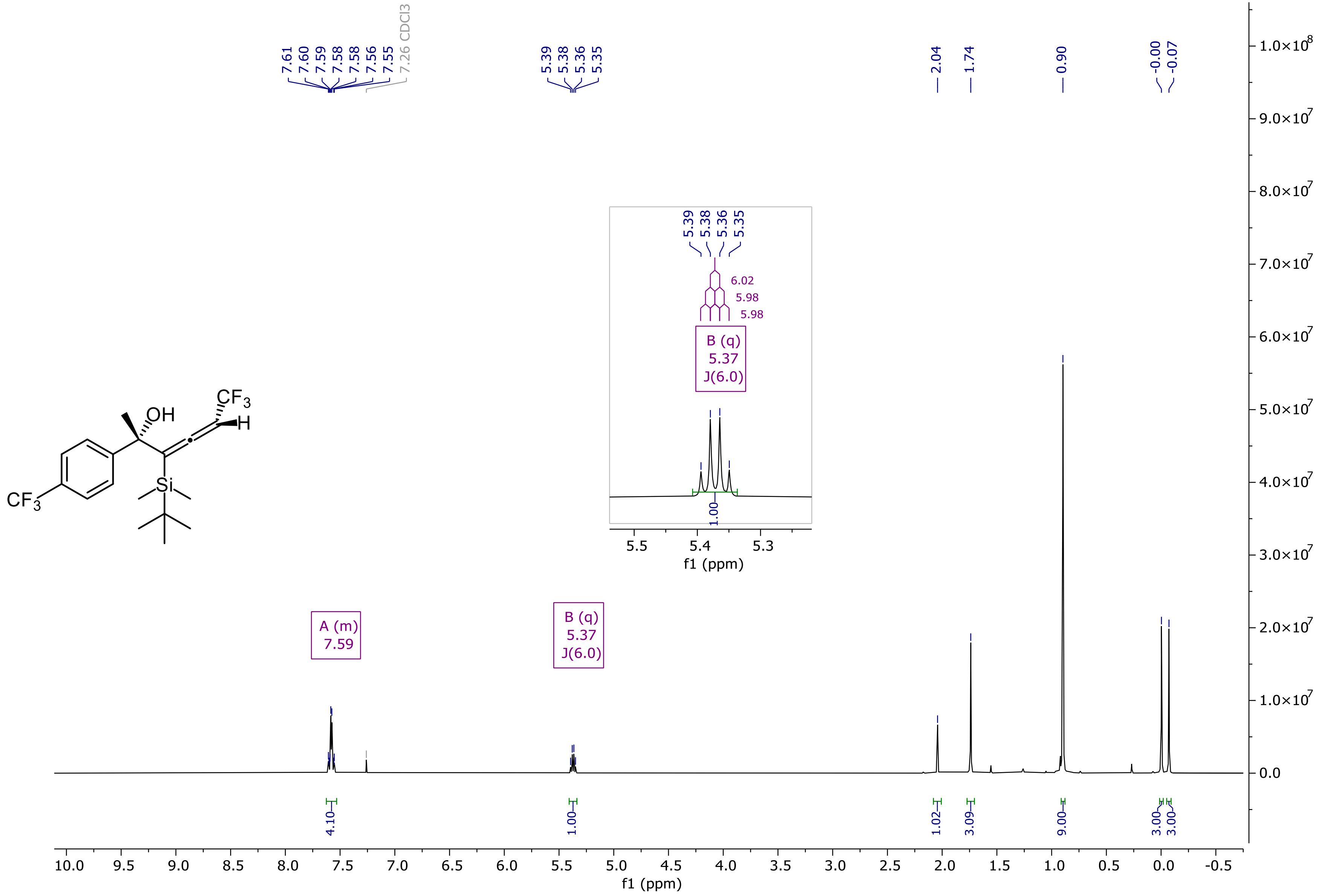

^{13}C NMR (126 MHz, $CDCl_3$) of compound **5i**

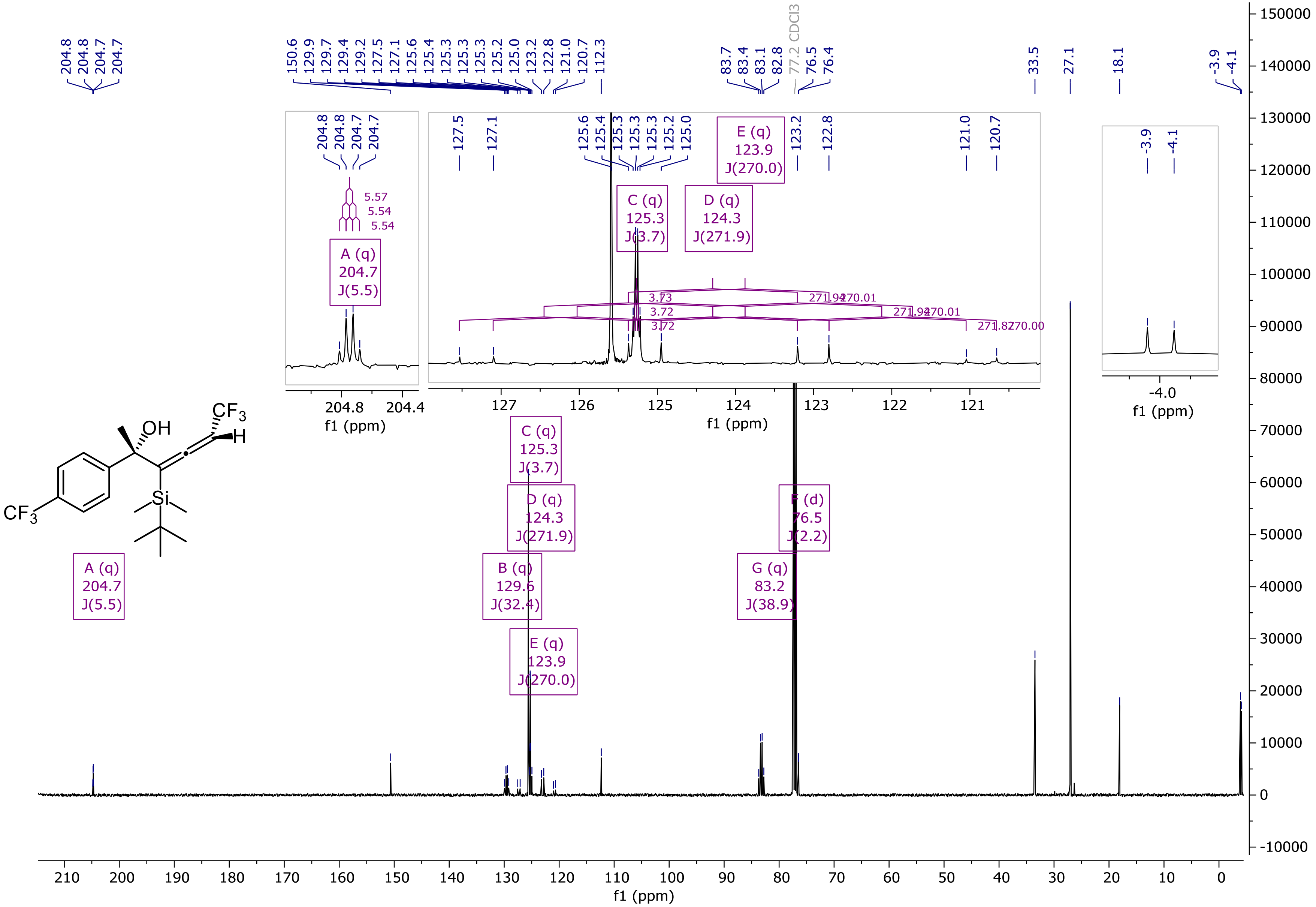

^{19}F NMR (377 MHz, $CDCl_3$) of compound **5i**

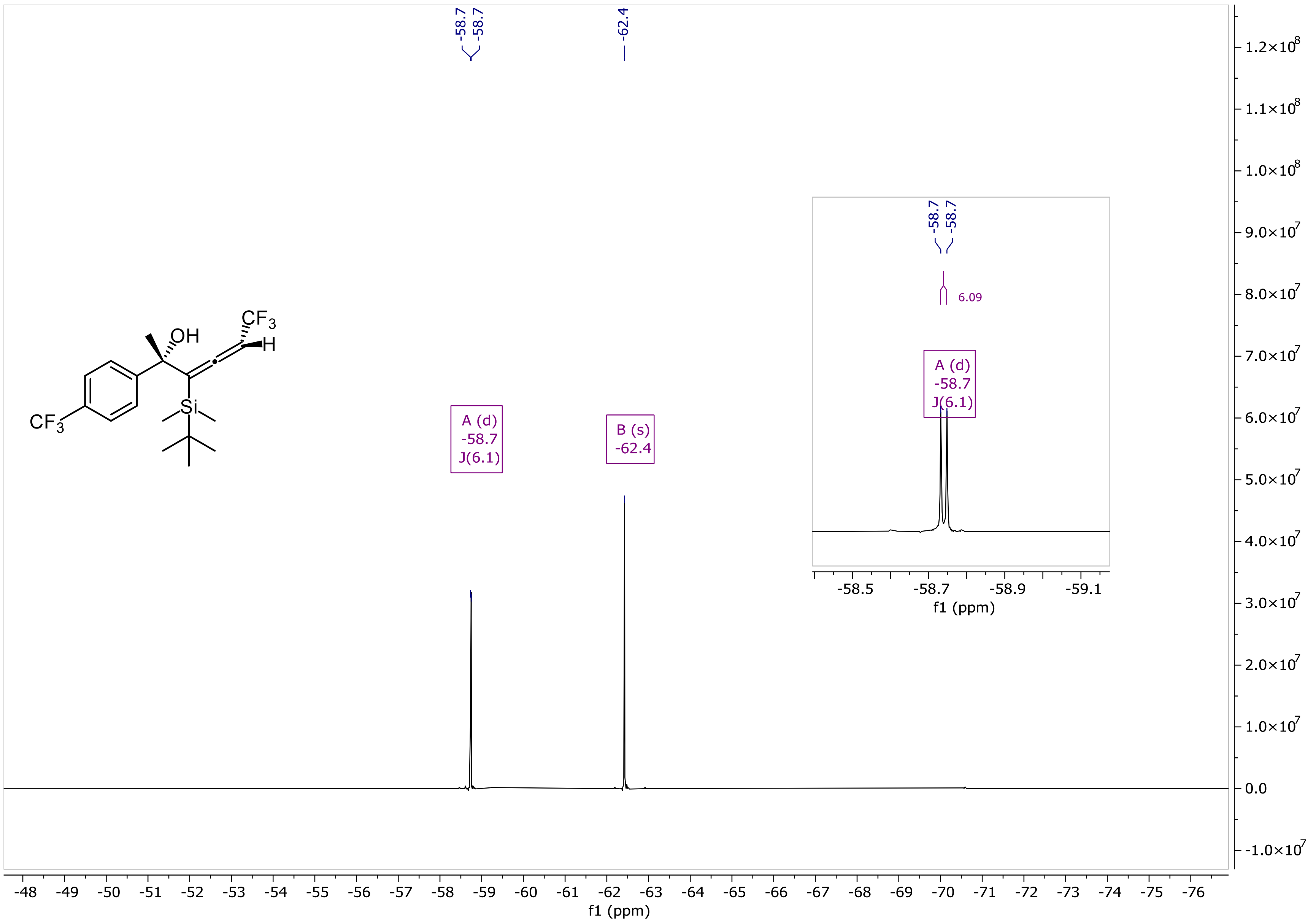

^{29}Si NMR (99 MHz, $CDCl_3$) of compound **5i**

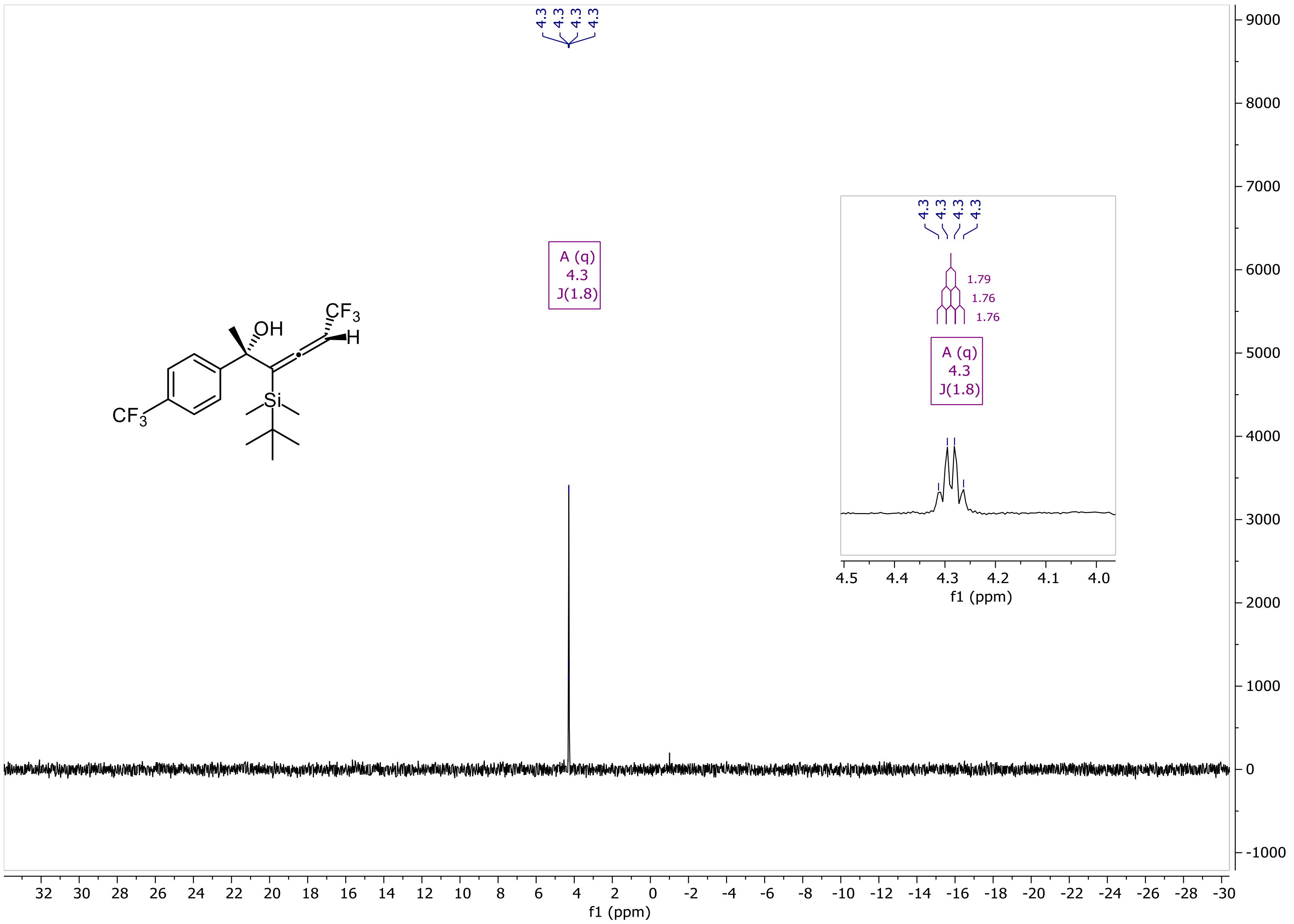

^{1}H NMR (500 MHz, $CDCl_3$) of compound **5j**

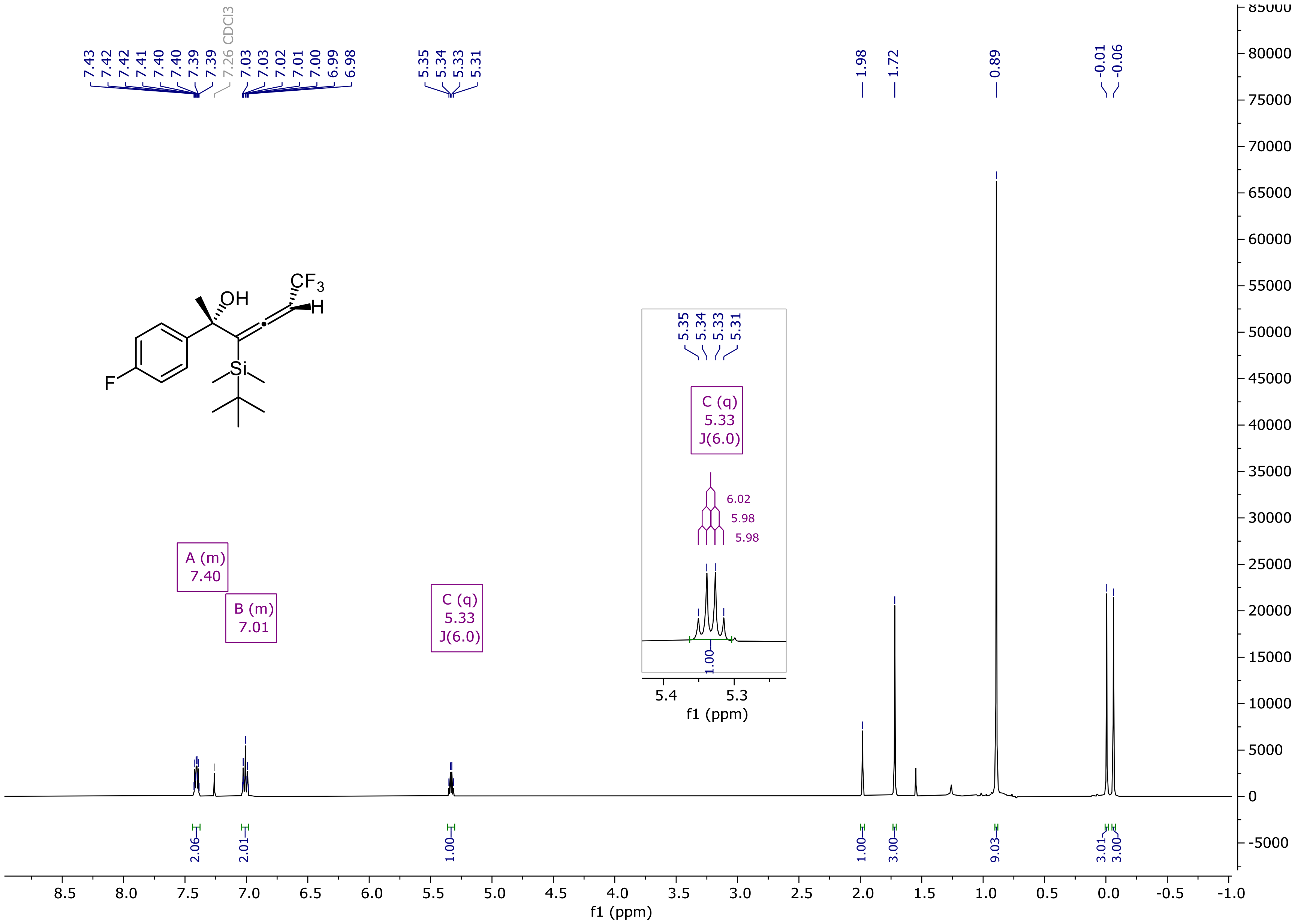

^{13}C NMR (126 MHz, $CDCl_3$) of compound **5j**

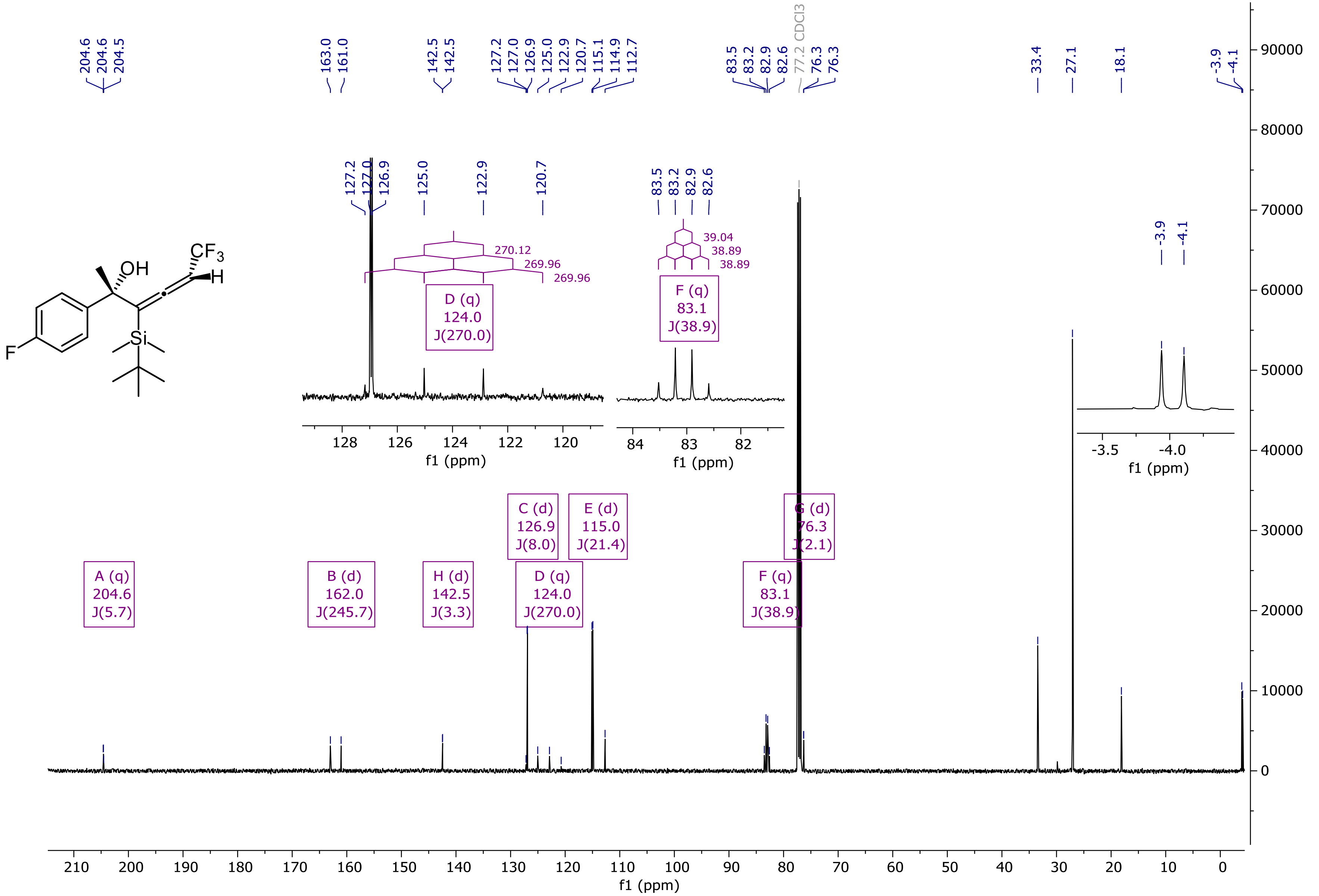

^{19}F NMR (377 MHz, $CDCl_3$) of compound **5j**

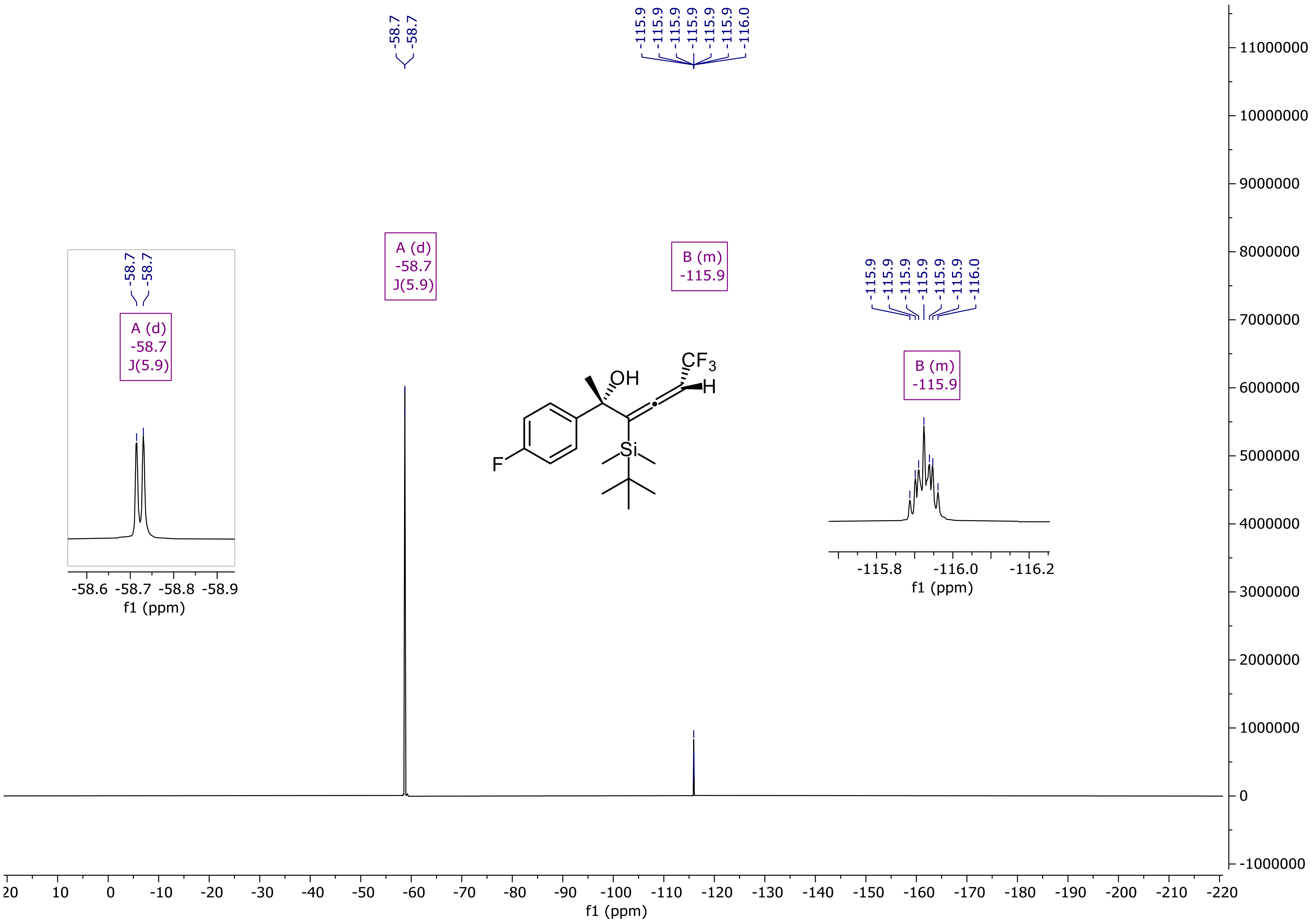

^{29}Si NMR (99 MHz, $CDCl_3$) of compound **5j**

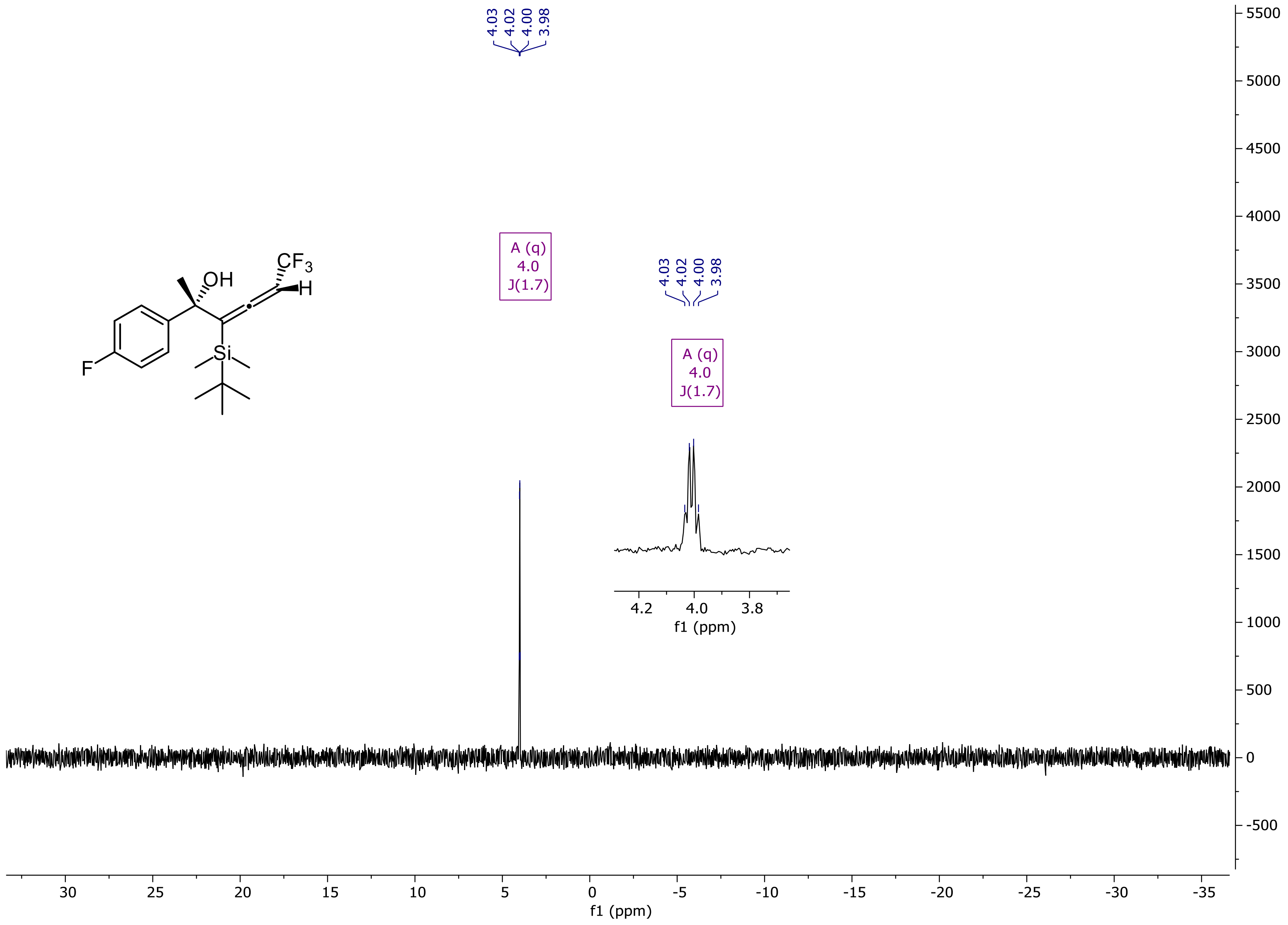

^{1}H NMR (500 MHz, $CDCl_3$) of compound **5k**

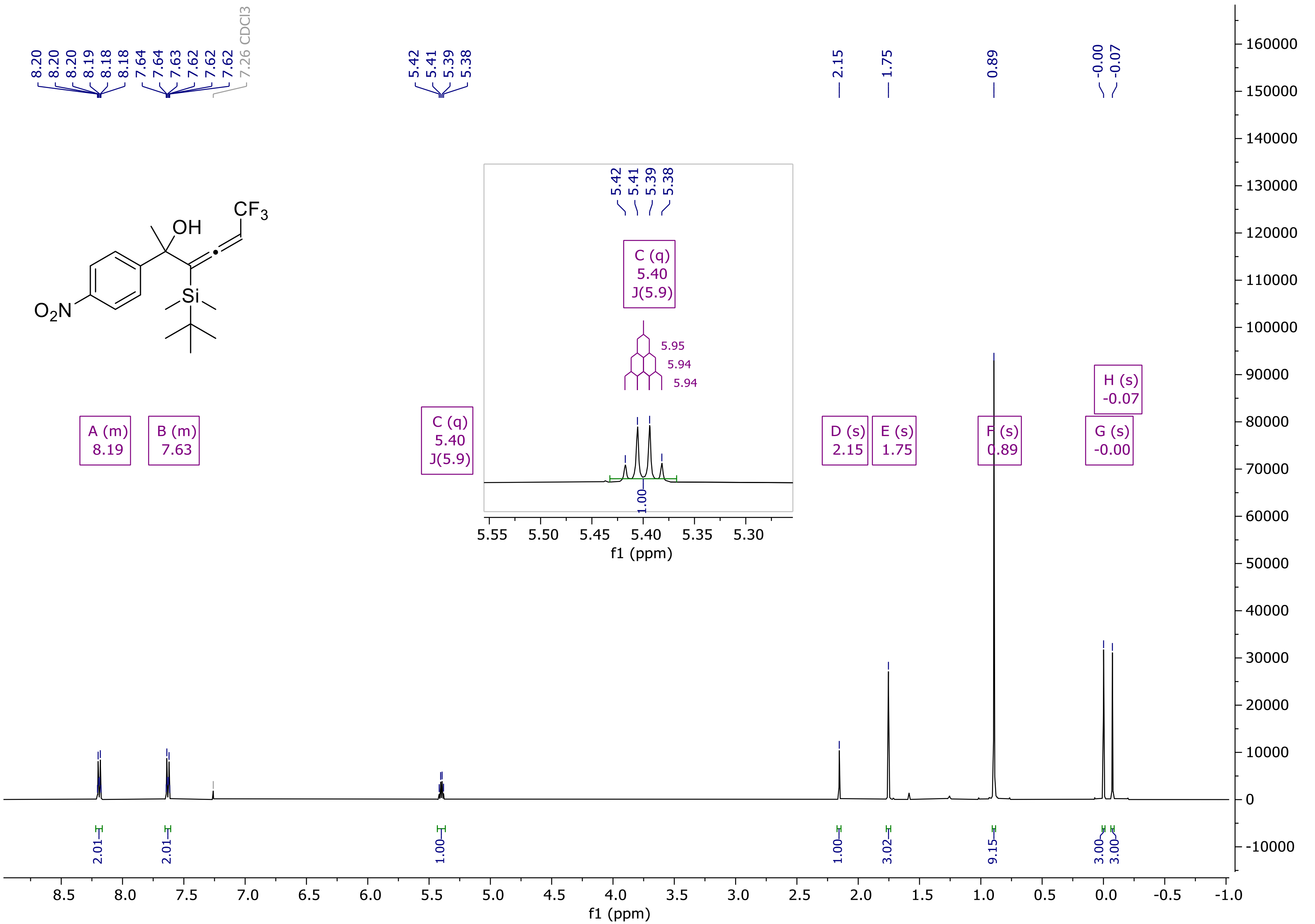

^{13}C NMR (126 MHz, $CDCl_3$) of compound **5k**

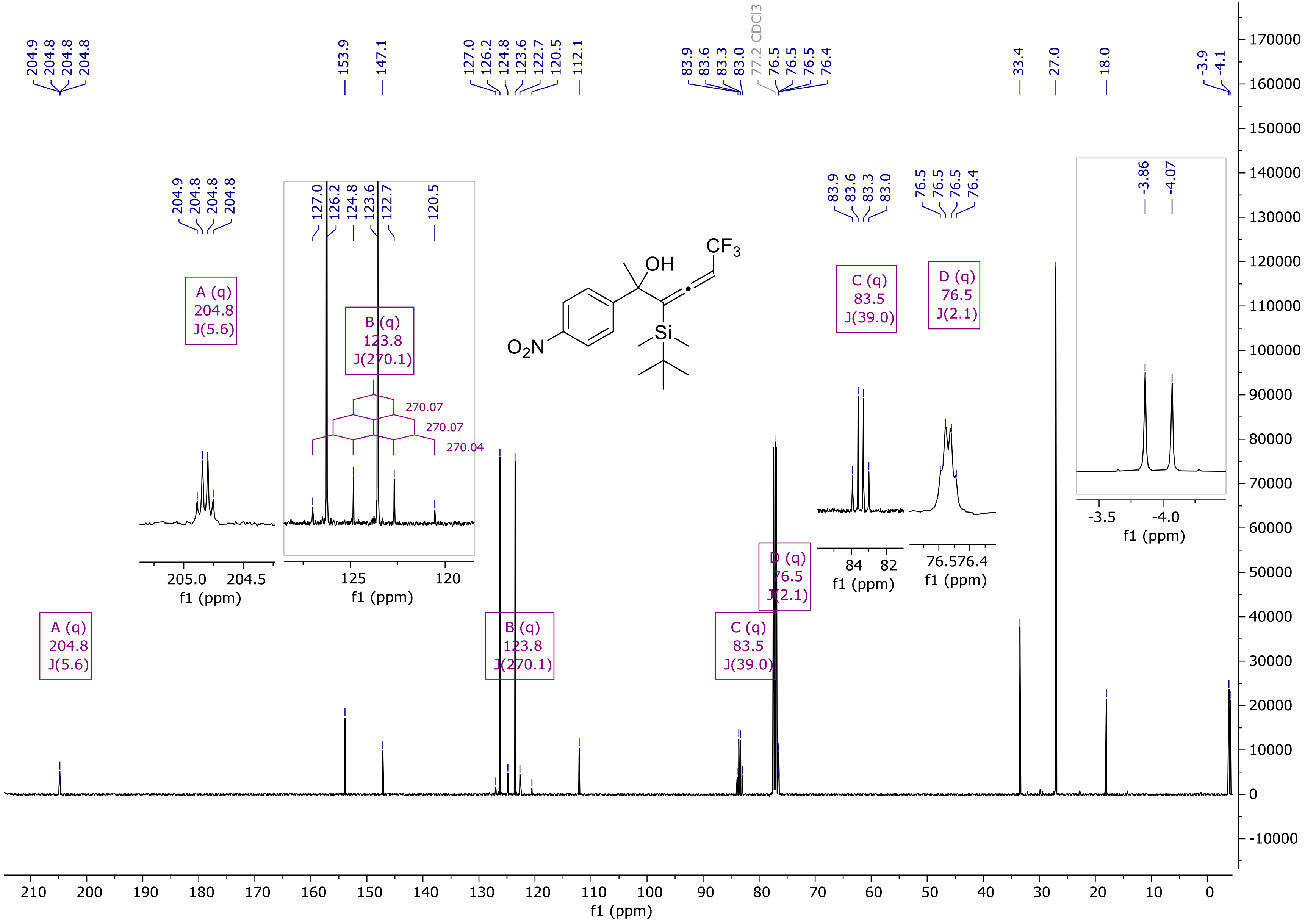

^{19}F NMR (377 MHz, $CDCl_3$) of compound **5k**

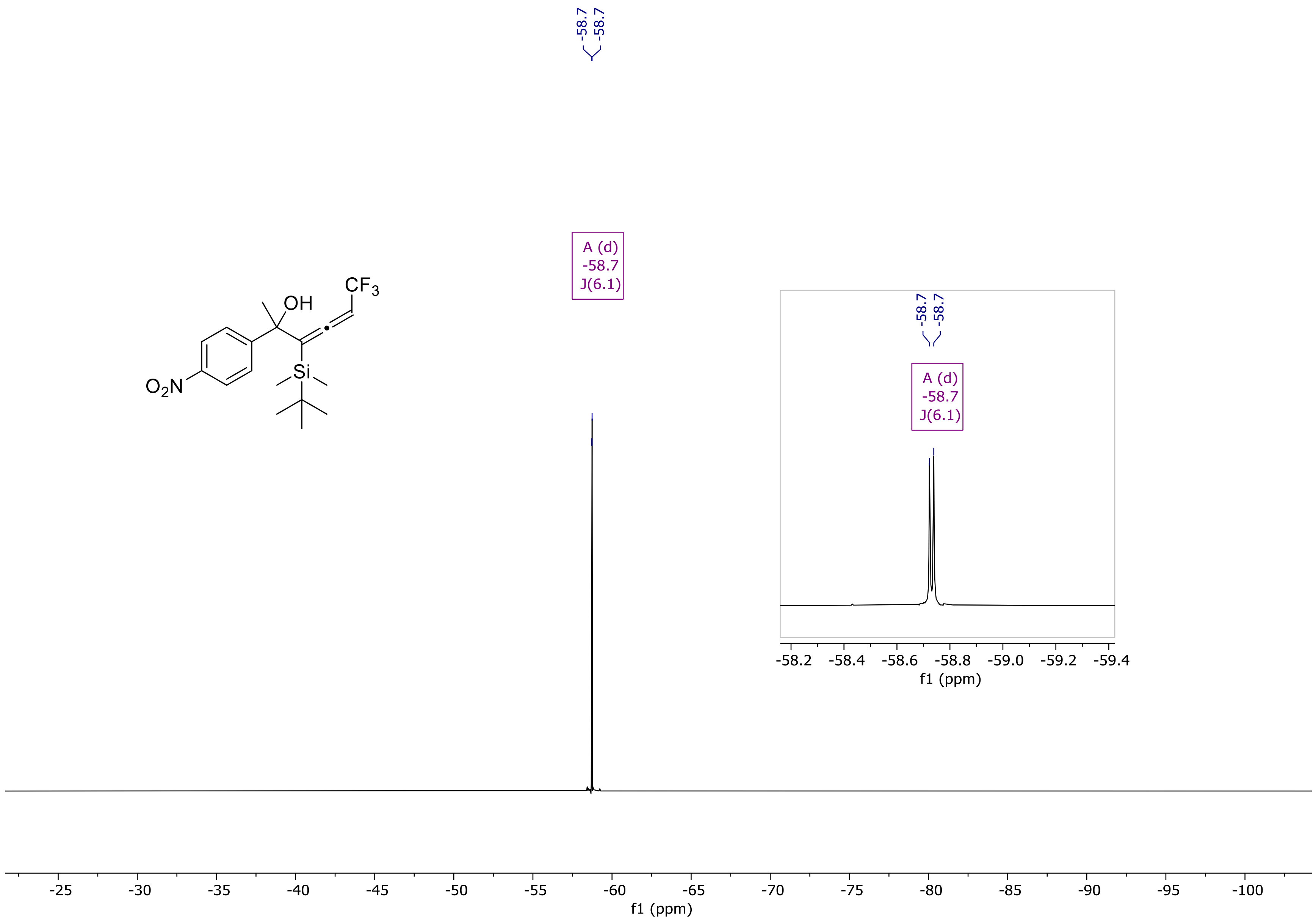

^{29}Si NMR (99 MHz, $CDCl_3$) of compound **5k**

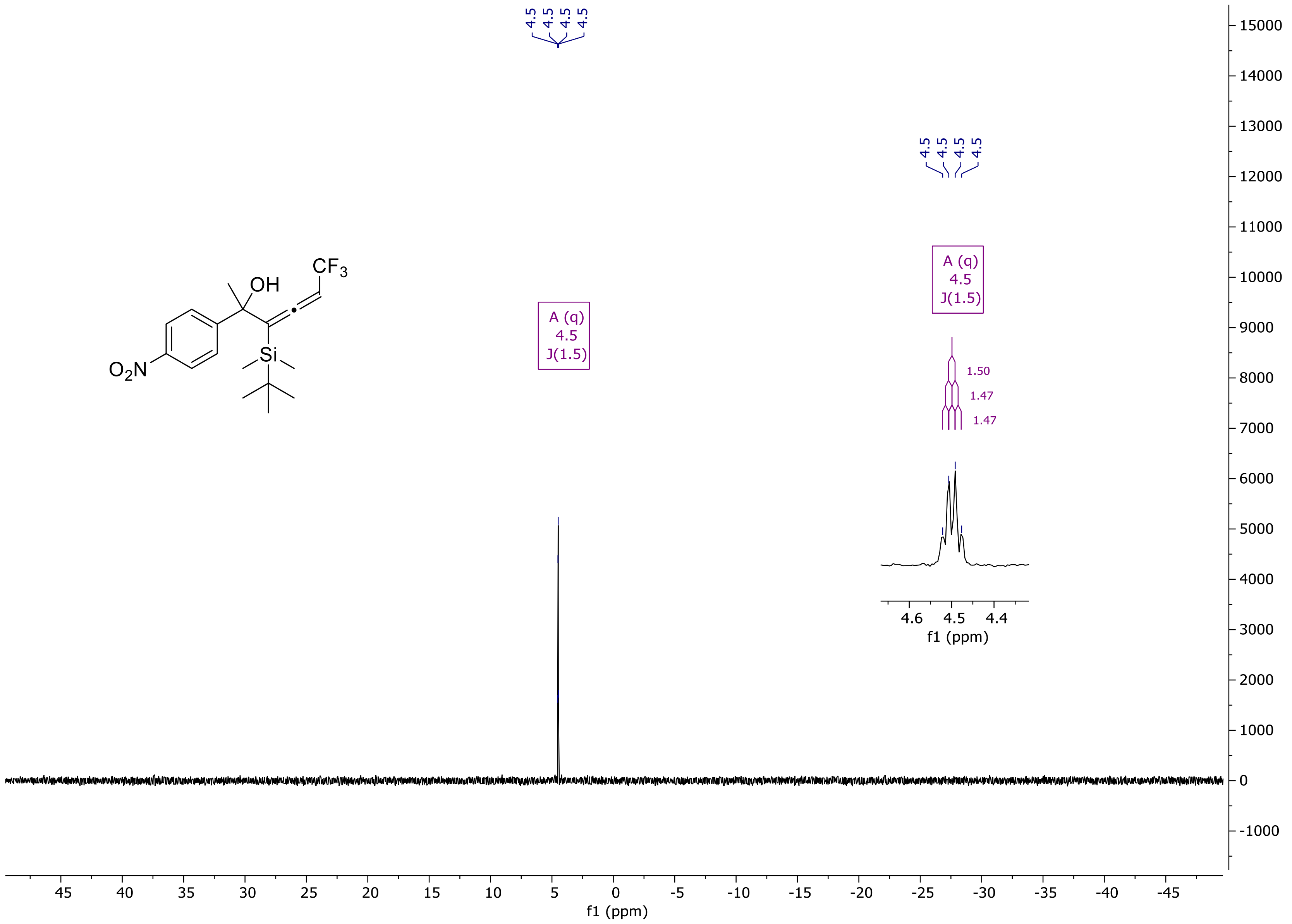

^{1}H NMR (400 MHz, $CDCl_3$) of compound **5l**

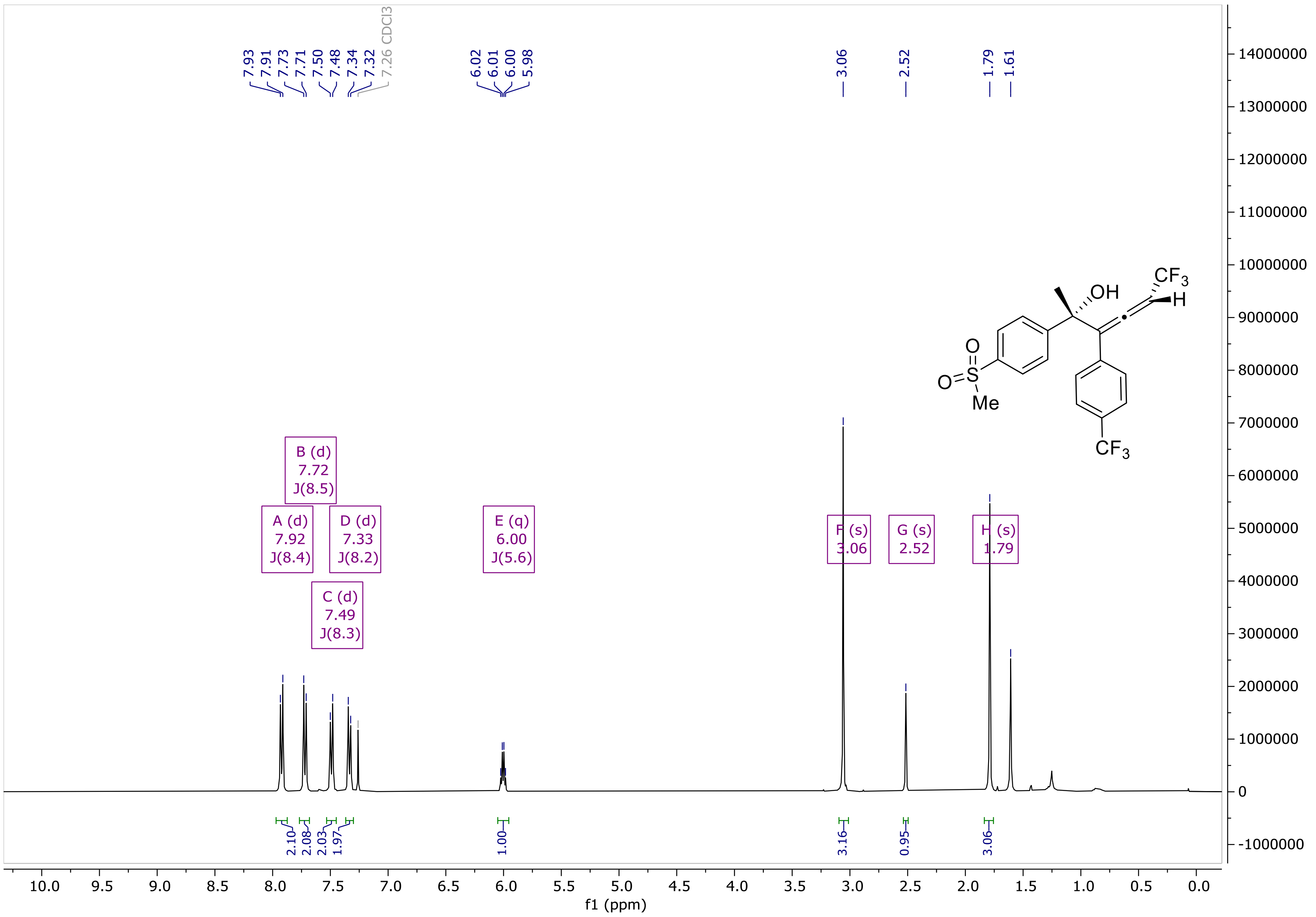

^{13}C NMR (101 MHz, $CDCl_3$) of compound **5l**

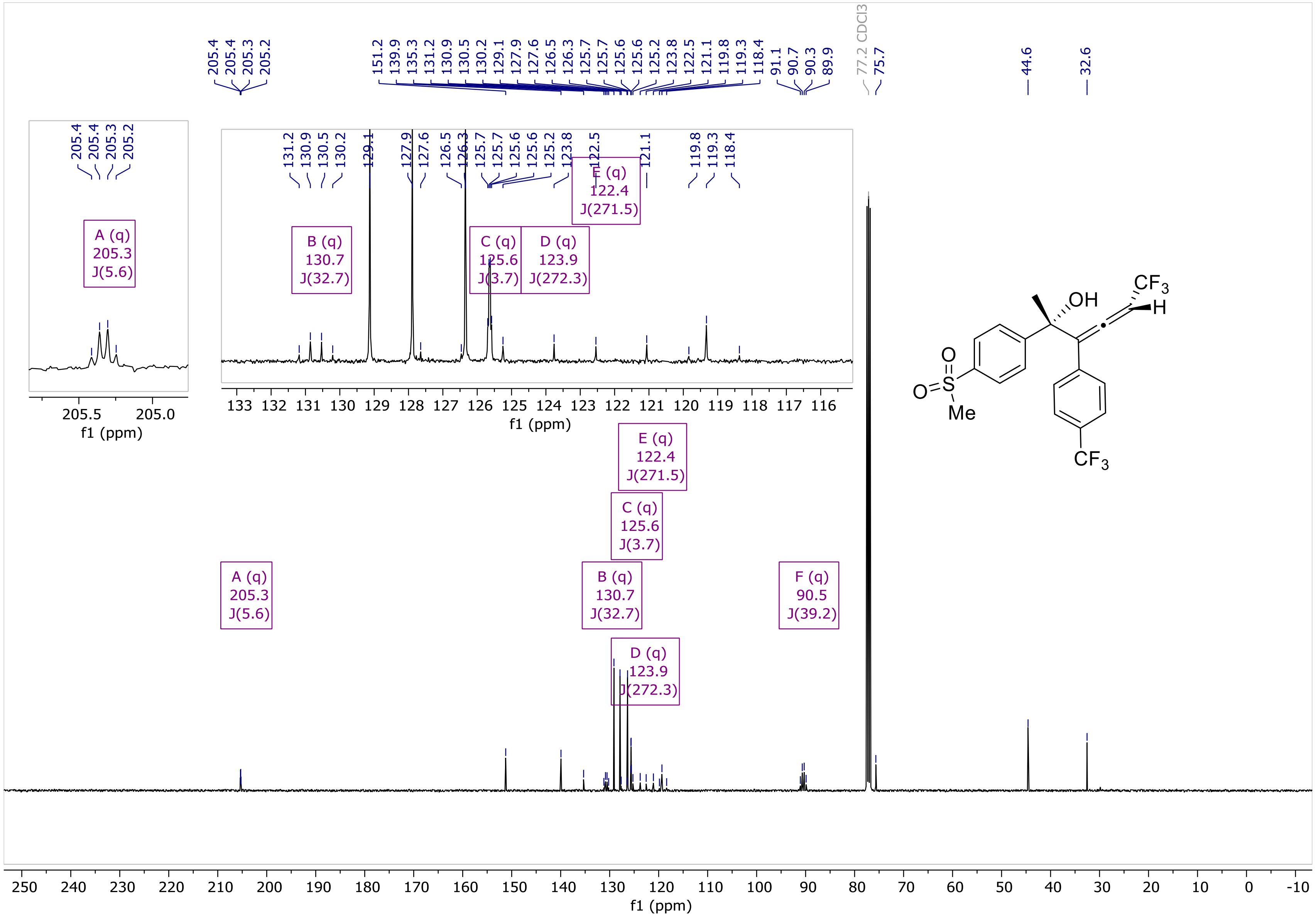

^{19}F NMR (377 MHz, $CDCl_3$) of compound **5l**

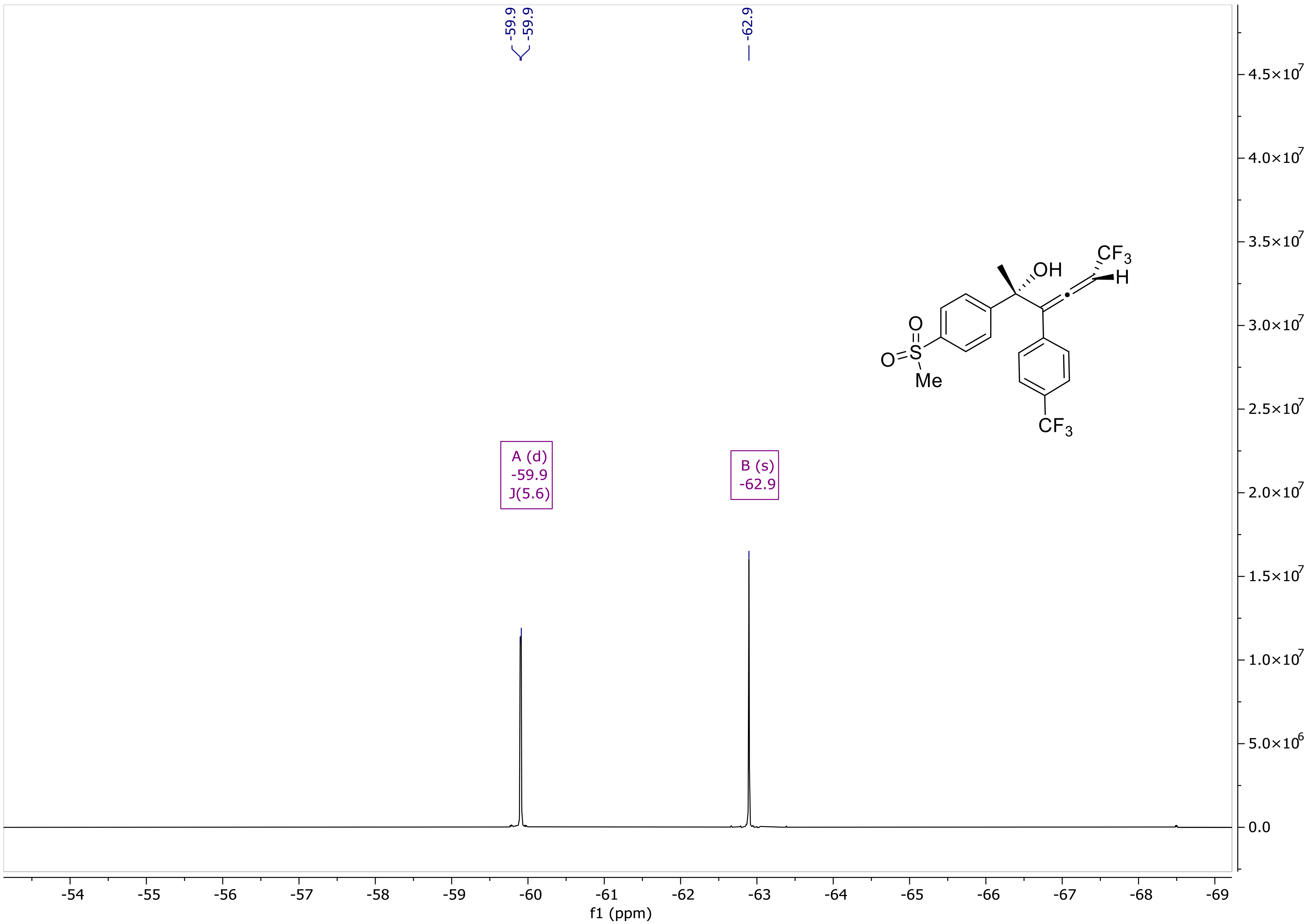

^{1}H NMR (500 MHz, $CDCl_3$) of compound **5m**

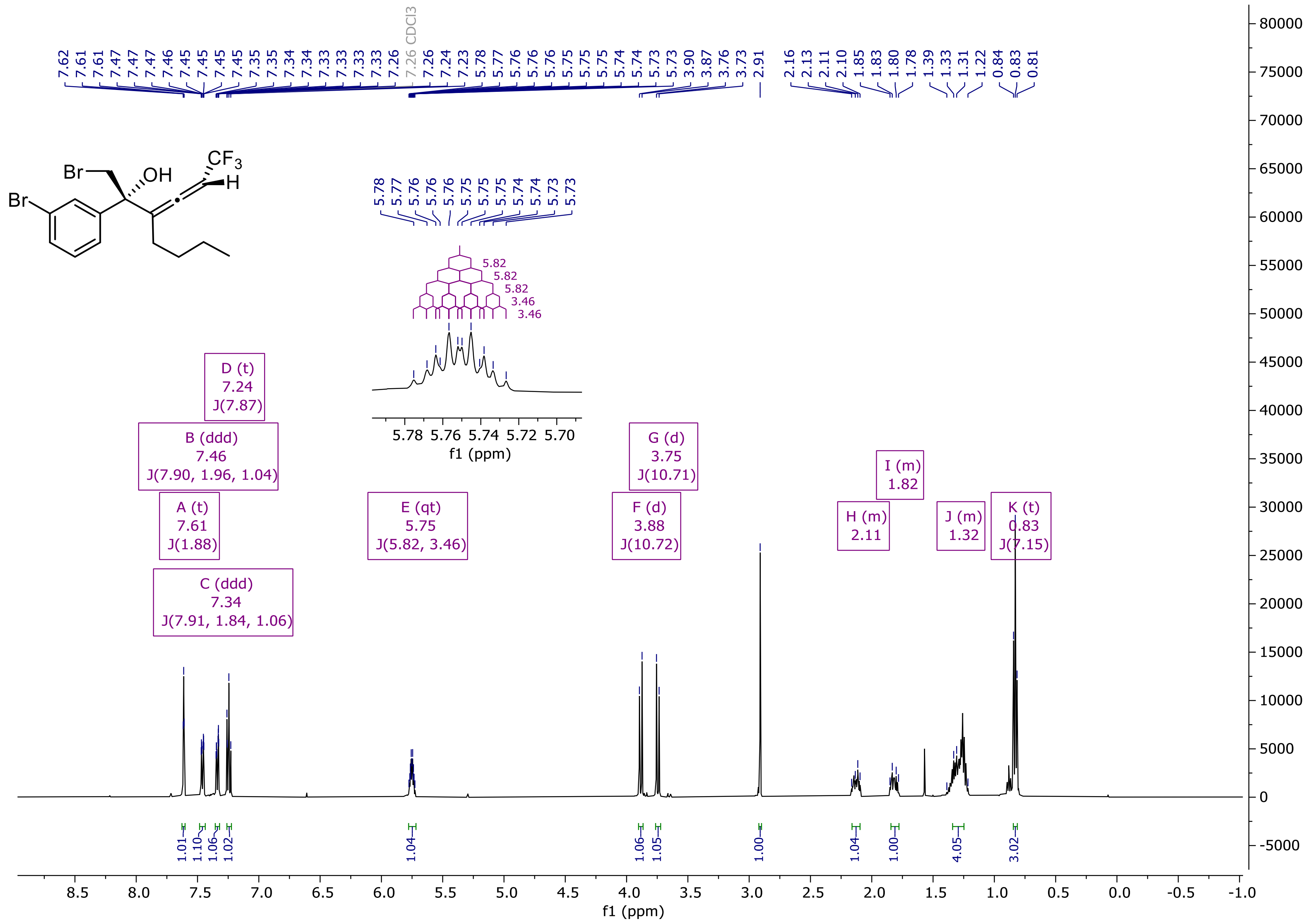

^{13}C NMR (126 MHz, $CDCl_3$) of compound **5m**

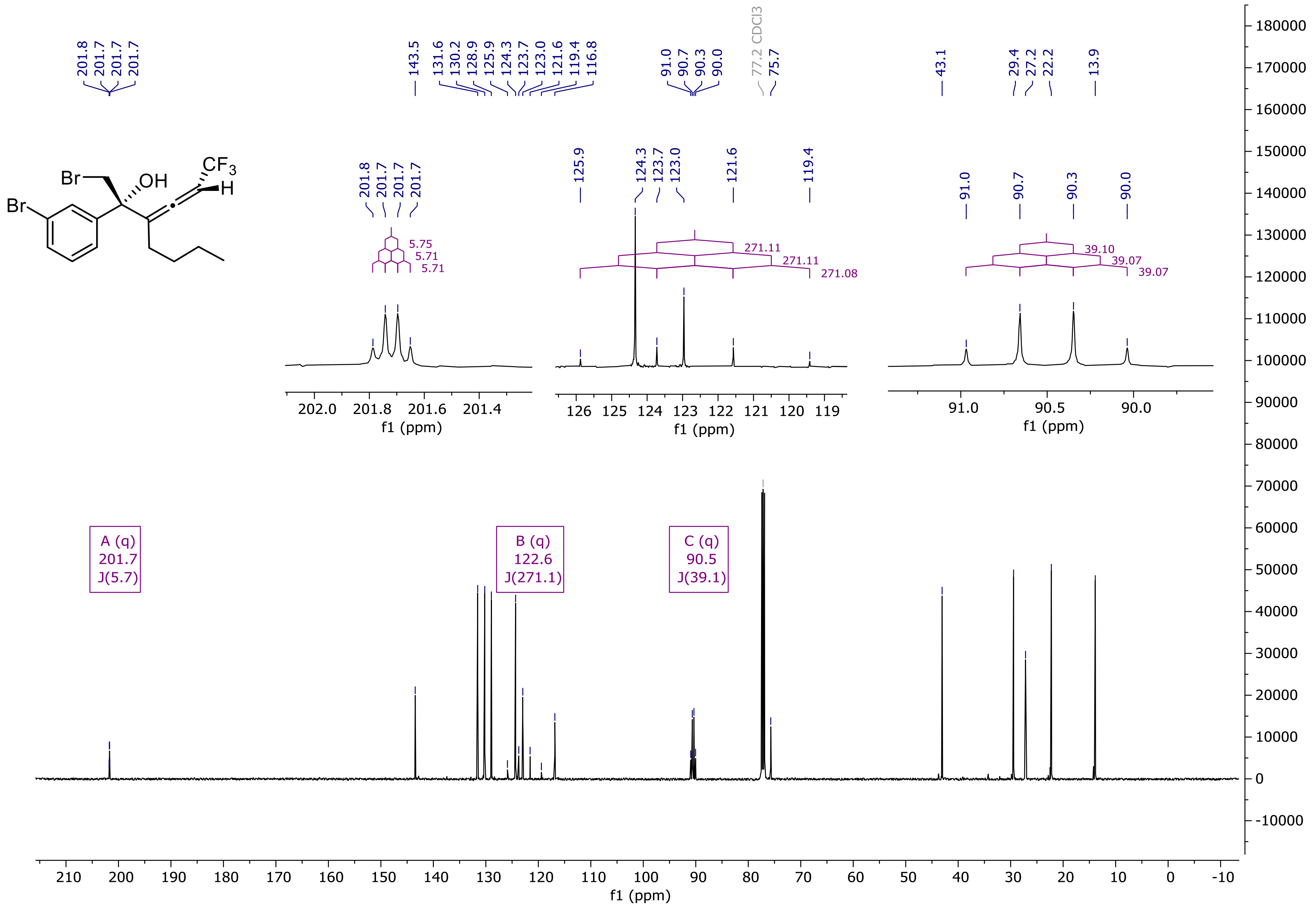

^{19}F NMR (377 MHz, $CDCl_3$) of compound **5m**

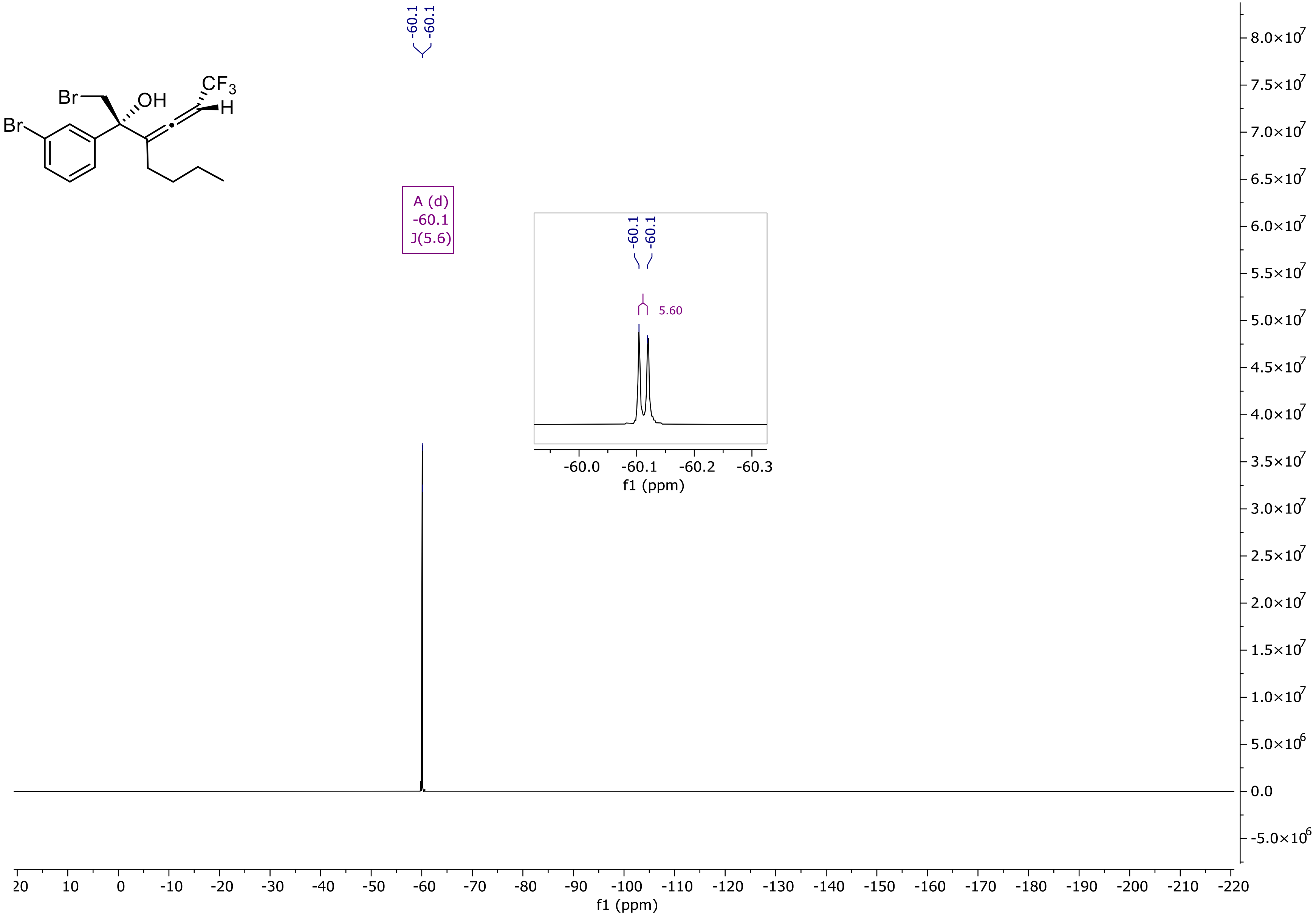

^{1}H NMR (400 MHz, $CDCl_3$) of compound **5n**

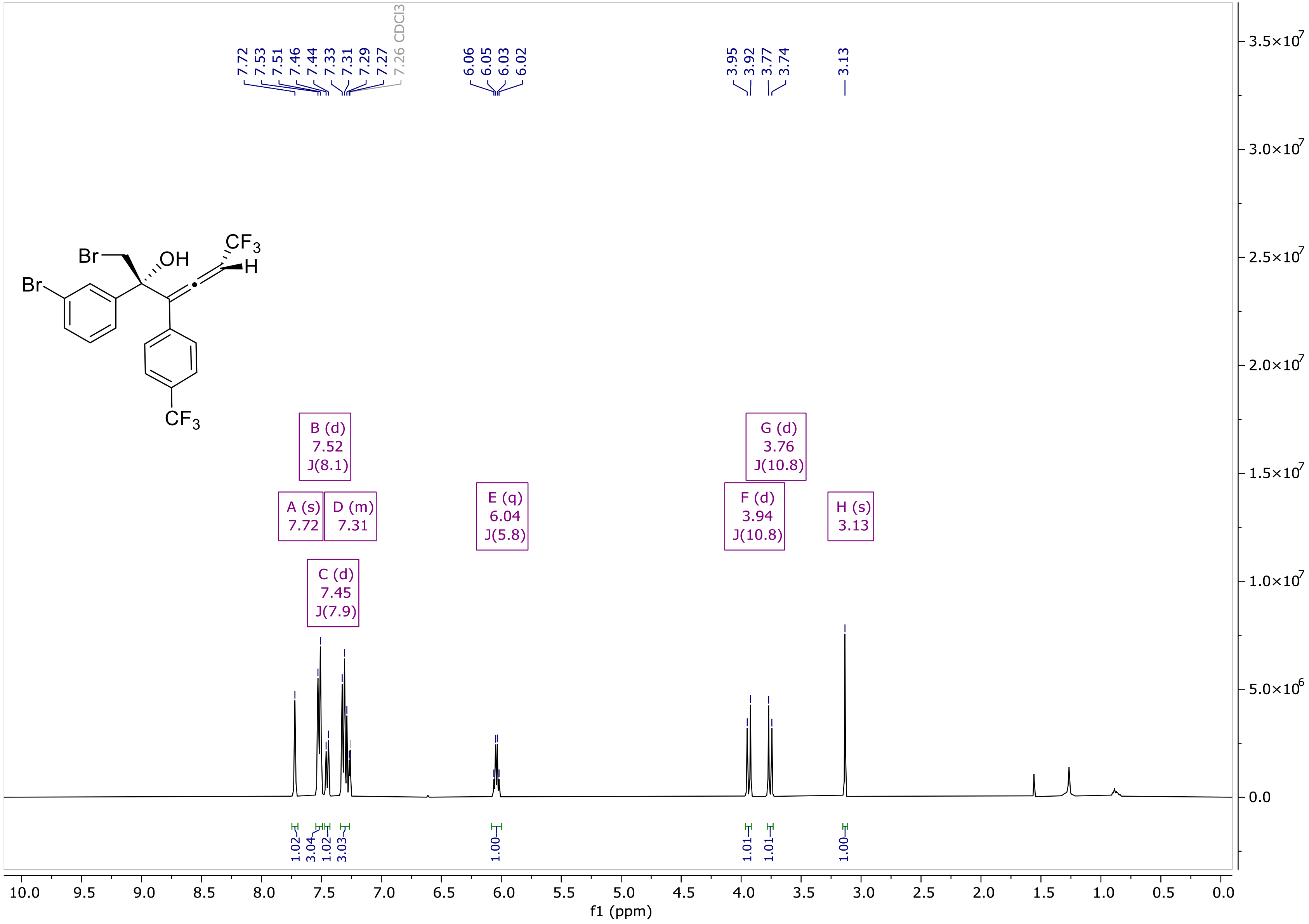

^{13}C NMR (101 MHz, $CDCl_3$) of compound **5n**

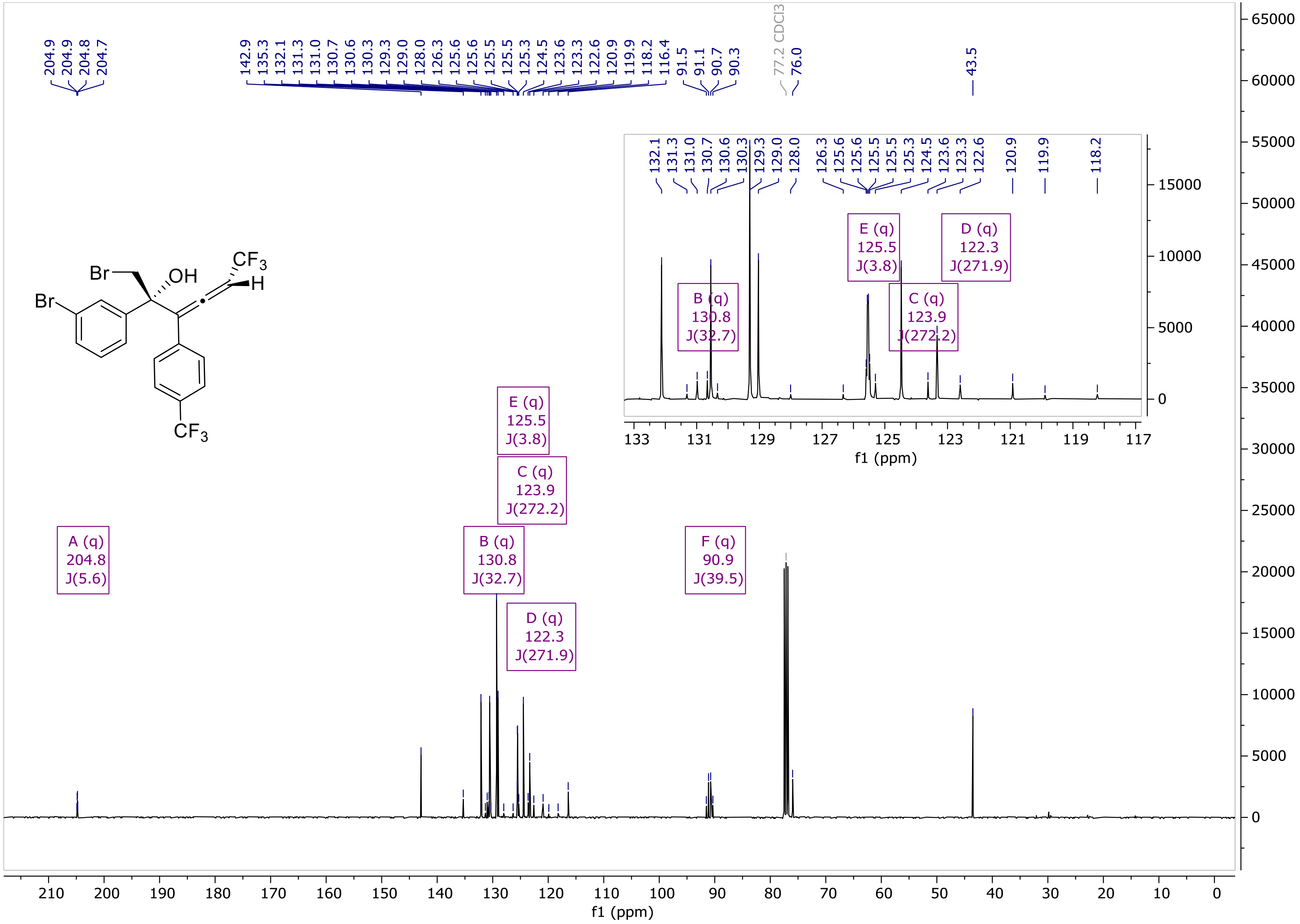

^{19}F NMR (377 MHz, $CDCl_3$) of compound **5n**

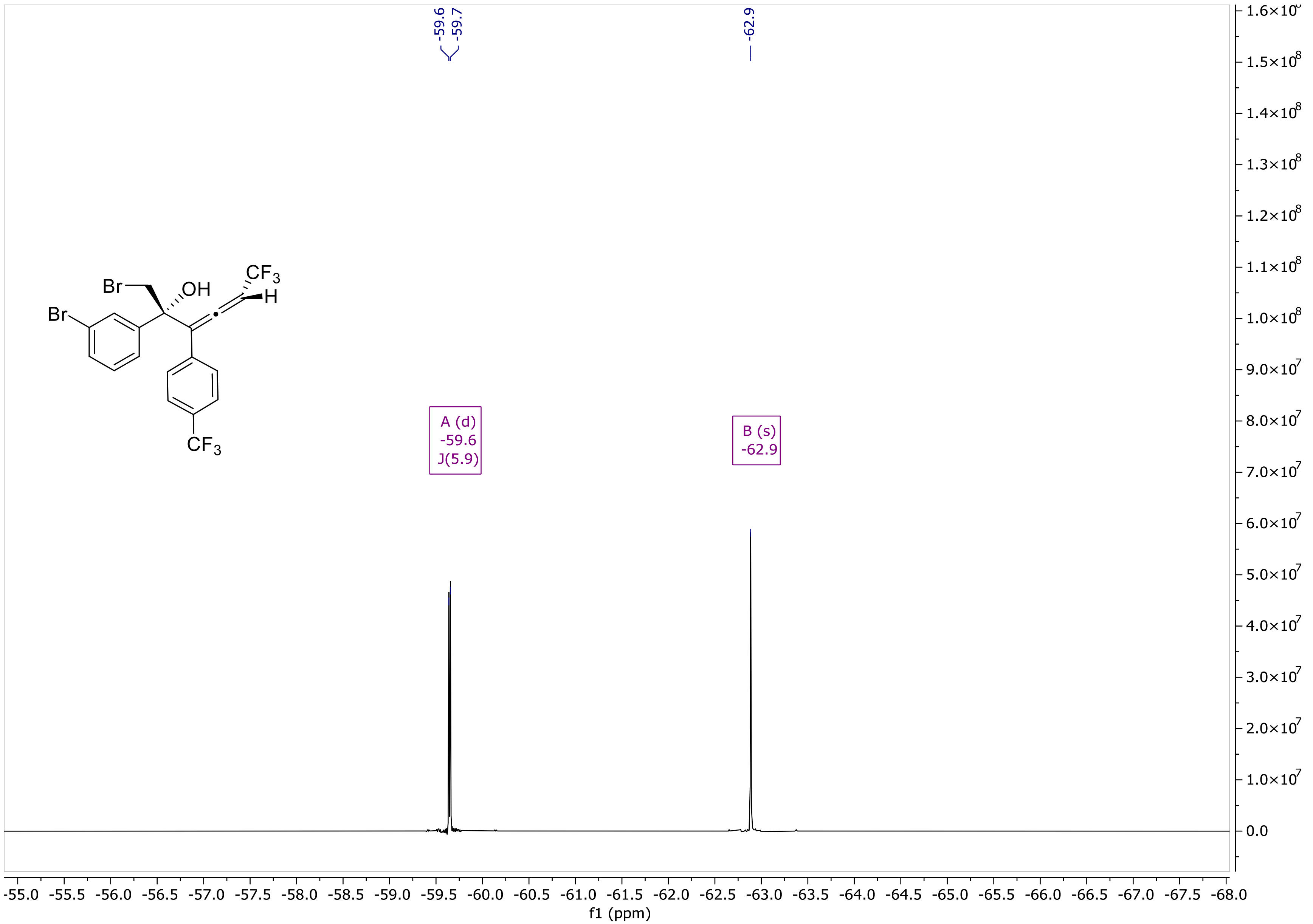

^{1}H NMR (400 MHz, $CDCl_3$) of compound **5o**

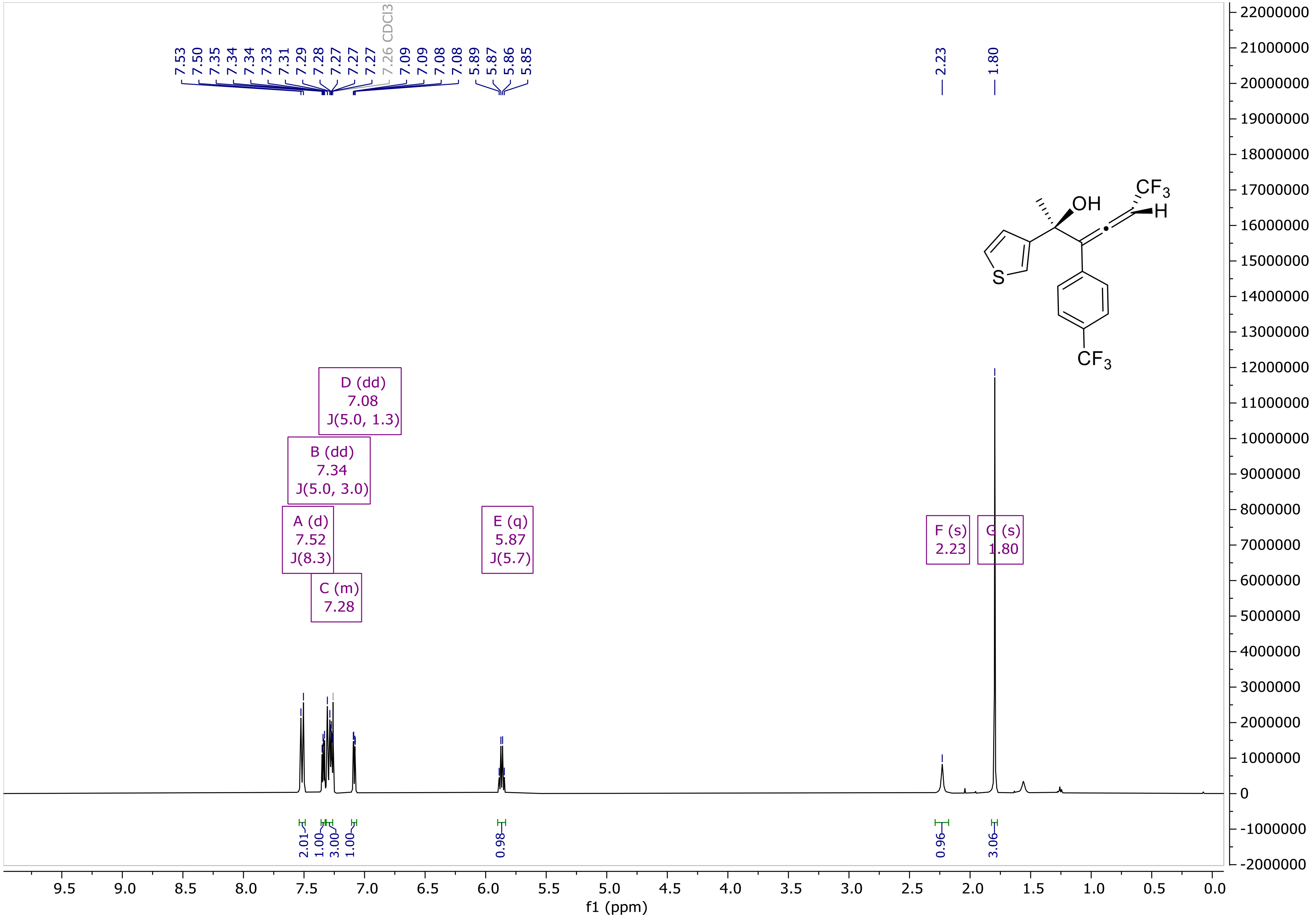

^{13}C NMR (101 MHz, $CDCl_3$) of compound **5o**

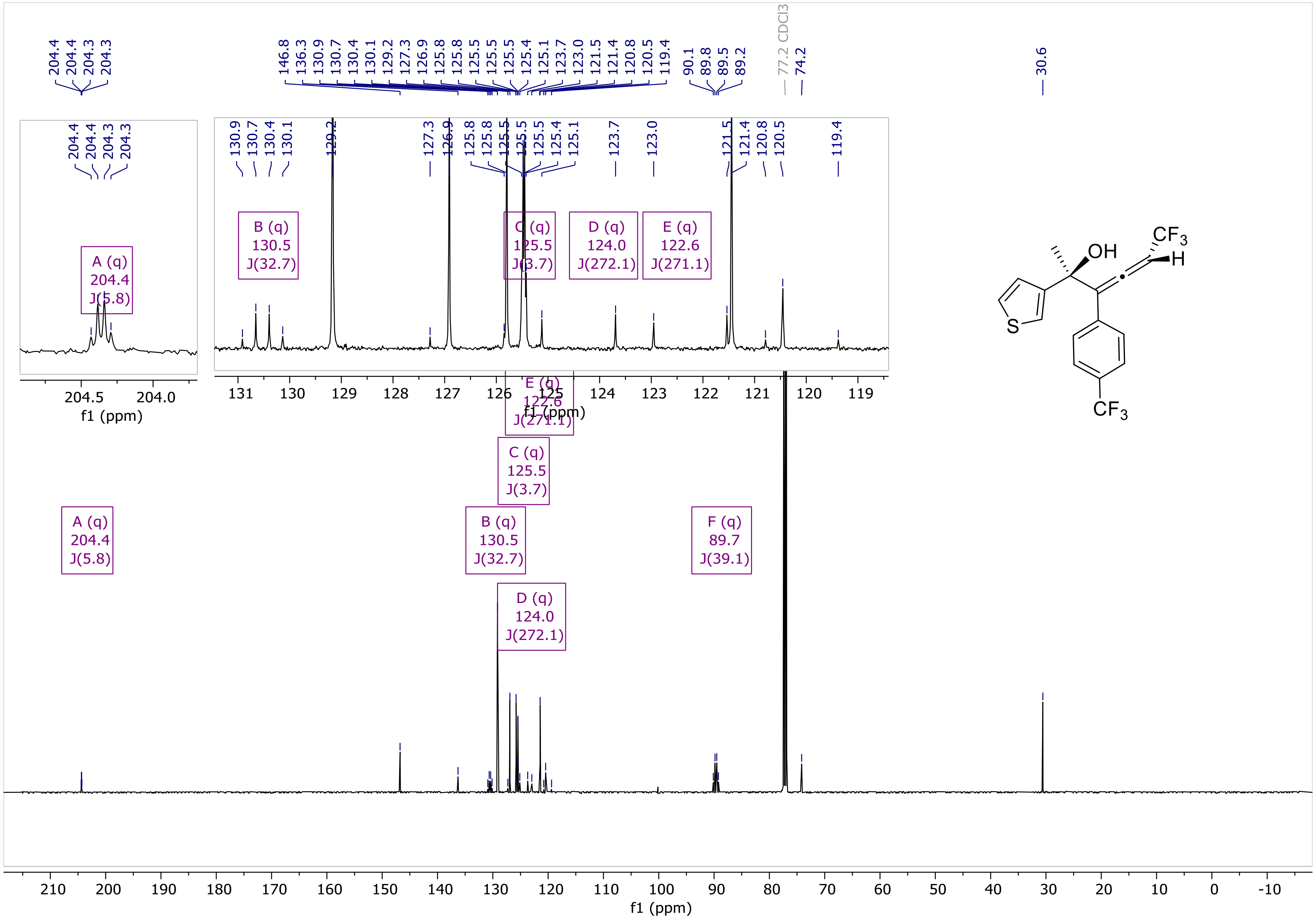

^{19}F NMR (377 MHz, $CDCl_3$) of compound **5o**

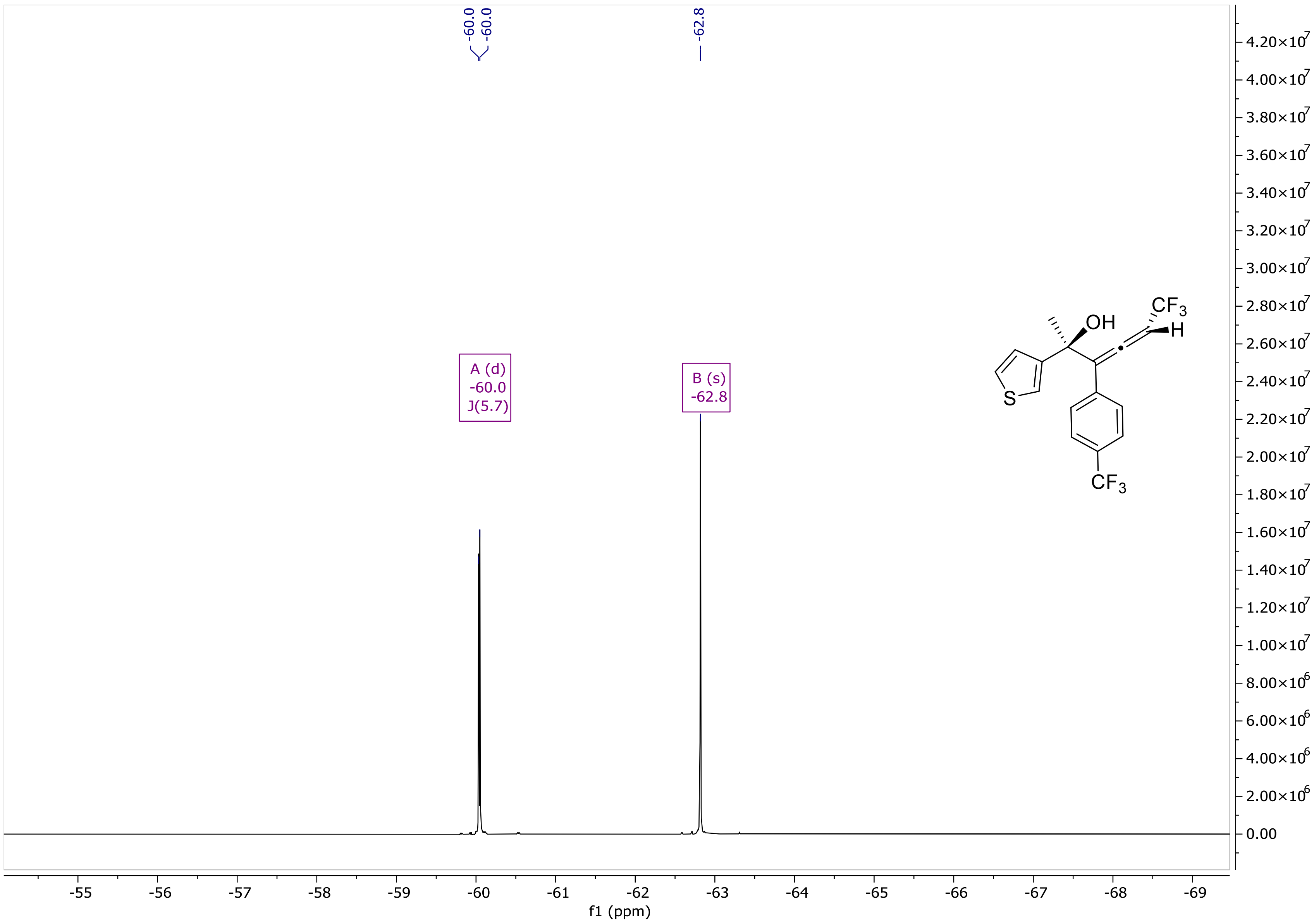

^{1}H NMR (400 MHz, $CDCl_3$) of compound **5p**

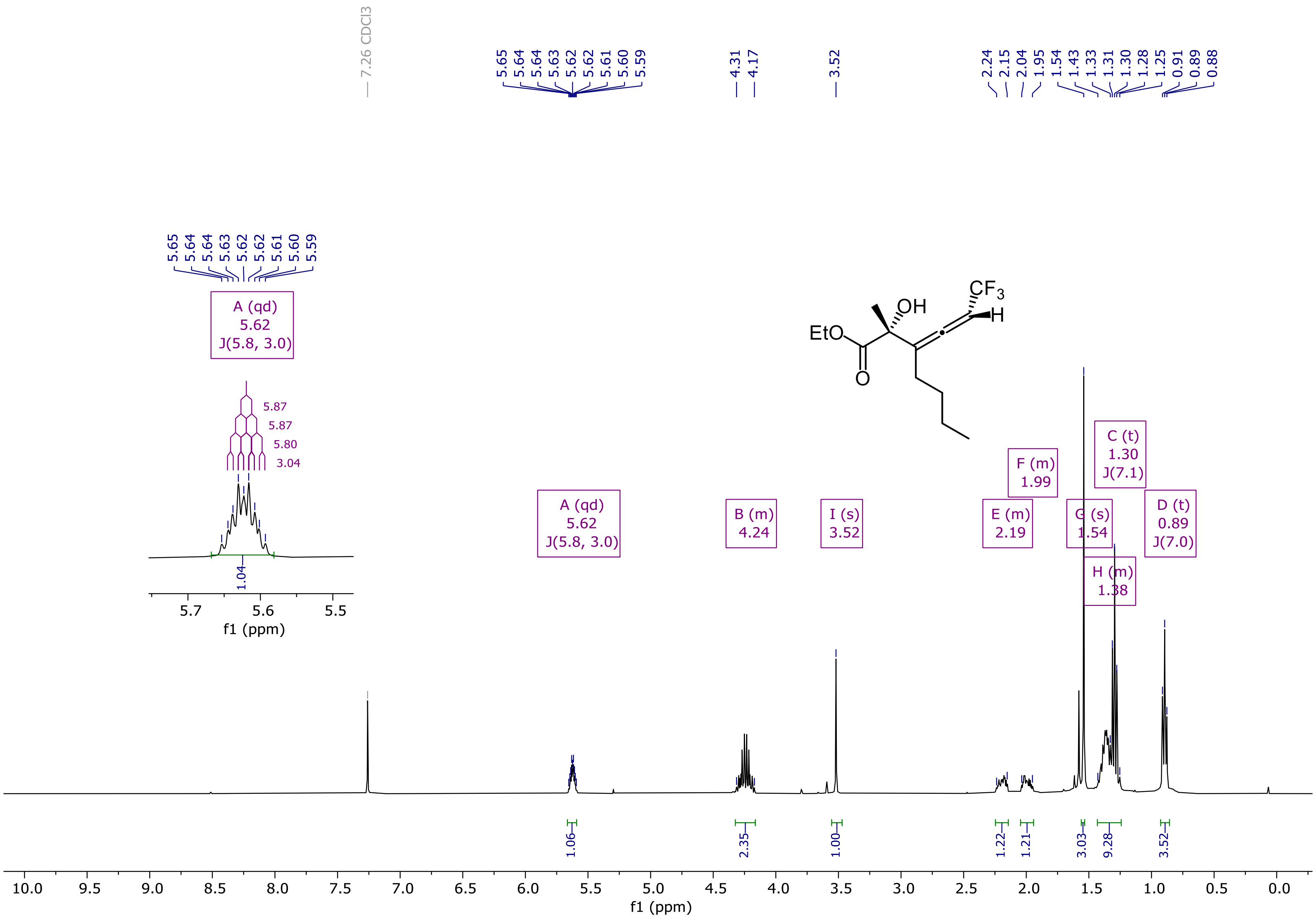

^{13}C NMR (101 MHz, $CDCl_3$) of compound **5p**

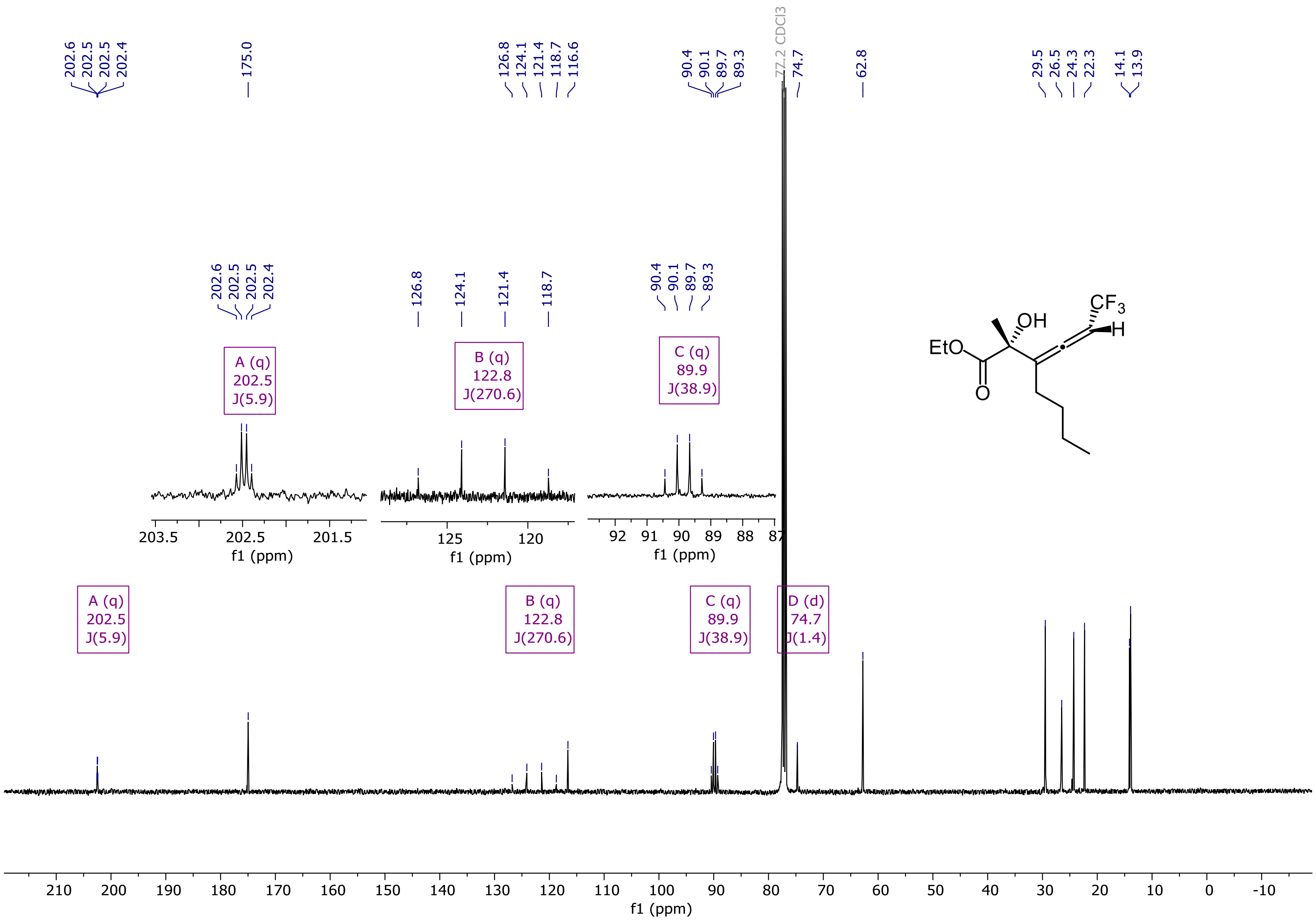

^{19}F NMR (377 MHz, $CDCl_3$) of compound **5p**

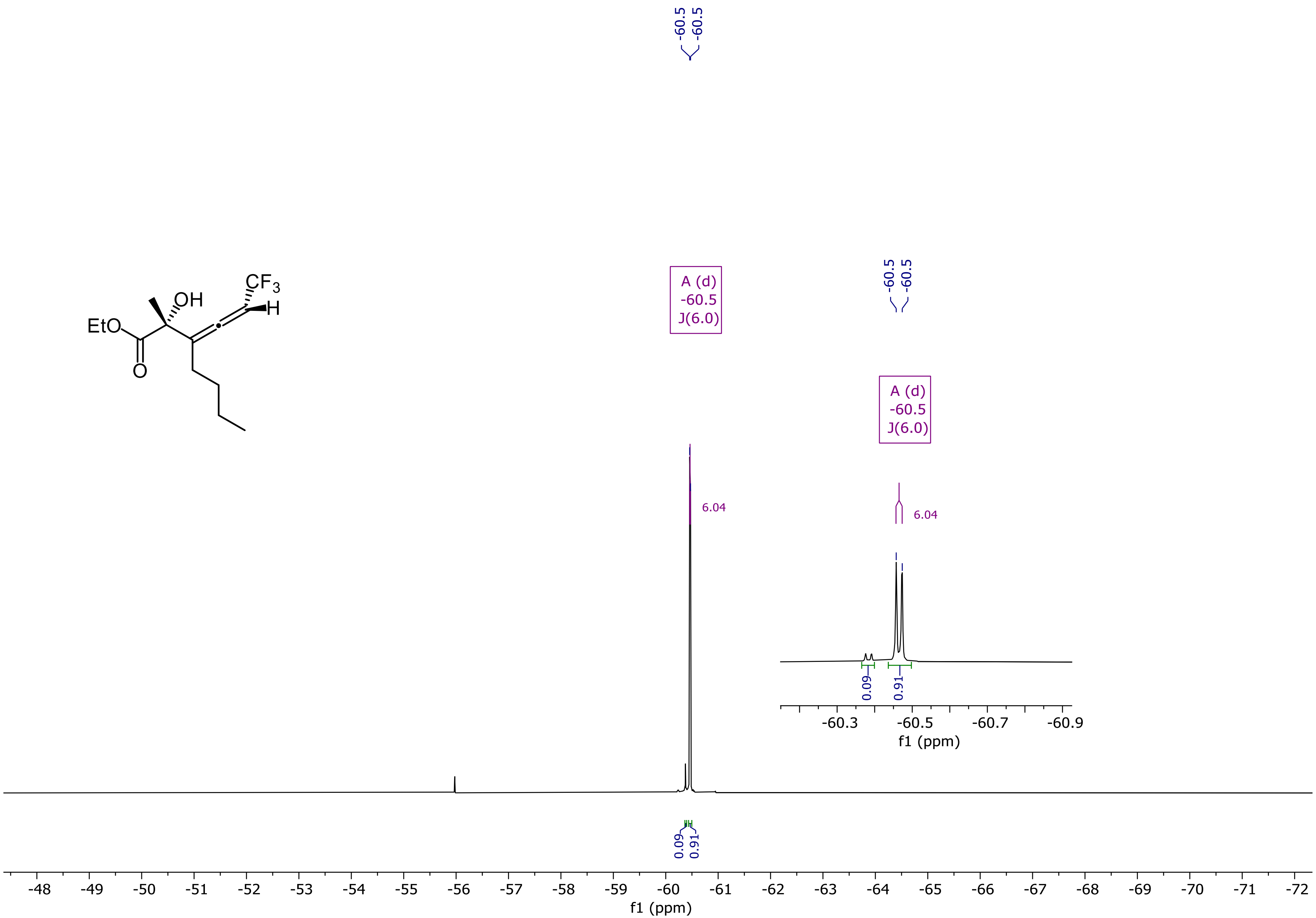

^{1}H NMR (400 MHz, $CDCl_3$) of **5q**

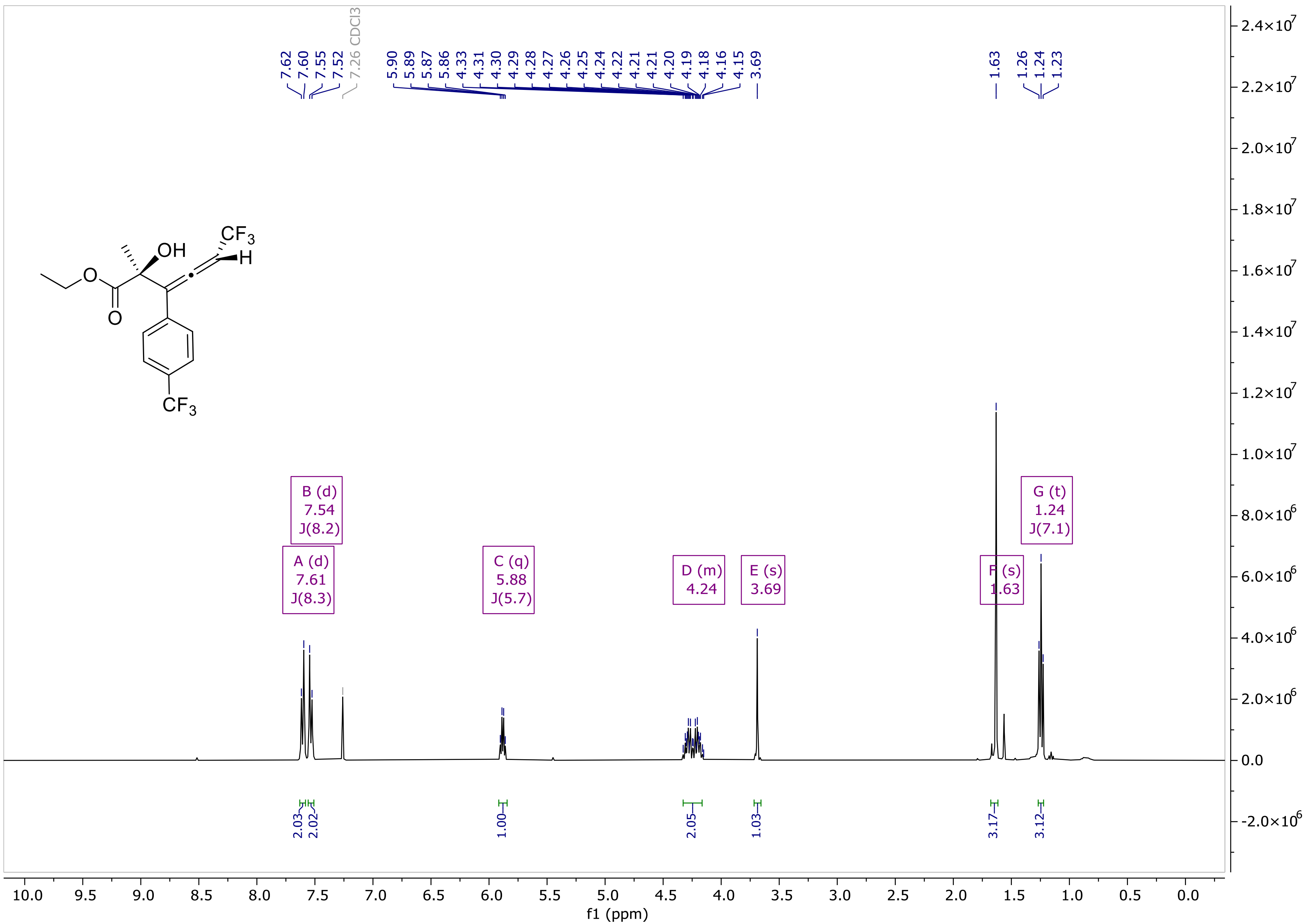

^{13}C NMR (101 MHz, $CDCl_3$) of compound **5q**

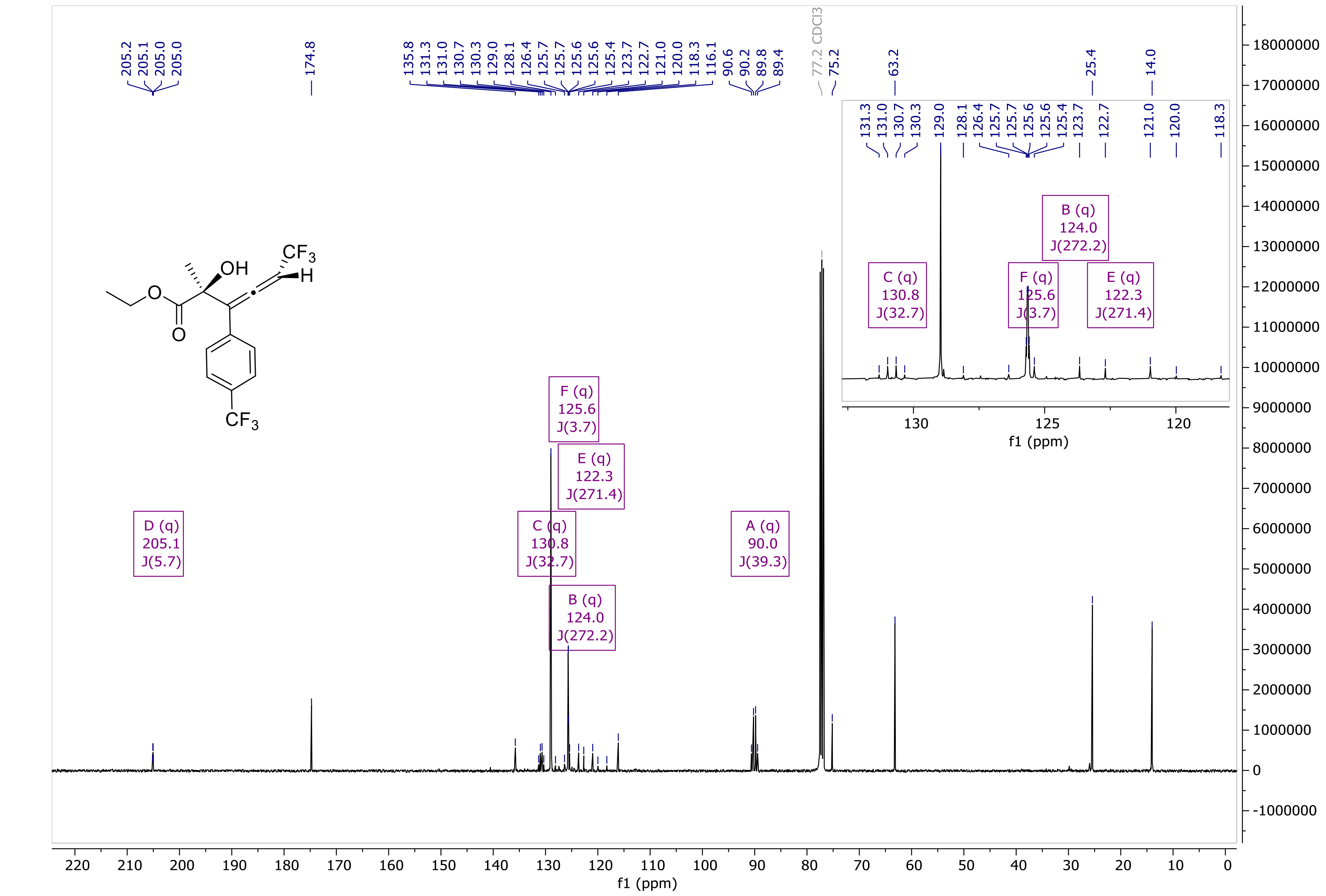

^{19}F NMR (377 MHz, $CDCl_3$) of compound **5q**

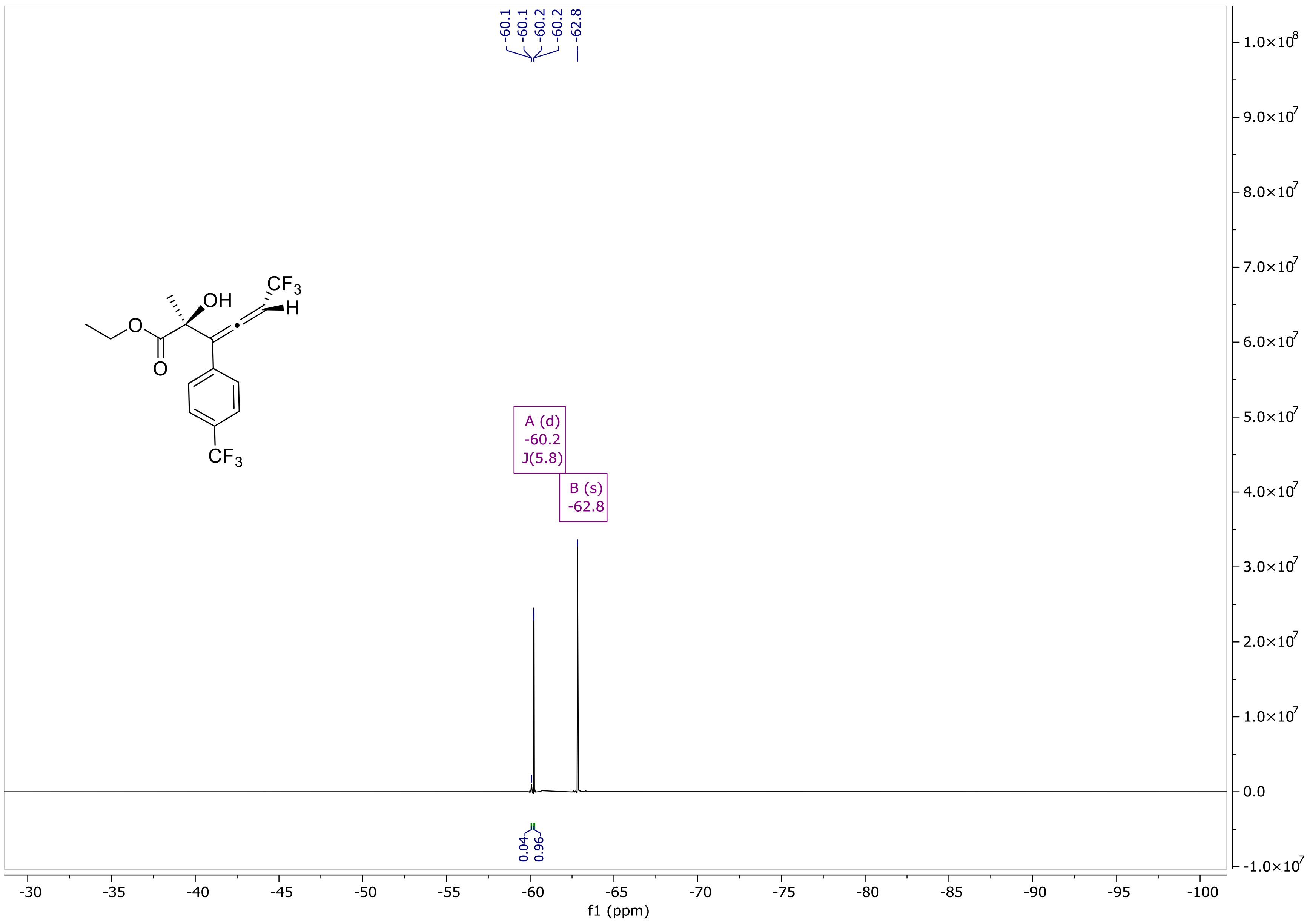

^{1}H NMR (400 MHz, $CDCl_3$) of compound **5r**

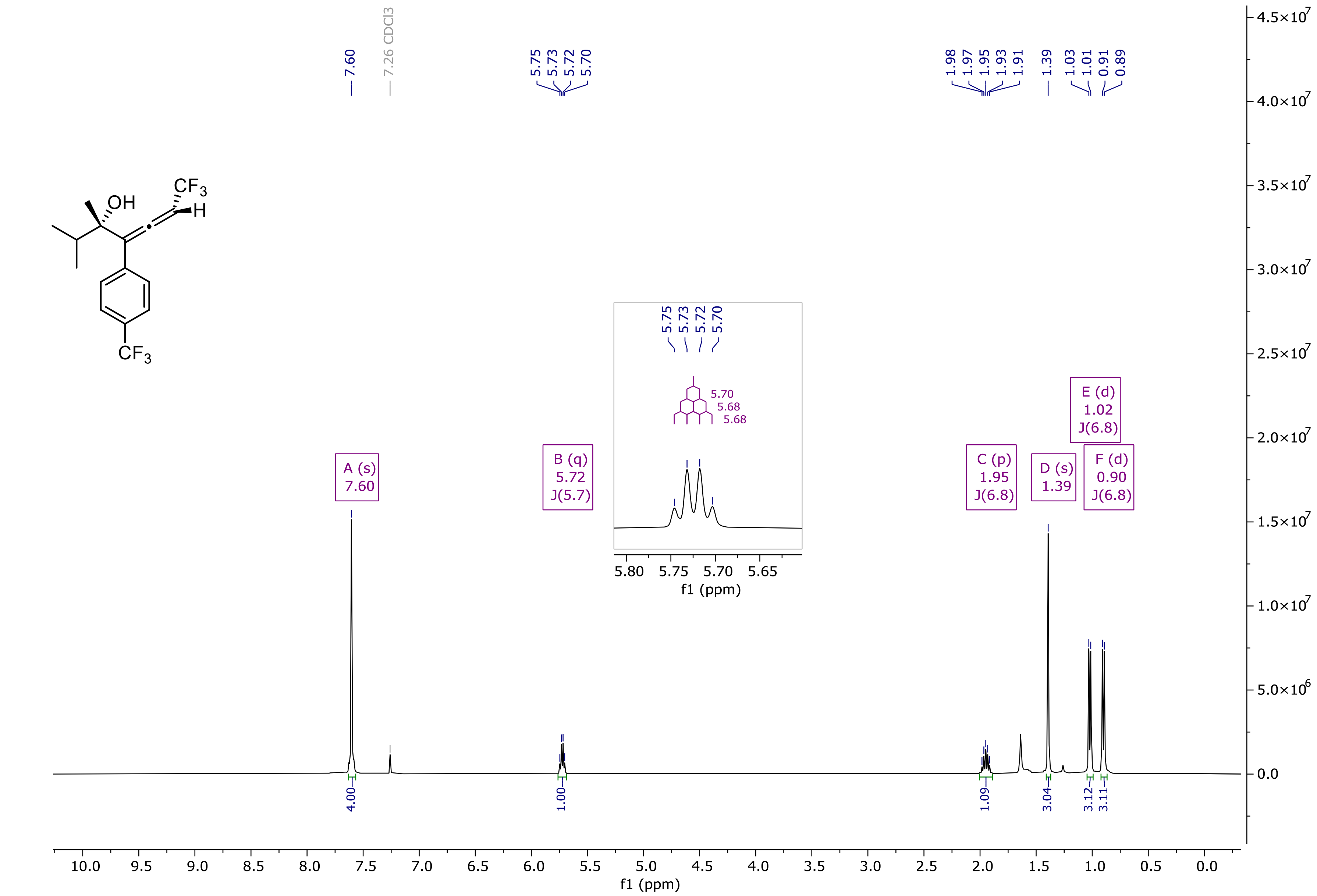

^{13}C NMR (126 MHz, $CDCl_3$) of compound **5r**

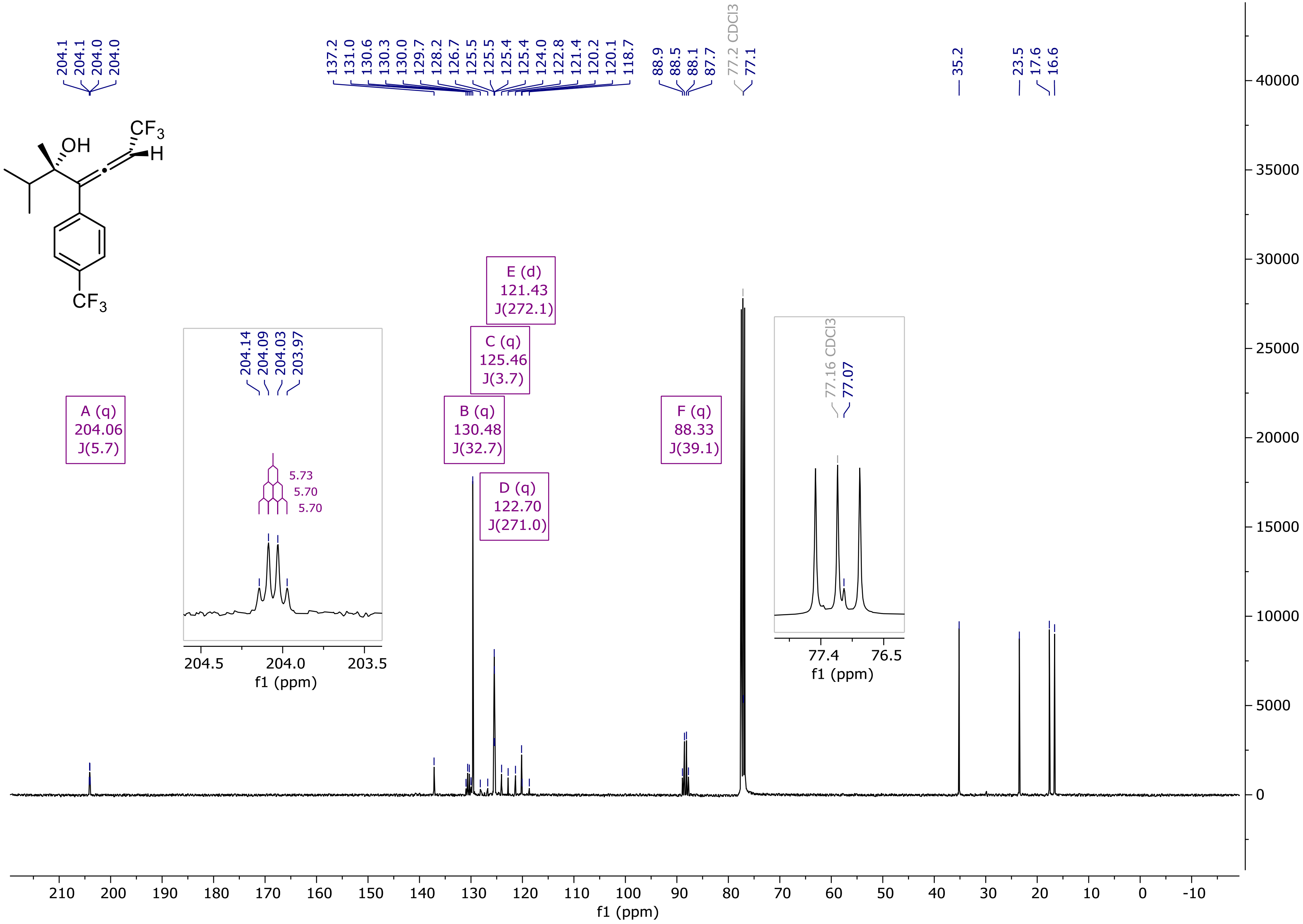

^{19}F NMR (377 MHz, $CDCl_3$) of compound **5r**

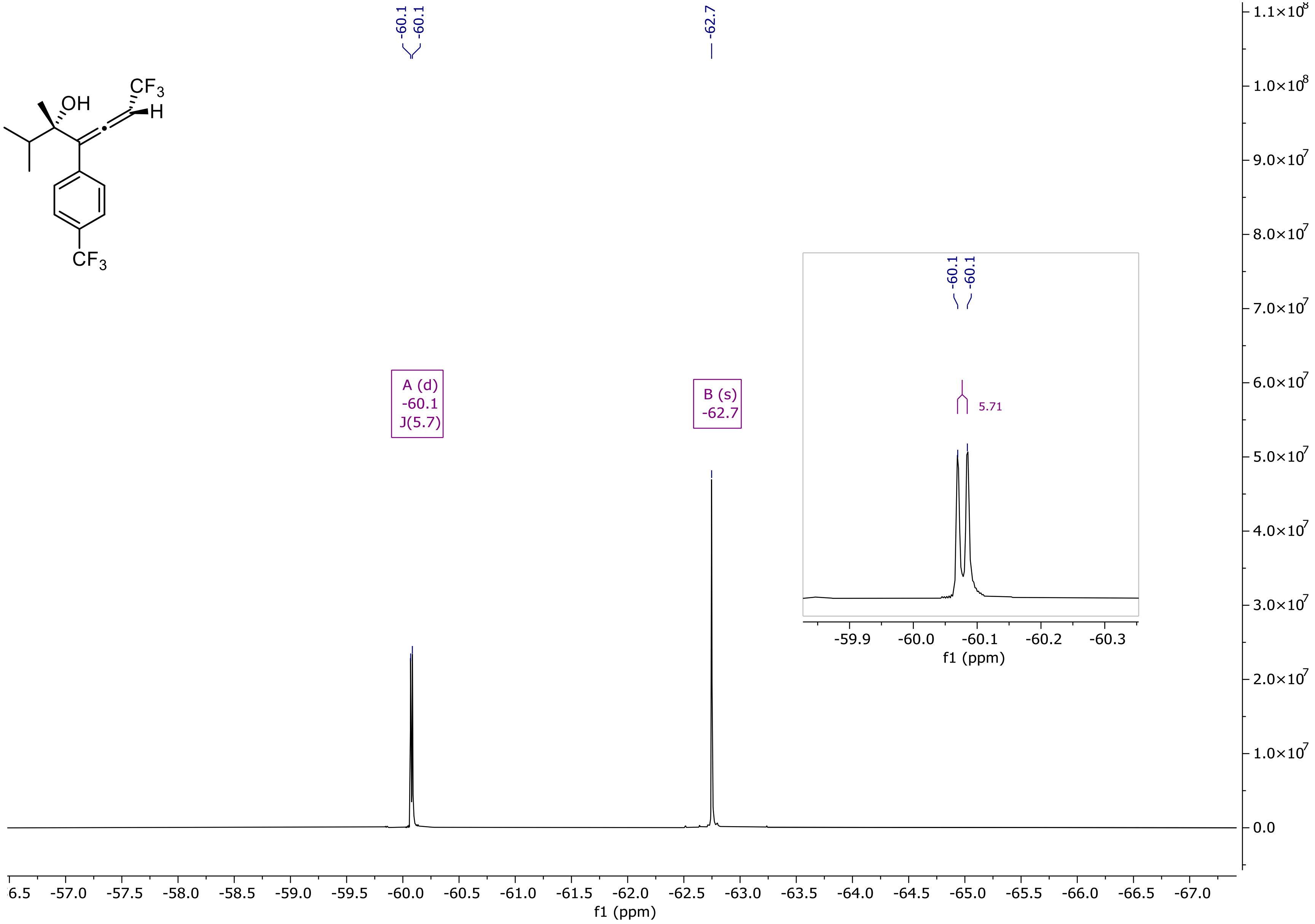

^{1}H NMR (400 MHz, $CDCl_3$) of compound **5s**

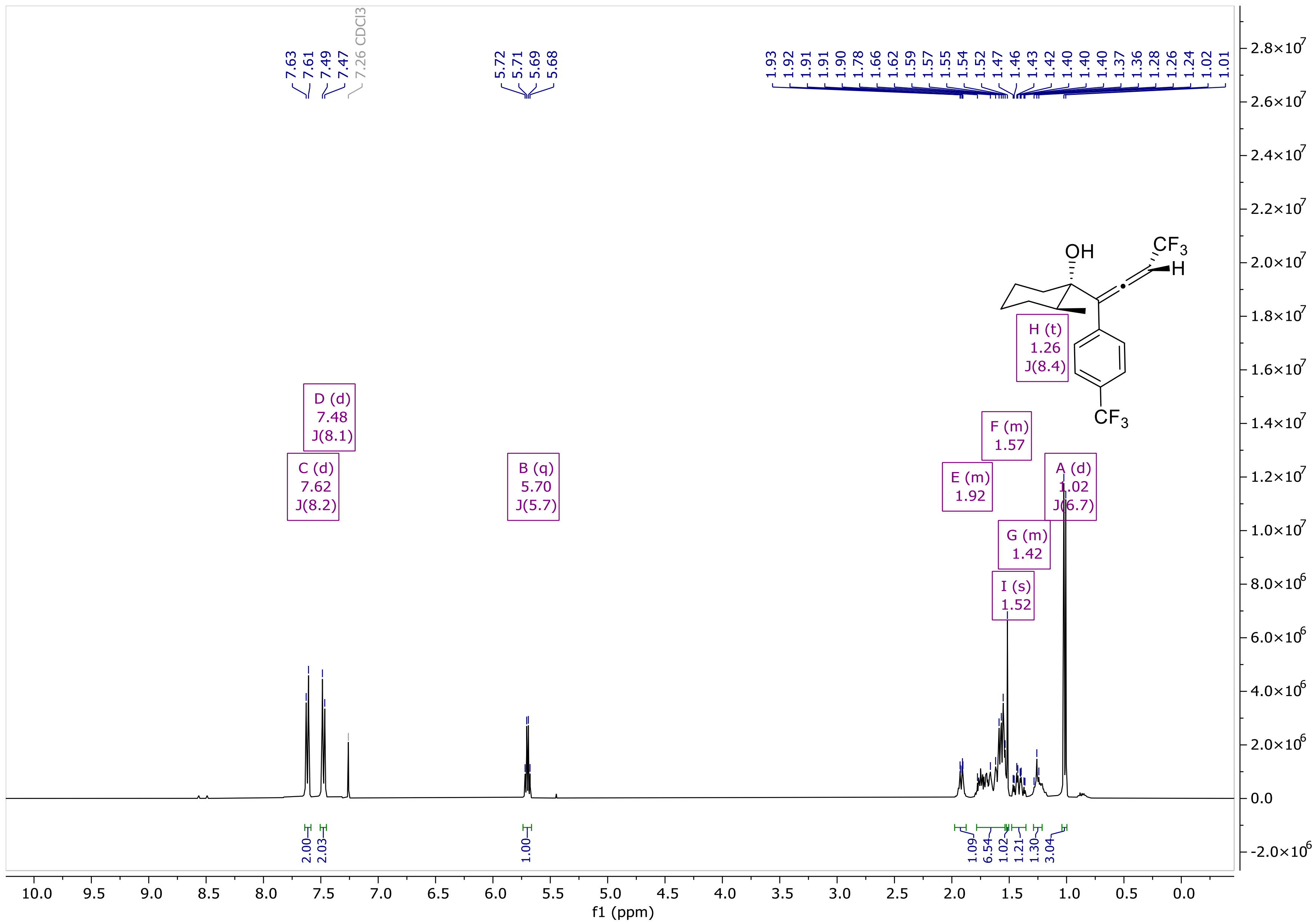

^{13}C NMR (126 MHz, $CDCl_3$) of compound **5s**

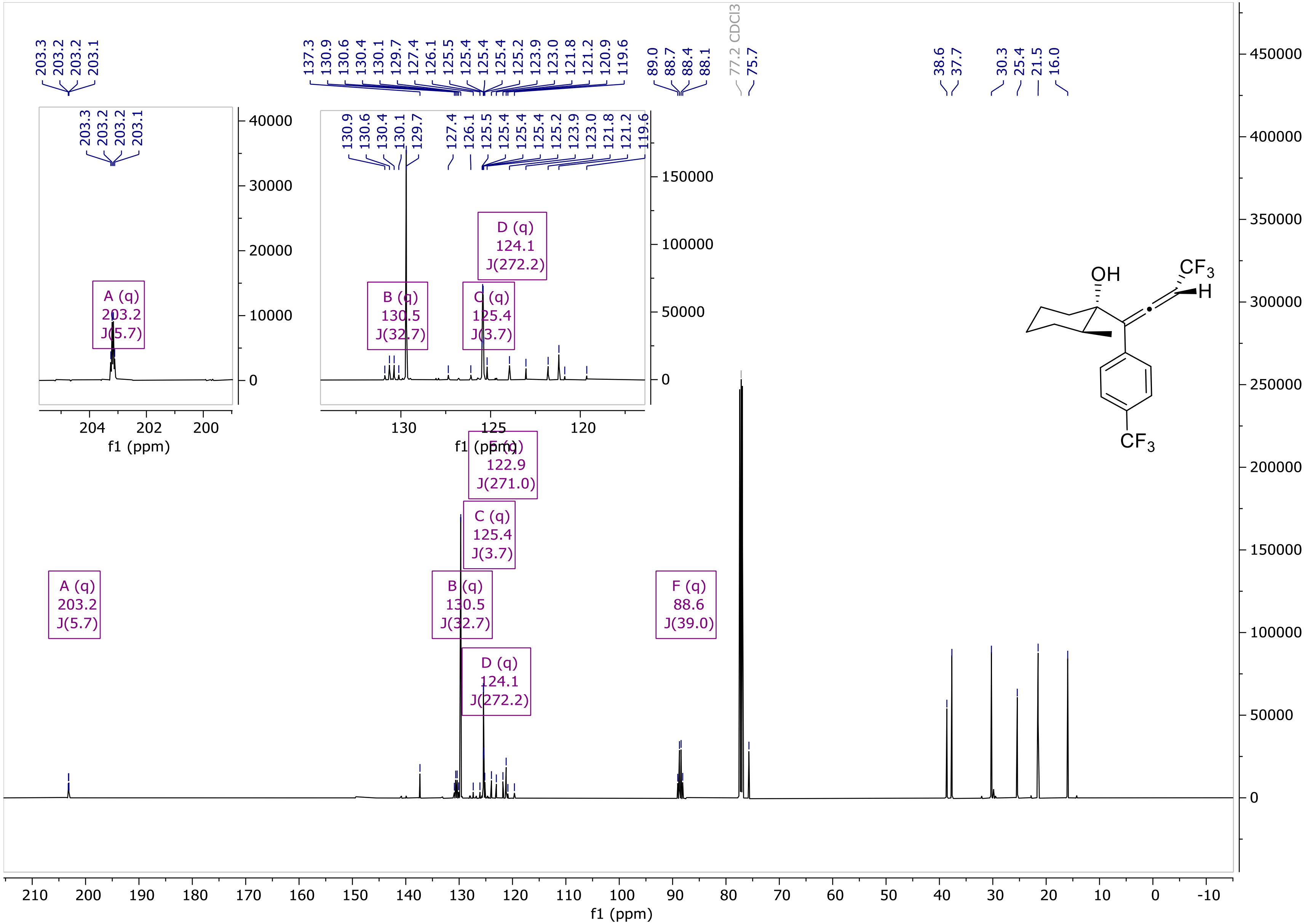

^{19}F NMR (377 MHz, $CDCl_3$) of compound **5s**

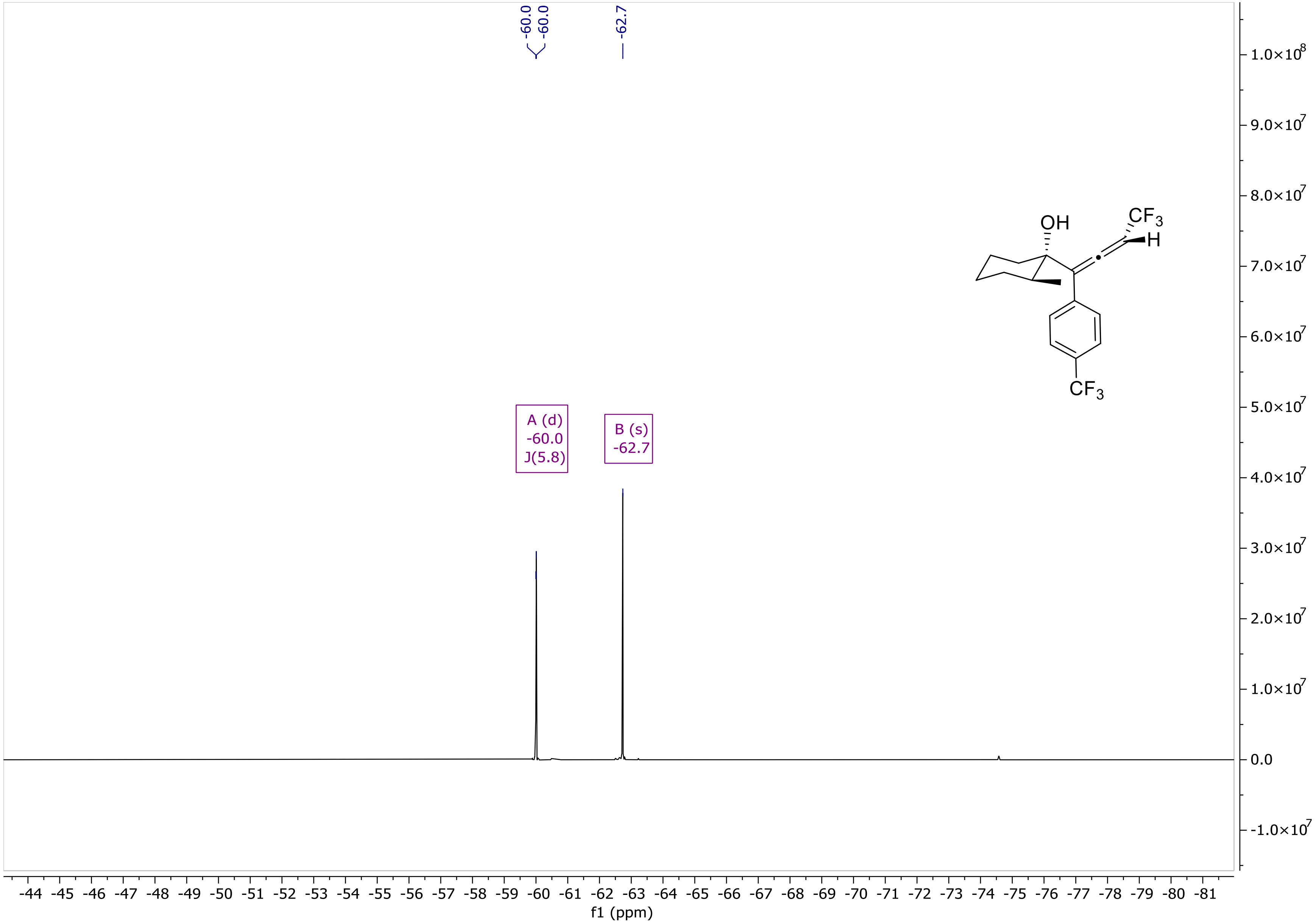

^{1}H NMR (400 MHz, $CDCl_3$) of compound **7a**

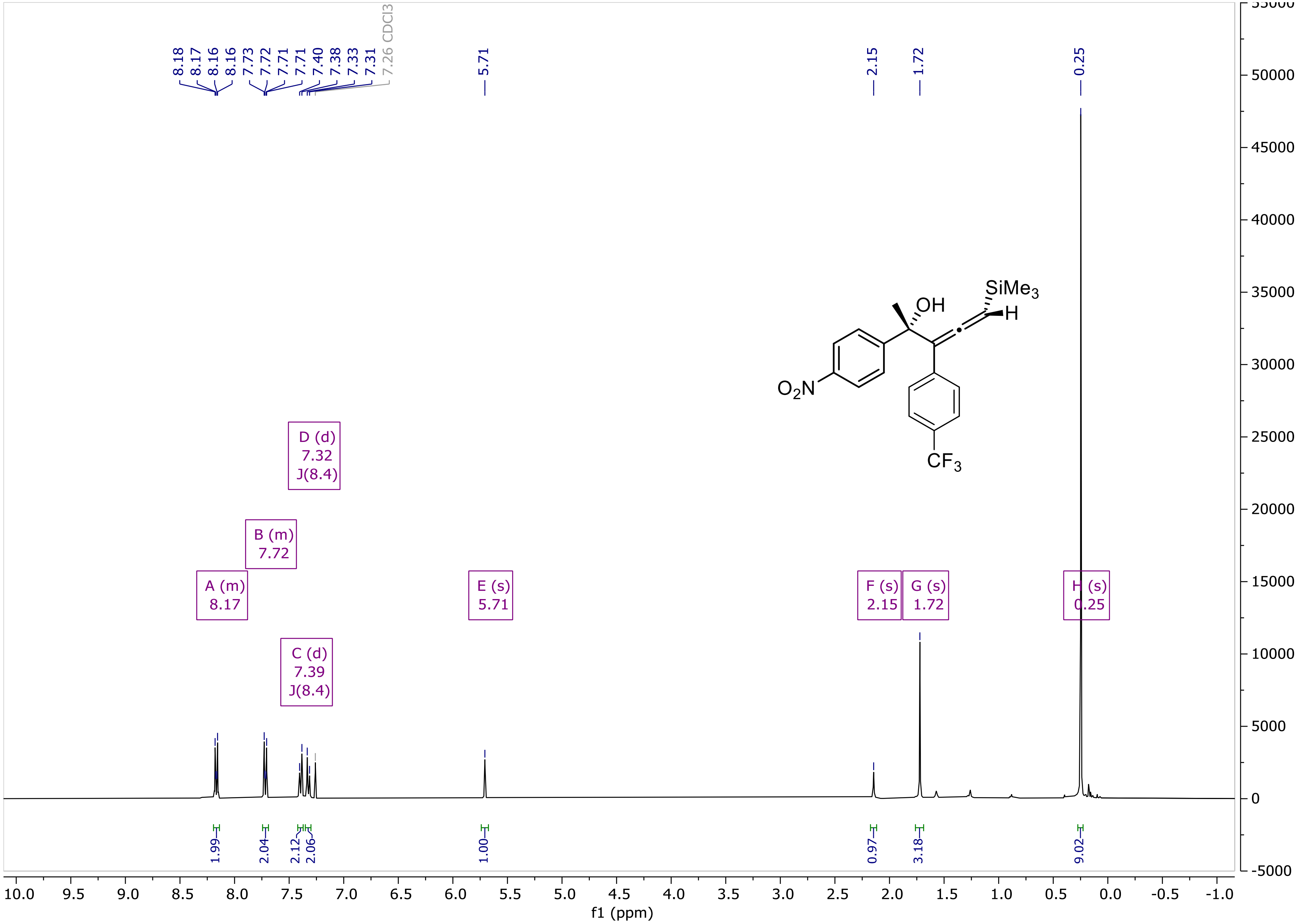

^{13}C NMR (126 MHz, $CDCl_3$) of compound **7a**

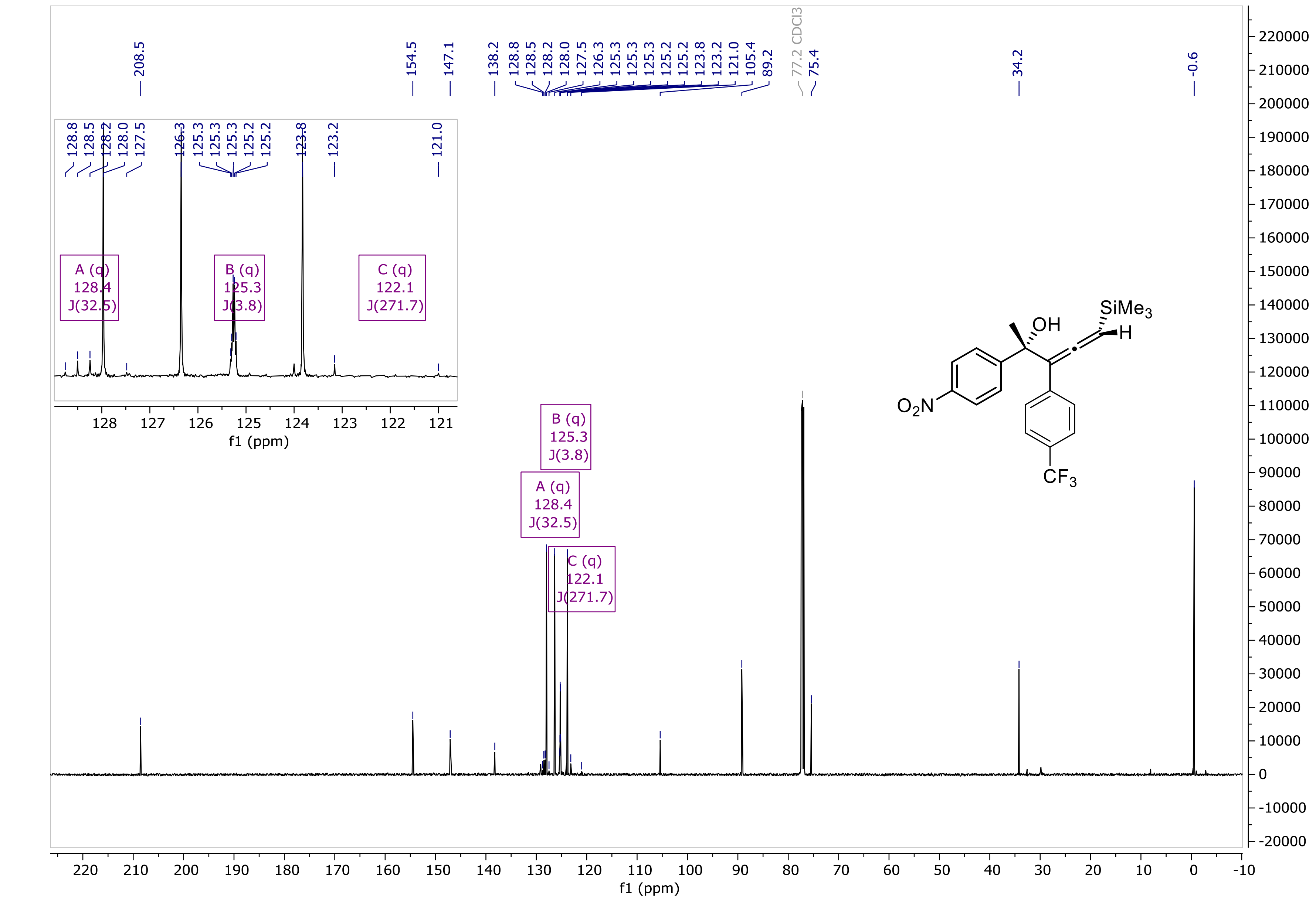

^{19}F NMR (377 MHz, $CDCl_3$) of compound **7a**

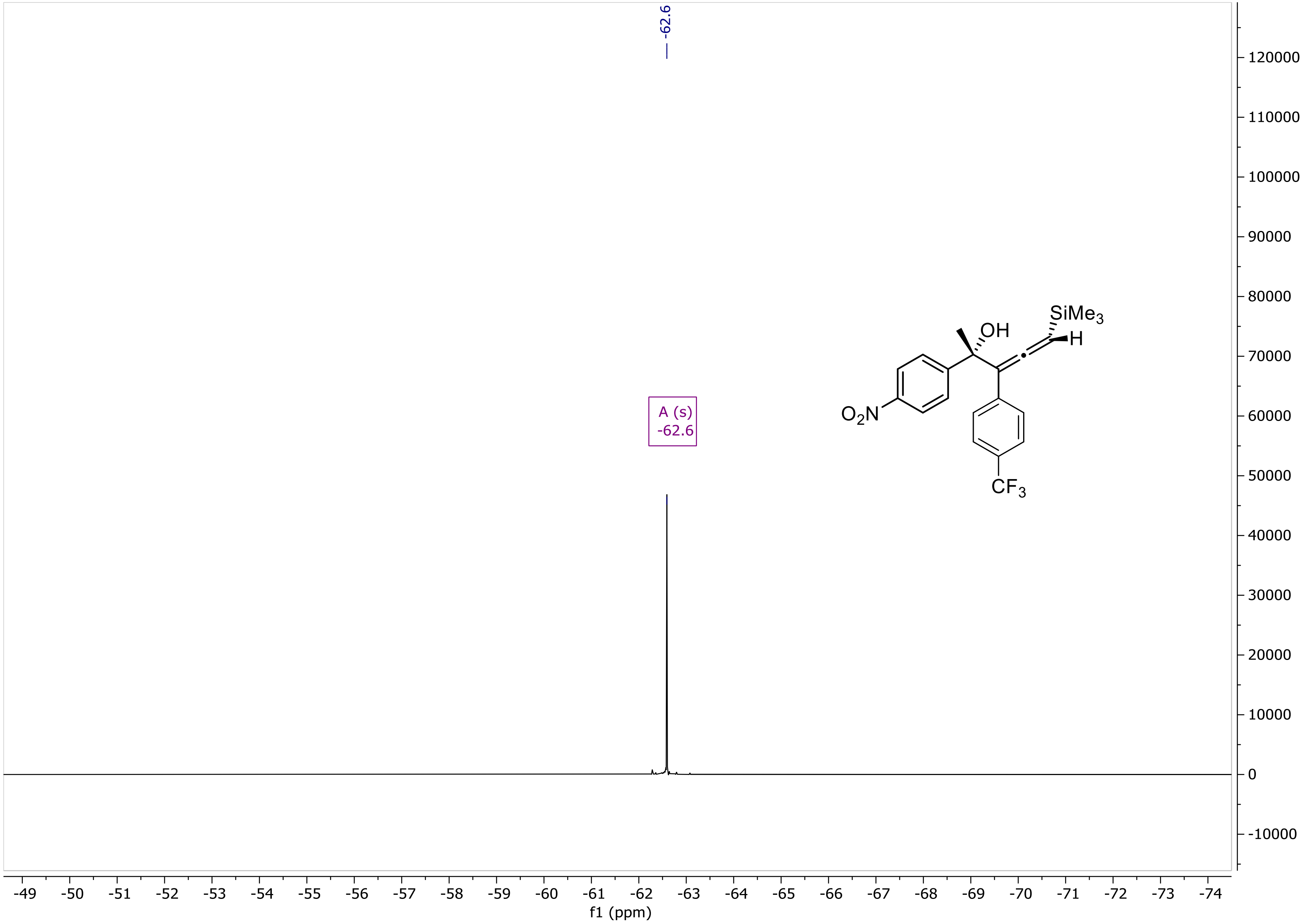

^{29}Si NMR (99 MHz, $CDCl_3$) of compound **7a**

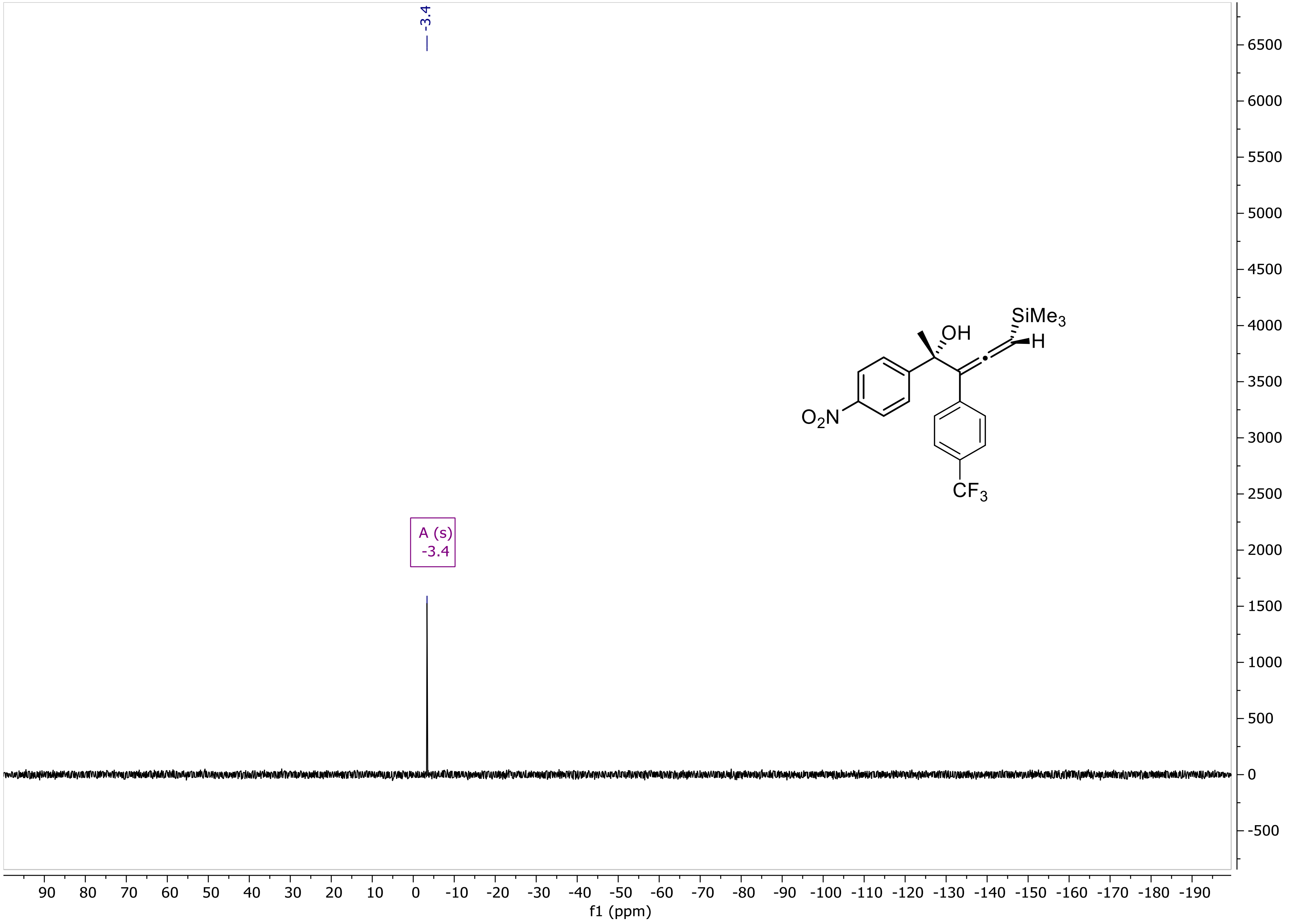

^{1}H NMR (400 MHz, $CDCl_3$) of compound **7b**

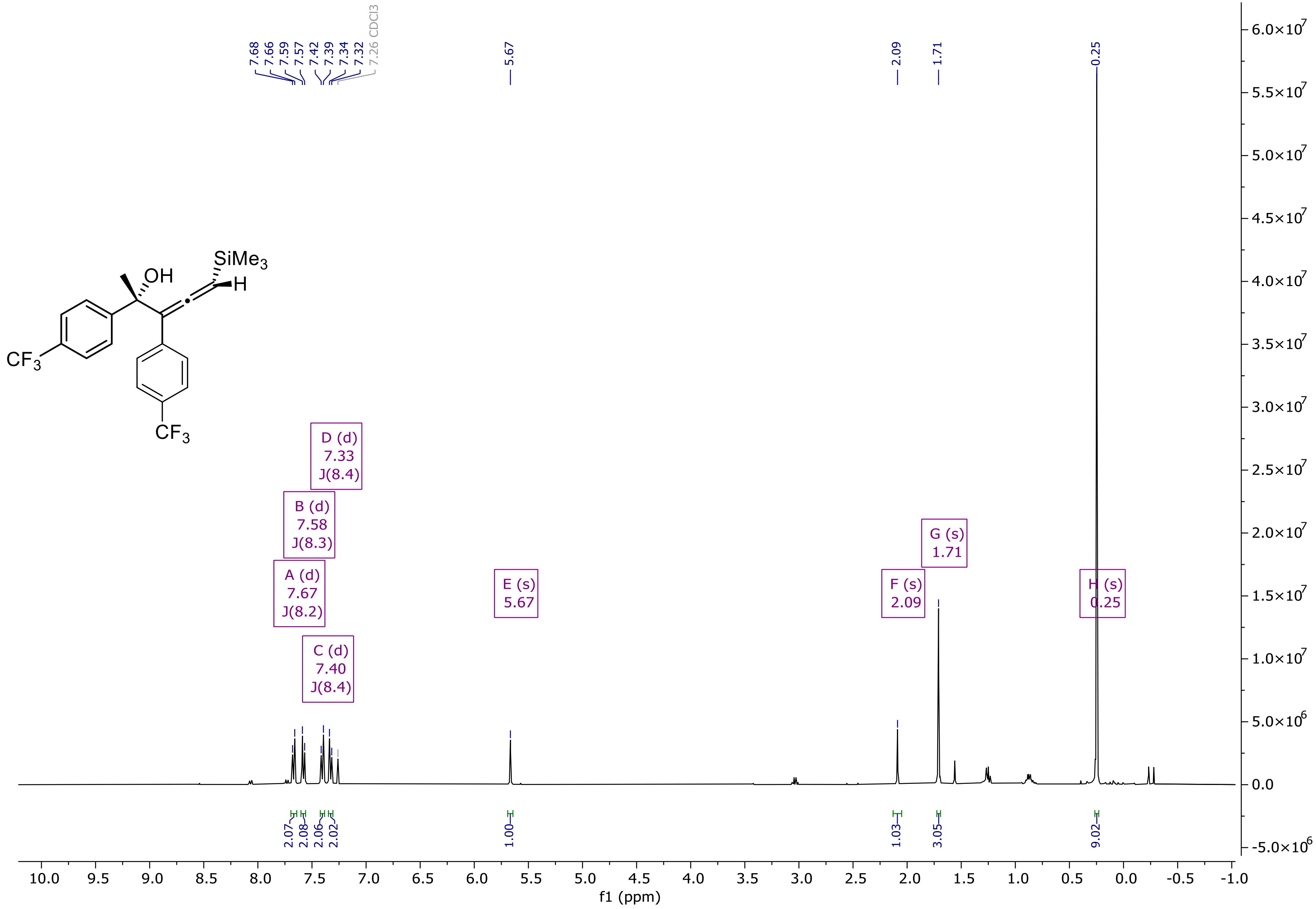

^{13}C NMR (126 MHz, $CDCl_3$) of compound **7b**

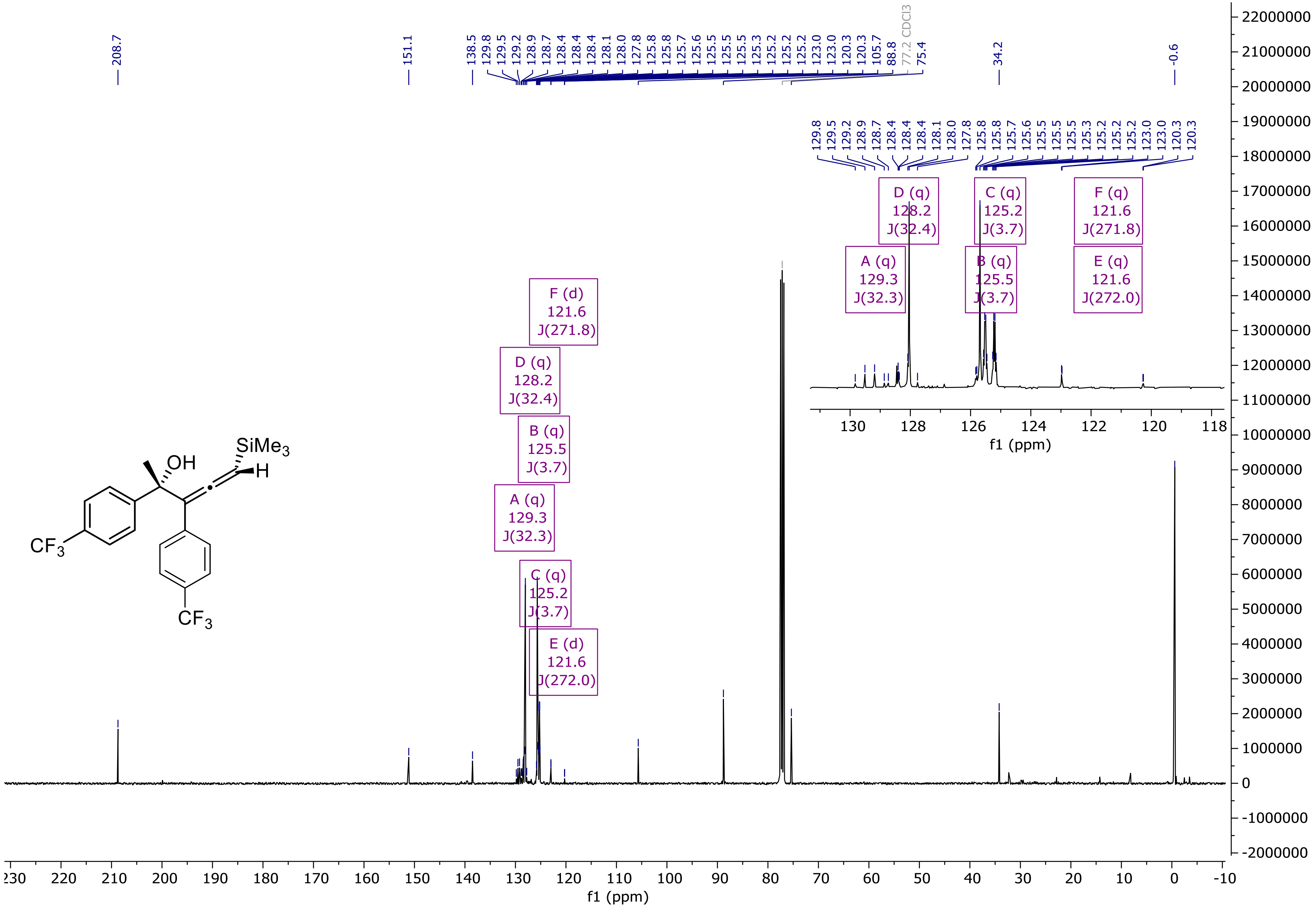

^{19}F NMR (377 MHz, $CDCl_3$) of compound **7b**

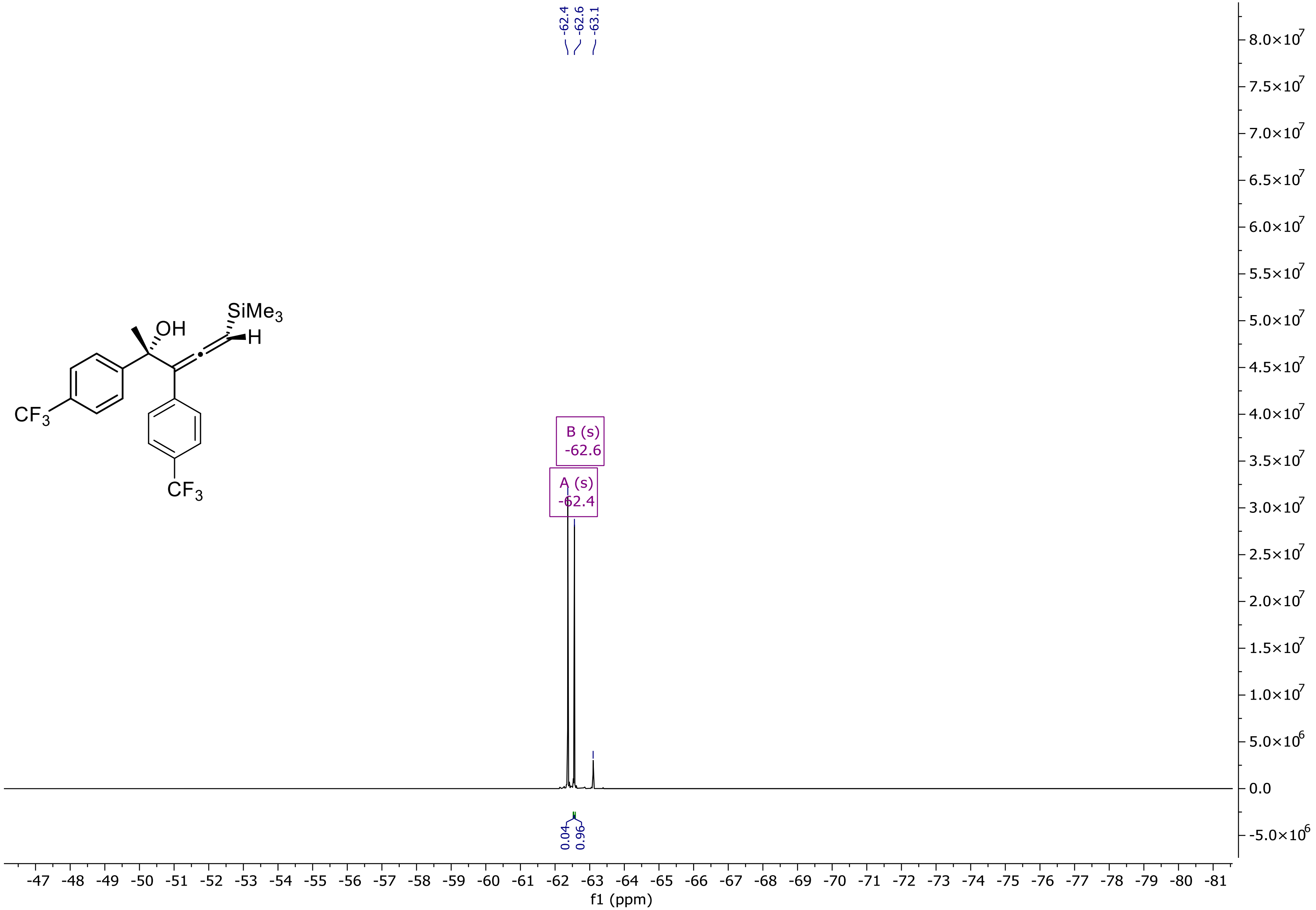

^{1}H NMR (400 MHz, $CDCl_3$) of compound **7c**

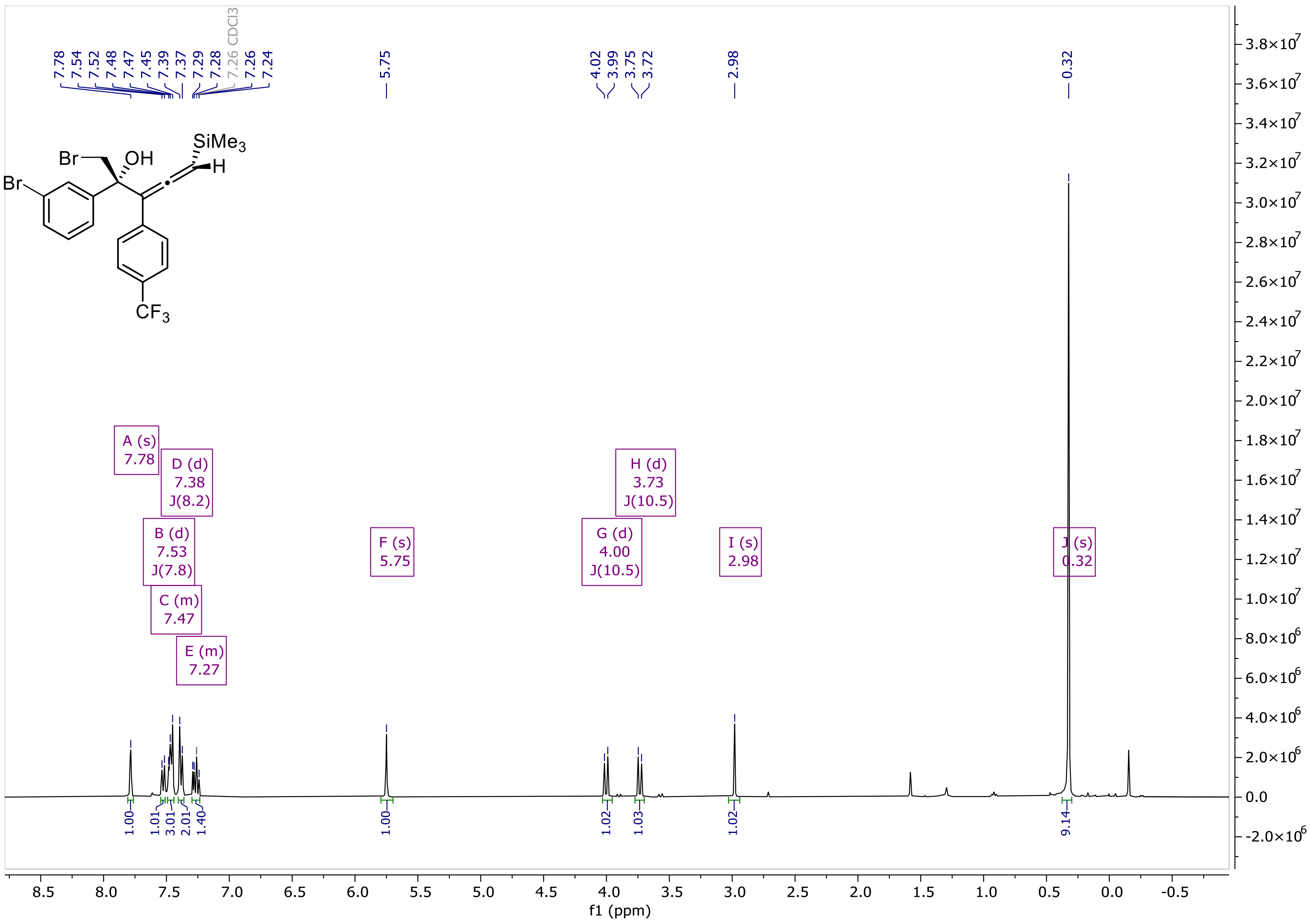

^{13}C NMR (126 MHz, CDCl$_3$) of compound **7c**

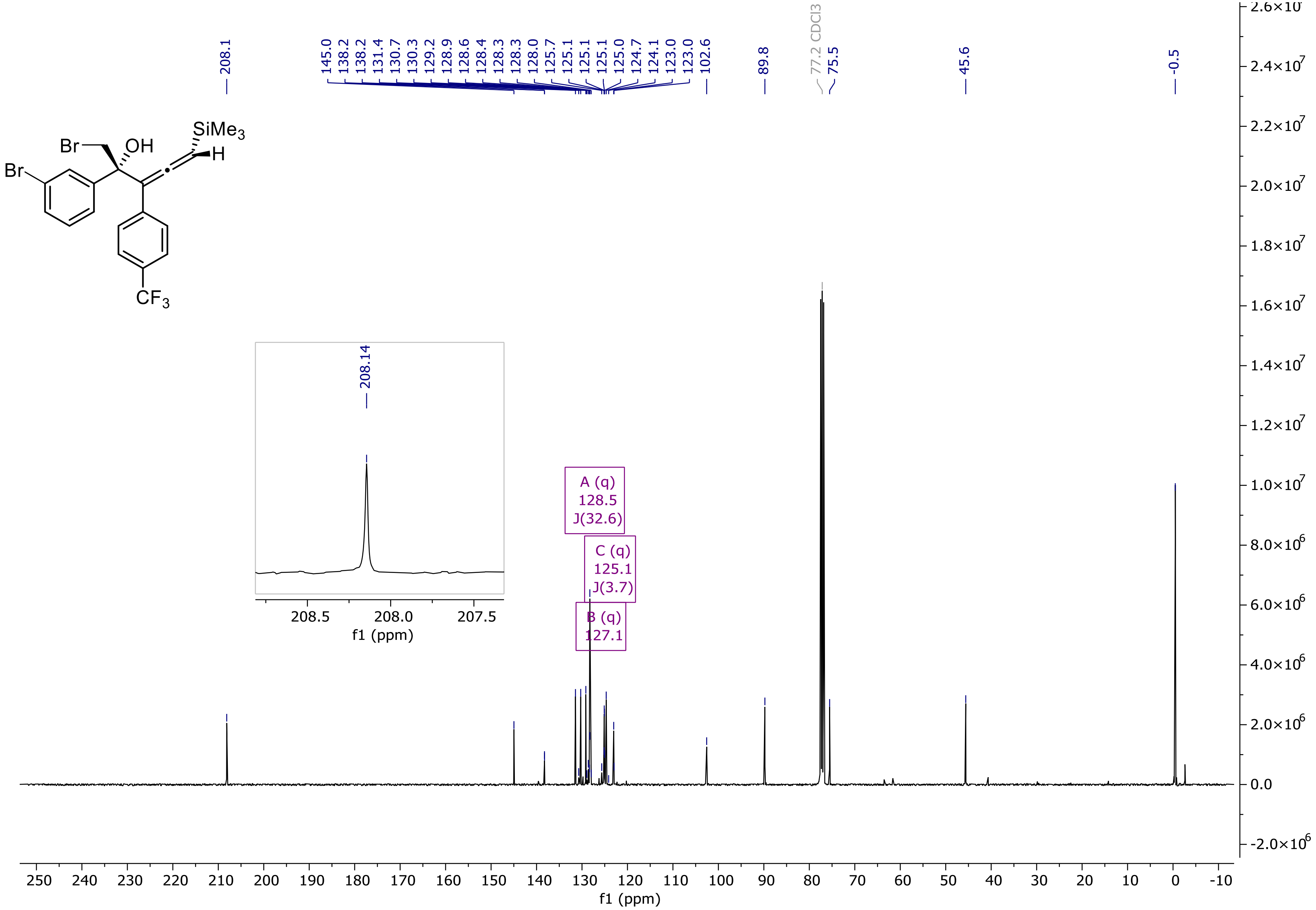

^{19}F NMR (377 MHz, $CDCl_3$) of compound **7c**

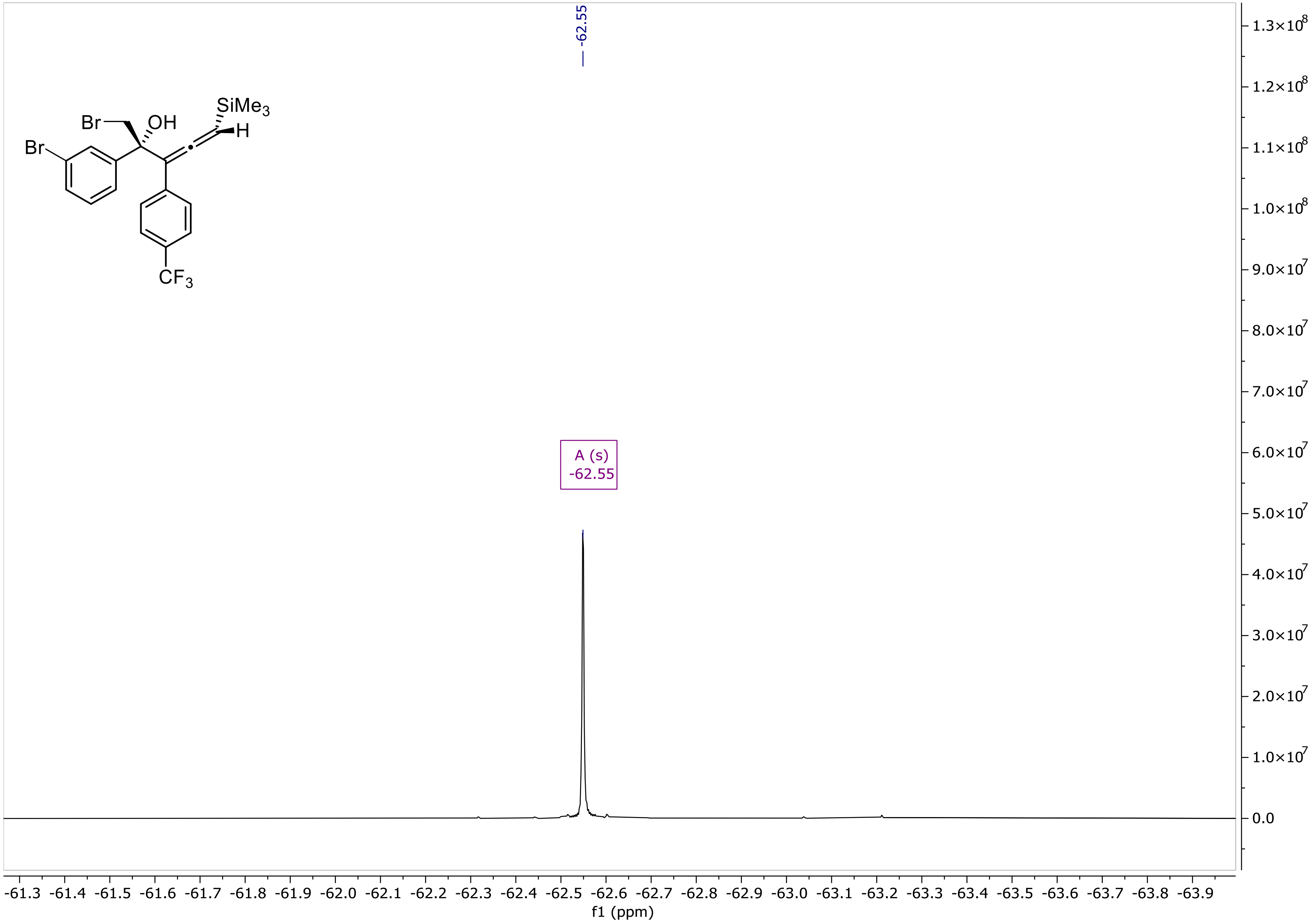

^{1}H NMR (400 MHz, $CDCl_3$) of compound **7d**

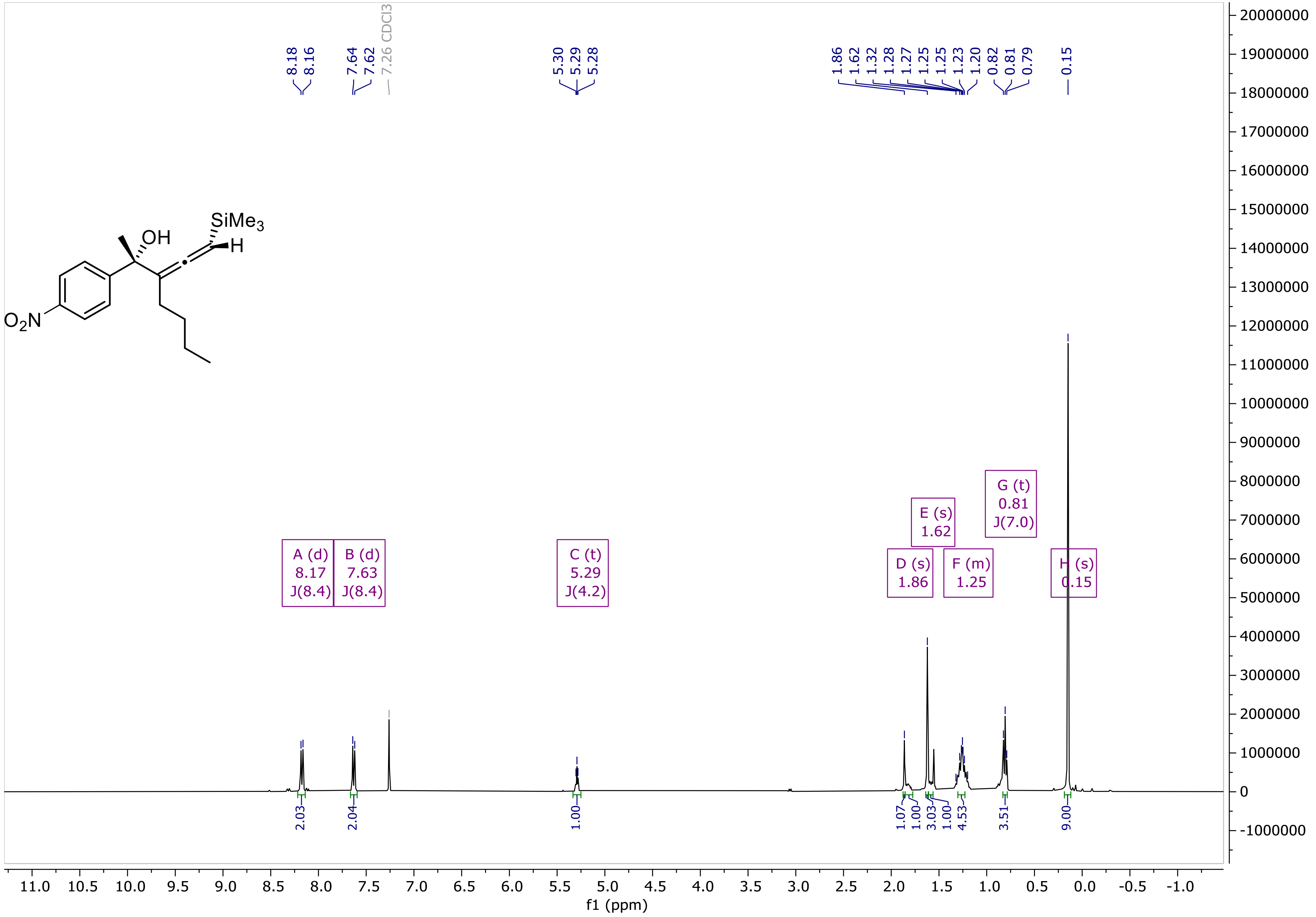

^{13}C NMR (126 MHz, $CDCl_3$) of compound **7d**

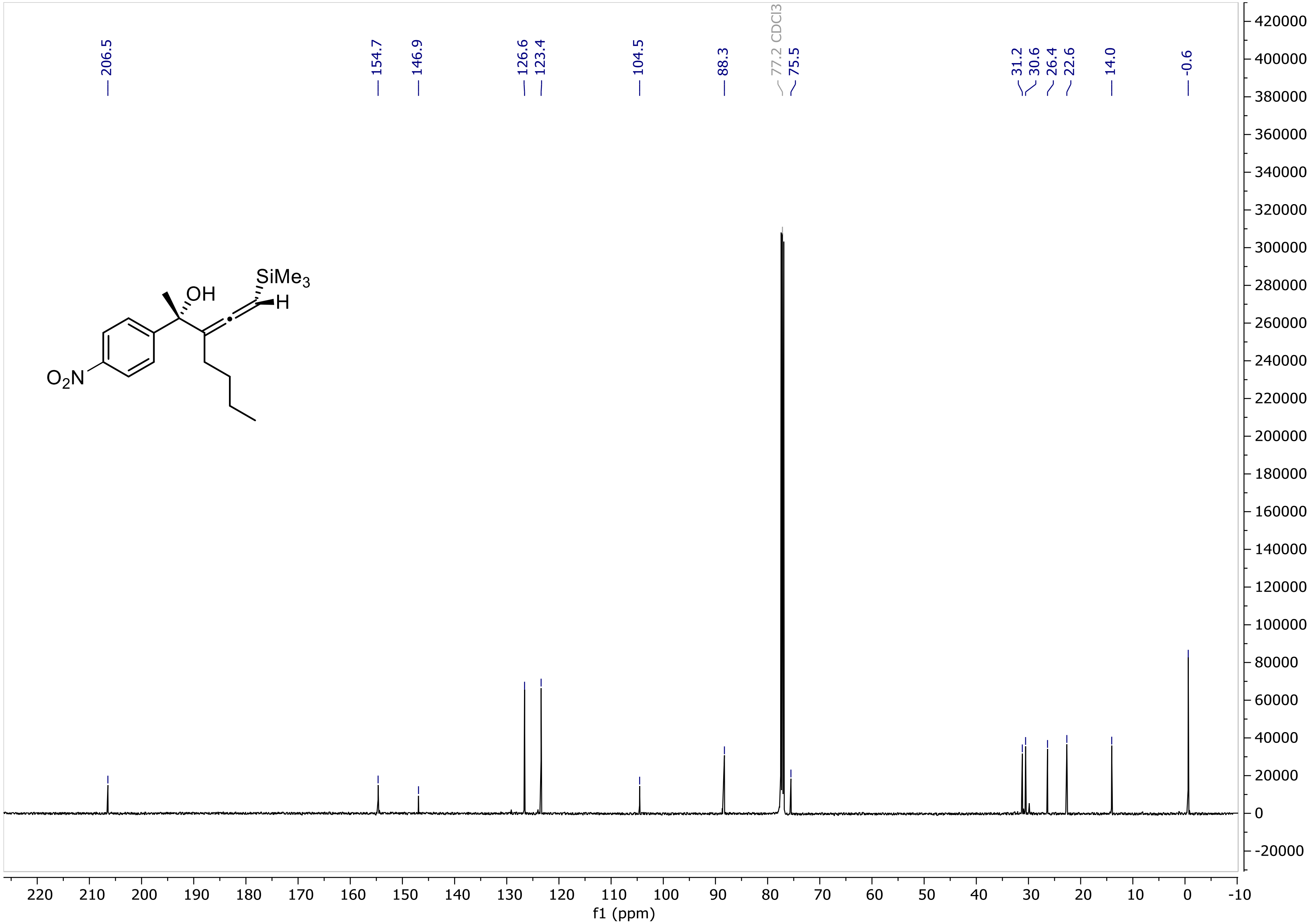
206.5
154.7
146.9
126.6
123.4
104.5
88.3
77.2 CDCl3
75.5
31.2
30.6
26.4
22.6
14.0
-0.6
SiMe3
OH
H
O2N
f1 (ppm)

^{19}F NMR (377 MHz, $CDCl_3$) of compound **7d**

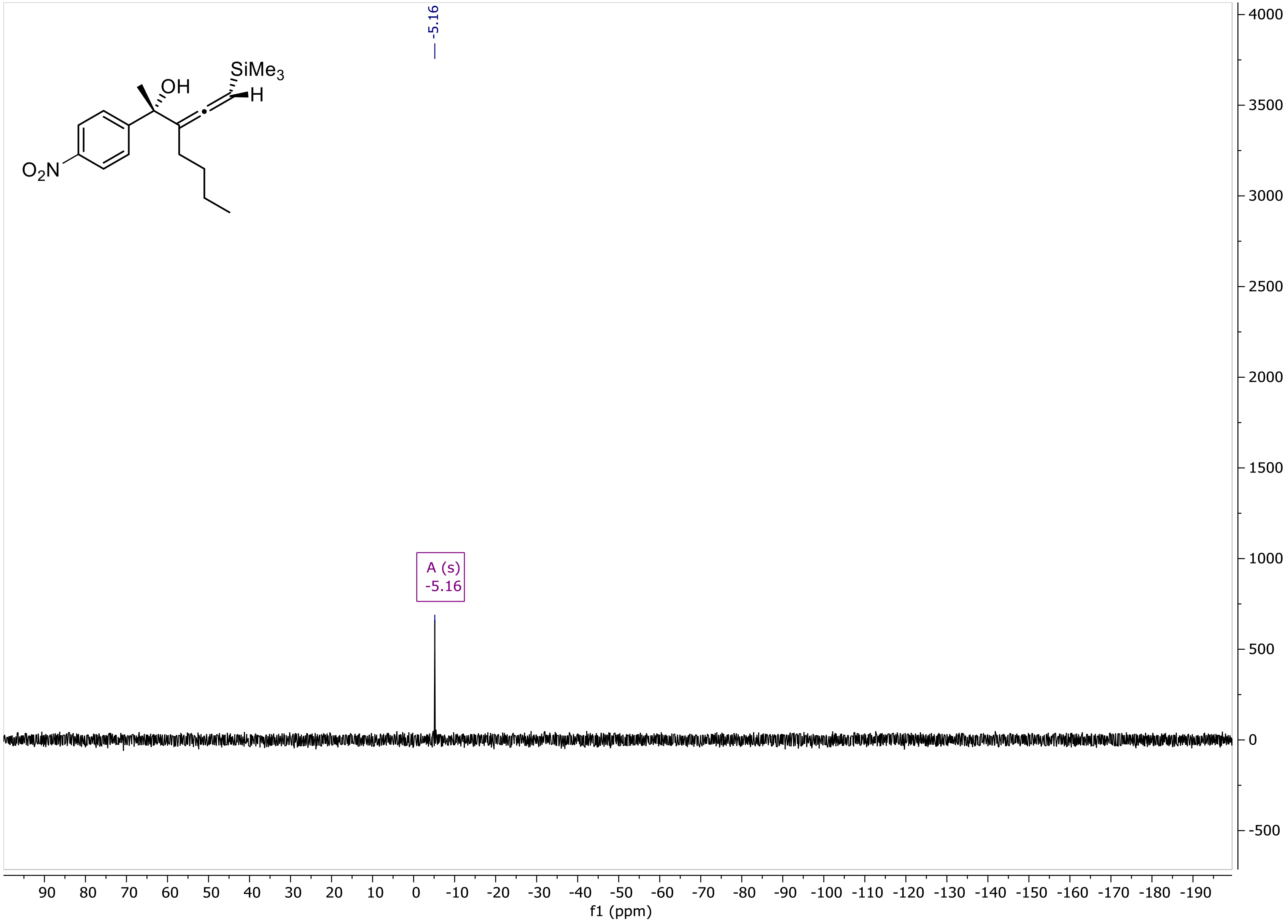

^{1}H NMR (500 MHz, $CDCl_3$) of compound **8**

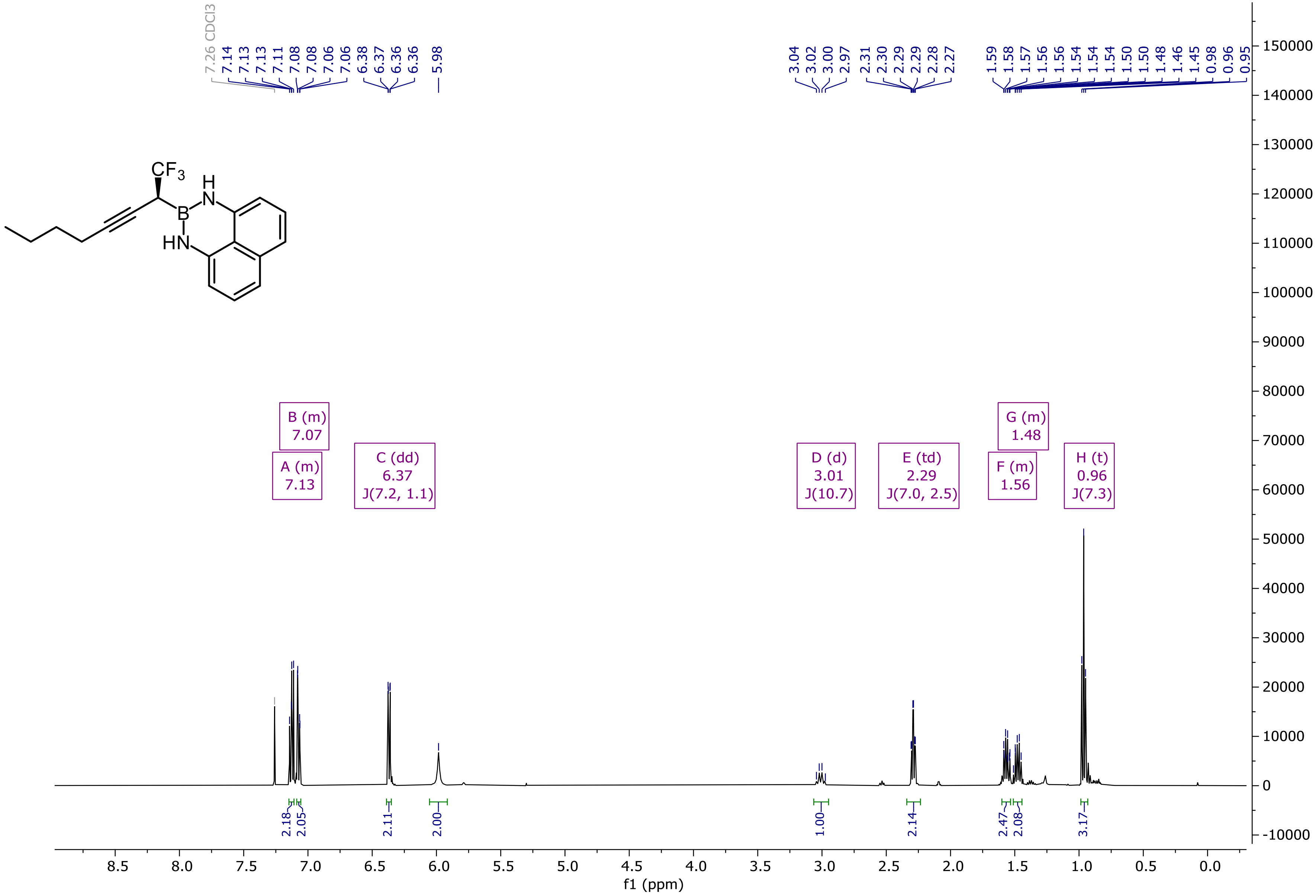

^{13}C NMR (126 MHz, $CDCl_3$) of compound **8**

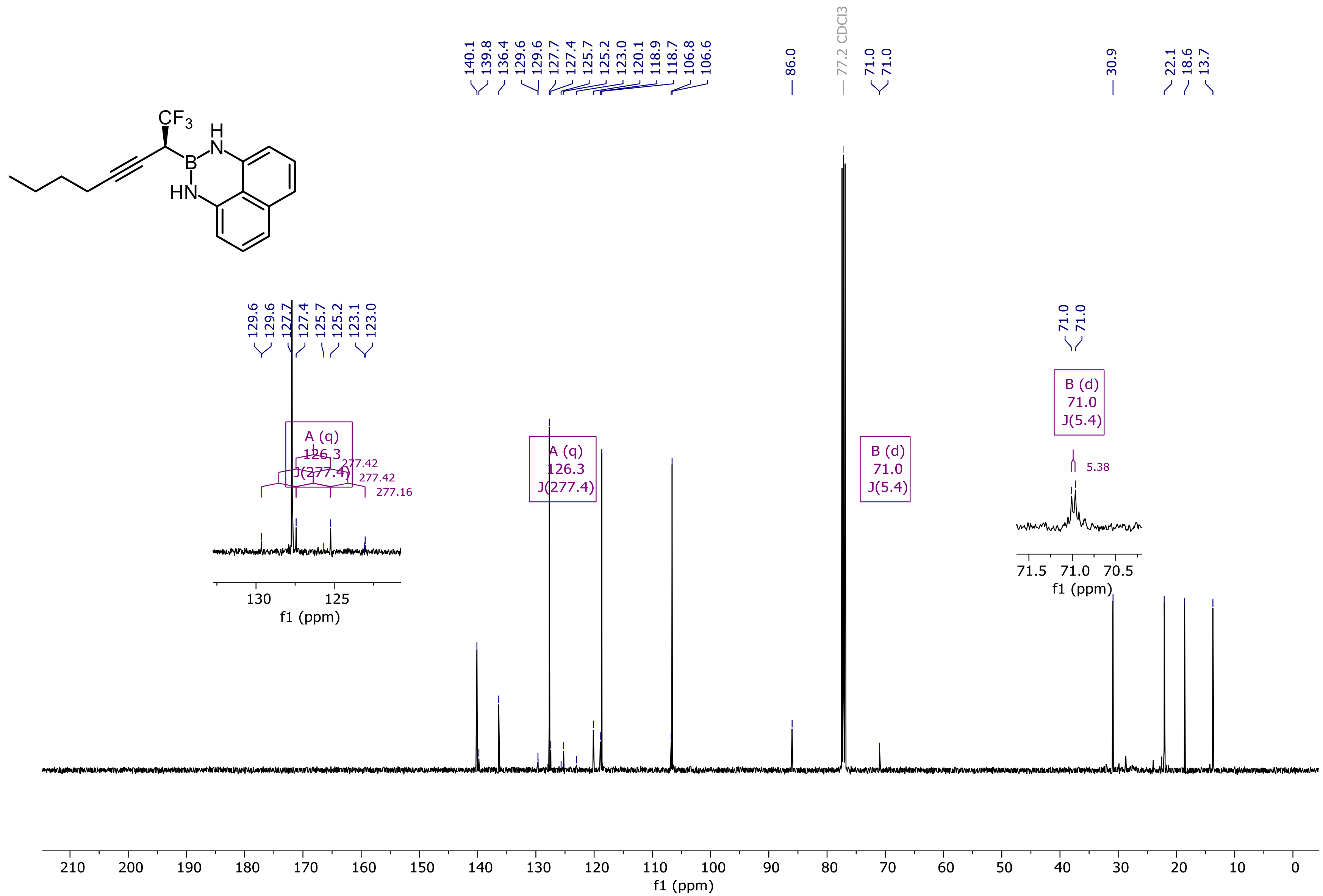

^{19}F NMR (377 MHz, $CDCl_3$) of compound **8**

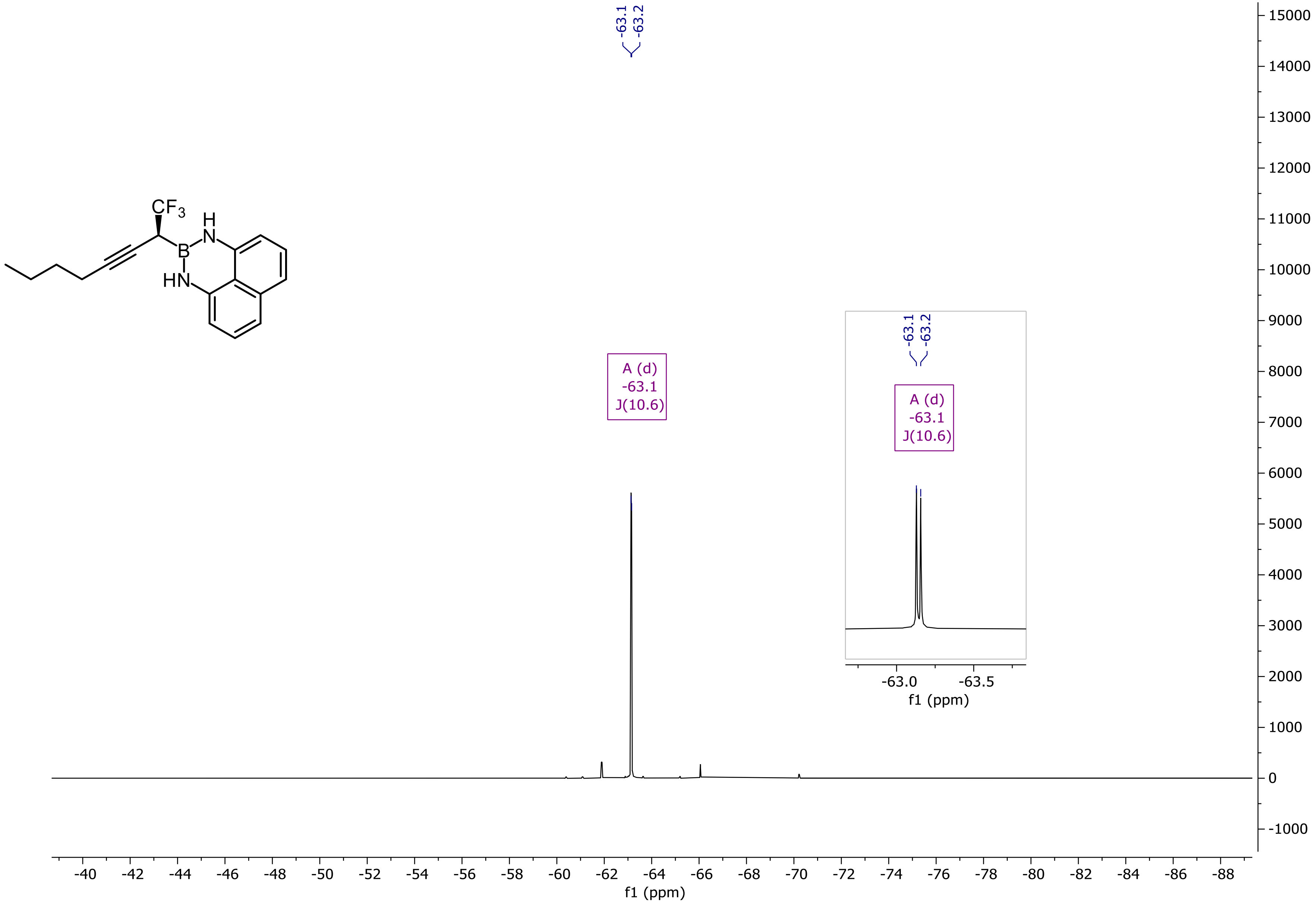

^{11}B NMR (128 MHz, $CDCl_3$) of compound **8**

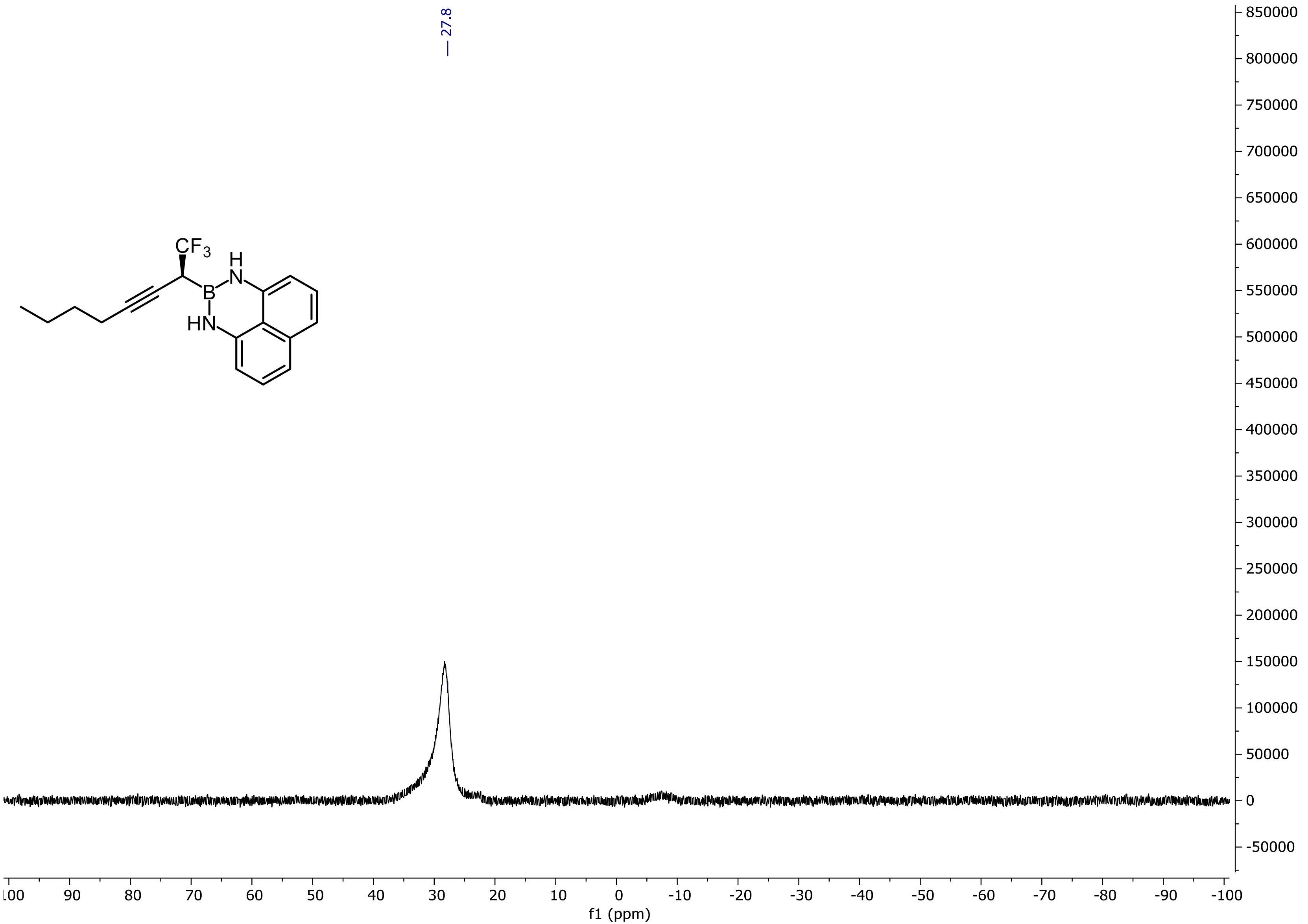

^{1}H NMR (500 MHz, $CDCl_3$) of compound **10b**

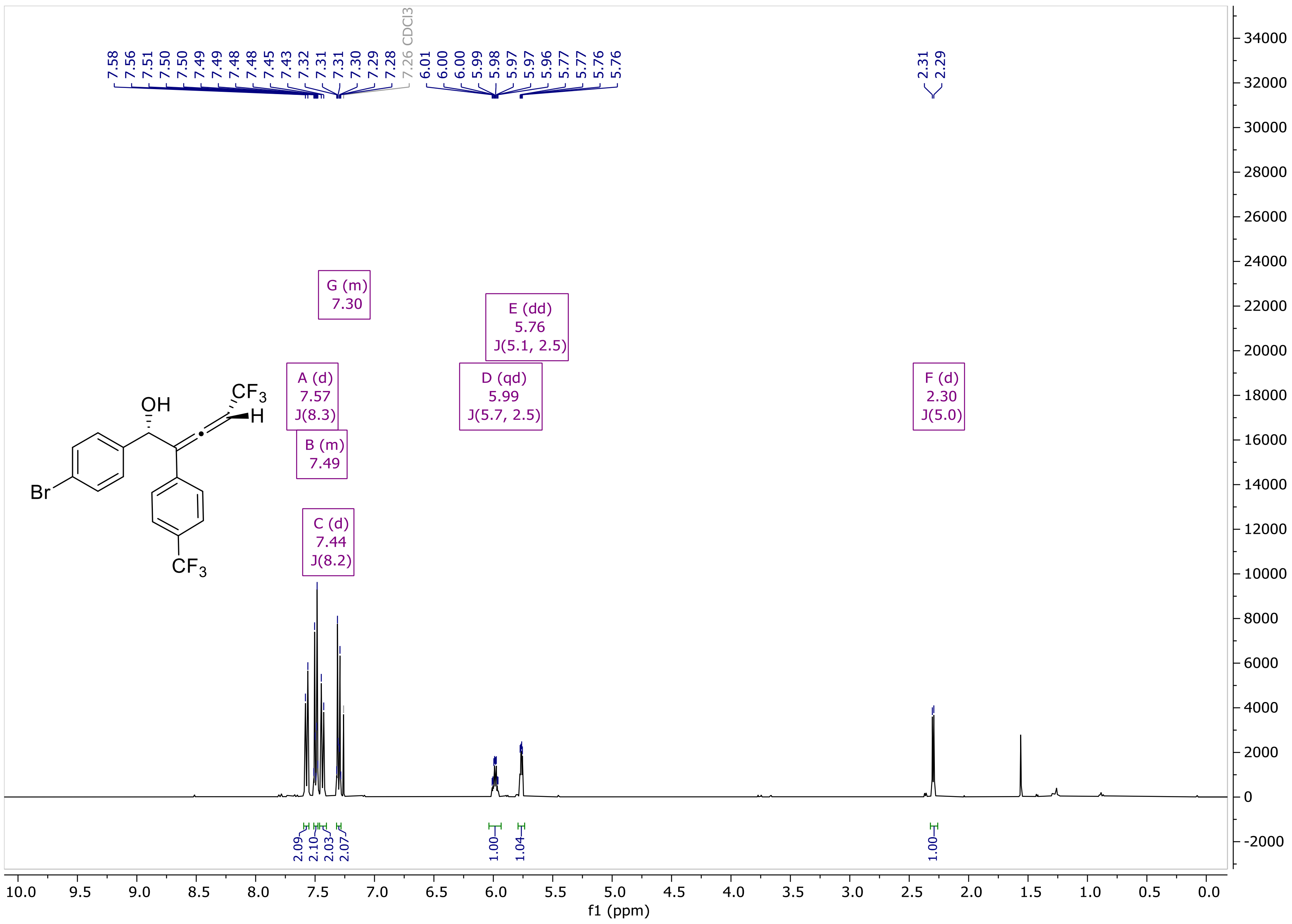

^{13}C NMR (101 MHz, $CDCl_3$) of compound **10b**

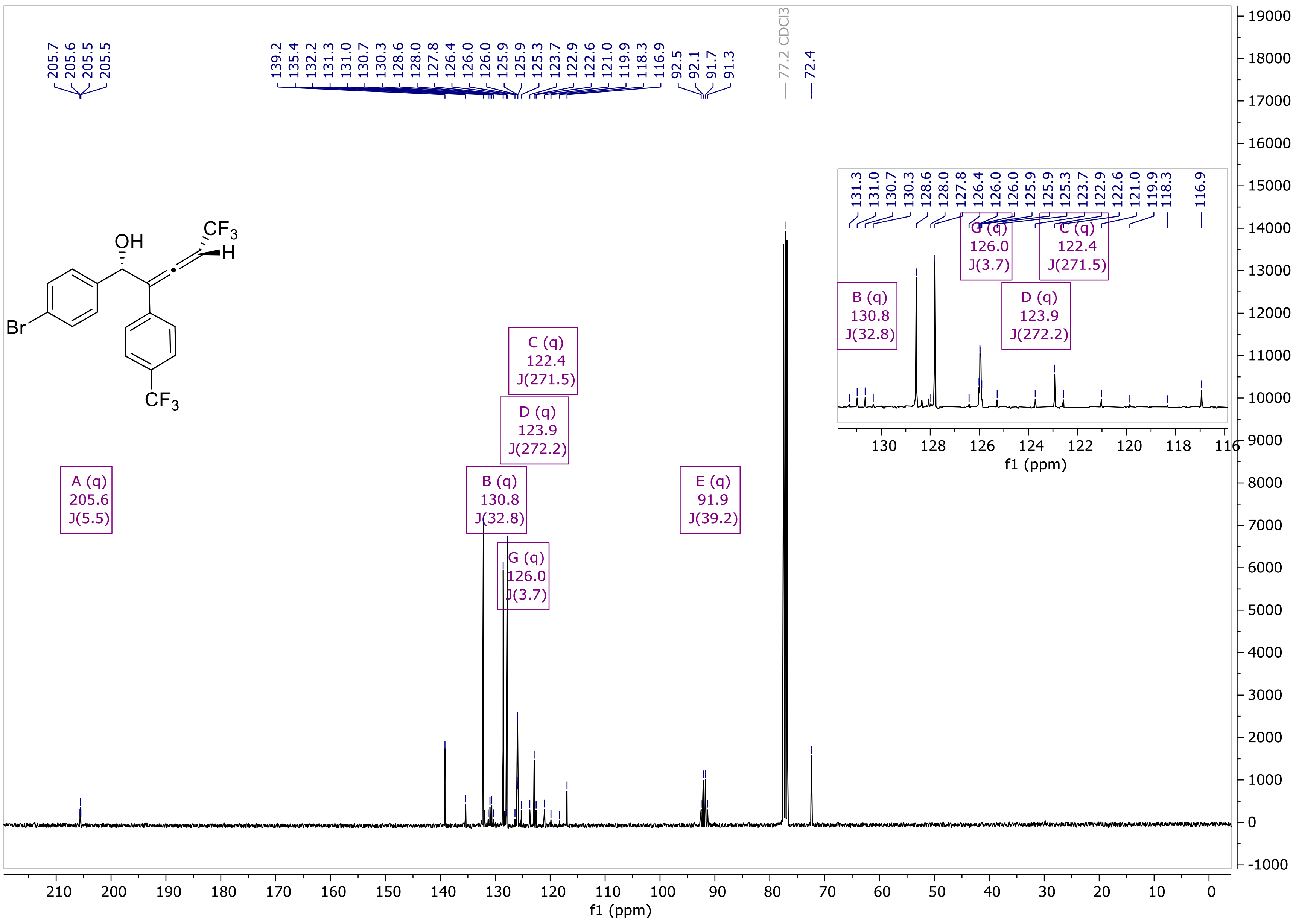

^{19}F NMR (377 MHz, CDCl$_3$) of compound **10b**

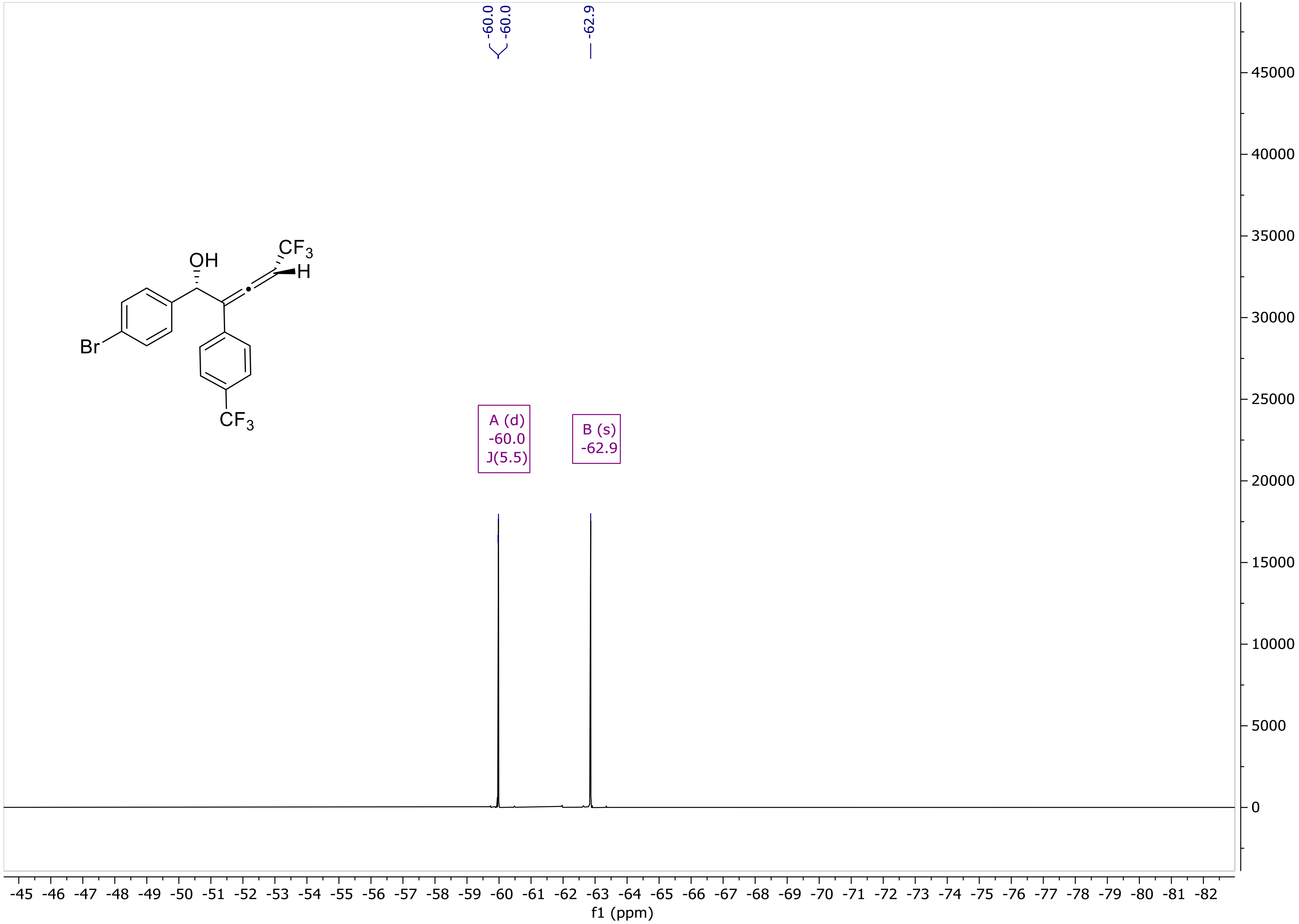

^{1}H NMR (500 MHz, $CDCl_3$) of compound **10c**

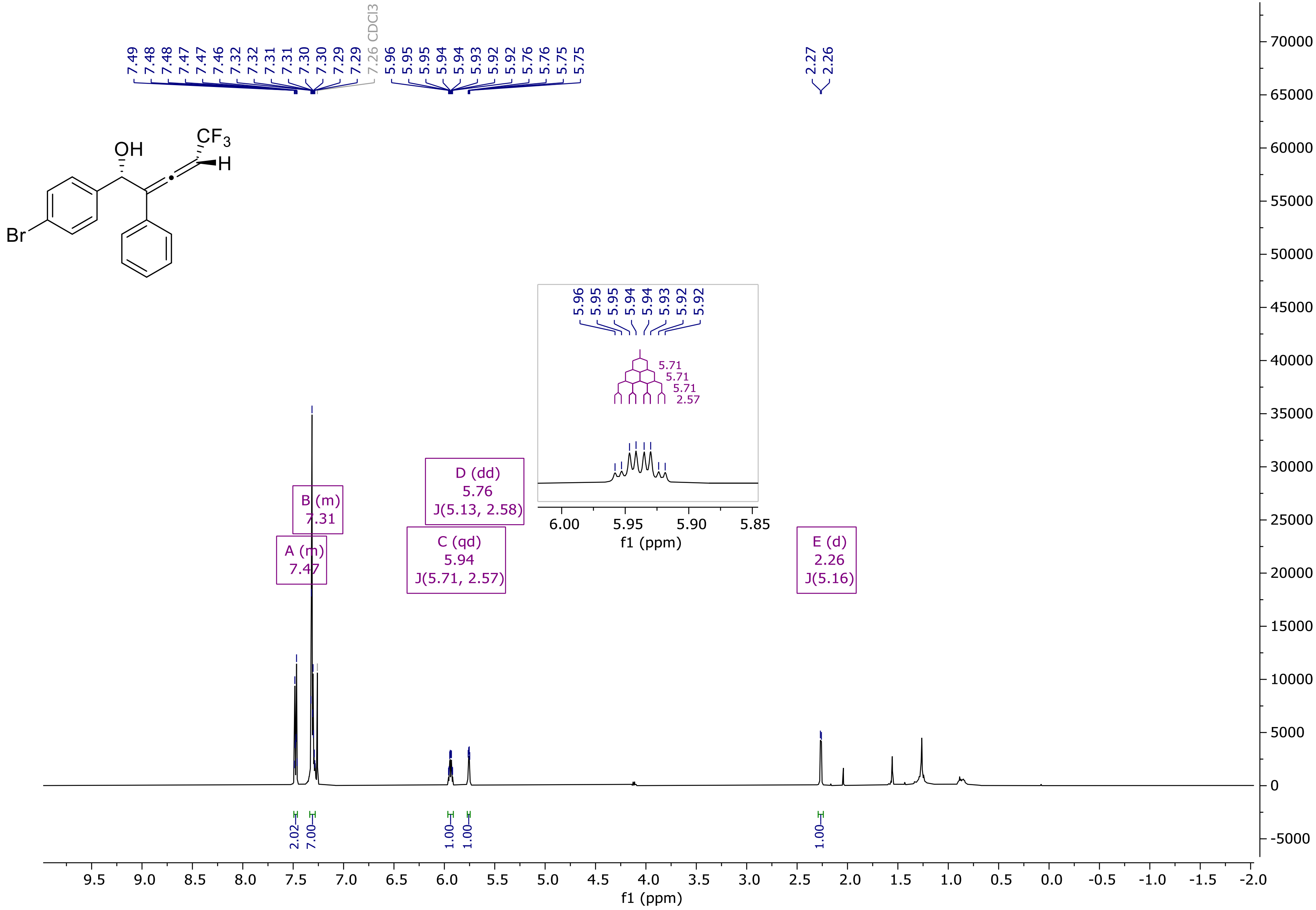

^{13}C NMR (101 MHz, $CDCl_3$) of compound **10c**

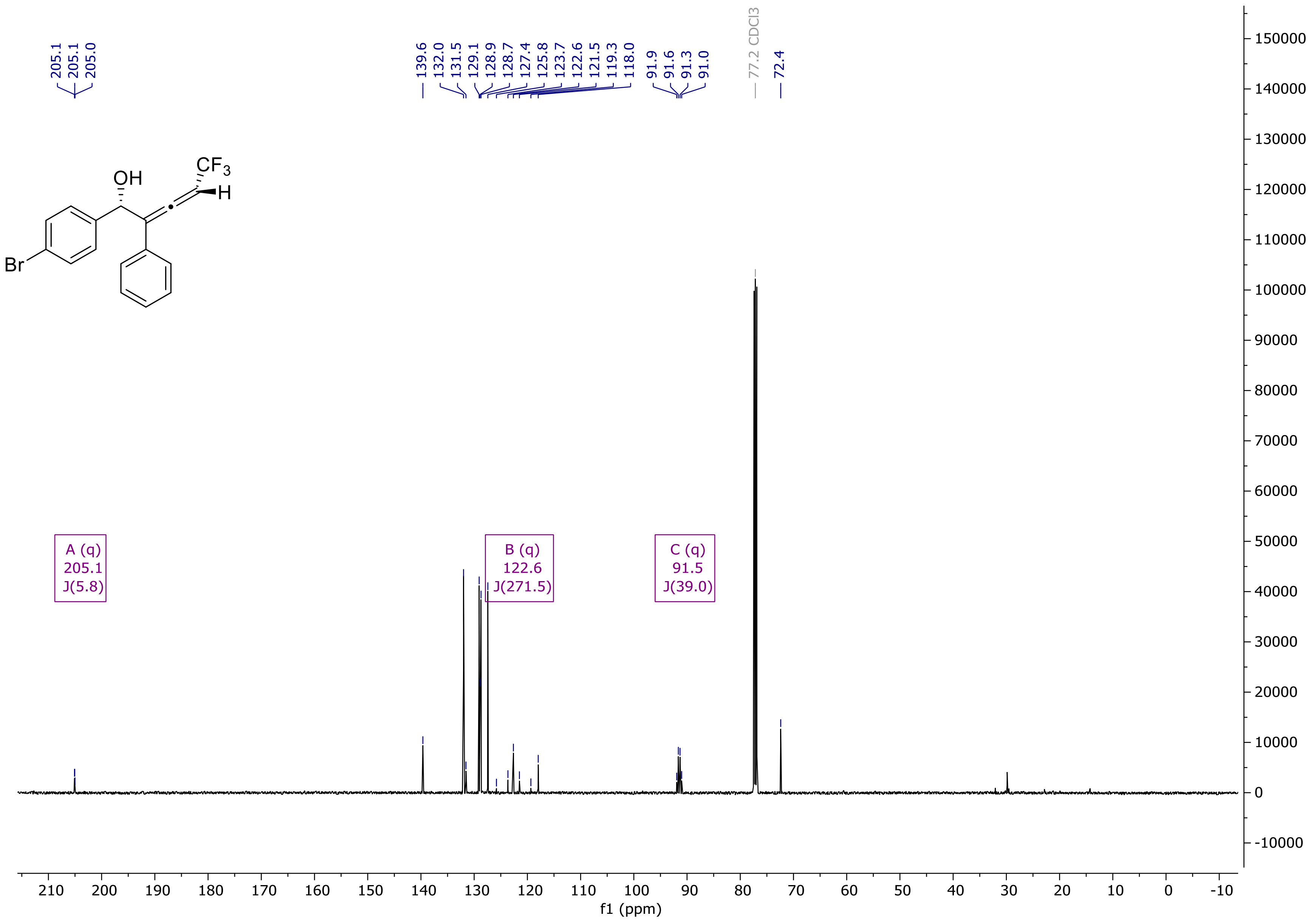

^{19}F NMR (377 MHz, $CDCl_3$) of compound **10c**

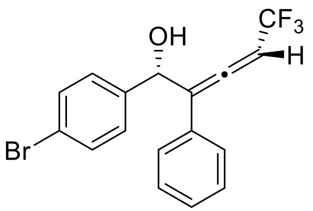
OH
CF3
H
Br

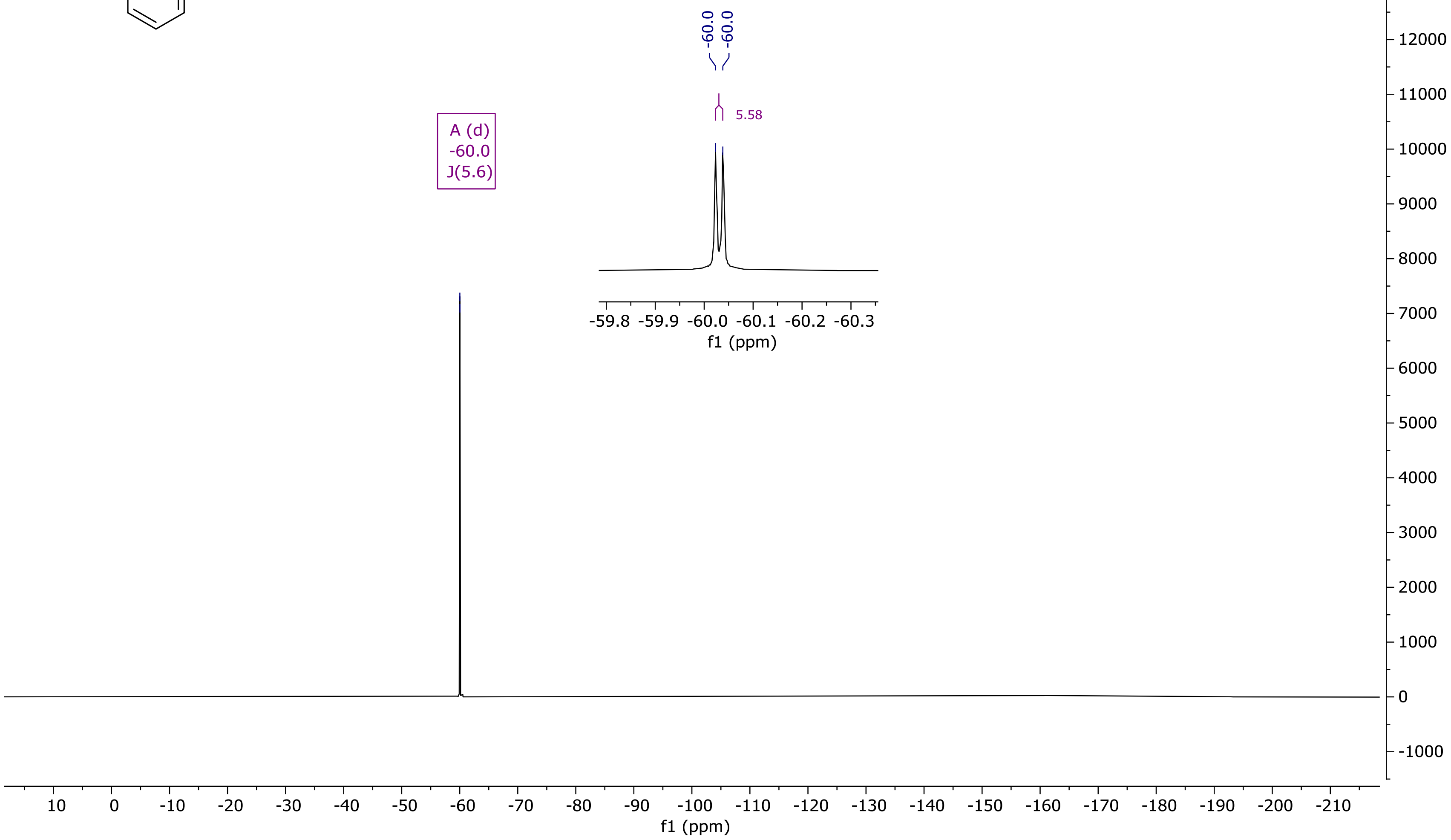
-60.0
-60.0
A (d)
-60.0
J(5.6)
5.58
-59.8 -59.9 -60.0 -60.1 -60.2 -60.3
f1 (ppm)
16000
15000
14000
13000
12000
11000
10000
9000
8000
7000
6000
5000
4000
3000
2000
1000
0
-1000
10 0 -10 -20 -30 -40 -50 -60 -70 -80 -90 -100 -110 -120 -130 -140 -150 -160 -170 -180 -190 -200 -210
f1 (ppm)
